# Homochiral Self‐Sorting During Macrocycle Formation and their Chiroptical Properties

**DOI:** 10.1002/open.202400400

**Published:** 2024-11-26

**Authors:** Diptiprava Sahoo, Soumen De

**Affiliations:** ^1^ School of Chemistry Indian Institute of Science Education and Research Thiruvananthapuram (IISER-TVM) 695551 Thiruvananthapuram India

**Keywords:** Chirality, Self-Sorting, Macrocycle, Imine bond, Chiroptical properties

## Abstract

Several BINOL‐derived C2‐symmetric aldehydes were synthesized to investigate chiral self‐sorting phenomena during macrocycle formation in the presence of aliphatic and aromatic bisamines. While self‐sorting was unsuccessful with aliphatic amines, aromatic amine dictated complete homochiral self‐sorting, confirmed by ^1^H NMR analysis and molecular modelling. Additionally, the impact of macrocyclization on the chiroptical properties of these macrocycles was examined. The Cotton effects band red‐shifted for aromatic amines owing to extended conjugation. Notably, a substantial increase in specific rotation was observed upon macrocycle formation.

## Graphical abstract

Imine condensation of C2‐symmetric chiral aldehydes with aliphatic amines results in 2 : 2 macrocycles lacking self‐sorting, whereas aromatic amines yield macrocycles with complete homochiral self‐sorting. These macrocycles exhibit unique chiroptical properties attributed to rigidification compared to the starting aldehydes.

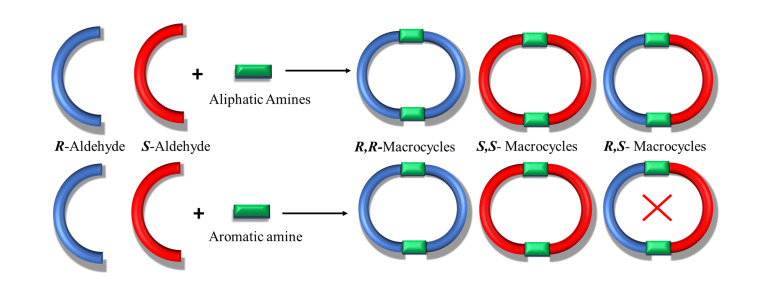

## Introduction

Self‐sorting^[1]^ is the ability of a system to spontaneously organize its components into distinct groups or categories based on the specific information encoded within them. For high‐fidelity self‐recognition, the interactions between the building blocks must be reversible to allow effective error correction. This phenomenon is widely observed across various natural and artificial systems, from the formation of galaxies and biological self‐assembly to synthetic molecular and supramolecular architectures.^[1]^ As such, self‐sorting is a key principle in developing complex architectures with diverse functionalities. Understanding self‐sorting offers valuable insights into how order and complexity arise from seemingly random processes.

Chiral self‐sorting^[2]^ is one of the subsets of “self‐sorting” and is particularly common in biological systems as most bio‐molecules are homochiral.^[3]^ However, it is still debated how and why one particular enantiomer of amino acids (L) and sugars (D) was favoured in proteins and DNA. Furthermore, cell compartmentalisation, a crucial phenomenon in life, may reflect an intricate illustration of self‐sorting (chiral). Inspired by these homochiral biotic assemblies, chiral self‐sorting^[2]^ has been drawing growing importance in recent years to understand these processes more deeply. Chiral self‐sorting is a process where racemic components are spontaneously organized into homo‐ or heterochiral assemblies over statistical distribution. Chiral self‐sorting could be classified into social and narcissistic chiral self‐sorting. Social self‐sorting^[1]^ happens when chiral molecules undergo self‐discrimination, leading to heterochiral self‐assembly (the assembly of molecules with opposite chirality). In contrast, narcissistic self‐sorting involves self‐recognition, resulting in homochiral assembly (the assembly of molecules with the same chirality). Chiral self‐sorting plays a crucial role in creating highly pure, specific chiral self‐assembled structures and has potential applications in chiral molecular switches, molecular machines, and host‐guest chemistry.^[4]^

Most of the reported self‐sorting relies on non‐covalent interactions such as π–π stacking,^[5]^ hydrogen bonding,^[6]^ metal‐ligand interaction,^[7]^ reversible covalent bond formation^[8]^ etc. We exploit reversible imine^[9]^ bond formation to realize homochiral self‐sorting to construct macrocyclic architectures. The reversible nature of imine bond formation offers a self‐amendment mechanism and thus provides better control over high selectivity and efficiency in the product distribution under thermodynamic control. Recently, Moore,^[8d]^ Mastalerz,^[10]^ and others^[11]^ have reported highly selective fabrications of homochiral macrocycles and cages. In one of their studies, Moore and co‐workers^[8a]^ synthesized a BINOL‐based C2 symmetric alkyne. When the racemic mixture of this alkyne was subjected to an alkyne metathesis reaction, they observed the formation of homochiral macrocycles, with no heterochiral macrocycles forming. Based on computational modelling and thermodynamic analysis, the authors concluded that the preference for homochiral macrocycle formation over heterochiral ones is due to the higher entropy associated with the highly symmetric (D2) homochiral macrocycles compared to the lower symmetry of the heterochiral macrocycles. In spite of the reversible nature of the bond, achieving quantitative self‐sorting in solution poses a considerable challenge due to the lack of a resilient preference for either hetero‐chiral or homo‐chiral assemblies. Sometimes, the use of templates improves selectivity.^[12]^ Herein, we demonstrated complete homochiral self‐sorting using BINOL‐derived C2‐symmetric bis aldehyde and diamine to fabricate several homochiral macrocycles and investigated their chiroptical properties.

## Results and Discussion

### Synthesis and Design

**Figure 1:**
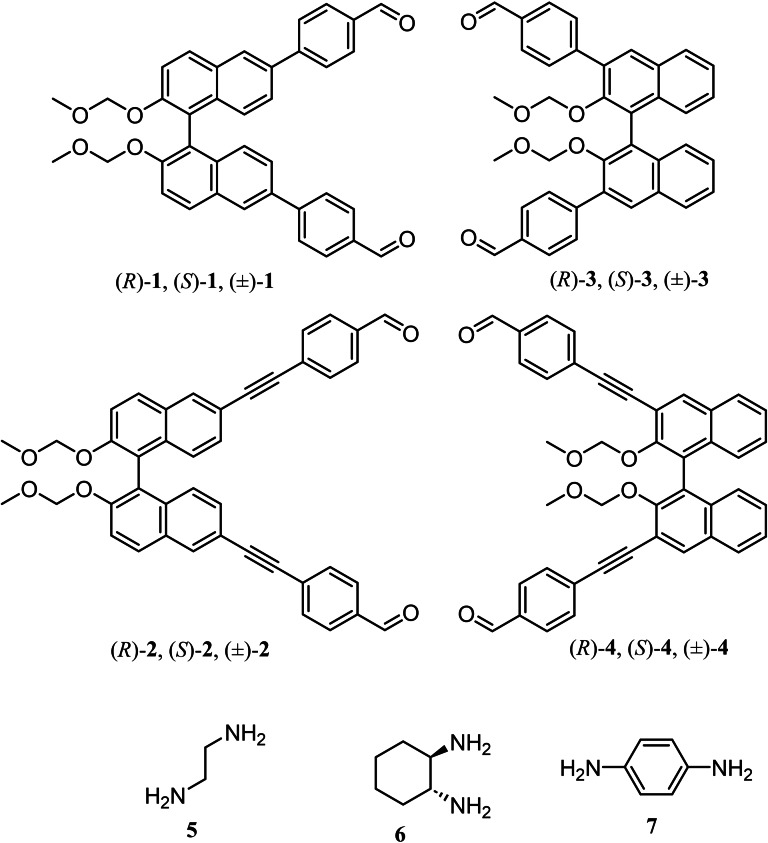
Structure of the aldehydes and amines used in this study.

We chose reversible imine bond formation as a tool to make the macrocycles. Accordingly, we synthesized four BINOL‐based aldehydes (*R*, *S* and racemic) to establish chiral self‐sorting via imine bond formation (Figure 1 and S1). Aldehydes **1** and **3** were synthesized by Suzuki coupling with halogenated BINOL and 4‐formylphenyl boronic acid in the presence of Pd(0) as a catalyst (Scheme S1). The longer aldehydes **2** and **4** were synthesized by Sonogashira coupling between halogenated BINOL and 4‐ethynylbenzaldehyde in the presence of Pd(0), CuI and triethylamine (Scheme S1). All the aldehydes were fully characterized by ^1^H NMR, ^13^C NMR, UV‐Vis, CD spectroscopies and mass‐spectrometry (Figure S2 to S20). For example, the peak at 10.05 ppm in ^1^H NMR for the aldehyde functional group, along with the presence of seven sets of aromatic protons and diastereotopic CH_2_ protons at 5.05 and 5.15 ppm (Figure S2), confirms the presence of aldehyde **1**. Furthermore, the presence of a peak at m/z 582.2041 in the HRMS (Figure S5) corroborates the formation of aldehyde **1**. Additionally, the formation of aldehyde **2** was established from a single crystal^[13]^ structure analysis (Figure S14).

**Scheme 1:**
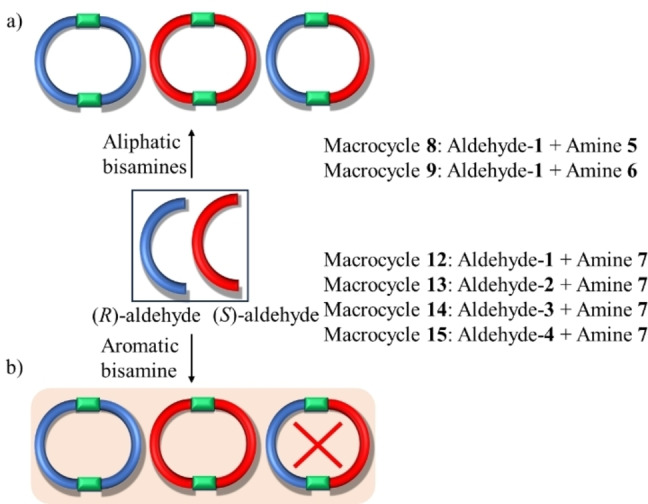
Cartoon representation of homochiral self‐sorting a) from aliphatic amines and b) from aromatic amine during macrocycle formation.

With the aldehydes in hand, we examined the chiral self‐sorting using aliphatic amines and aromatic amine during macrocycle formation (Scheme 1). Before investigating the chiral self‐sorting process during the macrocycle formation using racemic aldehydes and ethylene diamine, we first optimized the formation of enantiopure macrocycle (2 : 2) from enantiopure aldehyde and ethylenediamine (Figure S21 to S25). Therefore, we mixed aliphatic bis amine, i. e. ethylene diamine and aldehyde *(R*)‐**1** with CDCl_3_ in a 1 : 1 ratio in an NMR tube at 50 °C.

**Figure 2:**
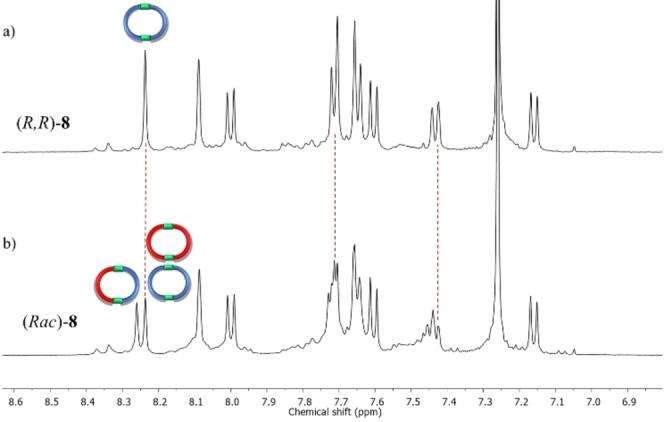
Partial ^1^H NMR comparison of macrocycle **8** obtained from a) (*R*)‐**1** and **5**; b) (*±*)‐**1** and **5**. The new imine‐derived peak in the bottom spectrum corresponds to (*R*,*S*)‐**8**. This experiment suggests there is no preference for homochiral or heterochiral self‐sorting.

The ^1^H NMR spectrum of the reaction mixture exhibited one imine signal at 8.24 ppm along with one set of the other aromatic peaks, signifying the formation of the macrocycle **8** (Figure 2a and S21). Moreover, detecting a peak at m/z 1213.5167 in the ESI‐TOF spectrum confirmed the macrocycle‘s formation (Figure S22). This peak, corresponding to [M+H]^+^, exhibited an isotopic distribution pattern consistent between experimental and theoretical spectra.

Next, (*±*)‐**1** was mixed with ethylene diamine, instead of (*R*)‐**1**, in the same stoichiometric ratio during the macrocyclization to probe the chiral self‐sorting phenomenon. The resulting reaction mixture showed two imine‐derived signals at 8.26 and 8.24 ppm in a 1 : 1 ratio, unlike one in the case of enantiopure aldehyde in the ^1^H NMR spectrum (Figure 2b and S26). A comparison of the ^1^H NMR of the macrocycles derived from (*±*)‐**1** and (*R*)‐**1** suggested that imine derived peak at 8.24 ppm corresponds to the (*R,R*)/(*S,S*) macrocycle, and the other peak at 8.26 ppm is due to the (*R,S*) macrocycle. The 1 : 1 integration ratio of the imine‐derived peaks indicates that there is no preference for either homochiral or heterochiral chiral self‐sorting during macrocycle formation with aliphatic diamine. Furthermore, when two independently prepared homochiral macrocycles with opposite chirality were mixed and heated at 50 °C for 2 hours, dynamic exchange took place. The final NMR spectrum resembled that obtained when racemic aldehydes were reacted with ethylene diamine (Figure S27).

Next, in order to understand the effect of rigidity and chirality in the aliphatic amine on the self‐sorting process, we took chiral cyclohexane diamine instead of ethylene diamine. Similar to the previous case, we first optimized the macrocycle synthesis with (*R*)‐**1** and amine (*R*,*R*)‐**6** by heating in an NMR tube. The presence of one imine‐derived peak at 8.23 ppm validated the macrocycle formation (Figure S23). Furthermore, the molecular ion peak at 1321.6011 Dalton, with a matching isotopic distribution pattern between experimental and theoretical spectra, in ESI‐TOF corroborated the formation of the desired macrocycle (Figure S24). Additionally, the formation of the macrocycle between (*R*)‐**1** and amine (*S*,*S*)‐**6** was verified by the emergence of an imine peak at 8.27 ppm in the ^1^H NMR spectrum (Figure S25).

To examine the sorting process, we utilized one equivalent of (*±*)‐**1** in conjunction with one equivalent of (*R*,*R*)‐**6**. Notably, in this scenario, the (*R,R*) and (*S,S*) macrocycle was favoured over the (*R,S*) macrocycle (only the aldehyde chirality is mentioned for the macrocycle, not for the amine), as observed by comparing the ^1^H NMR spectra (Figure S28). Through integration analysis of the desired imine peak compared to other imine peaks, it was found that there was up to 68 % homochiral self‐sorting. So, increasing the rigidity of amine helps to improve the degree of homochiral self‐sorting.

**Figure 3:**
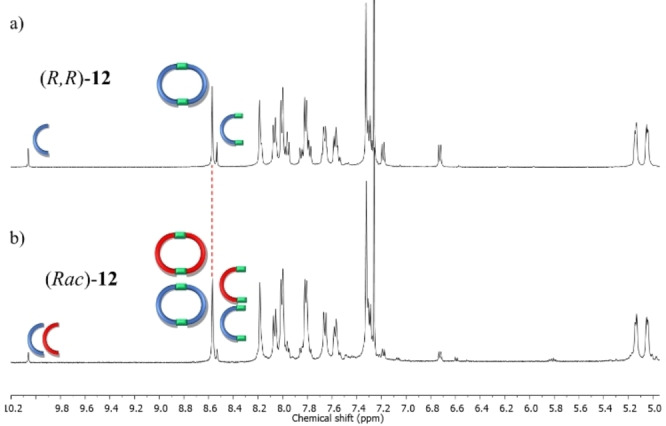
Partial ^1^H NMR comparison of macrocycle **12** obtained from a) (*R*)‐**1** and **7**; b) (*±*)‐**1** and **7**. The small imine‐derived peak at 8.53 ppm belongs to half of the macrocycle **16**. The presence of one set of sharp imine‐derived signals indicates the formation of a homochiral macrocycle (*R*,*R*)‐**12** only.

Next, we turn our attention to aromatic bisamine for chiral self‐sorting. Like aliphatic bisamine, we first established the synthesis of the enantiopure macrocycle from (*R*)‐**1** and 1,4‐phenylene‐amine **7**. When enantiopure (*R*)‐**1** was employed for condensation reaction at room temperature in the presence of a catalytic amount of acetic acid, two sets of imine signals were observed after 48 hours in the NMR spectrum (Figure S29 to S32). The peak at 8.53 ppm was attributed to half of the macrocycle (prepared independently by heating with excess **7**, Figure S53 and S31), and the peak at 8.57 ppm corresponds to the full macrocycle. Unfortunately, the reaction was not completed even after two days at room temperature. In contrast, if the reaction was carried out in refluxing conditions, the peak for half of the macrocycle was diminished, and the peak for the full macrocycle increased, suggesting the selective formation of the enantiopure macrocycle (Figure 3a). Notably, only half of the macrocycle was observed as intermediate; no other intermediates were observed in the NMR spectrum. A similar NMR spectrum was observed when the other three aldehydes (**2**, **3**, and **4**) were used for macrocycle formation (Figure S34 to S44). In all the cases, like before, the corresponding macrocycles (**13**, **14**, **15** respectively) were observed predominantly along with a small amount of the corresponding half of the macrocycle under a similar reaction condition (Figure S54 to S56).

However, we were unable to detect the molecular ion peaks of the macrocycles in the mass spectra, likely due to poor ionization or the presence of significant amounts of fragmented products under the mass spectrometry conditions (Figures S33, S37, S41, and S45). Consequently, the presence of other oligomers (e.g., 3+3, 4+4) cannot be definitively excluded based on the mass/NMR spectra.

After establishing the formation of the enantiopure macrocycle, we investigated the self‐sorting process under identical conditions. Surprisingly, unlike the results with aliphatic amines, refluxing a 1 : 1 mixture of racemic BINOL‐aldehyde **1** and diamine **7** in chloroform for 48 hours produced two distinct imine signals at δ=8.57 ppm and 8.53 ppm. The peak at 8.57 ppm was unequivocally assigned to a racemic mixture of the enantiopure macrocycle by comparing it with the chemical shifts of the previously prepared homochiral macrocycle from the pure R/S enantiomer (Figure 3b and S46). The peak at δ=8.53 ppm was attributed to half of the macrocycle **16** (Figure S53). Therefore, the ^1^H NMR study indicates complete homochiral self‐sorting with aromatic bisamine. Similarly, other three aldehydes were also used to further validate the self‐sorting during macrocycle formation. In each case, two imine‐derived peaks were observed (8.52 and 8.49 ppm for macrocycle **13**, 8.61 and 8.56 ppm for macrocycle **14**, and 8.53 and 8.49 ppm for macrocycle **15**). These results confirm the formation of only two homochiral enantiomers – (*R,R*)‐**13** and (*S,S*)‐**13**, (*R,R*)‐**14** and (*S,S*)‐**14**, and (*R,R*)‐**15** and (*S,S*)‐**15** – along with the corresponding half of the macrocycle (Figures S48 to S50).

**Figure 4:**
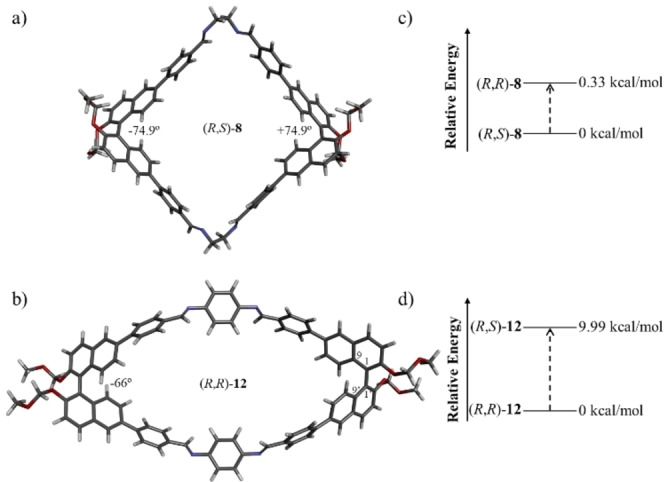
Geometry optimized structures of most stable isomer of a) **8** and b) **12**. Relative energies of diastereomeric macrocycles c) (*R*,*R*)‐**8** and (*R*,*S*)‐**8**; d) (*R*,*R*)‐**12** and (*R*,*S*)‐**12**. All the calculations were carried out at B3LYP/6‐311G level of theory. The calculations predict a strong preference for homochiral self‐sorting with aromatic amines but no preference for aliphatic macrocycles.

Efforts to grow crystals for these macrocycles to gain deeper insights into the structures and self‐sorting phenomenon were unsuccessful. Consequently, DFT calculations were conducted using Gaussian to delve deeper into the structural parameters and stability disparities between the diastereomers of the macrocycles to elucidate the variation in the degree of self‐sorting between aliphatic and aromatic amines (Figure S57 to S71). A meticulous analysis of the DFT findings unveils that the dihedral angle between the two naphthyl units (9‐1‐1’‐9’) in macrocycles (*R*,*R*)‐**8** and (*R*,*S*)‐**8** is −85.8° and −74.9°/+74.9° (Figure 4a and S57–S58), respectively. In contrast, the binaphthyl units in the aromatic bisamine macrocycle **12** are positioned in a more constrained environment. The dihedral angle between the two naphthyl units in (*R*,*R*)‐**12** and (*R*,*S*)‐**12** is approximately ‐66° and +65.8°/‐65.8°, respectively (Figure S60–61), while the dihedral angle between the naphthyl units in **1** (by comparing the crystal structure of **2**, in which the dihedral angle is of ‐101°) is around 100°. This indicates that macrocyclization reduces the dihedral angle, resulting in a more rigid conformation.

Crucially, the DFT results reveal a minimal energy difference (0.33 kcal/mol) between the two diastereomers (*R*,*R*)‐**8** and (*R*,*S*)‐**8** (Figure 4c and S59), indicating no discernible preference for either homochiral or heterochiral self‐assembly, mirroring the experimental observations. Conversely, it was noted that (*R*,*R*)‐**12** exhibits considerably greater stability (9.99 kcal/mol) than *R*,*S*‐**12** (Figure 4d and S62), signalling a pronounced preference for homochiral self‐sorting over heterochiral self‐sorting. DFT calculations for the remaining macrocycles similarly reveal that the homochiral macrocycles are more stable than their heterochiral counterpart (Figures S65, S68 and S71).

After establishing the self‐sorting, we checked the effect of rigidification and conjugation via macrocylization on the CD spectra (Figures S72 to S85). The CD spectrum of (*R*)‐**1** exhibits two distinct signals (Figure S7): one showing a bisignate pattern (positive cotton effect) spanning from 220 to 250, indicative of (*R*)‐BINOL, and another appearing from 275 to 350, representing the phenyl aldehyde moiety. Upon forming the macrocycle with ethylene diamine, a notable change in the intensity of the band corresponding to the phenyl aldehyde is observed, probably due to the increase in rigidity. On the other hand, CD amplitude at the BINOL region also changes, likely due to a change in the dihedral angle caused by macrocycle formation (Figure S72 and S73). The theoretical calculation also suggests a decrease in dihedral angle upon macrocycle formation. Likewise, employing a chiral cyclohexane diamine yielded analogous results in the CD spectrum (Figure S74 to S77).

**Figure 5:**
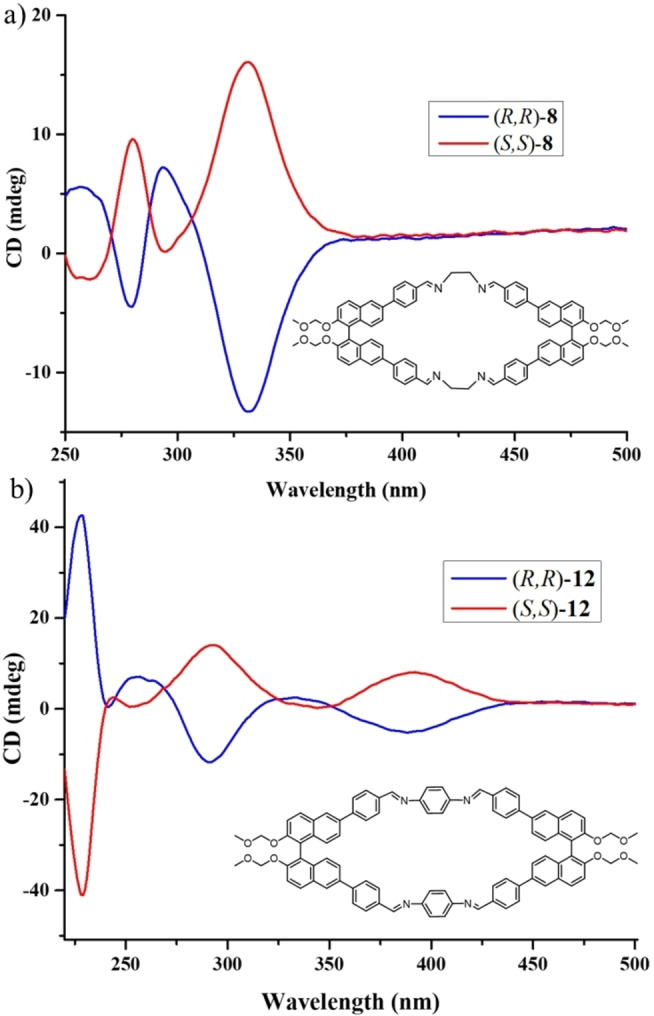
CD spectra of macrocycles a) (*R*,*R*)‐**8** (blue) and (*S*,*S*)‐**8** (red) (DCM, 10^−4^ M, 298 K); b) (*R*,*R*)‐**12** (blue) and (*S*,*S*)‐**12** (red) (THF, 5×10^−5^ M, 298 K).

The CD spectra of the macrocycles derived from aromatic bisamine are distinctly different from aliphatic amine‐derived macrocycles. For example, in the case of macrocycle **12**, a red‐shifted band emerges in the 350 to 450 regions, attributed to the increase in conjugation via imine bond formation indirectly indicating macrocyclization (Figure S78). Like the aliphatic amine‐derived macrocycle, the CD amplitude at the BINOL range also changes, indicating an alteration in dihedral angle due to macrocyclization (Figure S79). Notably, the CD spectrum derived from (*S*)‐**1** revealed a mirrored image relationship with the macrocycle derived from (*R*)‐**1** (Figure 5b). Furthermore, investigating all three aldehydes and their macrocycle formation with the aromatic amine consistently revealed a red‐shifted band corresponding to the newly introduced phenyl group from the amine upon macrocycle formation and reduction of CD amplitude in the BINOL regions (Figure S80 to S85). We also investigated the effect of macrocycle formation on the specific rotations. Notably, the specific rotation increased in all cases compared to free aldehyde (Table 1 and S1), which may be due to a decrease in dihedral angle and an increase in rigidity caused by macrocycle formation.

**Table 1:**
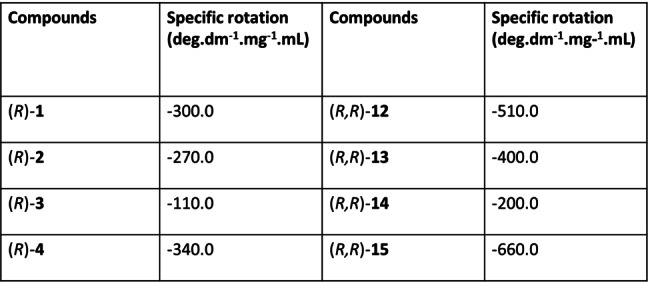
Specific rotations of aldehydes and aromatic macrocycles.

## Conclusions

In conclusion, we have explored chiral self‐sorting during macrocycle formation by condensation of chiral C2‐symmetric aldehydes with aliphatic or aromatic bisamine. Our findings indicate that while aliphatic amines do not exhibit a preference for either homochiral or heterochiral self‐sorting, aromatic bisamines notably favour homochiral self‐sorting, likely due to increased rigidity of aromatic macrocycles and due to the higher entropy associated with the highly symmetric (D2) homochiral macrocycles compared to the lower symmetry of the heterochiral macrocycles (C2h).^[8a,d]^ The enantiopure macrocycles were characterized by ^1^H NMR spectroscopy, CD spectroscopy and mass spectrometry.

The confirmation of homochiral self‐sorting was unequivocally established by comparing the ^1^H NMR spectra of macrocycles obtained from enantiopure and racemic aldehydes. Additionally, DFT calculations supported the notion of homochiral self‐sorting by indicating the superior thermodynamic stability of homochiral macrocycles. Furthermore, we examined the impact of macrocyclization on CD spectra and specific rotation. This investigation lays the groundwork for the development of 3D chiral cages and interlocked molecules based on reversible imine bond formation,^[14]^ a project currently underway in our laboratory and slated for future reporting.

## Conflict of Interests

The authors declare no conflict of interest.

## Supporting information

As a service to our authors and readers, this journal provides supporting information supplied by the authors. Such materials are peer reviewed and may be re‐organized for online delivery, but are not copy‐edited or typeset. Technical support issues arising from supporting information (other than missing files) should be addressed to the authors.

Supporting Information

## Data Availability

The data that support the findings of this study are available in the supplementary material of this article.
