# Supplementary material for: Homochiral Self‐Sorting During Macrocycle Formation and their Chiroptical Properties
Source: ChemistryOpen. 2024 Nov 26;14(6):e202400400. doi: 10.1002/open.202400400 (PMC13062943; doi:10.1002/open.202400400)
Supplement: Supplementary file 1 — Supporting Information [file OPEN-14-e202400400-s001.pdf]

ChemistryOpen

Supporting Information

Homochiral Self-Sorting During Macrocycle Formation and their Chiroptical Properties

Diptiprava Sahoo and Soumen De*

Supporting Information

for

Homochiral Self-Sorting During Macrocycle Formation and Their Chiroptical Properties

Diptiprava Sahoo and Soumen De*

School of Chemistry, Indian Institute of Science Education and Research Thiruvananthapuram (IISER-TVM), Thiruvananthapuram 695551, India

Email: soumende@iisertvm.ac.in

1 Contents

1 General Information and methods: 2
2 List of ligands and Synthetic Schemes: 4
3 Synthesis and Characterization 9
3.1 Synthesis of 1: 9
3.2 Synthesis of 2: 13
3.3 Synthesis of Aldehyde 4: 17
3.4 Synthesis of macrocycles from aliphatic bisamine 21
3.4.1 General procedure for the synthesis of the macrocycle 21
3.4.2 Macrocycle 8 21
3.4.3 Macrocycle 9 23
3.5 Self-sorting during macrocycle formation from aliphatic bisamine 27
3.6 Synthesis of macrocycles from aromatic amine 29
3.6.1 General procedure for the synthesis of macrocycle 29
3.6.2 Macrocycle 12 30
3.6.3 Macrocycle 13 33
3.6.4 Synthesis of macrocycle 14 36
3.6.5 Synthesis of macrocycle 15 39
3.7 Self-sorting during macrocycle formation from aromatic bisamine 42
3.8 Synthesis of half of the macrocycle 47
3.9 DFT optimized structures: 54
3.10 CD spectra of macrocycles 62

3.11 Specific Rotation of aldehydes and macrocycles: 75
3.12 Coordinates for crystal structure:..76
3.13 Coordinates for DFT optimized structures:...82

1 General Information and methods:

Commercial reagents were purchased from Sigma-Aldrich, TCI, Spectrochem, Avra or BLD Pharma and were utilised without additional purification unless stated otherwise. All solvents were distilled prior to use for column chromatography. Anhydrous tetrahydrofuran (THF) was distilled over sodium/benzophenone, while triethyl amine and chloroform were dried over calcium hydride. Thin-layer chromatography was performed using thin-layer chromatography plates from Merck (silica gel 60 F254). Silica gel 60 was used as a stationary phase for column chromatography.

NMR Spectroscopic Studies:

Nuclear magnetic resonance (NMR) spectra were acquired on a Bruker Advance III 500 MHz NMR spectrometer, with the deuterated solvent serving as the lock and the residual solvent as the internal reference. Chemical shift (δ) values are reported in ppm relative to tetramethylsilane (TMS), while the residual ^{1}H and ^{13}C signals of the deuterated solvent served as an internal standard ($CDCl_3$: δ H 7.26 ppm, δ C 77.16 ppm).

For ^{1}H NMR assignments, the chemical shifts (in ppm) are provided first, followed by the multiplicity of the signal in brackets (s: singlet, d: doublet, t: triplet, dd: doublet of doublets, m: multiplet), the value of coupling constants in Hertz if applicable, the number of protons involved, and, whenever possible, the assignment of the proton. The carbon atom numbering indicated in the experimental section is solely used for NMR assignments and may not adhere to IUPAC nomenclature rules.

UV–vis Spectroscopic Studies:

UV–vis spectra were obtained using a Shimadzu UV-3600 Vis-NIR spectrophotometer at room temperature, utilizing a quartz cuvette with a path length of 2 mm. Solutions for UV–vis analysis were freshly prepared by dissolving isolated compounds in UV-grade solvents unless specified otherwise.

CD Spectroscopic Studies:

Circular Dichroism (CD) spectra were obtained using a JASCO J-815 CD spectropolarimeter equipped with a Peltier-controlled thermostatic cell holder. CD measurements were conducted in a quartz cuvette with a path length of 2 mm.

Specific rotation measurement:

10 mg of sample was dissolved in 10 ml of chloroform and specific rotation was measured using JASCO polarimeter (P 2000) in a 100 mm Faraday cell. Na light source of wavelength 589 was used at 299 K to measure the specific rotation.

Single Crystal X-ray Diffraction (SCXRD) Analysis:

Data for the crystal were collected at 140 K on a Bruker Kappa diffractometer equipped with an APEXII CCD detector and Mo fine focus sealed tube source. All the data were collected by employing graphite monochromated MoKα radiation ($\lambda = 0.7107$ Å), and the data sets were processed using APEX II software. Integration of the data sets was carried out with the Bruker SAINT program. Structure solutions were performed using SHELXTS1 and refined employing SHELXLS1 through the OLEX2S2 program. Intensities were corrected for Lorentz and polarization effects, and an empirical absorption correction was applied using Blessing's method as incorporated into the program SADABS.S3 Non-hydrogen atoms were refined with anisotropic thermal parameters. The positions of the hydrogen atoms were calculated in the idealized positions unless otherwise noted. The molecular structures were rendered using MERCURY 3.10.3 software

Mass spectrometry:

Electrospray Ionization – Mass Spectrometric (ESI–MS) Analyses**:** High-resolution mass spectra (HRMS) were obtained using the Thermo Scientific™ Q Exactive™ Hybrid Quadrupole-Orbitrap Mass Spectrometer employing the electrospray ionization (ESI) technique. The calculated m/z values were reported as acquired from the ThermoXCalibur software.

Electrospray Ionization-Time Of Flight (ESI-TOF) Analyses**:** Waters Q-TOF mass spectrometer equipped with Z-spray source was used for the electrospray ionization (ESI) mass spectrometry measurement in positive mode.

Molecular modelling:

All the computations were conducted utilizing the Gaussian 16 program package.[1] The calculations were executed via the density functional theory (DFT) method employing the

restricted B3LYP (Becke's three-parameter hybrid exchange functional and the Lee-Yang-Parr correlation functional)[2] level, utilizing a 6-311G basis set.

2 List of ligands and Synthetic Schemes:

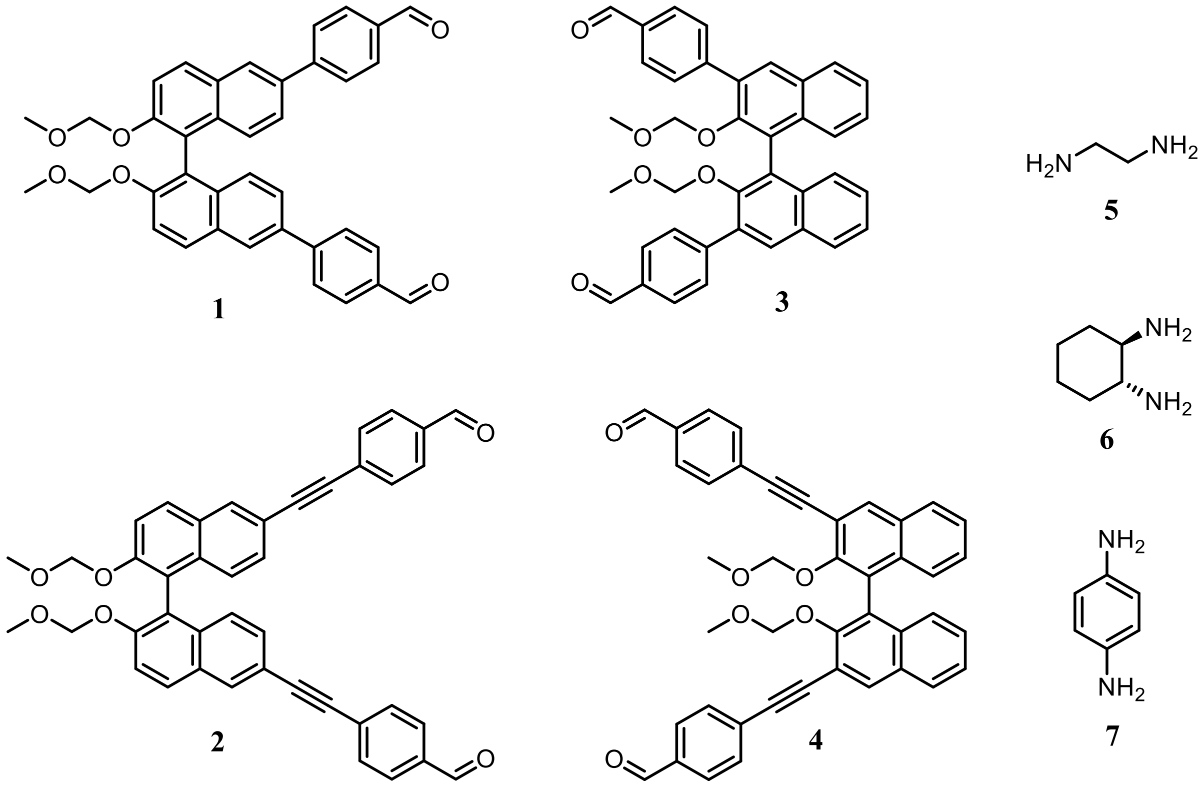

Figure S 1: List of ligands used in the study of chiral self-sorting.

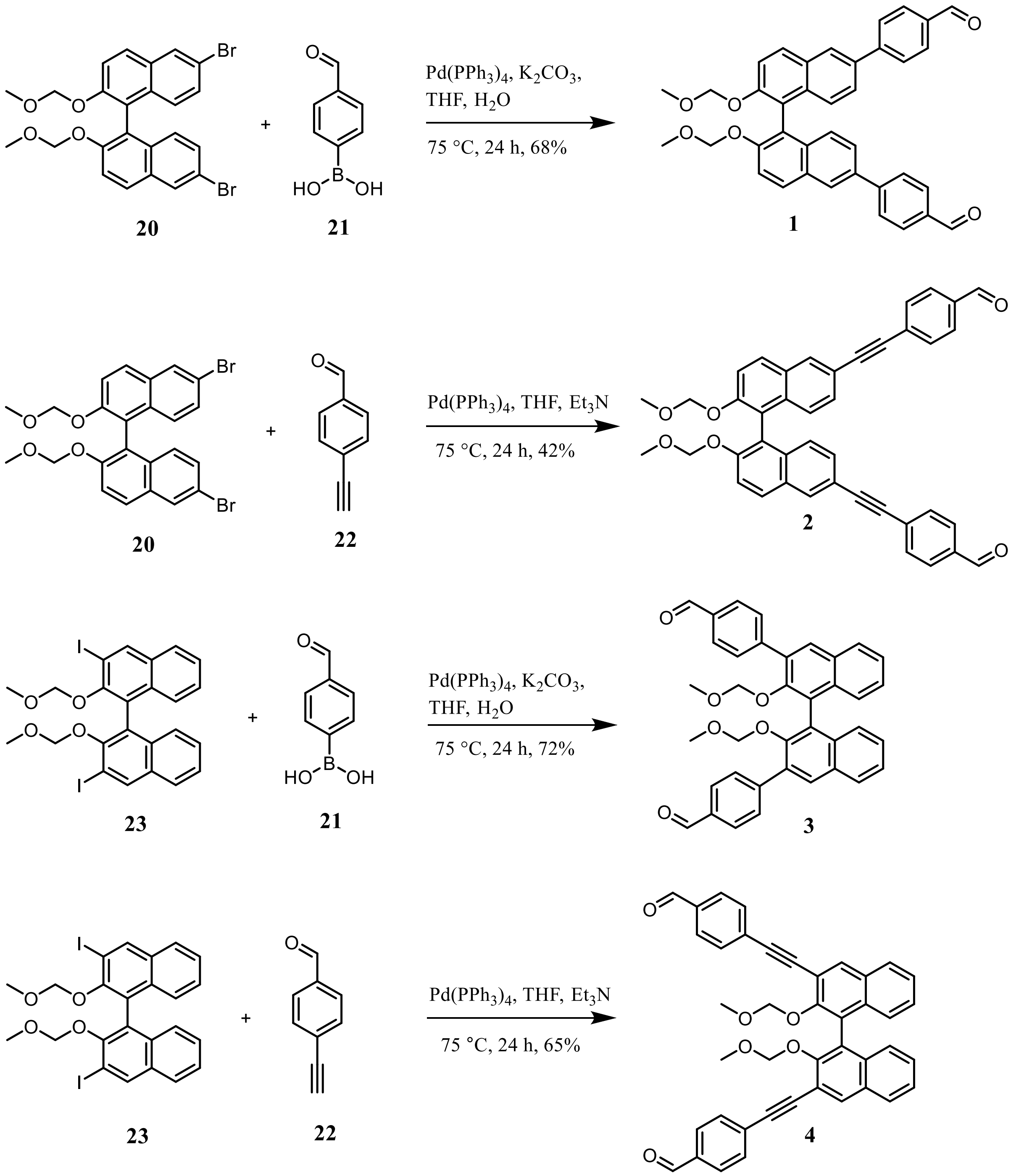

Scheme S 1: Synthetic schemes to prepare the C2-symmetric BINOL-derived aldehydes.

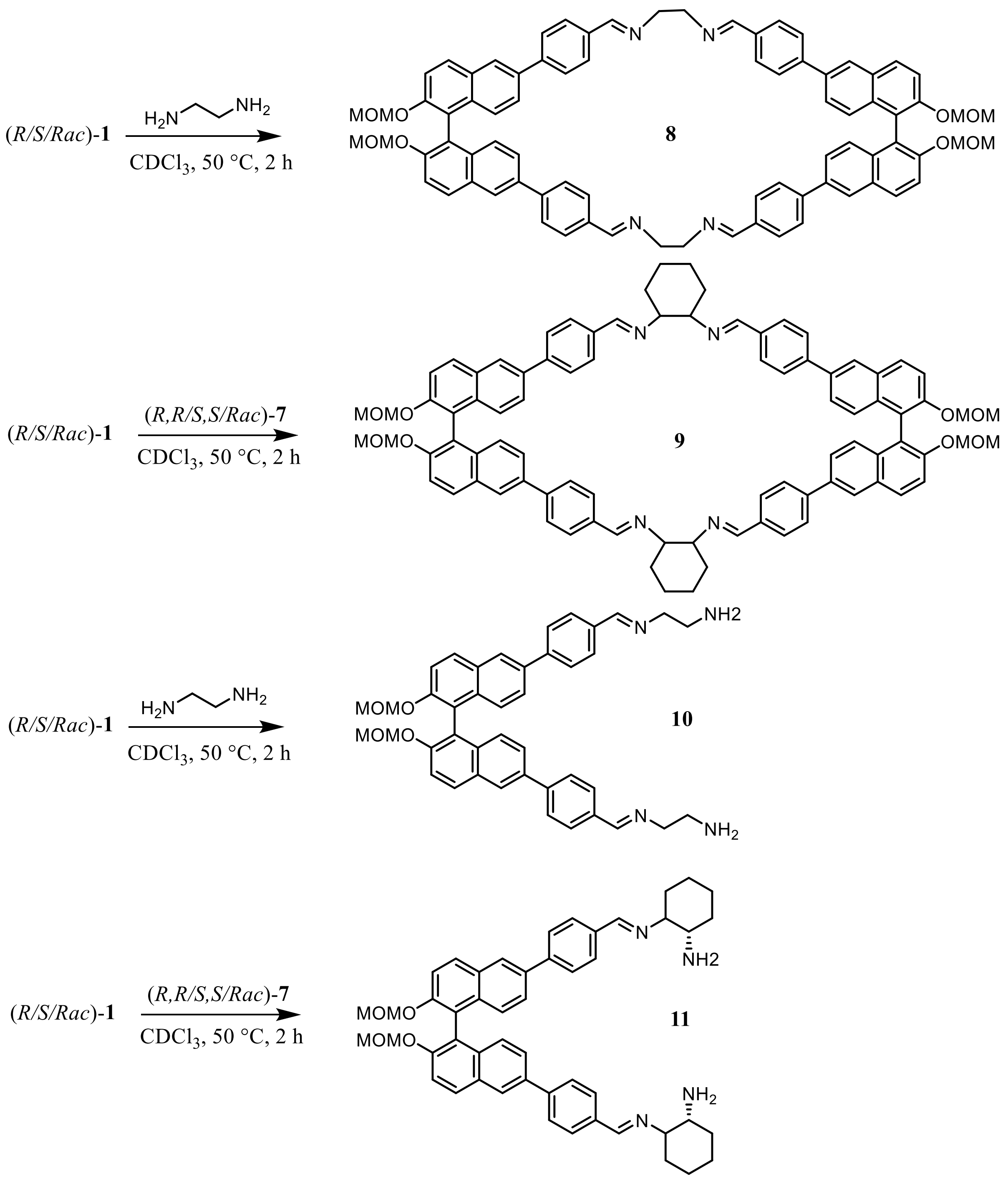

Scheme S 2: Synthetic schemes for the formation of aliphatic amine-based macrocycles and half-of-the macrocycles.

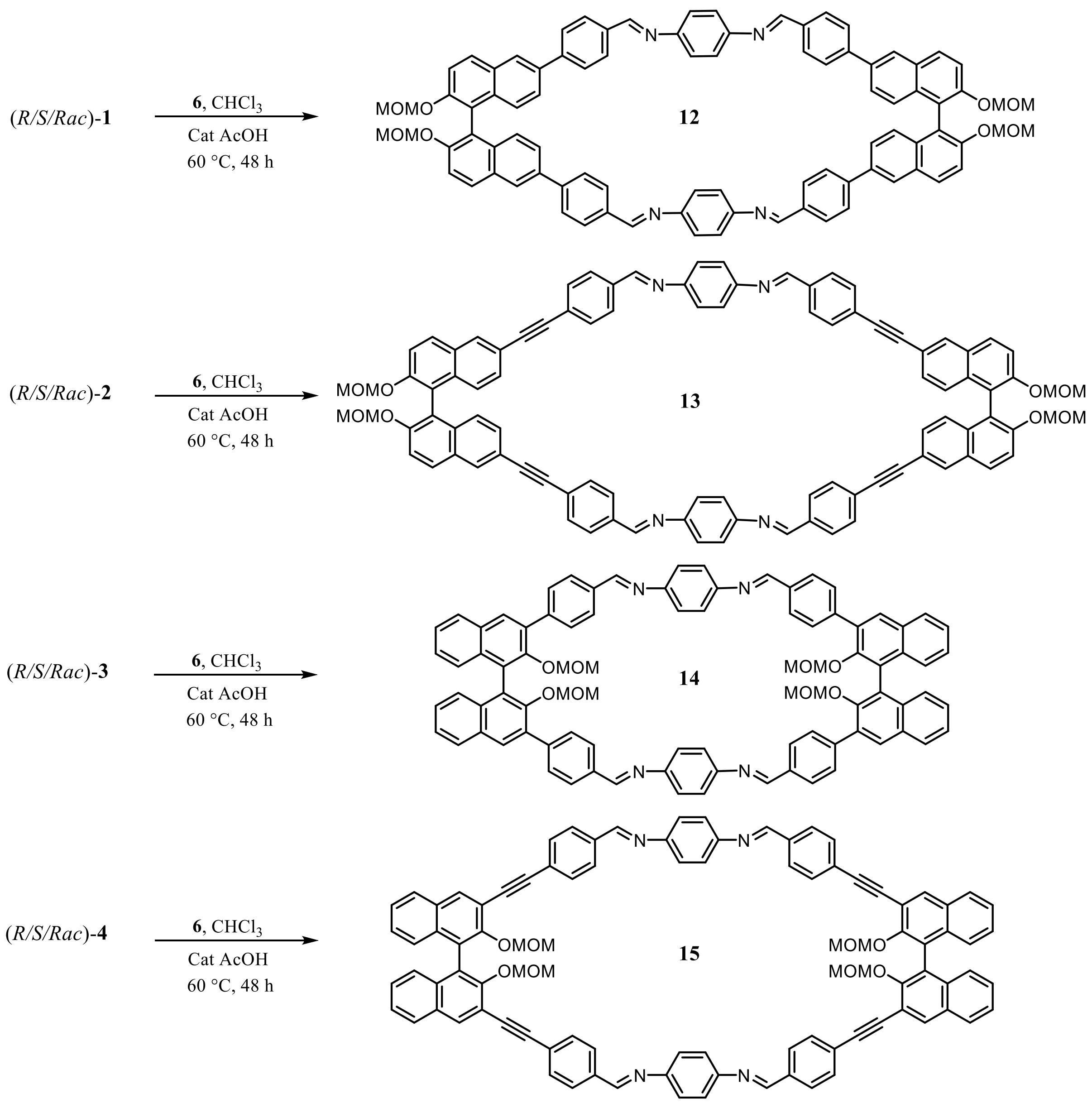

Scheme S 3: Synthetic schemes for the formation of the aromatic amine-based macrocycles.

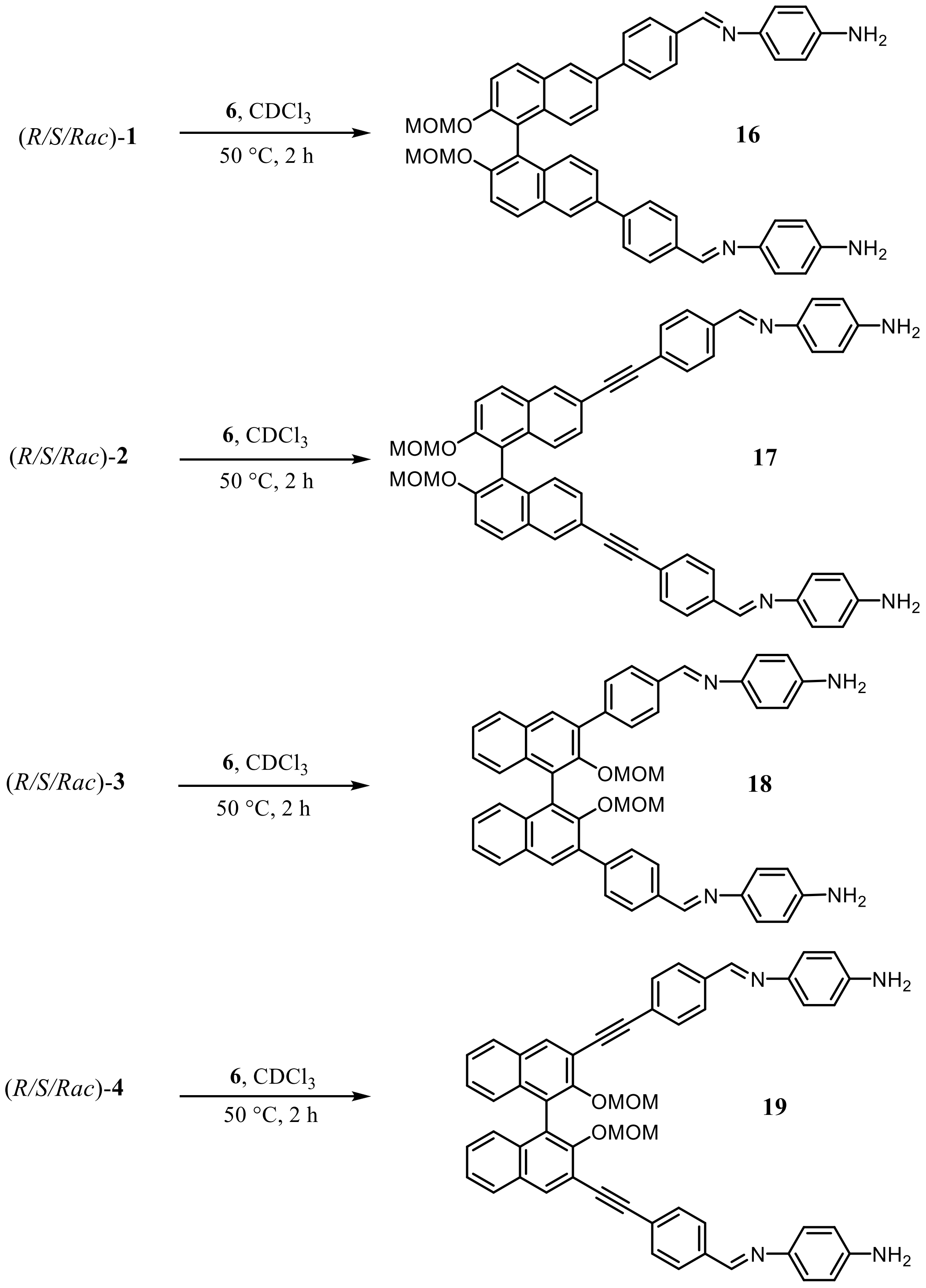

Scheme S 4: Synthetic scheme for the formation of half of the macrocycles.

3 Synthesis and Characterization

3.1 Synthesis of 1:

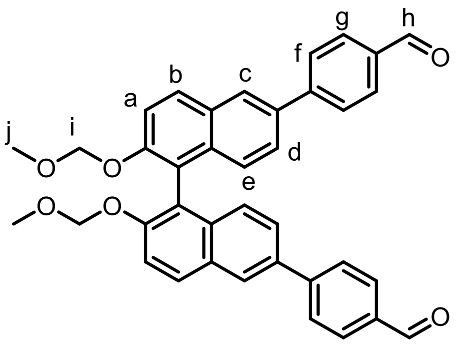

A 100 mL Schlenk tube was charged with compound **20**[3] (500 mg, 939 µmol), 4-formyl phenylboronic acid (310 mg, 2.07 mmol) and K_2CO_3 (780 mg, 5.65 mmol). THF (20 mL) and water (6 mL) were added to the mixture and purged with an argon air balloon for 30 minutes. The reaction mixture was then heated at 75 ˚C, $Pd(PPh_3)_4$ (75.0 mg, 64.9 µmol) was added, and the stirring was continued for 24 hours at the same temperature. After completing the reaction (monitored by TLC), the reaction mixture was cooled down to room temperature and extracted with ethyl acetate (2 × 50 mL). The organic layer was washed with water (2 × 50 mL) and brine (50 mL) and dried over sodium sulphate. After removing the solvent, the residue was purified by chromatography on silica gel, eluting with 30% ethyl acetate in hexane (R_f = 0.5 in 3:7 ethylacetate: hexane solvent) to furnish the desired product as a white crystalline solid.

Yield: 0.45 g (772 µmol, 82%).

^{1}H NMR (500 MHz, $CDCl_3$) δ 10.05 (s, 2 H, h-H), 8.17 (d, ^4J =1.9 Hz, 2 H, c-H), 8.06 (d, ^3J = 9 Hz, 2 H, b-H), 7.96 (d, ^3J = 8.3 Hz, 4 H, g-H), 7.84 (d, ^3J = 8.3 Hz, 4 H, f-H), 7.67 (d, ^3J = 9.2 Hz, 2 H, a-H), 7.54 (dd, ^3J = 9 Hz, ^4J = 1.5 Hz, 2 H, d-H) 7.28 (d, ^3J = 8.8 Hz, 2 H, e-H), 5.13 (d, ^2J = 6.8 Hz, 2 H, i-H), 5.04 (d, ^2J = 6.8 Hz, 2 H, i-H), 3.2 (s, 6 H, j-H) ppm.

^{13}C NMR (125 MHz, $CDCl_3$) δ 192.0, 153.5, 147.1, 135.3, 135.2, 133.9, 130.5, 130.3, 130.0, 127.8, 126.8, 126.5, 125.7, 120.9, 117.9, 95.2, 56.1 ppm.

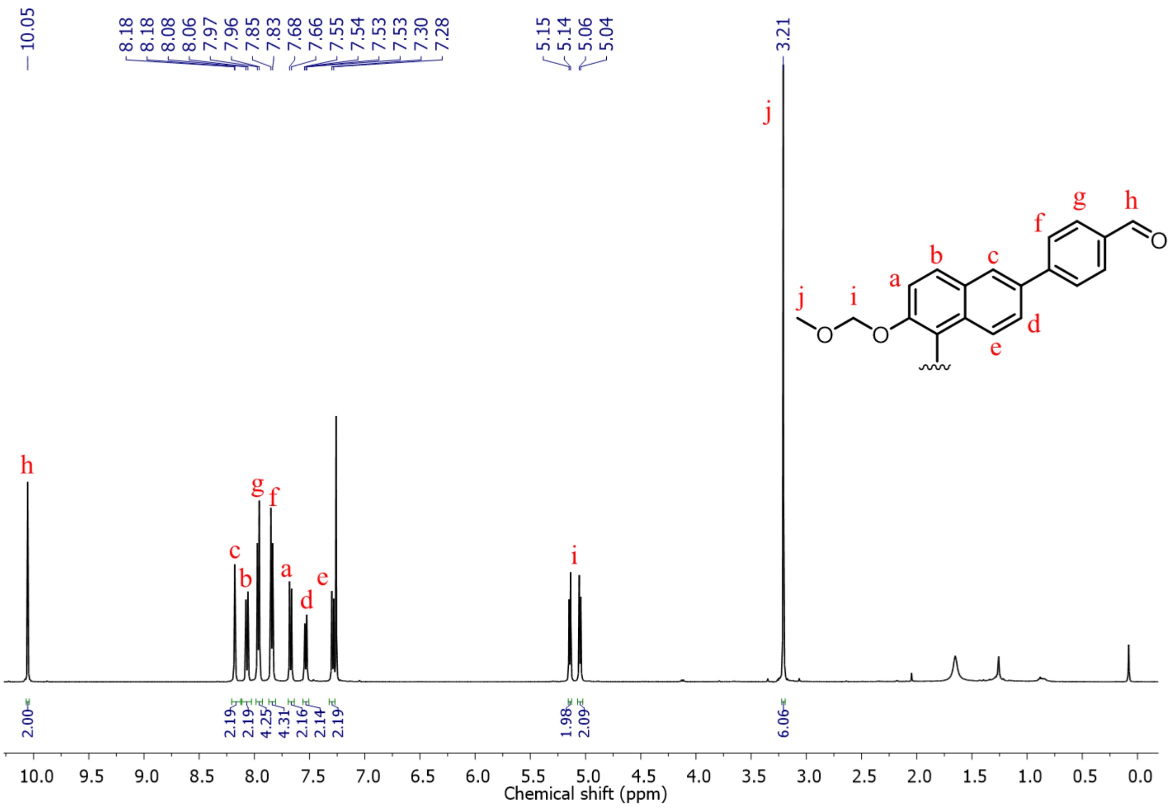

Figure S 2: ^{1}H NMR (500 MHz, $CDCl_3$, 298 K) spectrum of compound **1**.

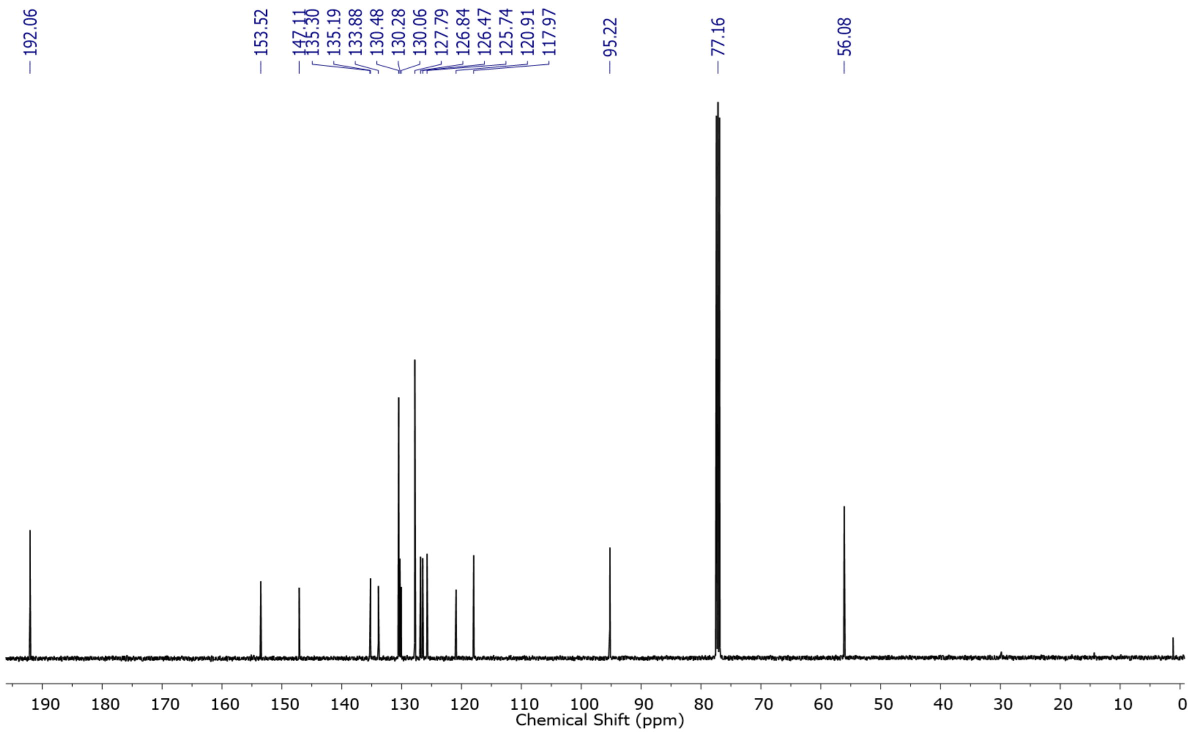

Figure S 3: ^{13}C NMR (126 MHz, $CDCl_3$, 298 K) spectrum of compound **1**.

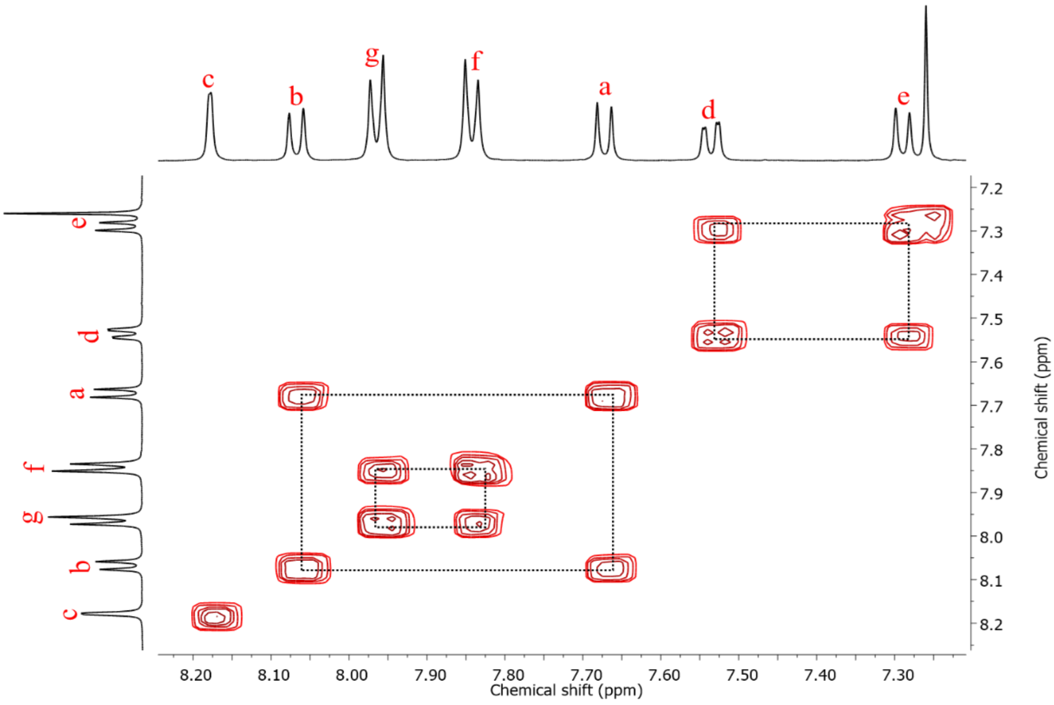

Figure S 4: ^{1}H-^{1}H COSY NMR (500 MHz, $CDCl_3$, 298 K) spectrum of compound **1**.

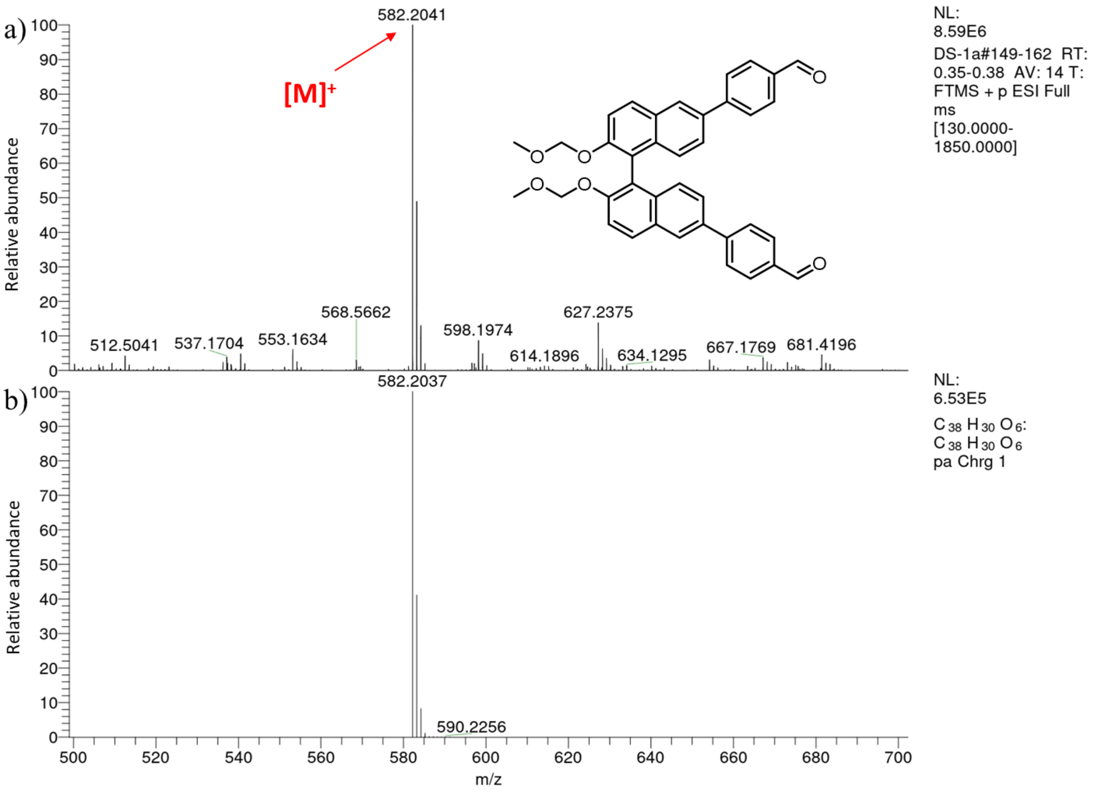

Figure S 5: High-Resolution Mass Spectrum (HRMS) of compound **1**. (a) experimental and (b) theoretical of compound **1**.

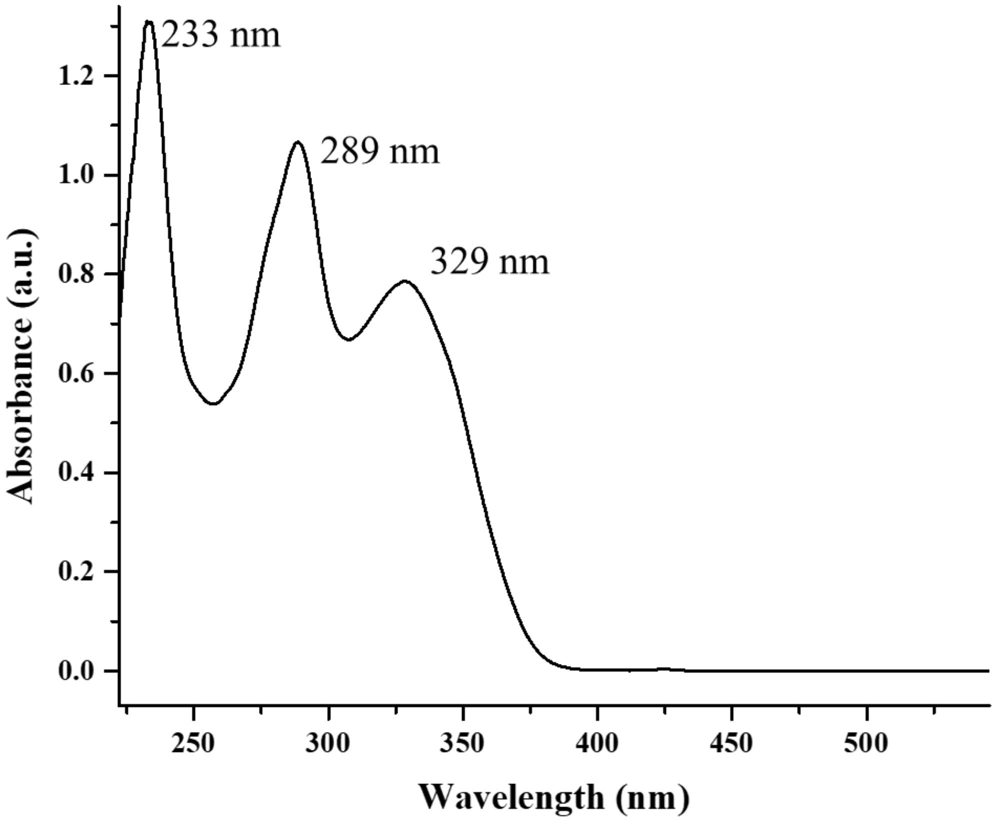

Figure S 6: UV spectrum of (*R*)-**1** (THF, 1 × 10^{-4} M, 298 K).

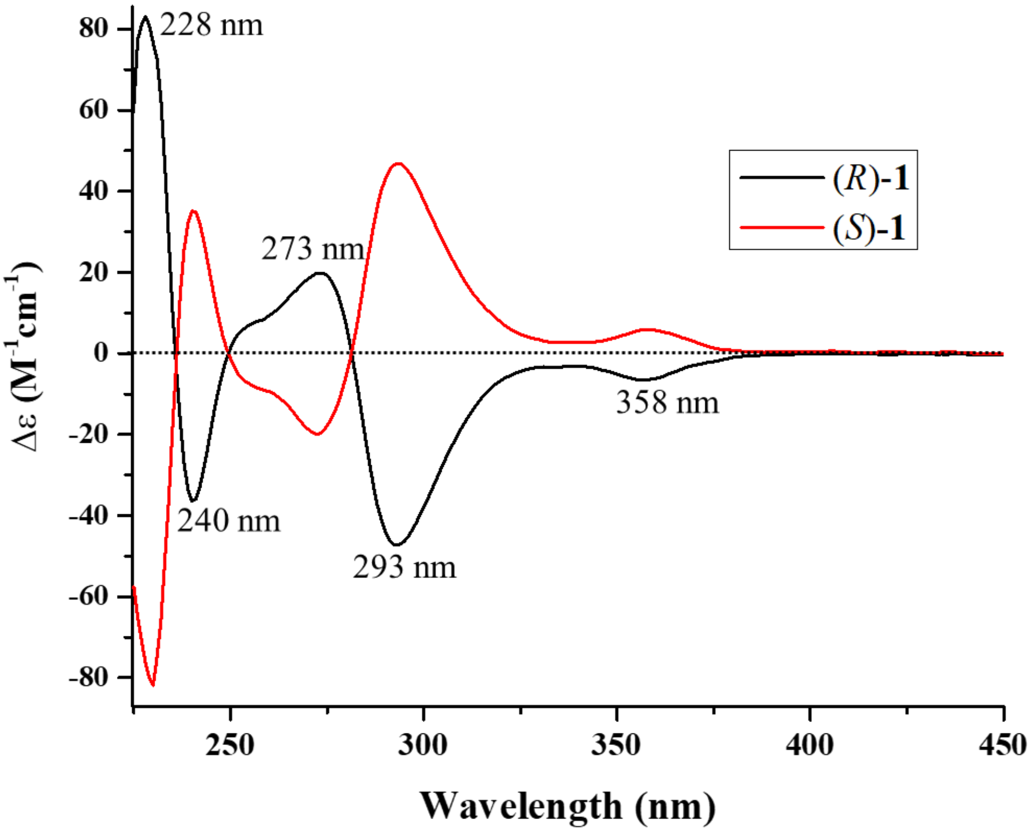

Figure S 7: CD spectra of (*R*)-**1**(black) and (*S*)-**1** (red) (THF, 5 × 10^{-5} M, 298 K).

3.2 Synthesis of 2:

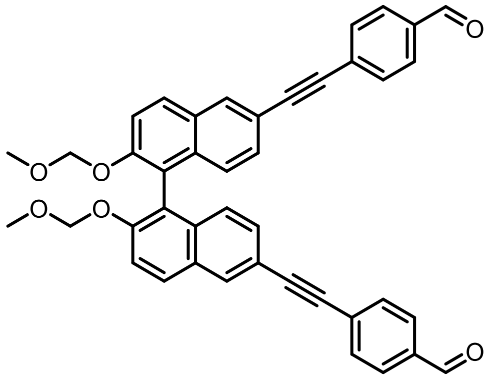

To a 25 mL Schlenk tube, compounds **20** (500 mg, 939 µmol), **22**[4] (306 mg, 2.35 mmol), dry THF (3 mL), and dry Et_3N (1 mL) were added successively under an argon atmosphere. The reaction mixture was then purged with an argon balloon for 30 minutes and heated at 75 ˚C. $Pd(PPh_3)_4$ (100 mg, 86.5 µmol) was added to the reaction mixture and the stirring was continued for 24 hours at the same temperature. After completion of the reaction (monitored by TLC), the reaction was quenched by saturated brine (5 mL) and extracted with ethyl acetate twice (2 × 10 mL). The combined organic phase was washed with brine and dried over anhydrous sodium sulphate. The concentrated residue was purified by column chromatography over silica gel using 20% ethyl acetate (R_f = 0.5 in 35:65 ethylacetate: hexane solvent) in hexane to afford the desired products as a yellow crystalline solid.

Yield: 180 mg (285 µmol, 30%).

^{1}H NMR (500 MHz, $CDCl_3$) δ 10.02 (s, 2 H), 8.14 (s, 2 H), 7.97(d, ^3J = 9 Hz, 2 H), 7.87 (d, ^3J = 8.2 Hz, 4 H), 7.69 (d, ^3J = 8.4 Hz, 4 H), 7.63 (d, ^3J = 9.4 Hz, 2 H), 7.34 (d, ^3J = 9.9 Hz, 2 H), 7.14 (d, ^3J = 9.7 Hz, 2 H), 5.14 (d, ^2J = 6.9 Hz, 2 H), 5.02 (d, ^2J = 7 Hz, 2 H), 3.18 (s, 6 H) ppm.

^{13}C NMR (126 MHz, $CDCl_3$) δ 191.6, 153.8, 135.4, 133.8, 132.3, 132.2 (2), 129.8, 129.7, 129.4, 128.9, 125.8, 120.7, 118.1, 117.7, 95.0, 94.2, 88.8, 55.1 ppm.

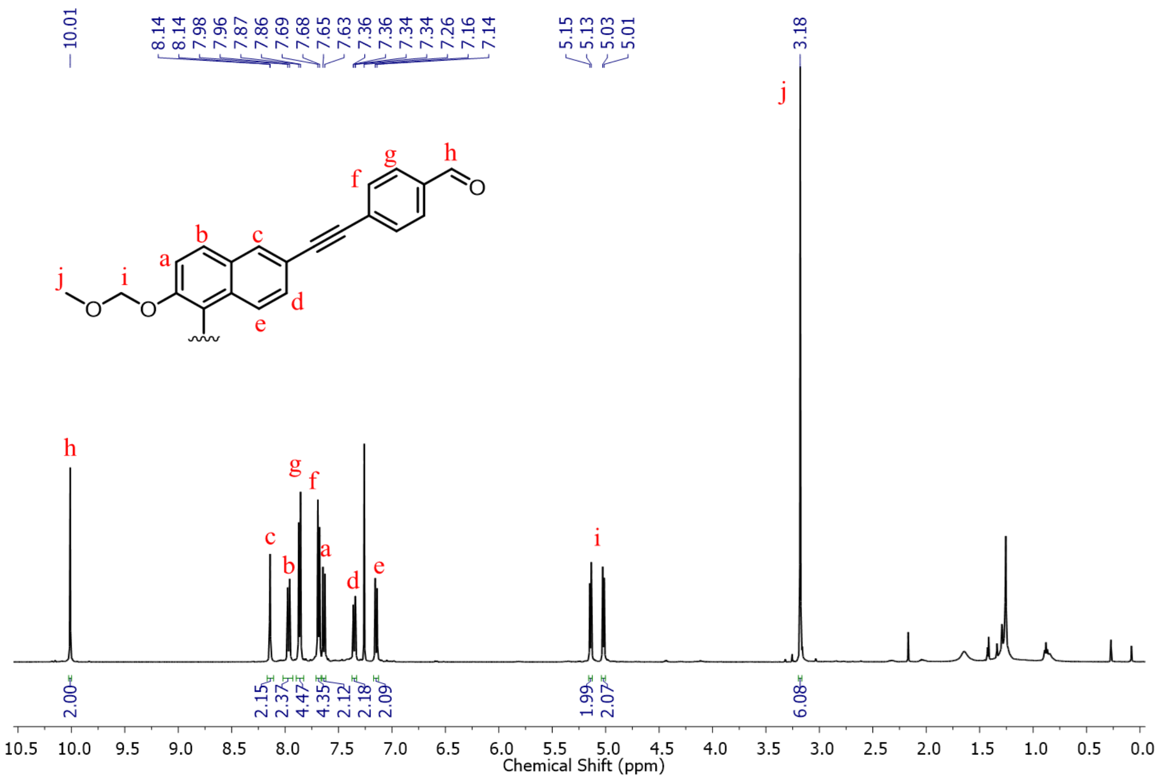

Figure S 8: ^{1}H NMR (500 MHz, $CDCl_3$, 298 K) spectrum of compound **2**.

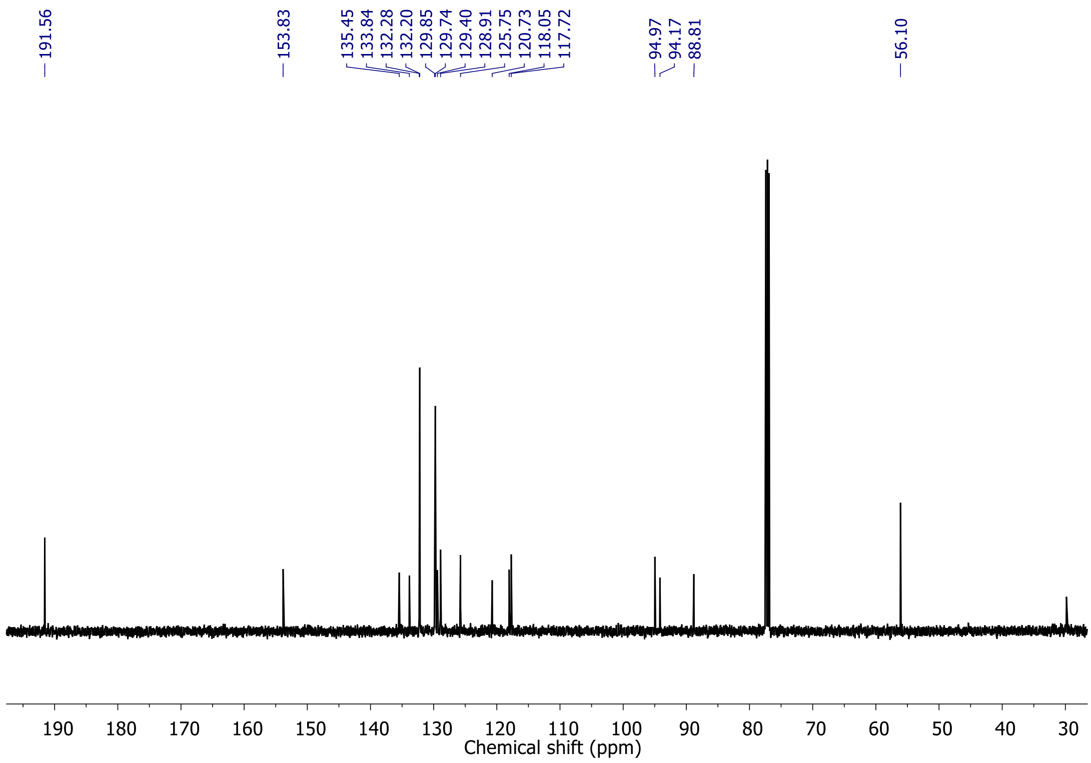

Figure S 9: ^{13}C NMR (126 MHz, $CDCl_3$, 298 K) spectrum of compound **2**.

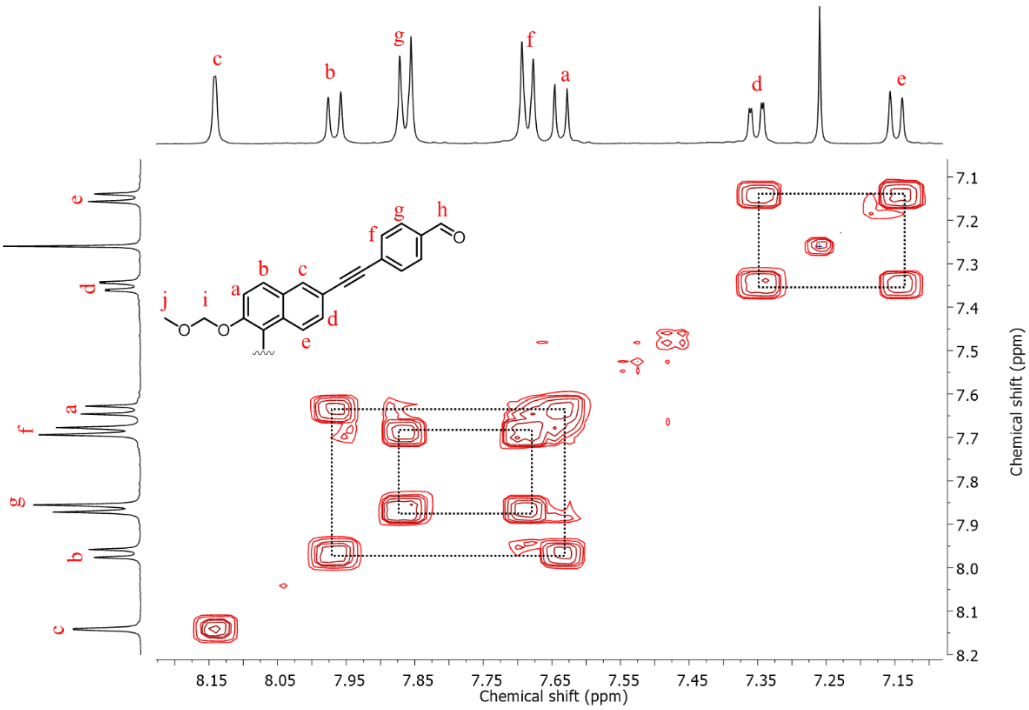

Figure S 10: Partial ^{1}H-^{1}H COSY NMR (500 MHz, $CDCl_3$, 298 K) spectrum of compound **2**.

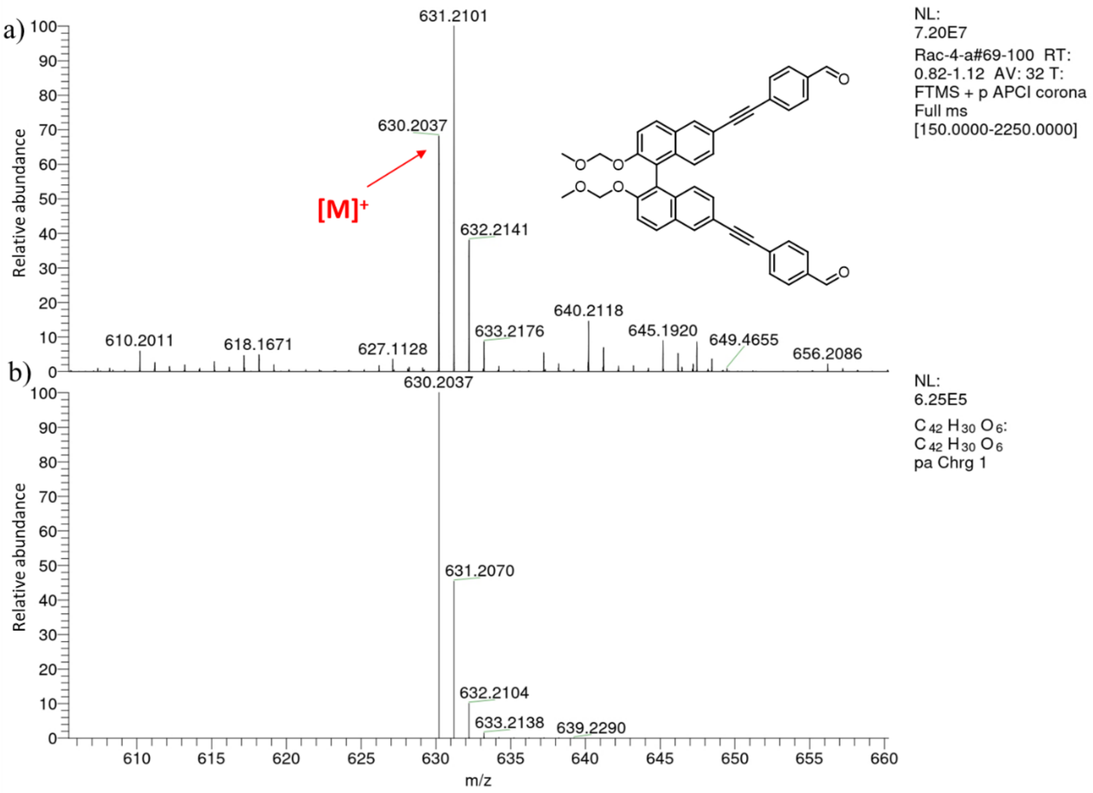

Figure S 11: High-Resolution Mass Spectrum (HRMS) (a) experimental and (b) theoretical of compound **2**.

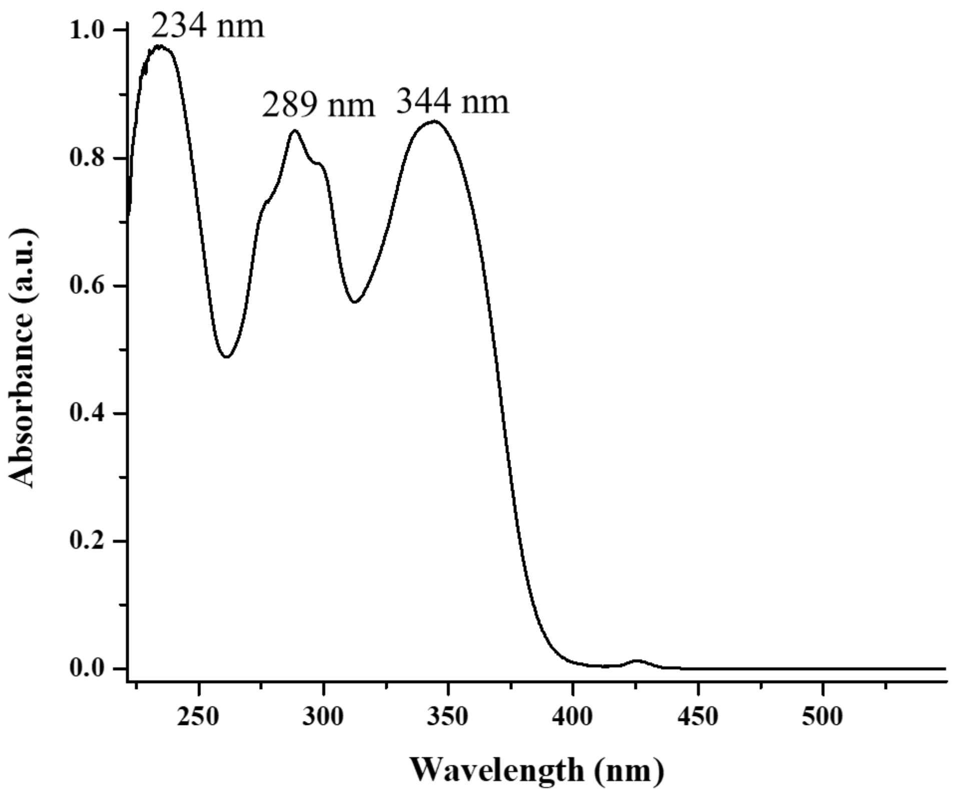

Figure S 12: UV spectrum of (*R*)-**2** (THF, 1 × 10^{-4} M, 298 K).

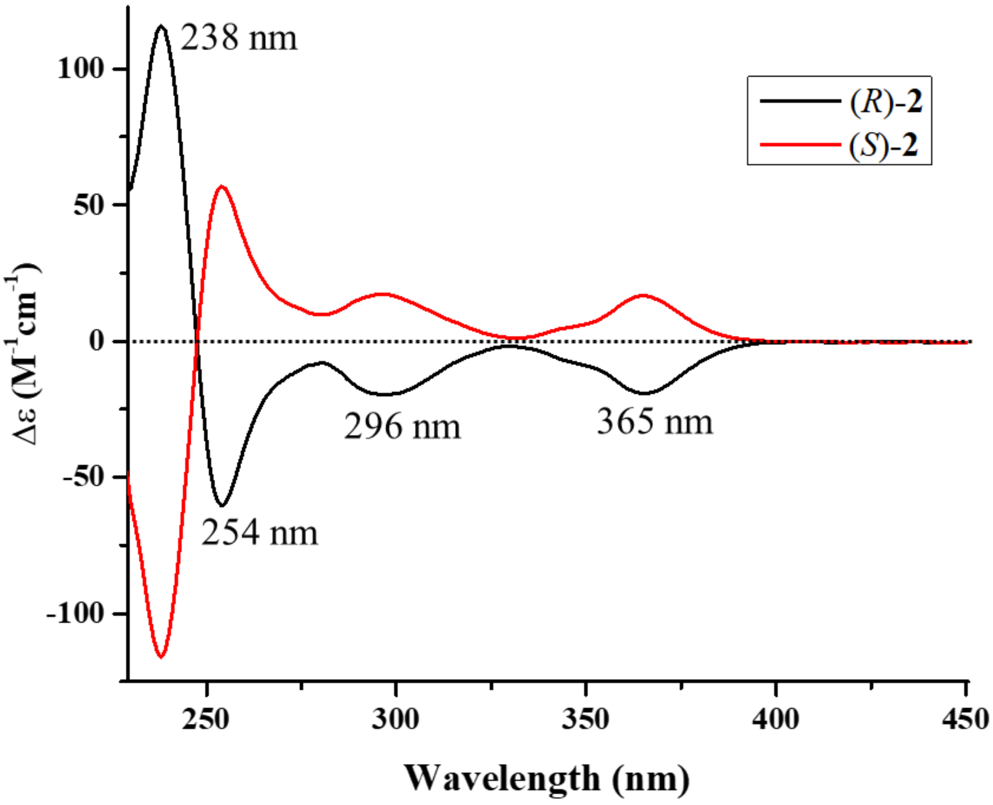

Figure S 13: CD spectra of (*R*)-**2** (black) and (*S*)-**2** (red) (THF, 5 × 10^{-5} M, 298 K).

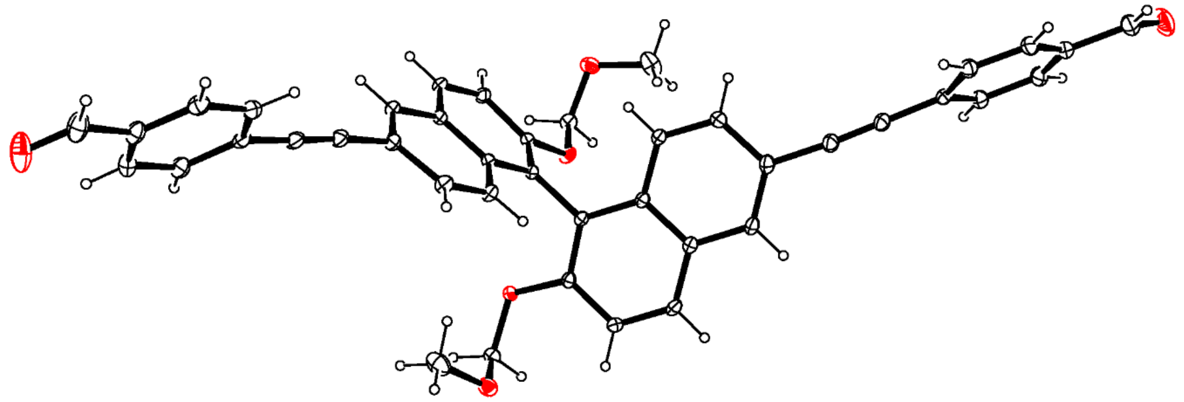

Figure S 14: Crystal structure of (±)-**2** (single crystal obtained by slow evaporation of solvent from a concentrated solution of **2** in ethyl acetate). The CCDC number is 2351058.

3.3 Synthesis of Aldehyde 4:

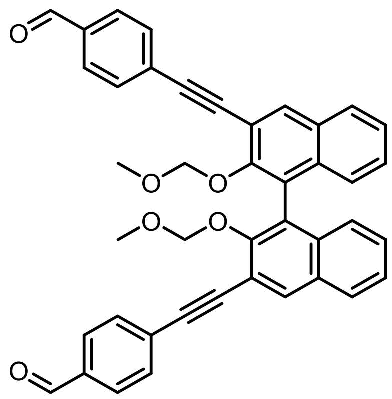

To a 50 mL Schlenk tube, compounds **23**[5] (0.25 g, 0.4 mmol), **22** (0. g, 1 mmol), dry THF (6 mL), and dry Et_3N (2 mL) were added successively under an argon atmosphere. The reaction mixture was then purged with an argon balloon for 30 minutes and heated at 75 ˚C. $Pd(PPh_3)_4$ (0.064 g, 0.12 mmol) and CuI were added to the reaction mixture and the stirring was continued for 24 hours at the same temperature. After completion of the reaction (monitored by TLC), the reaction was quenched by saturated brine (5 mL) and extracted with ethyl acetate twice (2 × 25 mL). The combined organic phases were washed with brine and dried over anhydrous sodium sulphate. The concentrated residue was purified by column chromatography over silica gel using 20% ethyl acetate (R_f = 0.5 in 25% ethylacetate in hexane) in hexane to afford the desired products as yellow colour crystalline solid.

Yield: 163 mg (0.26 mmol, 65%).

^{1}H NMR (500 MHz, $CDCl_3$) δ 10.02 (s, 2 H), 8.28 (s, 2 H), 7.89 (d, ^{3}J = 7 Hz, 2 H), 7.88 (d, ^{3}J = 8 Hz, 4 H), 7.71 (d, ^{3}J = 8 Hz, 4 H), 7.47 (dd, ^{3}J = 8 Hz, ^{3}J = 8 Hz, 2 H), 7.34 (dd, ^{3}J = 8 Hz, ^{3}J = 8 Hz, 2 H), 7.26 (d, ^{3}J = 7 Hz, 2 H), 5.17 (d, ^{2}J = 10 Hz, 2 H), 4.96 (d, ^{2}J = 10 Hz, 2 H), 2.54 (s, 6 H) ppm.

^{13}C NMR (126 MHz, $CDCl_3$) δ 191.5, 153.1, 135.7, 134.9, 134.2, 132.2, 130.4, 129.8, 129.5, 127.9 (2), 126.7, 126.0 (2), 116.7, 99.2, 92.9, 90.6, 56.3 ppm.

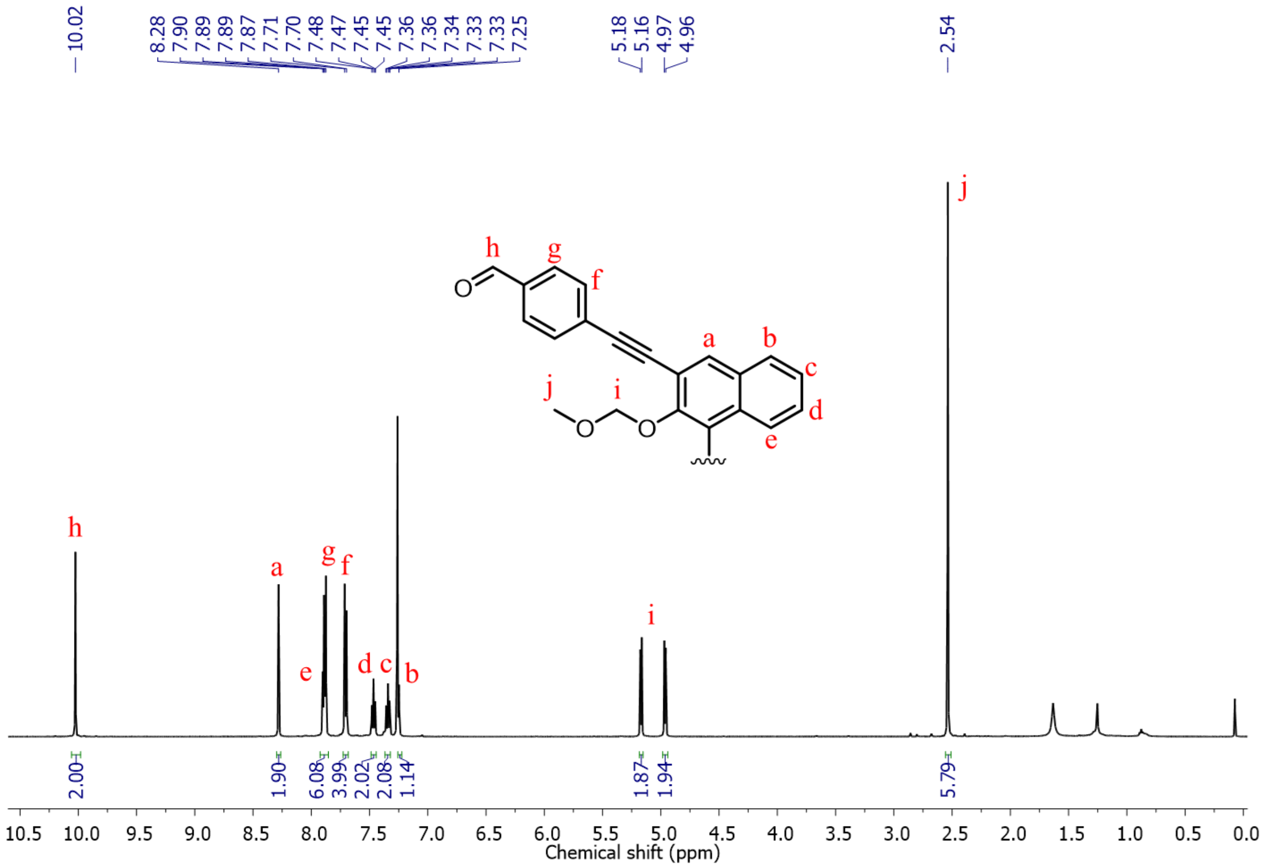

Figure S 15: ^{1}H NMR (500 MHz, $CDCl_3$, 298 K) spectrum of compound **4**.

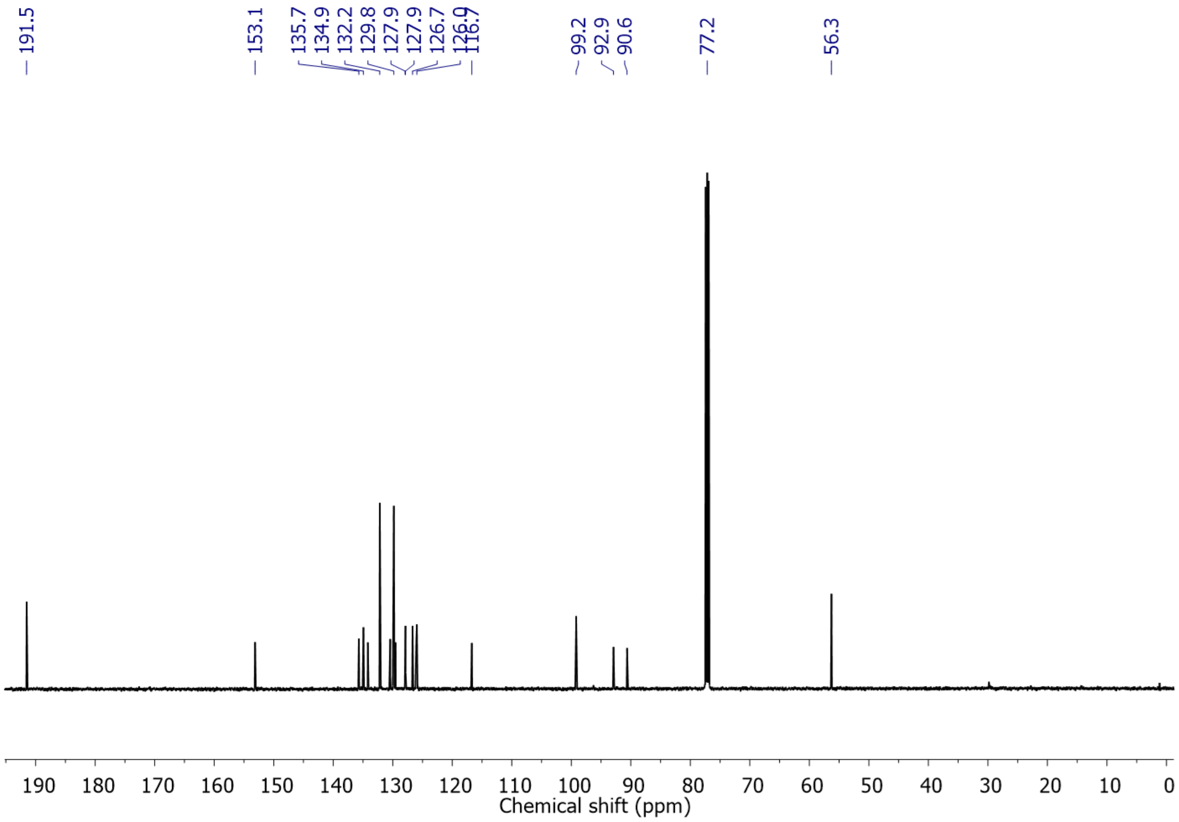

Figure S 16: ^{13}C NMR (126 MHz, $CDCl_3$, 298 K) spectrum of compound **4**.

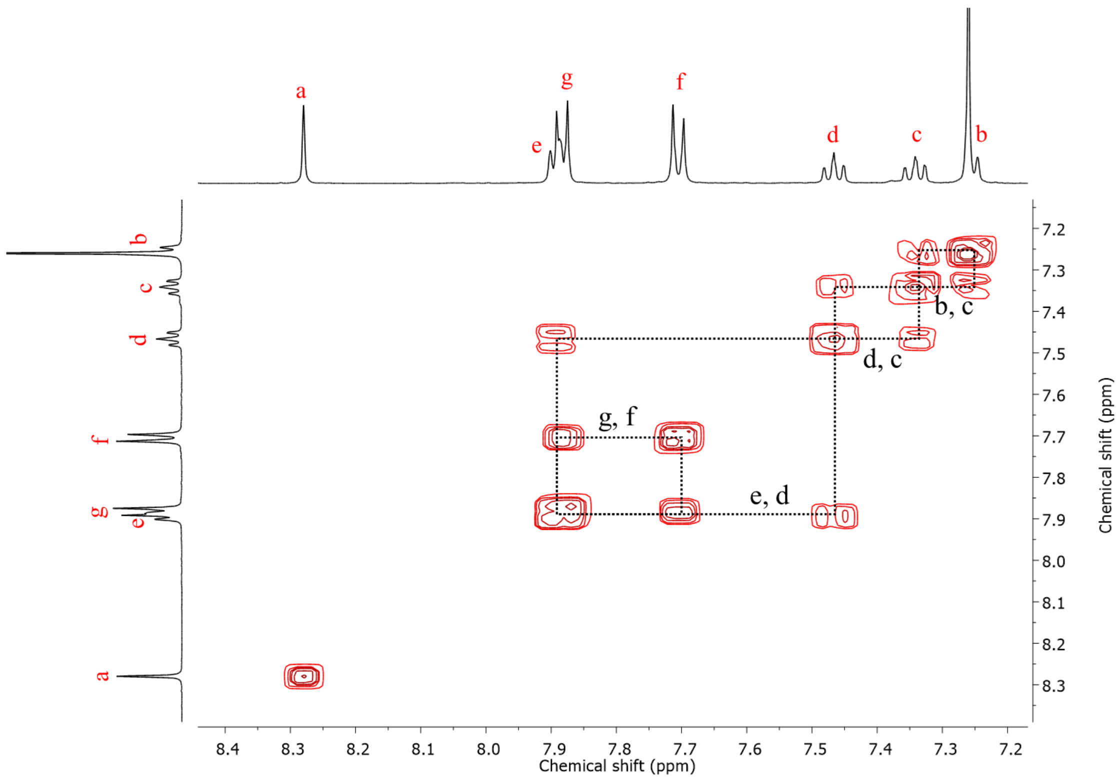

Figure S 17: Partial ^{1}H-^{1}H COSY NMR (500 MHz, $CDCl_3$, 298 K) spectrum of compound **4**.

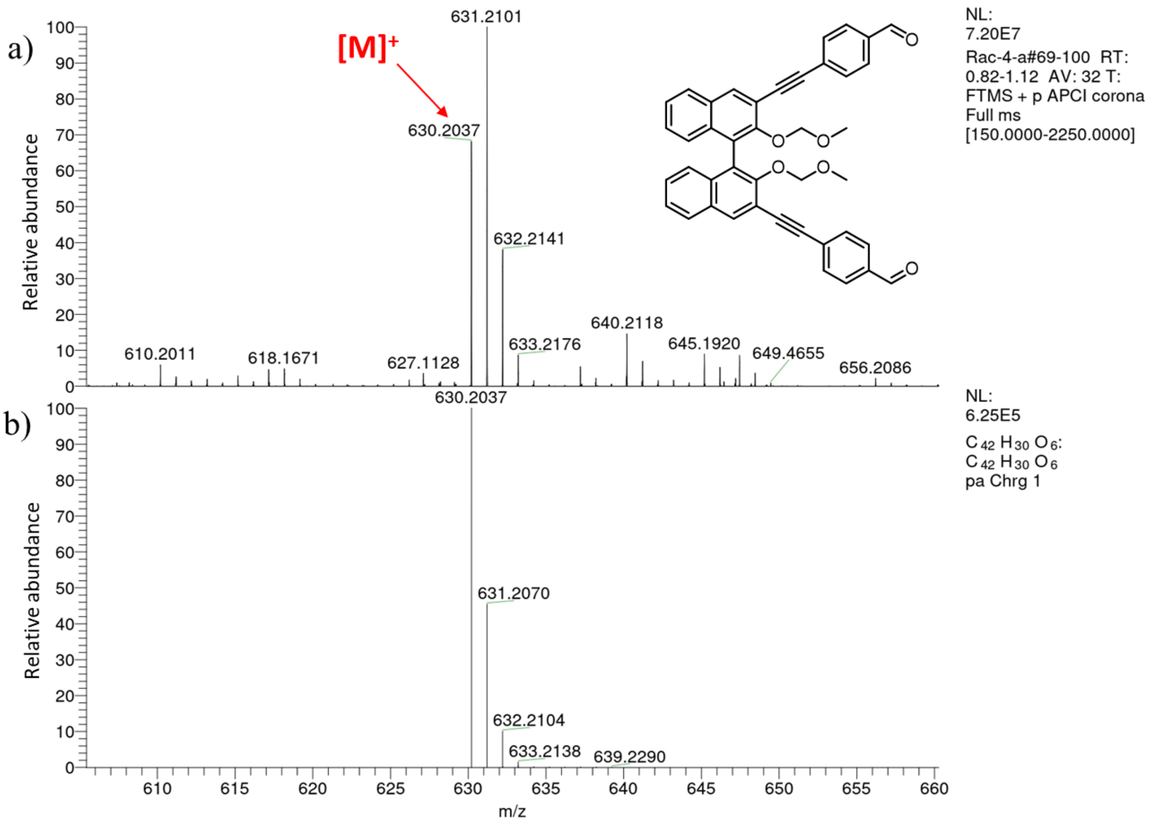

Figure S 18: High-Resolution Mass Spectrum (HRMS) (a) experimental and (b) theoretical of compound **4**.

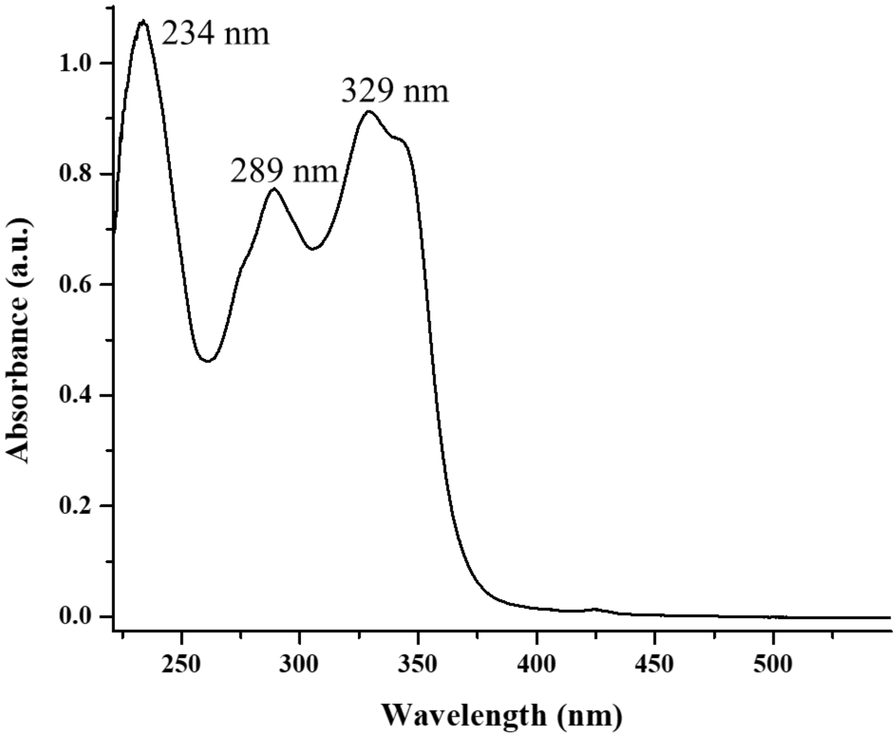

Figure S 19: UV spectrum of (*R*)-**4** (THF, 1 × 10^{-4} M, 298 K).

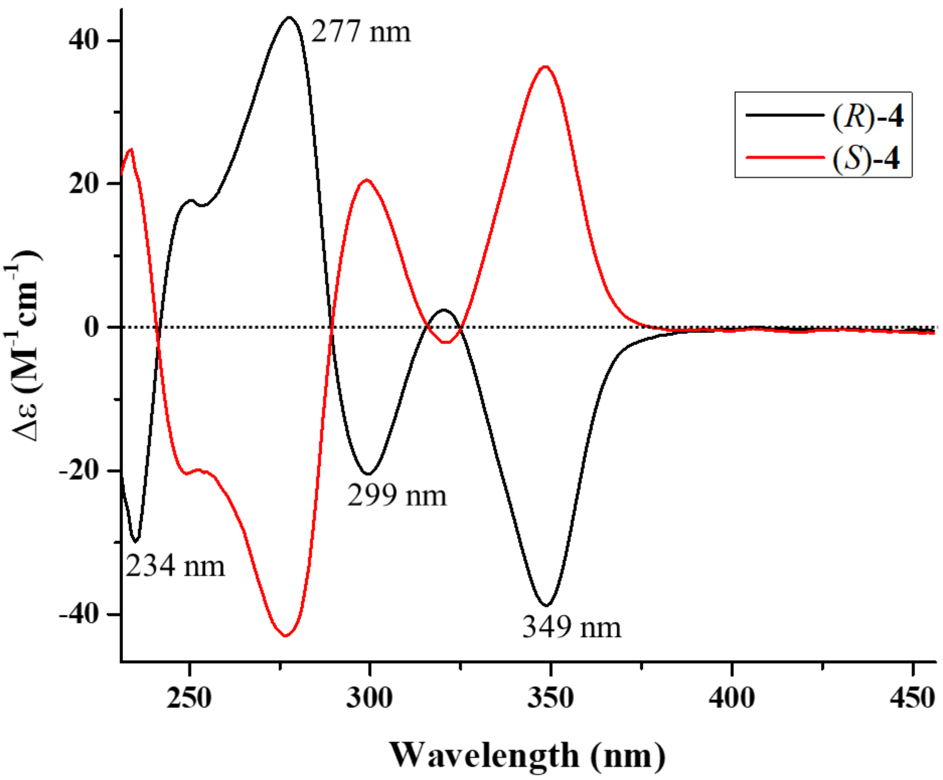

Figure S 20: CD spectrum of (*R*)-**4** (black) and (*S*)-**4** (red) (THF, 5 × 10^{-5} M, 298 K).

3.4 Synthesis of macrocycles from aliphatic bisamine

3.4.1 General procedure for the synthesis of the macrocycle

Macrocycles were prepared by mixing aldehydes **1** (one equivalent) and amine **5/6** (one equivalent) in an NMR tube (0.5 mL $CDCl_3$) and kept in heating at 50 °C for 2 h. NMR was recorded without any purification.

3.4.2 Macrocycle 8

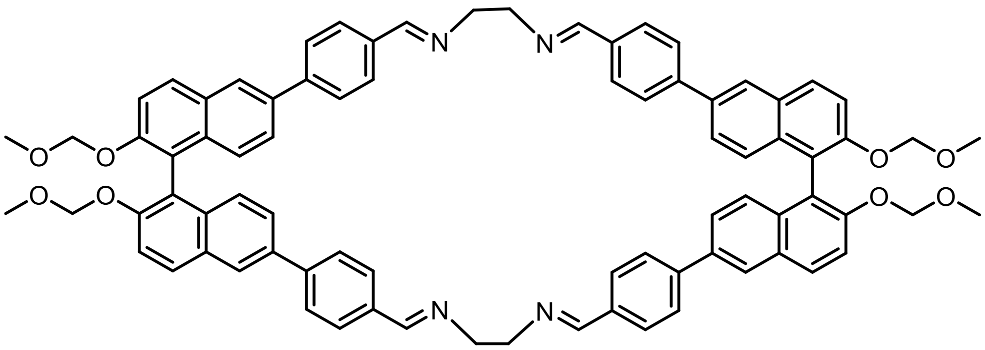

^{1}H NMR (500 MHz, $CDCl_3$, 298 K) δ 8.24 (s, 4 H), 8.09 (s, 4 H), 8.00 (d, ^3J = 9 Hz, 4 H), 7.71 (d, ^3J = 8 Hz, 8 H), 7.65 (d, ^3J = 8 Hz, 8 H) 7.60 (d, ^3J = 9 Hz, 4 H), 7.43 (d, ^3J = 8 Hz, 4

H), 7.16 (d, ^3J = 9 Hz, 4 H), 5.11 (d, ^2J = 7 Hz, 4 H), 5.01 (d, ^2J = 7 Hz, 4 H), 4.10 (d, ^2J = 9 Hz, 4 H), 4.01 (d, ^2J = 9 Hz, 4 H), 3.18 (s, 12 H) ppm.

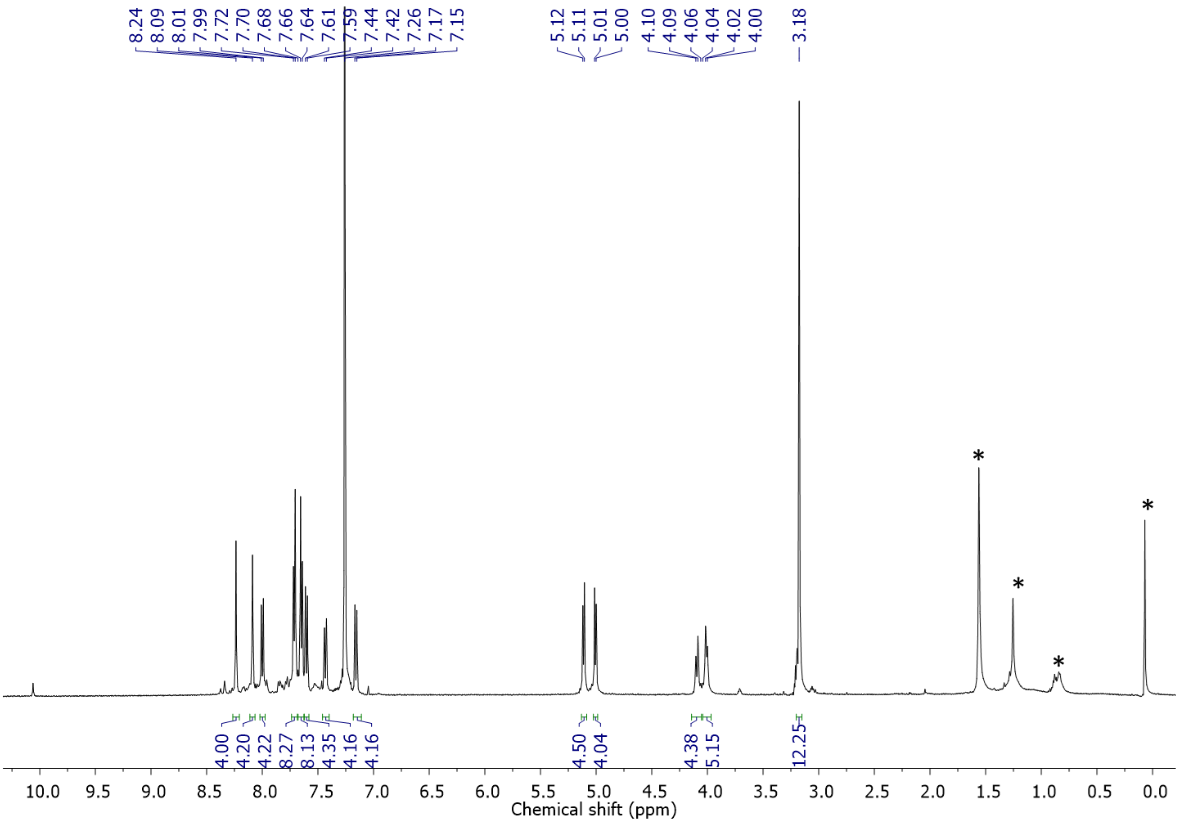

Figure S 21: ^{1}H NMR (500 MHz, $CDCl_3$, 298 K) spectrum of macrocycle **8**. The asterisks represent solvent impurities- water, and hexane.

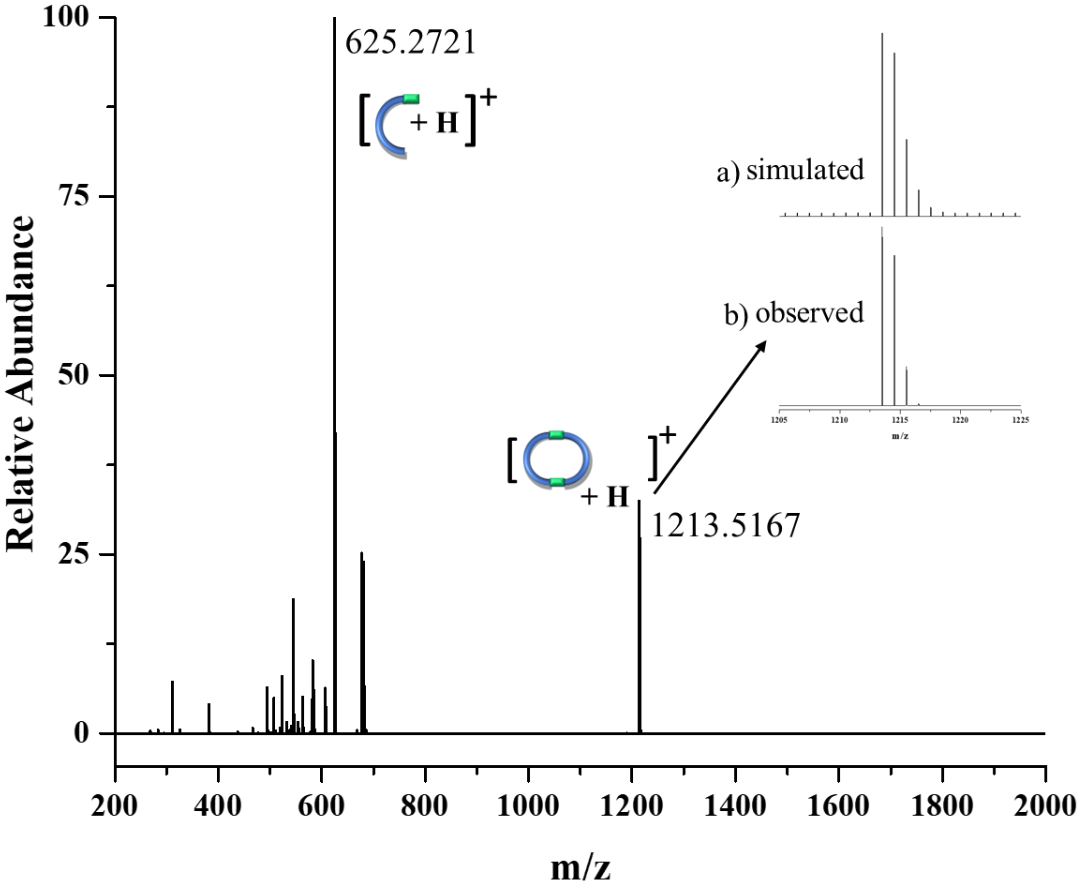

Figure S 22: ESI-TOF spectrum of macrocycle **8**. The macrocycle is dissociating in the mass condition, leading to the observation of fragmentation along with the molecular mass. Inset shows a) theoretical and b) experimental isotopic distribution.

3.4.3 Macrocycle 9

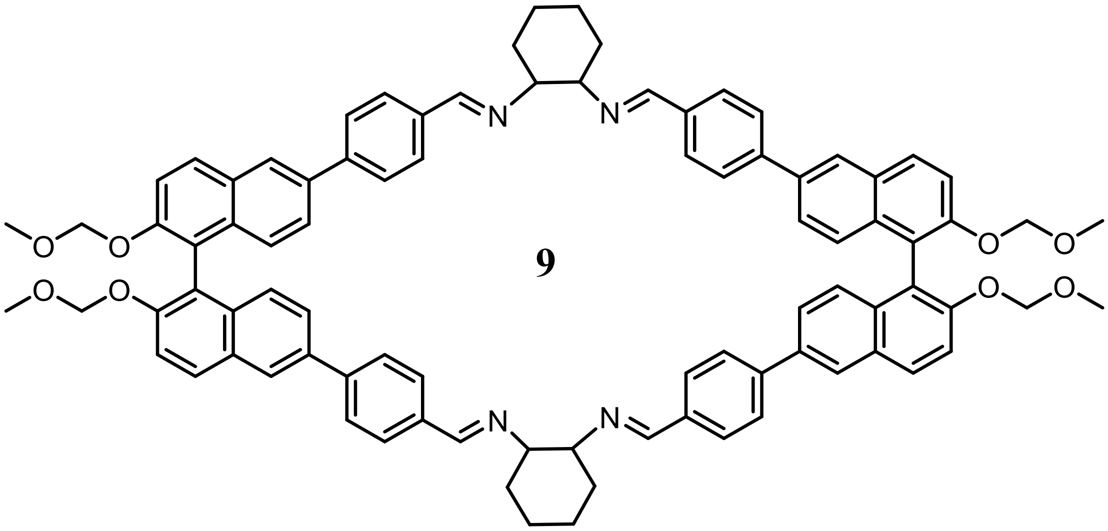

(*R*)-**1**+ (*R*,*R*)-**6**:

^{1}H NMR (500 MHz, $CDCl_3$, 298 K) δ 8.23 (s, 4 H), 8.07 (d, ^{4}J= 1.5 Hz, 4 H), 7.67 (d, ^{3}J= 8.3 Hz, 4 H), 7.62 (d, ^{3}J= 8.3 Hz, 8 H) 7.58 (d, ^{3}J= 9 Hz, 4 H) 7.43 (dd, ^{3}J= 8.8 Hz, ^{4}J= 1.5 Hz, 4 H), 7.17 (d, ^{3}J= 8.8 Hz, 4 H), 5.08 (d, ^{2}J= 5 Hz, 4 H), 4.99 (d, ^{2}J= 5 Hz, 4 H), 3.45 (m, 4 H), 3.17 (s, 12 H), 1.89 (m, 16 H).

(*R*)-**1**+ (*S*,*S*)-**6**:

^{1}H NMR (500 MHz, $CDCl_3$, 298 K) δ 8.27 (s, 4 H), 8.04 (s, 4 H), 7.98 (d, ^{3}J= 10 Hz, 4 H), 7.66 (d, ^{3}J= 10 Hz, 8 H) 7.59 (m, 12 H) 7.38 (d, ^{3}J= 10 Hz, 4 H), 7.14 (d, ^{3}J= 10 Hz, 4 H), 5.08 (d, ^{2}J= 5 Hz, 4 H), 5.01(d, ^{2}J= 5 Hz, 4 H), 3.45 (m, 4 H), 3.18 (s, 12 H), 1.86 (m, 16 H).

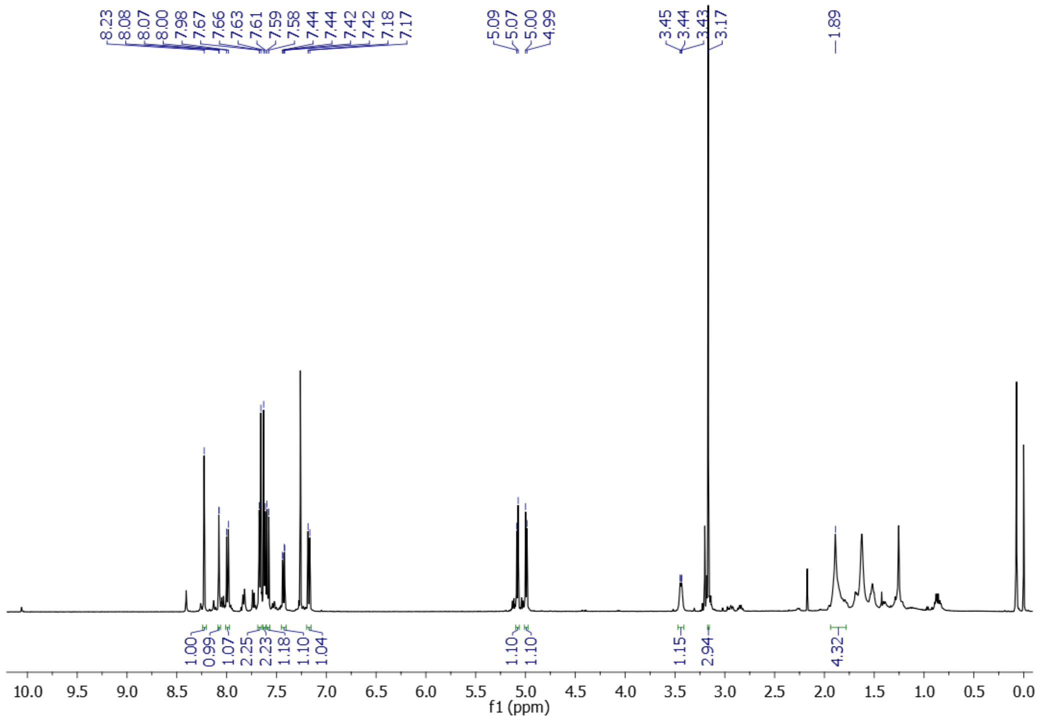

Figure S 23: ^{1}H NMR (500 MHz, $CDCl_3$, 298 K) spectrum of (*R*)-**1**+ (*R*,*R*)-**6** or (*S*)-**1**+ (*S*,*S*)-**6**.

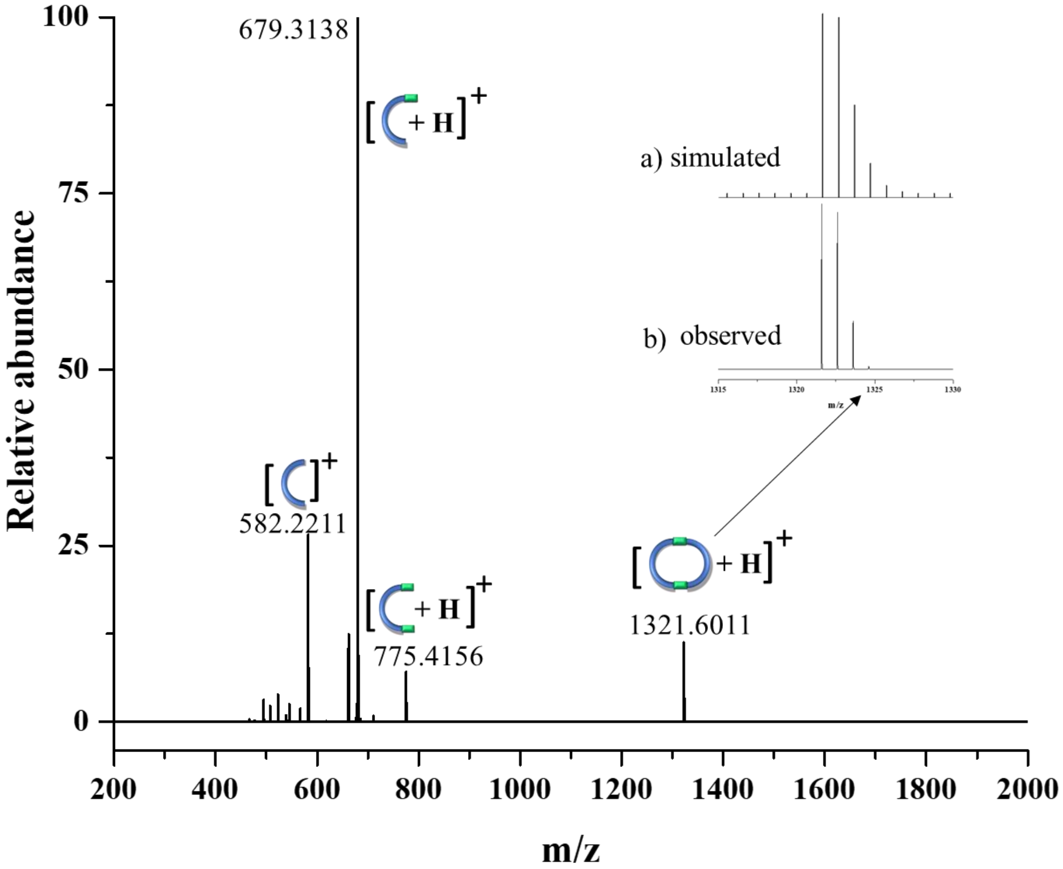

Figure S 24: ESI-TOF spectrum of macrocycle **9**. The macrocycle is dissociating in the mass condition, leading to the observation of fragmentation along with the molecular mass. Inset shows a) theoretical and b) experimental isotopic distribution.

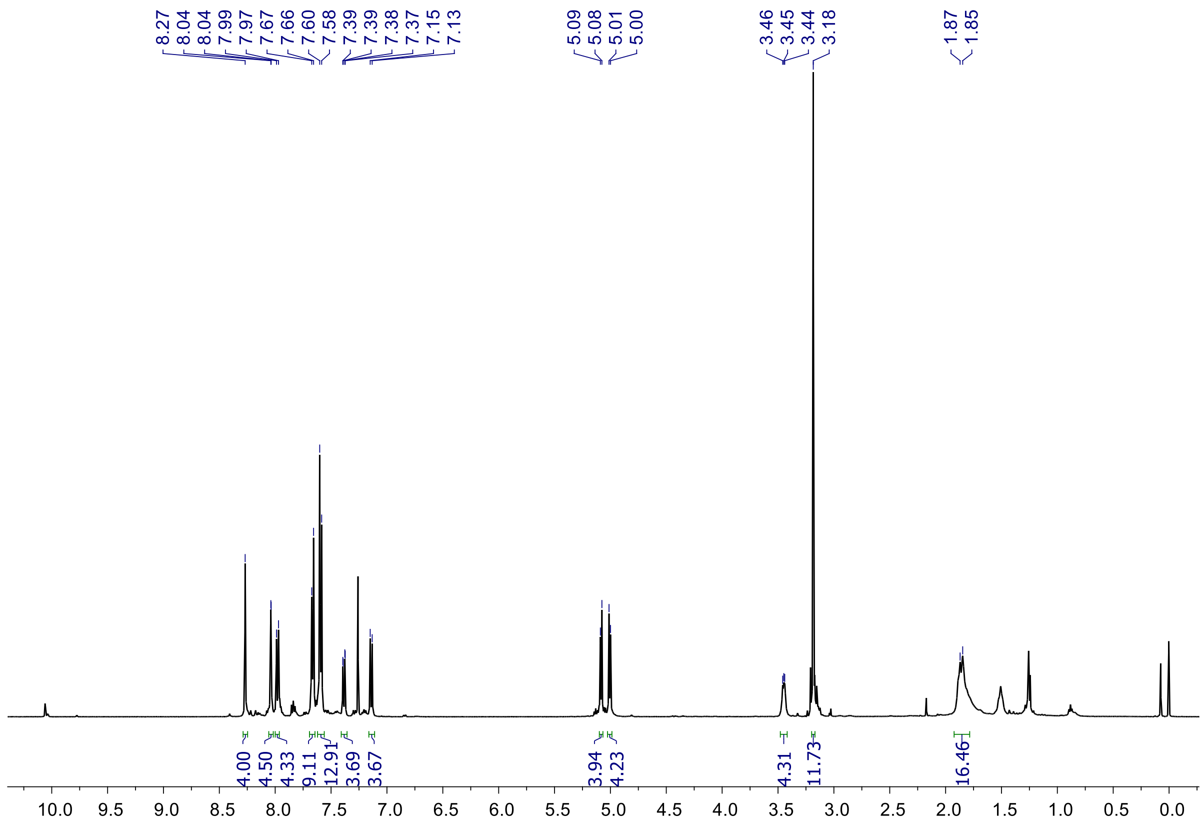

Figure S 25: ^{1}H NMR (500 MHz, $CDCl_3$, 298 K) spectrum of (*R*)-**1**+ (*S*,*S*)-**6** or (*S*)-**1**+ (*R*,*R*)-**6**.

3.5 Self-sorting during macrocycle formation from aliphatic bisamine

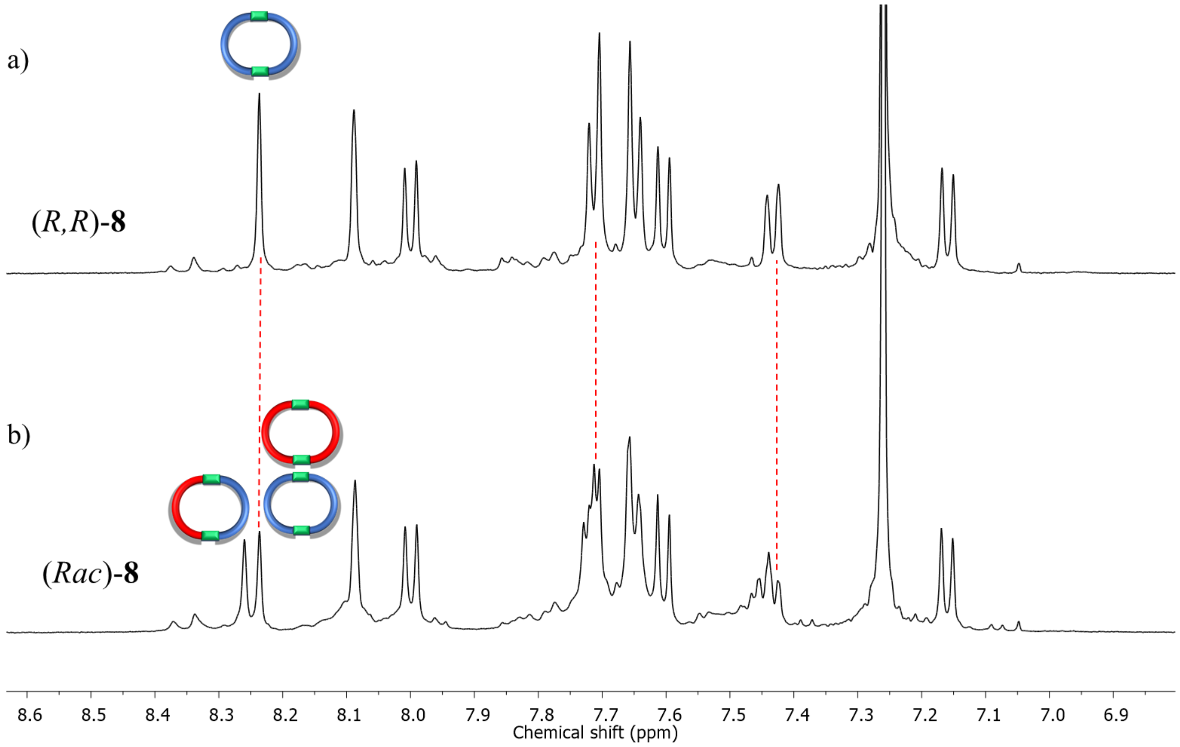

Figure S 26: Partial ^{1}H NMR (500 MHz, $CDCl_3$, 298 K) comparison of macrocycle **8** obtained from a) (*R*)-**1** and **5**; b) (±)-**1** and **5**. The new imine-derived peak at 8.26 ppm in the bottom spectrum corresponds to *R*,*S*-**8**. This experiment suggests there is no preference for homochiral or heterochiral self-sorting.

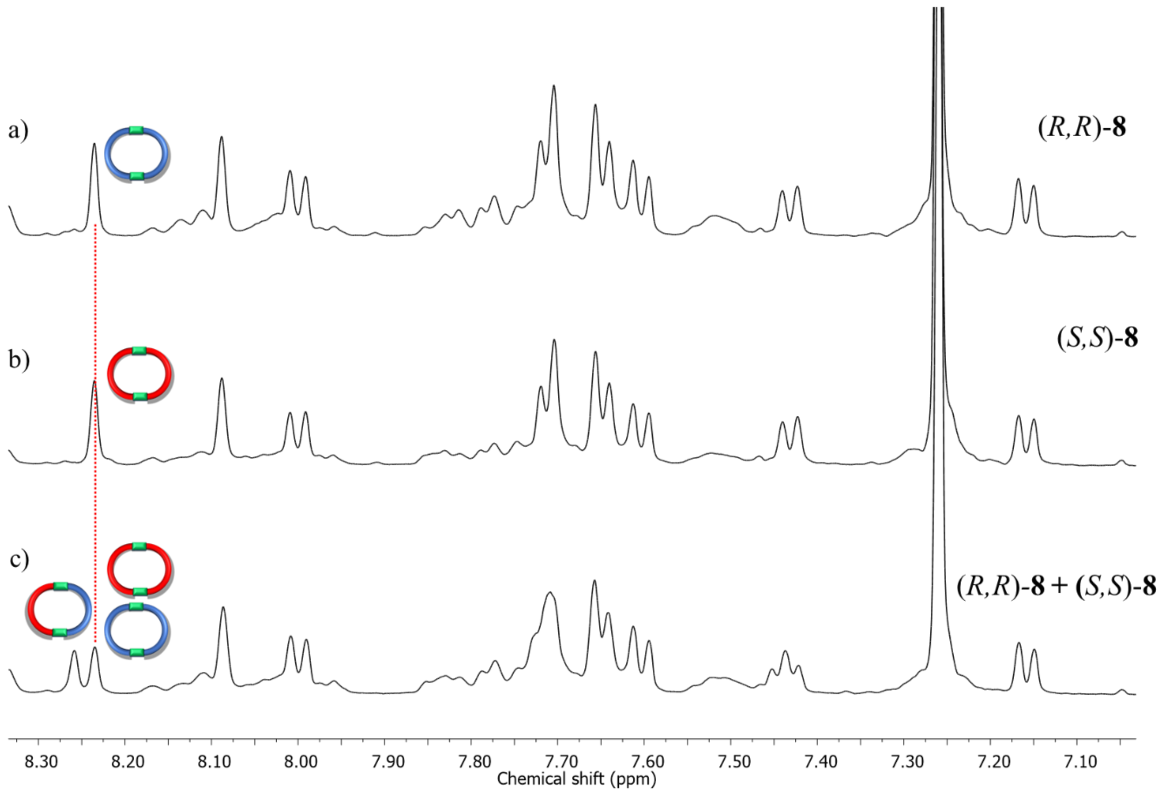

Figure S 27: Partial ^{1}H NMR (500 MHz, $CDCl_3$, 298 K) showing the comparison between the macrocycles obtained from the reaction of ethylne diamine with a) (*R*)-**1** b) (*S*)-**1** and c) upon mixing the two macrocycles and heating at 50 °C for 2 hours.

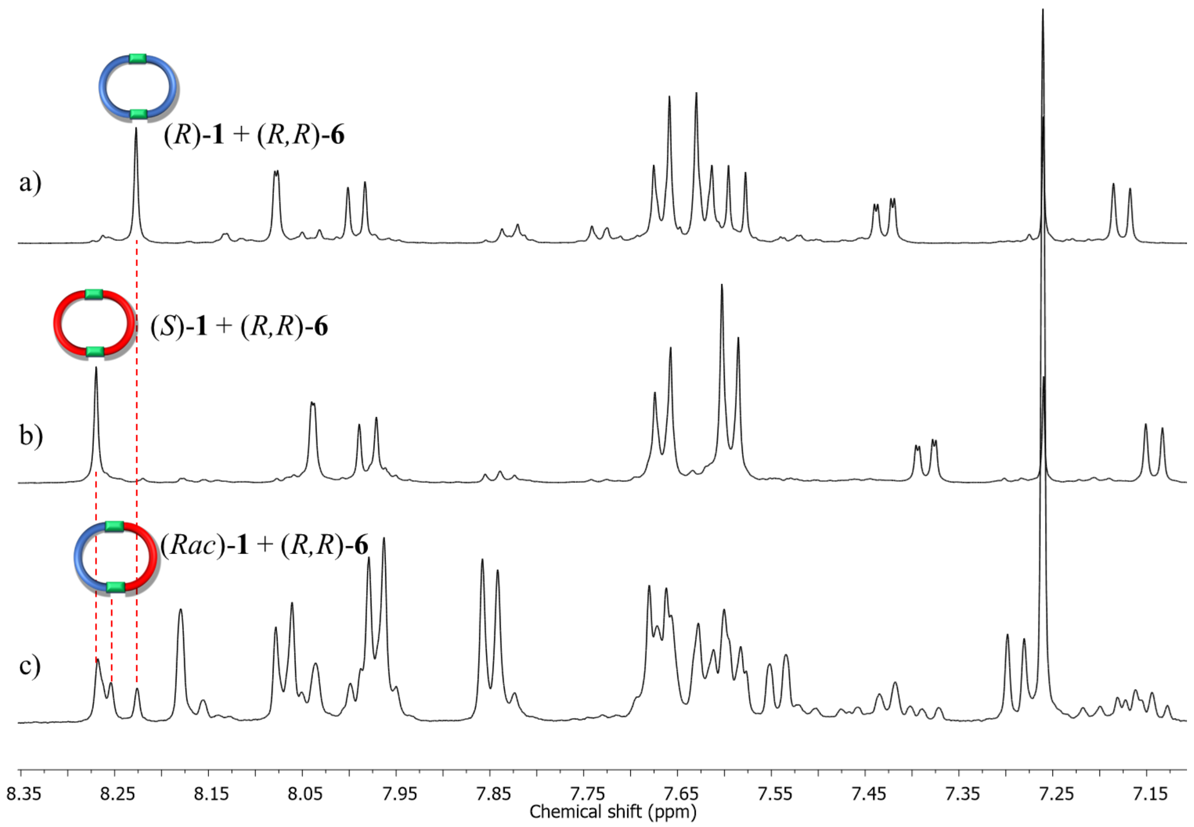

Figure S 28: Partial ^{1}H NMR (500 MHz, $CDCl_3$, 298 K) comparison of macrocycle **9** obtained from a) (*R*)-**1** and (*R*,*R*)-**6**; b) (*S*)-**1** and (*R*,*R*)-**6**; c) (±)-**1** and (*R*,*R*)-**6**. This experiment suggests little preference for homochiral over heterchiral self-sorting during macrocycle formation.

3.6 Synthesis of macrocycles from aromatic amine

3.6.1 General procedure for the synthesis of macrocycle

Aldehyde (0.02 mmol) and amine **7** (0.02 mmol) were combined in a 100 mL round-bottom flask containing 30 mL of anhydrous chloroform. 0.1 µL of acetic acid was added to this mixture and the solution was stirred under reflux conditions for 48 hours. Upon completion of the reaction, the solvent was filtered through solid sodium bicarbonate and subsequently evaporated under reduced pressure, yielding the desired compound.

3.6.2 Macrocycle 12

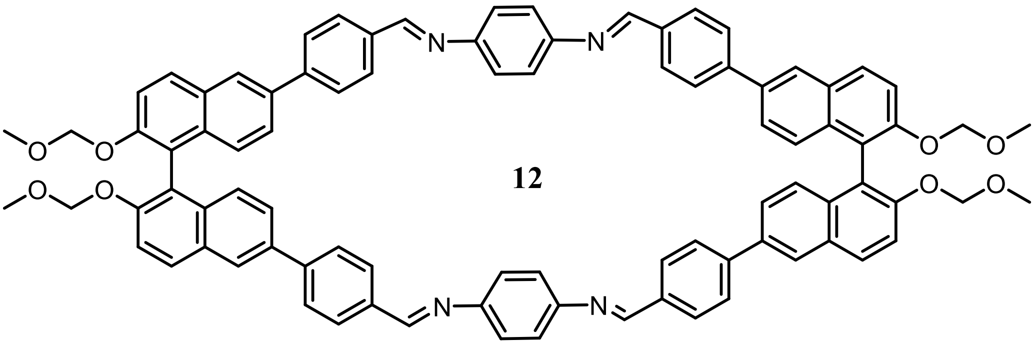

^{1}H NMR (500 MHz, $CDCl_3$) δ 8.96 (s, 4 H), 8.19 (s, 4 H), 8.07 (d, ^{3}J = 9.7 Hz, 4 H), 8.00 (d, ^{3}J = 8.1 Hz, 8 H), 7.81(d, ^{3}J = 7.5 Hz, 8 H), 7.66 (d, ^{3}J = 8.9 Hz, 4 H), 7.57 (d, ^{3}J = 9.7 Hz, 4 H), 7.33 (s, 8 H), 7.30 (d, ^{3}J = 8.6, 4 H), 5.14 (d, ^{2}J = 5.7 Hz, 4 H), 5.05 (d, ^{2}J = 5.7 Hz, 4 H), 3.21(s, 12 H) ppm.

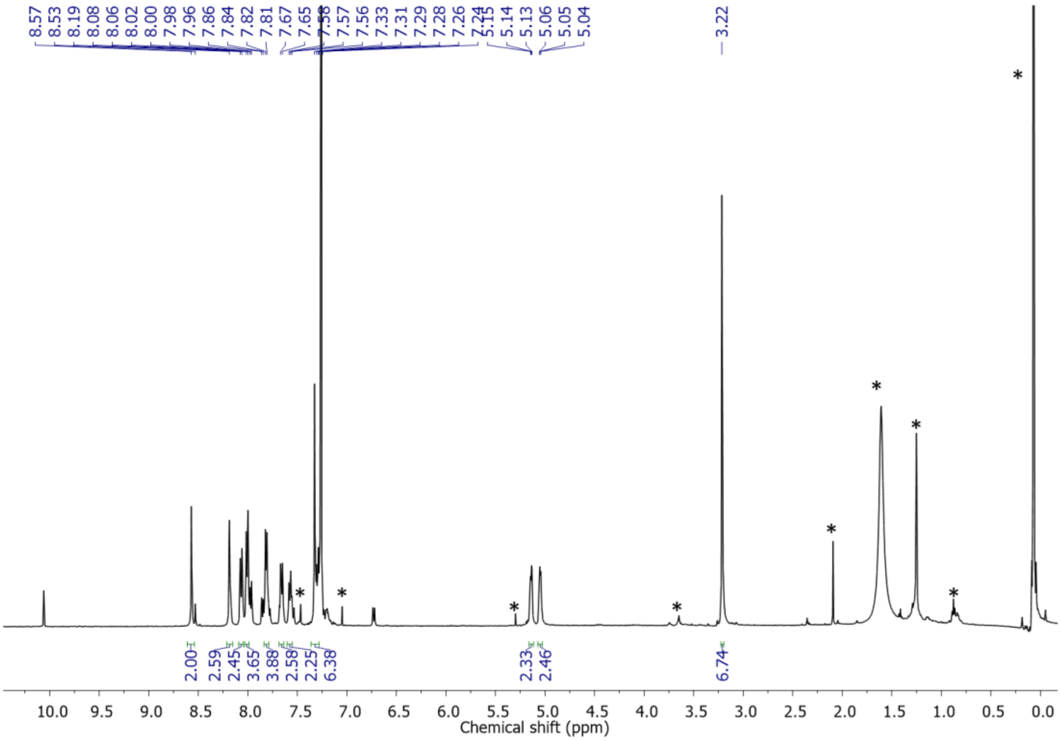

Figure S 29: ^{1}H NMR (500 MHz, $CDCl_3$, 298 K) spectrum of macrocycle **12**. The asterisks represent solvent impurities.

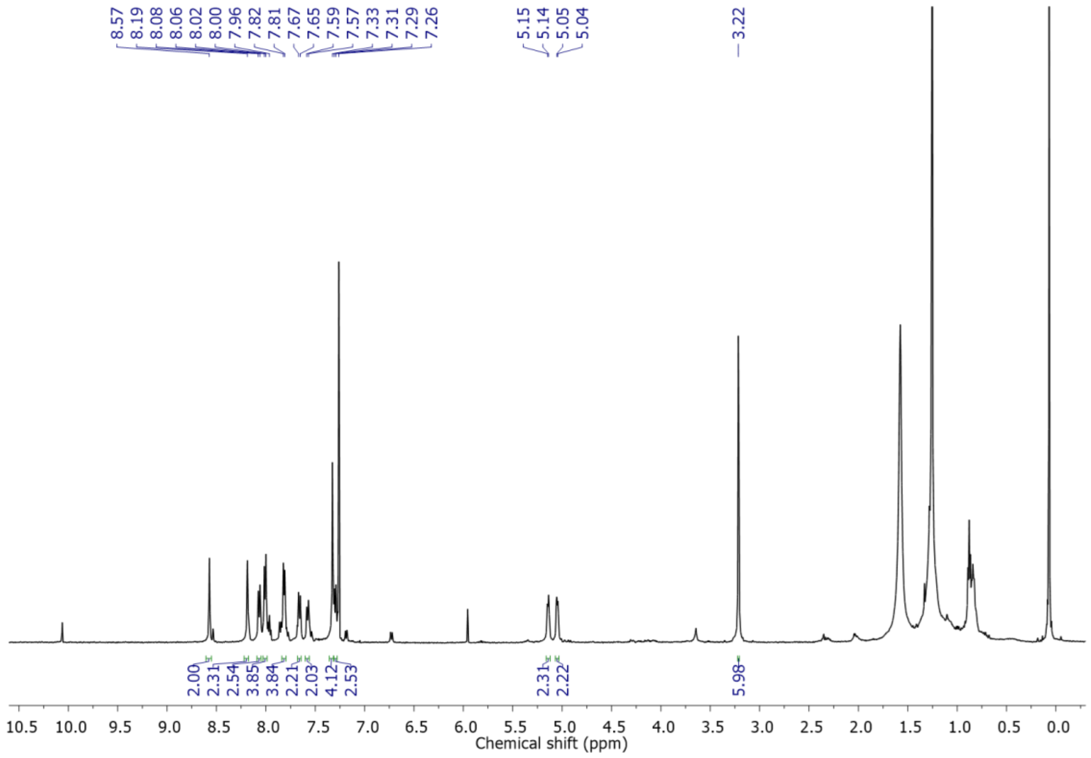

Figure S 30: ^{1}H NMR (500 MHz, $CDCl_3$, 298 K) spectrum of macrocycle **12** obtained by condensing **1** and **7** in toluene at 100 ºC for 2 days.

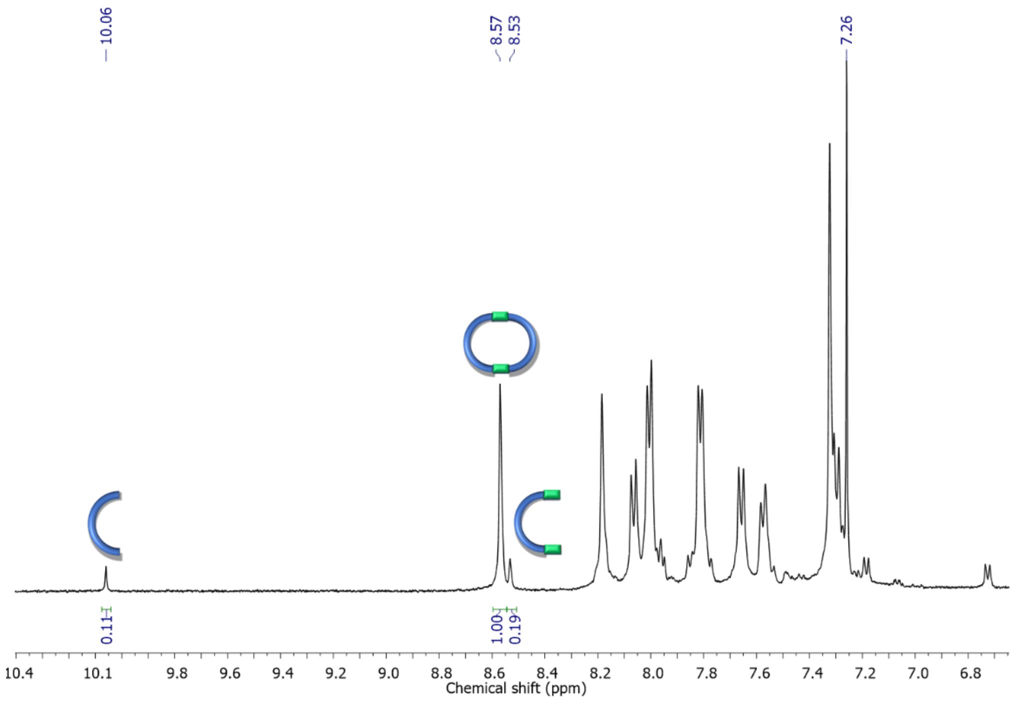

Figure S 31: Partial ^{1}H NMR (500 MHz, $CDCl_3$, 298 K) spectrum of macrocycle **12** showing the ratio between the macrocycle, unreacted aldehyde and half of the macrocycle.

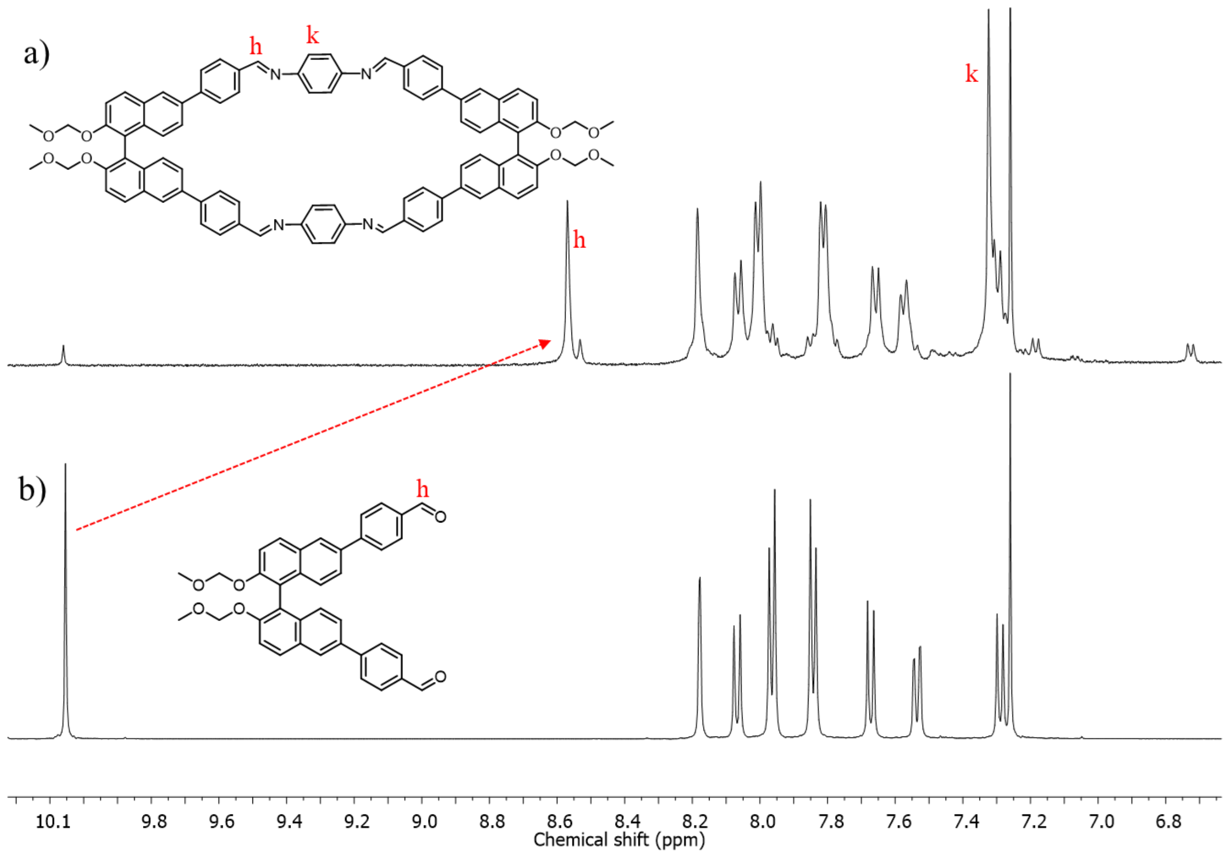

Figure S 32: Partial ^{1}H NMR (500 MHz, $CDCl_3$, 298 K) spectrum of a) macrocycle **12**; b) aldehyde **1**.

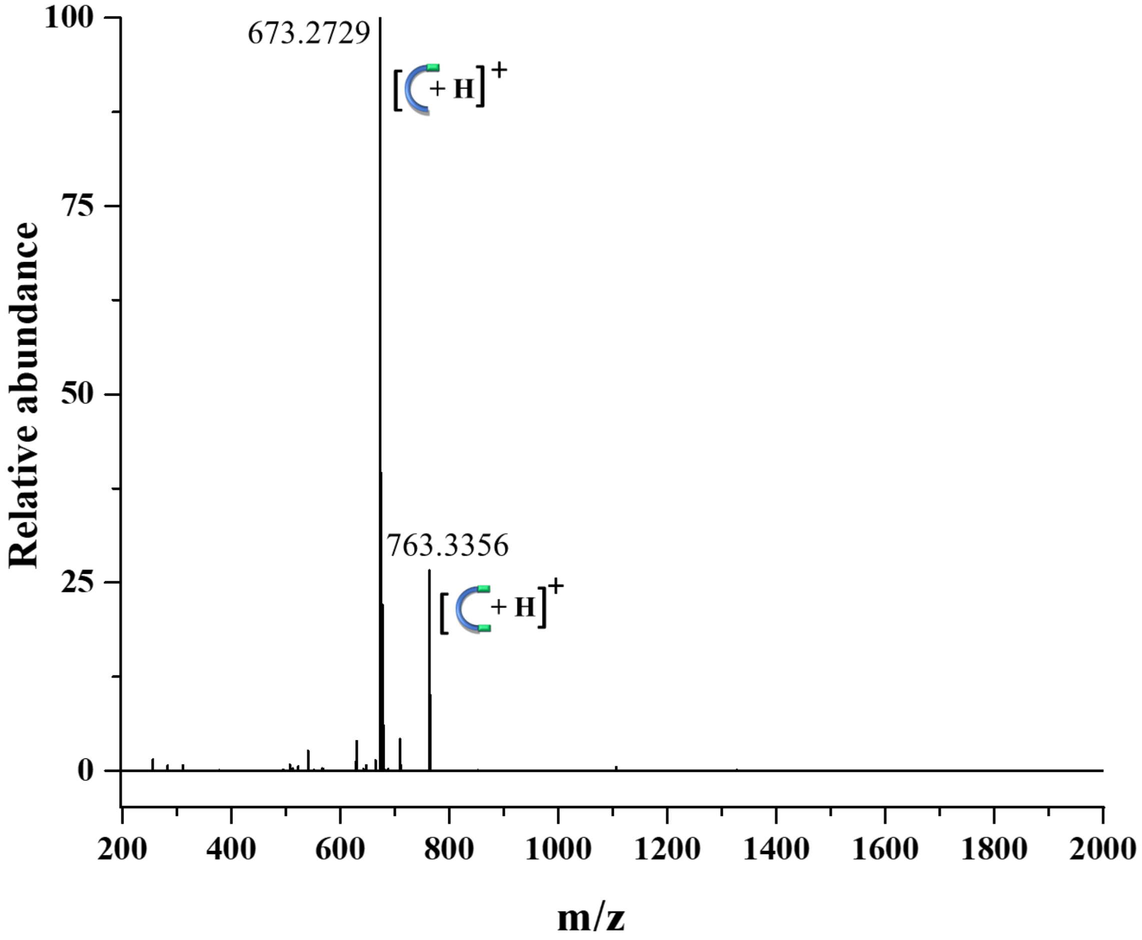

Figure S 33: ESI-TOF spectrum of macrocycle **12**. The macrocycle is dissociating in the mass condition. In this case, only the fragments were observed.

3.6.3 Macrocycle 13

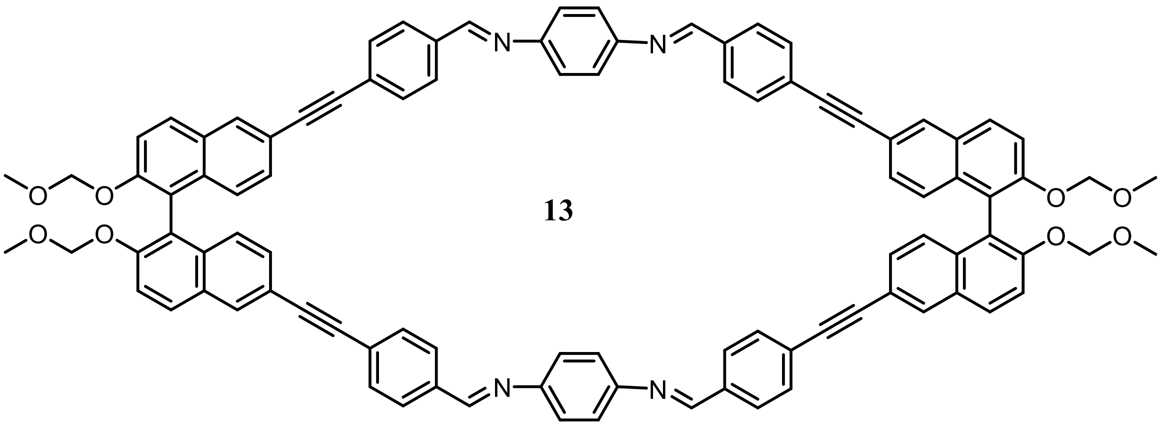

13

^{1}H NMR (500 MHz, $CDCl_3$, 298 K) δ 8.52 (s, 4 H), 8.13 (s, 4 H), 7.96 (d, ^{3}J = 9.7 Hz, 4 H), 7.90 (d, ^{3}J = 8.1 Hz, 8 H), 7.86 (d, ^{3}J = 8.67 Hz, 4 H), 7.67 (d, ^{3}J = 8.12 Hz, 8 H), 7.62 (d, ^{3}J =

8.4 Hz, 4 H), 7.30 (s, 8 H), 7.15 (d, ^3J = 9.1 Hz, 4 H), 5.13 (d, ^3J = 7.1 Hz, 4 H), 5.02 (d, ^3J = 6.32 Hz, 4 H), 3.18 (s, 12 H) ppm.

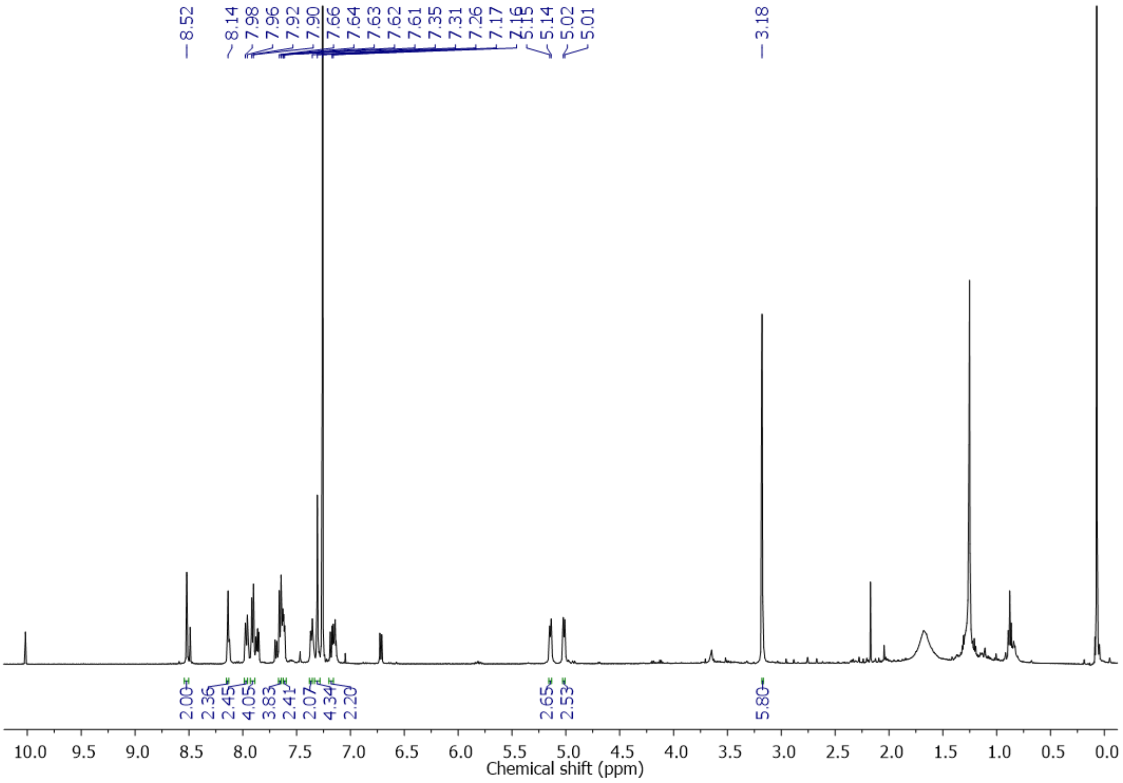

Figure S 34: ^{1}H NMR (500 MHz, $CDCl_3$, 298 K) spectrum of macrocycle **13**.

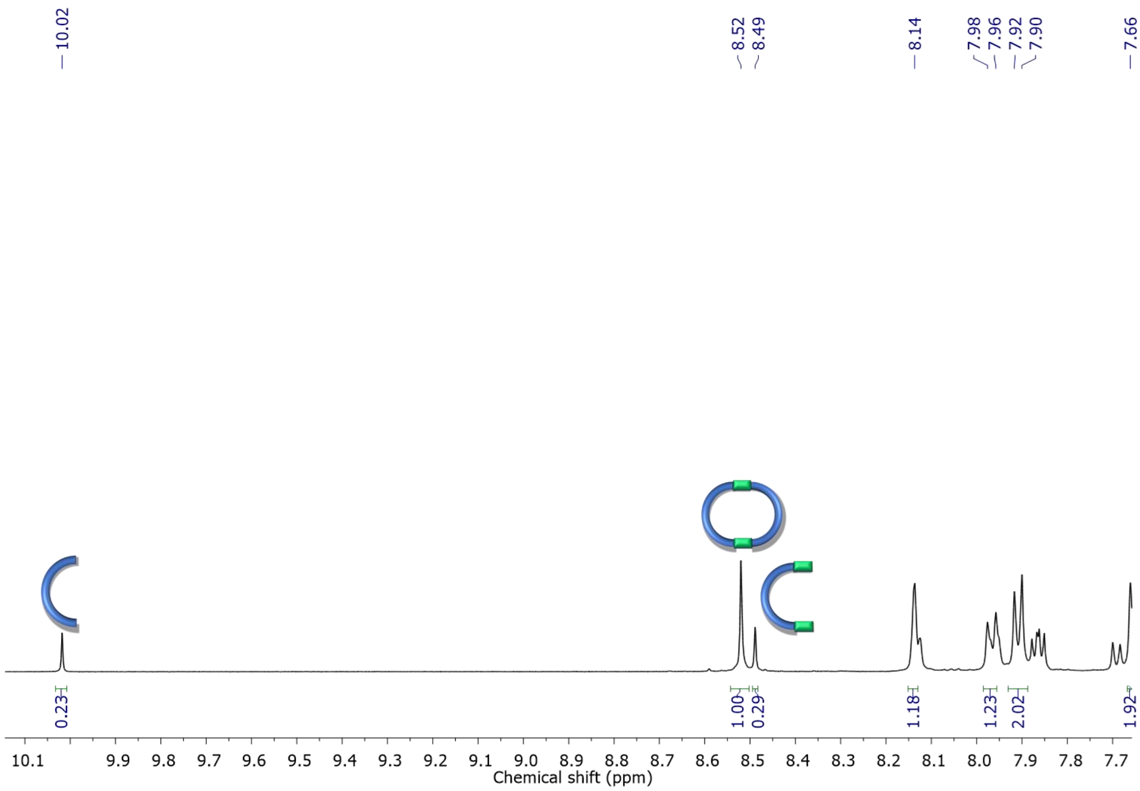

Figure S 35: Partial ^{1}H NMR (500 MHz, $CDCl_3$, 298 K) spectrum of macrocycle **13** showing the ratio between the macrocycle, unreacted aldehyde and half of the macrocycle.

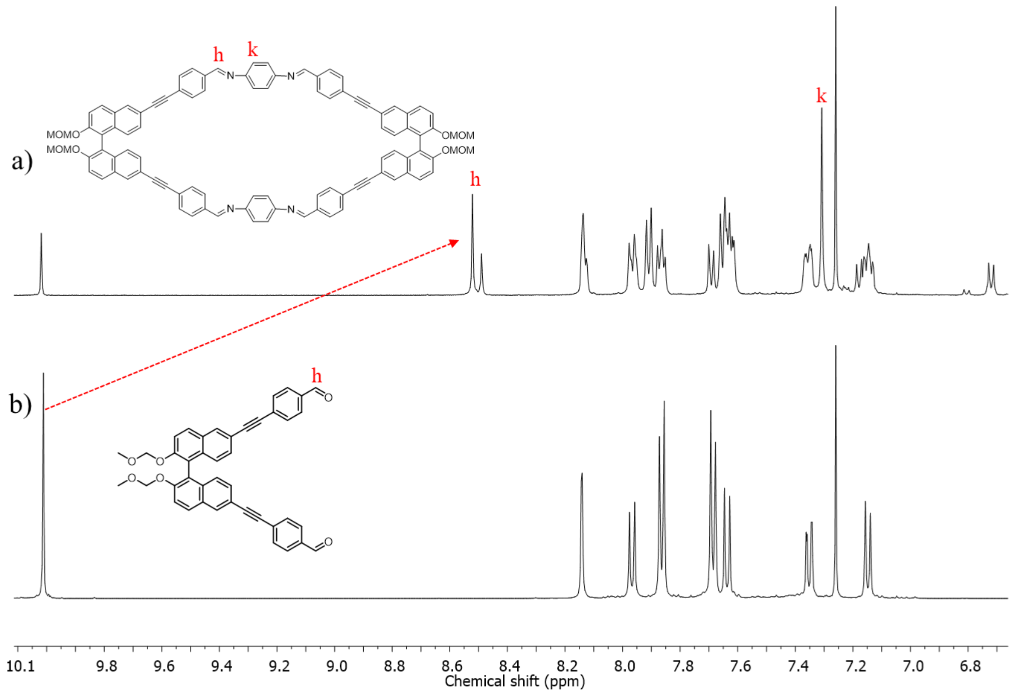

Figure S 36: Partial ^{1}H NMR (500 MHz, $CDCl_3$, 298 K) spectrum of a) macrocycle **13**; b) aldehyde **2**.

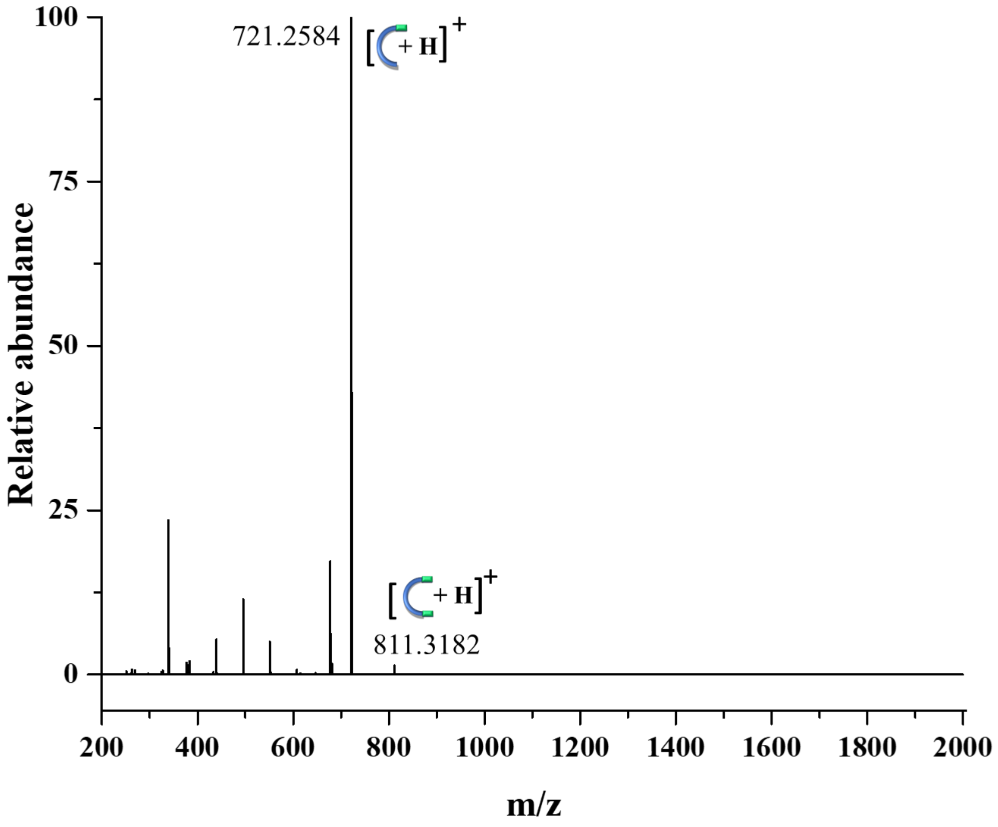

Figure S 37: ESI-TOF spectrum of macrocycle **13**. The macrocycle is dissociating in the mass condition. In this case, only the fragments were observed.

3.6.4 Synthesis of macrocycle 14

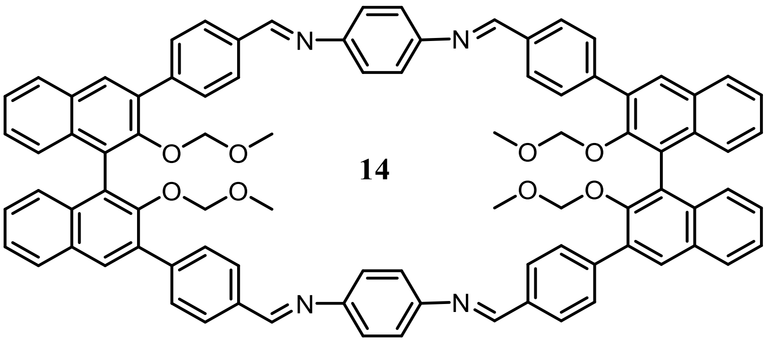

14

^{1}H NMR (500 MHz, $CDCl_3$, 298 K) δ 8.61 (s, 4 H), 8.1-7.85 (m, 24 H), 7.49-7.41 (m, 4 H), 7.35 (s, 8 H), 7.35-7.27 (m, 8 H), 4.48-4.33 (m, 8 H), 2.41 (s, 12 H) ppm.

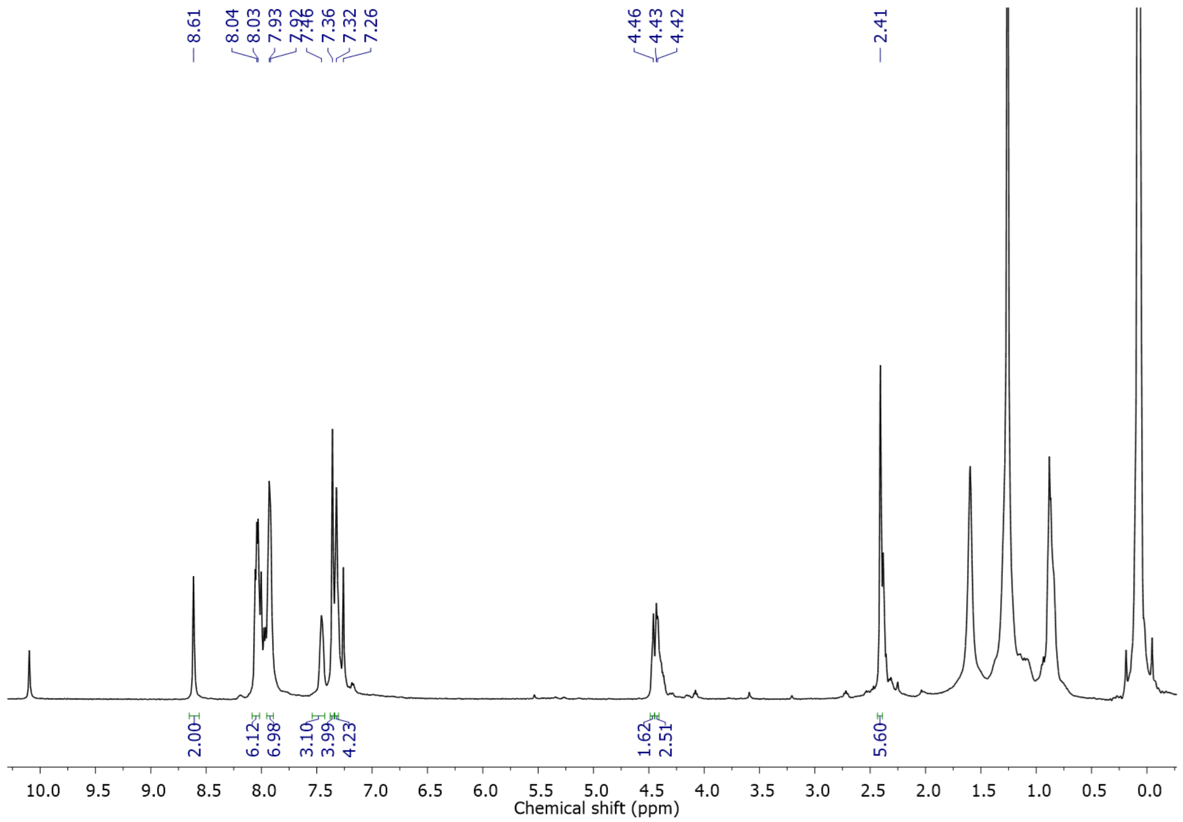

Figure S 38: ^{1}H NMR (500 MHz, $CDCl_3$, 298 K) spectrum of compound **14**.

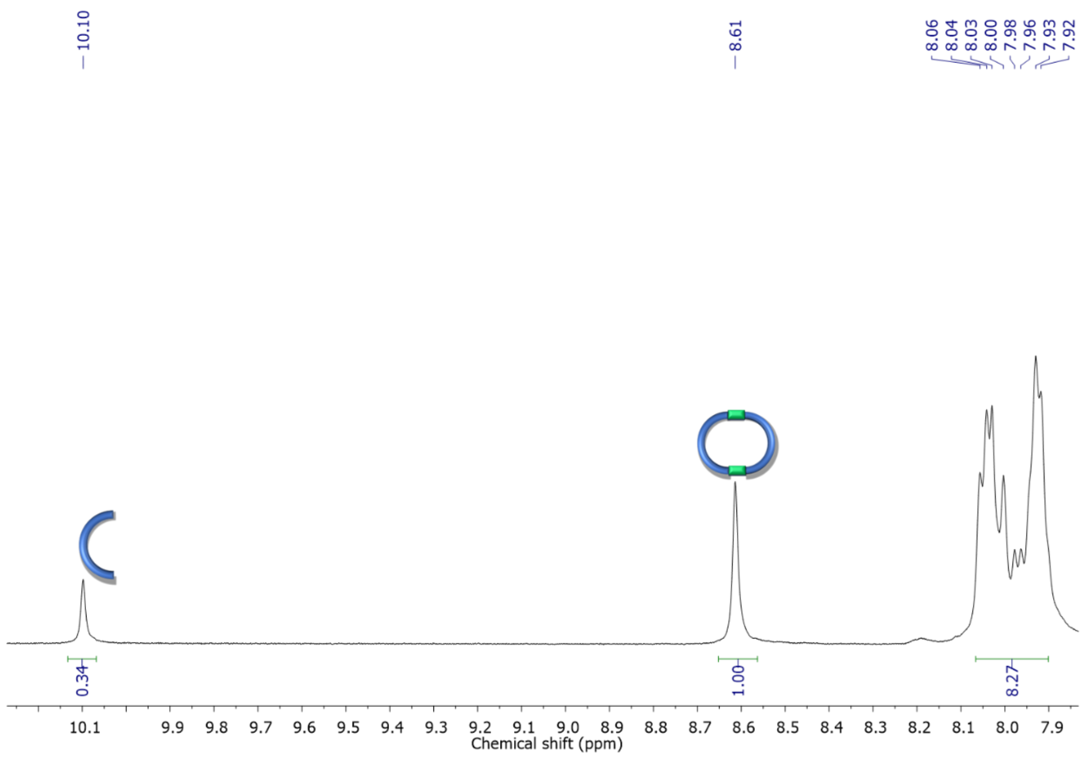

Figure S 39: Partial ^{1}H NMR (500 MHz, $CDCl_3$, 298 K) spectrum of macrocycle **14** showing the ratio between the macrocycle and unreacted aldehyde.

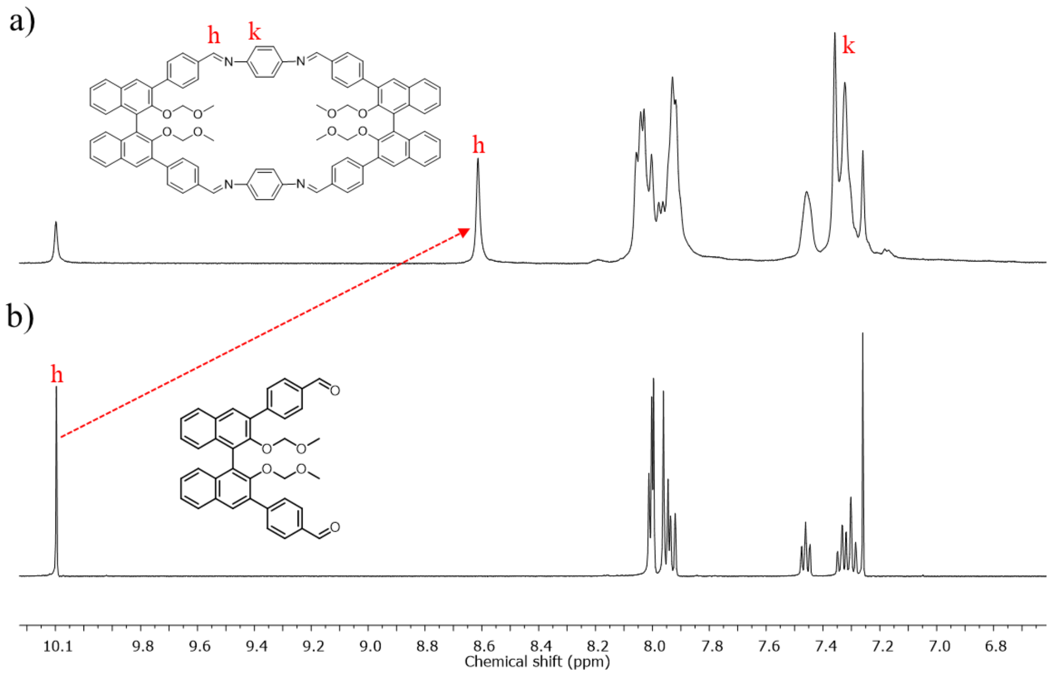

Figure S 40: Partial ^{1}H NMR (500 MHz, $CDCl_3$, 298 K) spectrum of a) macrocycle **14**; b) aldehyde **3**.

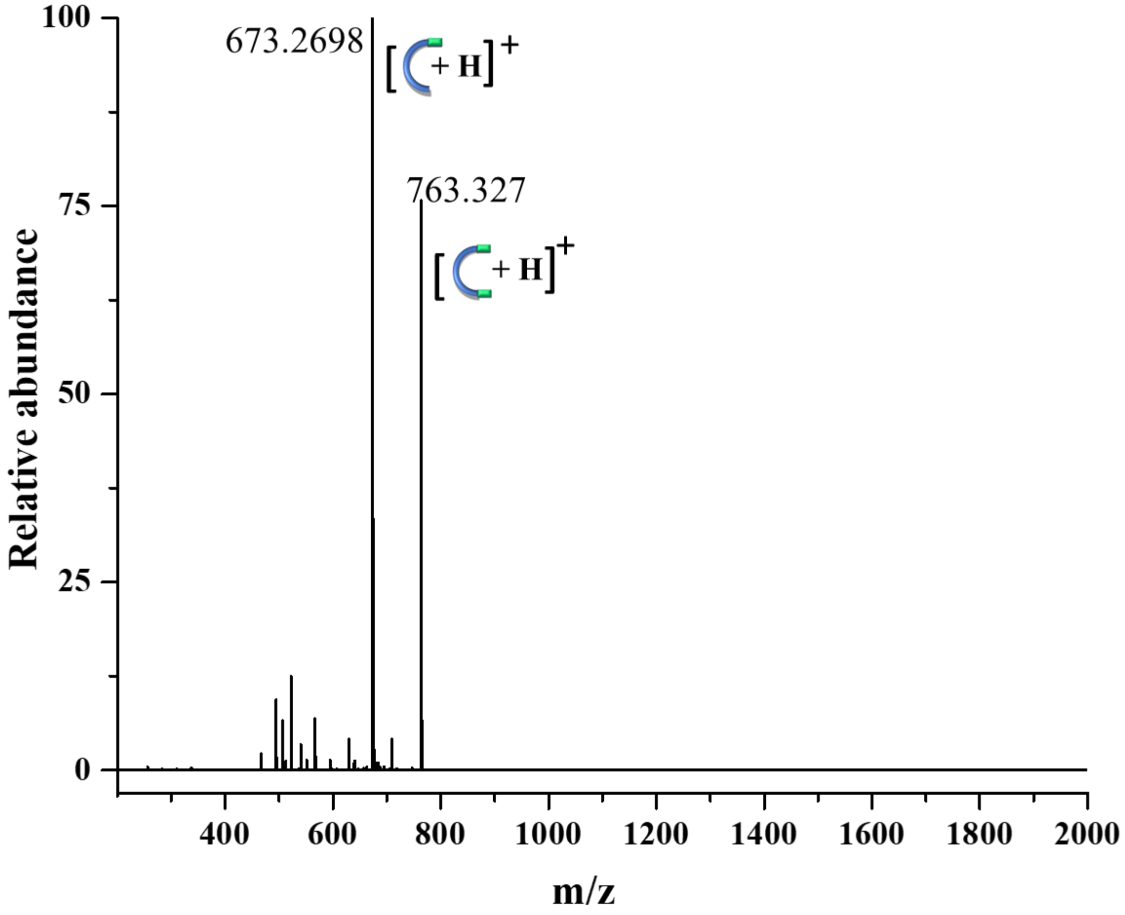

Figure S 41: ESI-TOF spectrum of macrocycle **14**. The macrocycle is dissociating in the mass condition. In this case, only the fragments were observed.

3.6.5 Synthesis of macrocycle 15

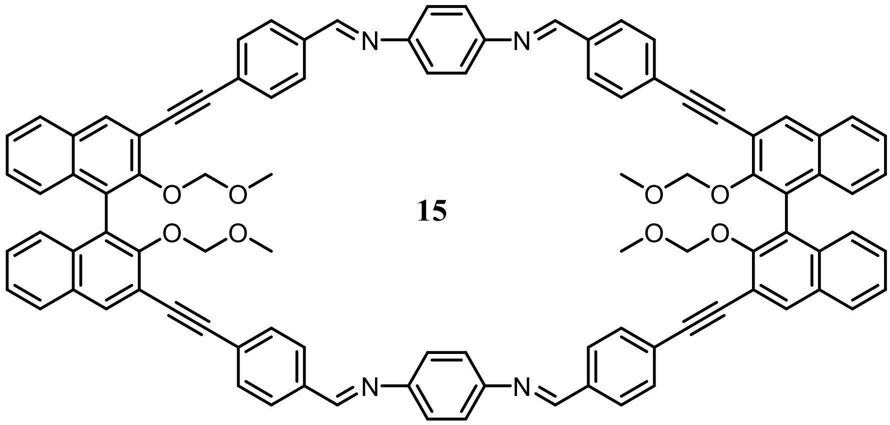

^{1}H NMR (500 MHz, $CDCl_3$) δ 8.53 (s, 4 H), 8.28 (s, 4 H), 7.92 (d, ^3J = 8.1 Hz, 8 H,), 7.91-7.85 (m, 8 H), 7.67 (d, ^3J = 8.1 Hz, 8 H), 7.36-7.32 (m, 4 H), 7.34 (d, ^3J = 8.1 Hz, 4 H), 7.31 (s, 8 H), 5.24-5.19 (m, 4 H), 5.01-4.96 (m, 4 H), 2.55 (s, 12 H) ppm.

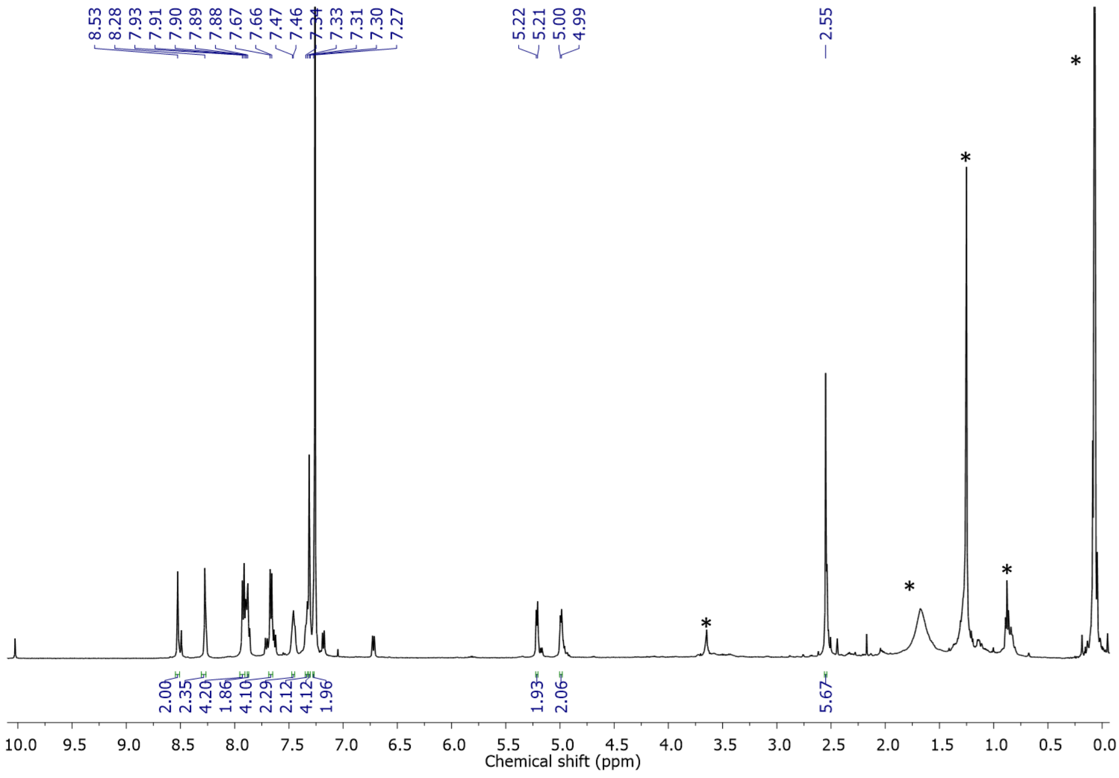

Figure S 42: ^{1}H NMR (500 MHz, $CDCl_3$, 298 K) spectrum of compound **15** (Asterisks represent the solvent impurities).

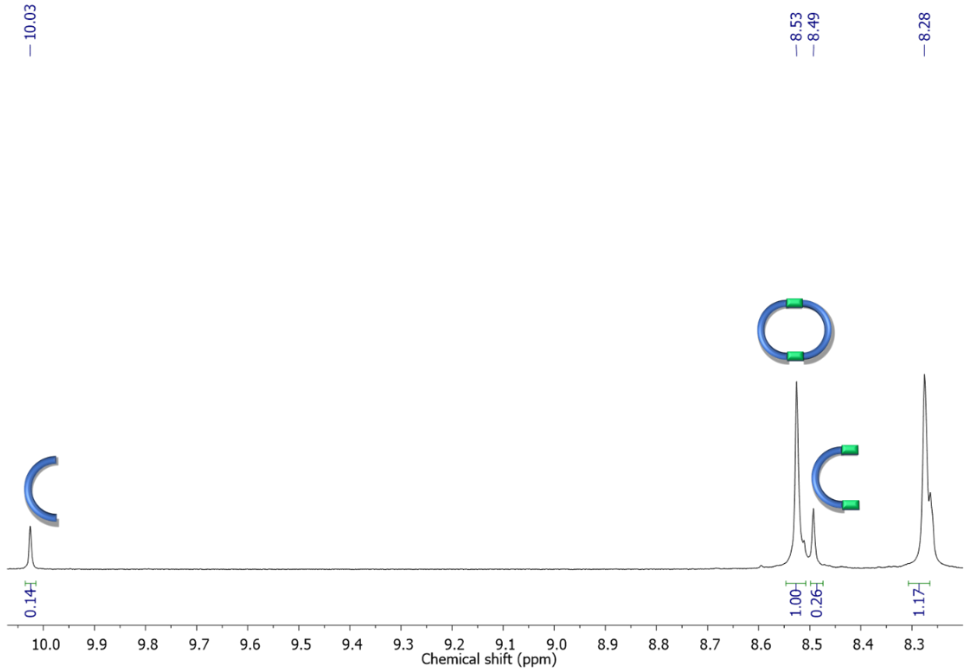

Figure S 43: Partial ^{1}H NMR (500 MHz, $CDCl_3$, 298 K) spectrum of macrocycle **15** showing the ratio between the macrocycle and unreacted aldehyde.

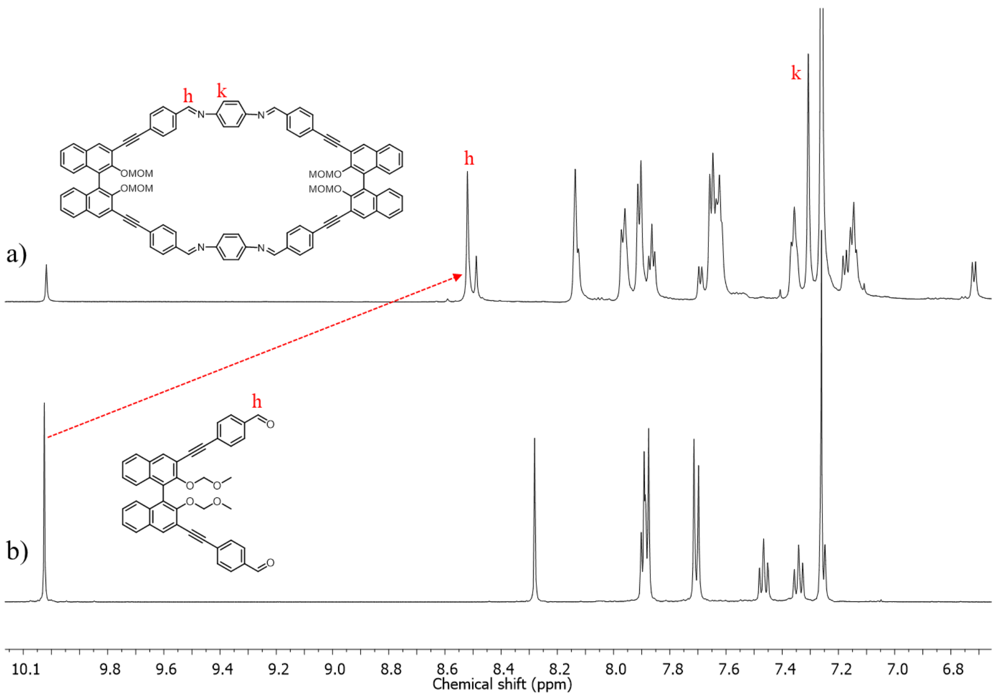

Figure S 44: Partial ^{1}H NMR (500 MHz, $CDCl_3$, 298 K) spectrum of a) macrocycle **15**; b) aldehyde **4**. The small set of peaks belongs to half of the macrocycle **19**.

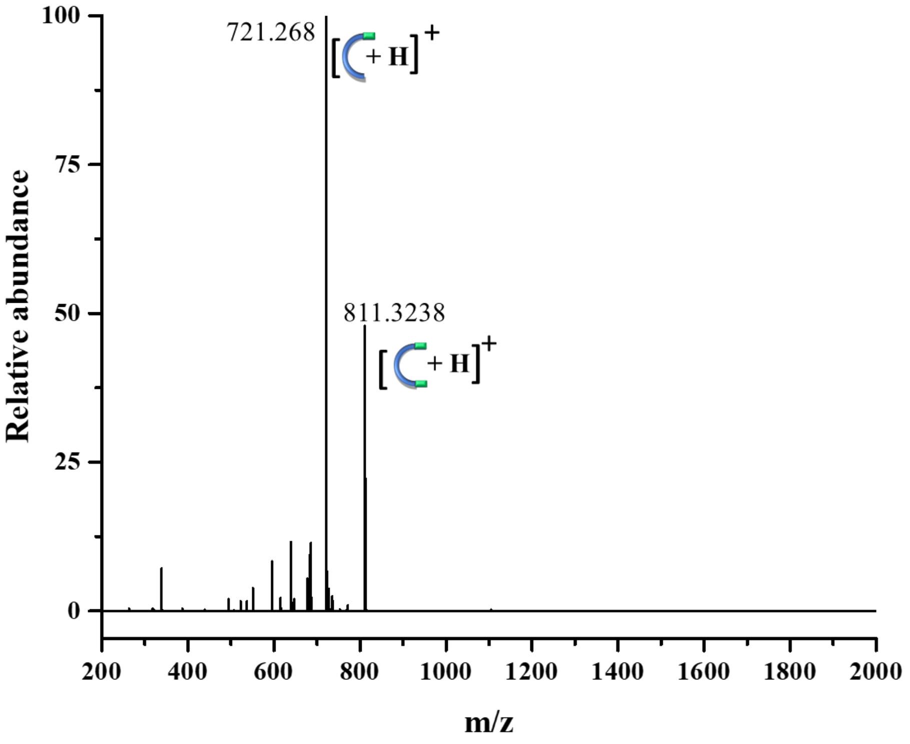

Figure S 45: ESI-TOF spectrum of macrocycle **15**. The macrocycle is dissociating in the mass condition. In this case, only the fragments were observed.

3.7 Self-sorting during macrocycle formation from aromatic bisamine

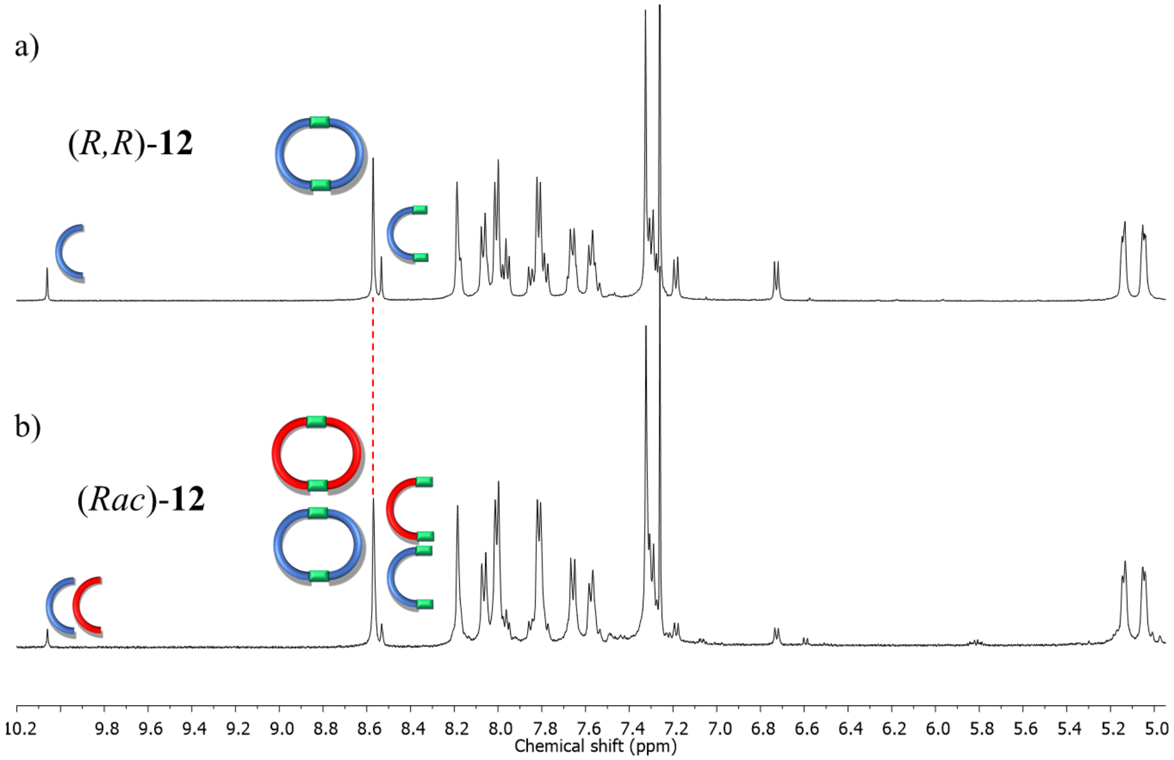

Figure S 46: Partial ^{1}H NMR (500 MHz, $CDCl_3$, 298 K) comparison of macrocycle **12** obtained from a) (*R*)-**1** and **7**; b) (±)-**1** and **7**. The small imine-derived peak at 8.53 ppm belongs to half of the macrocycle **16**. The presence of one set of sharp imine-derived signals indicates the formation of two homochiral macrocycles (*R*,*R*-**12** and *S*,*S*-**12**) only. The diastereomer (*R*,*S*)-**12** did not form.

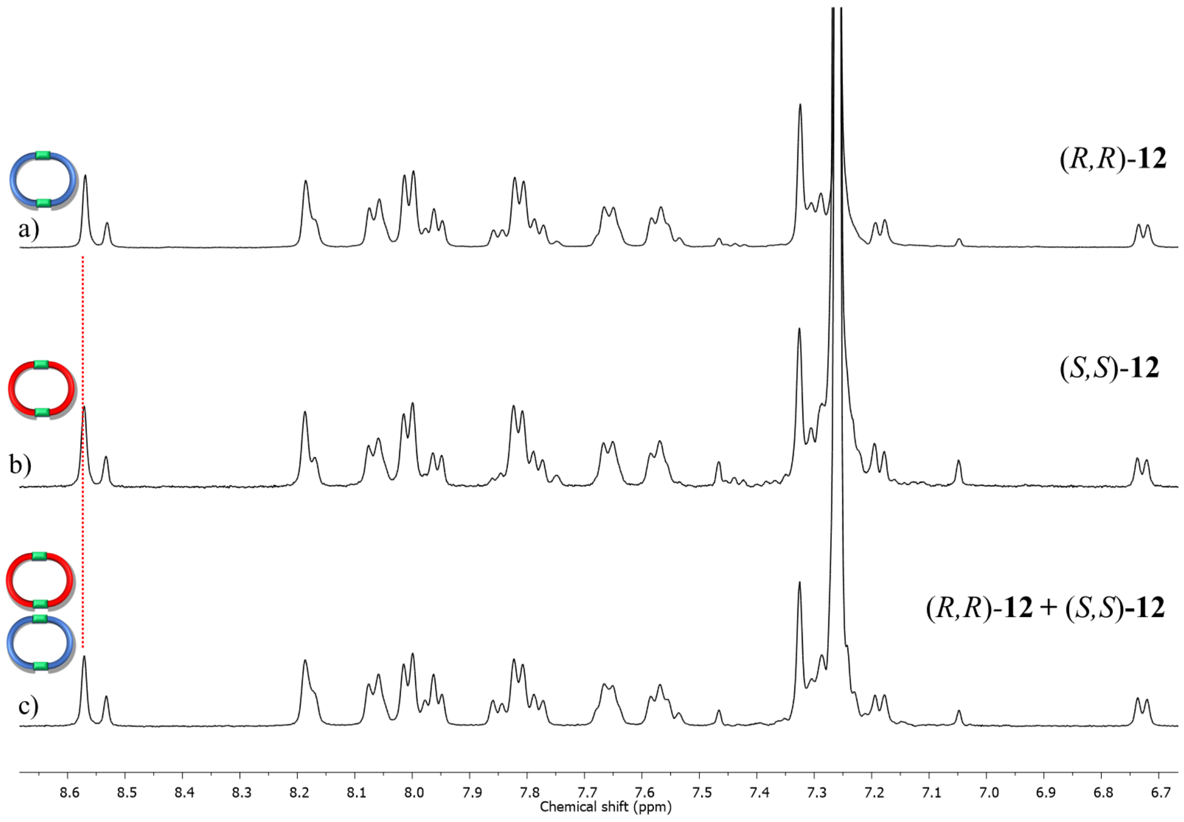

Figure S 47: Partial ^{1}H NMR (500 MHz, $CDCl_3$, 298 K) showing the comparison between the macrocycles obtained from the reaction of **7** with a) (*R*)-**1** b) (*S*)-**1** and c) upon mixing the two macrocycles in chloroform and reflux for 48 hrs

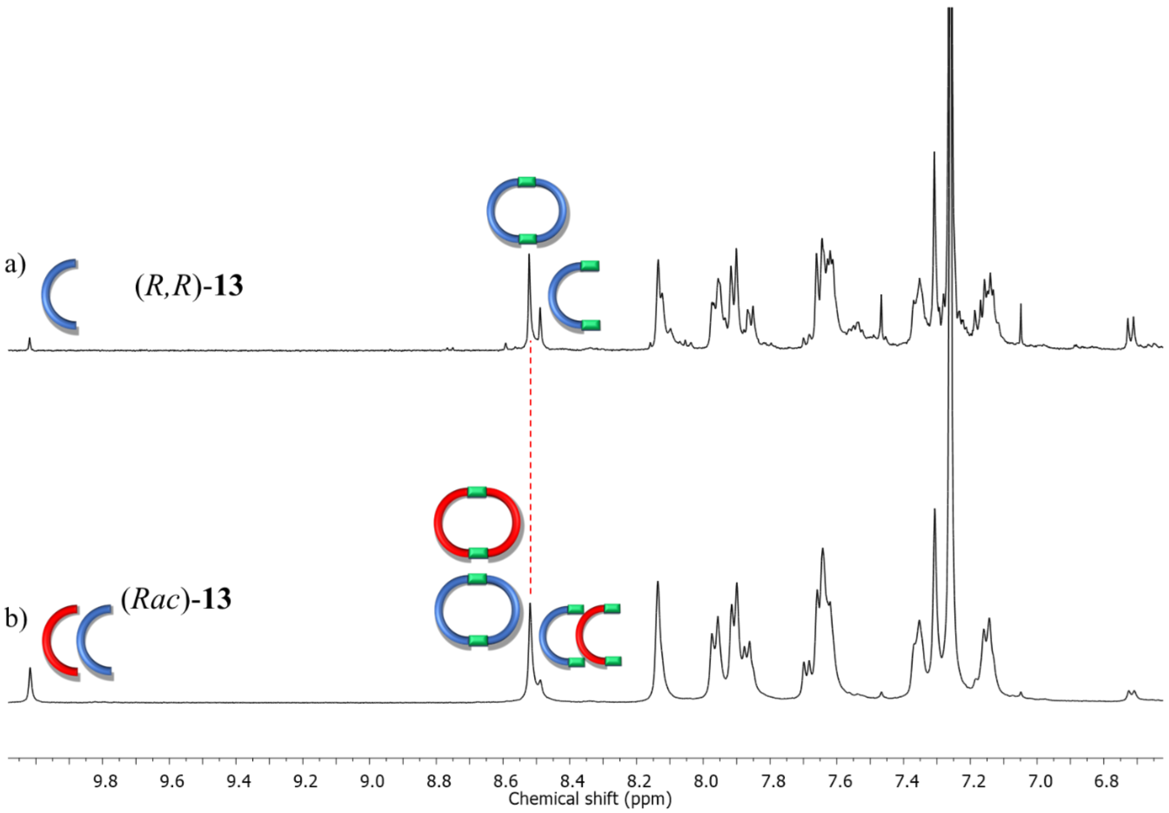

Figure S 48: Partial ^{1}H NMR (500 MHz, $CDCl_3$, 298 K) comparison of macrocycle **13** obtained from a) (*R*)-**2** and **7**; b) (±)-**2** and **7**. The small imine-derived peak at 8.49 ppm belongs to half of the macrocycle **17**. The presence of one set of sharp imine-derived signals indicates the formation of two homochiral macrocycles (*R*,*R*-**13,** *S*,*S*-**13**) only. The diastereomer (*R*,*S*)-**13** did not form.

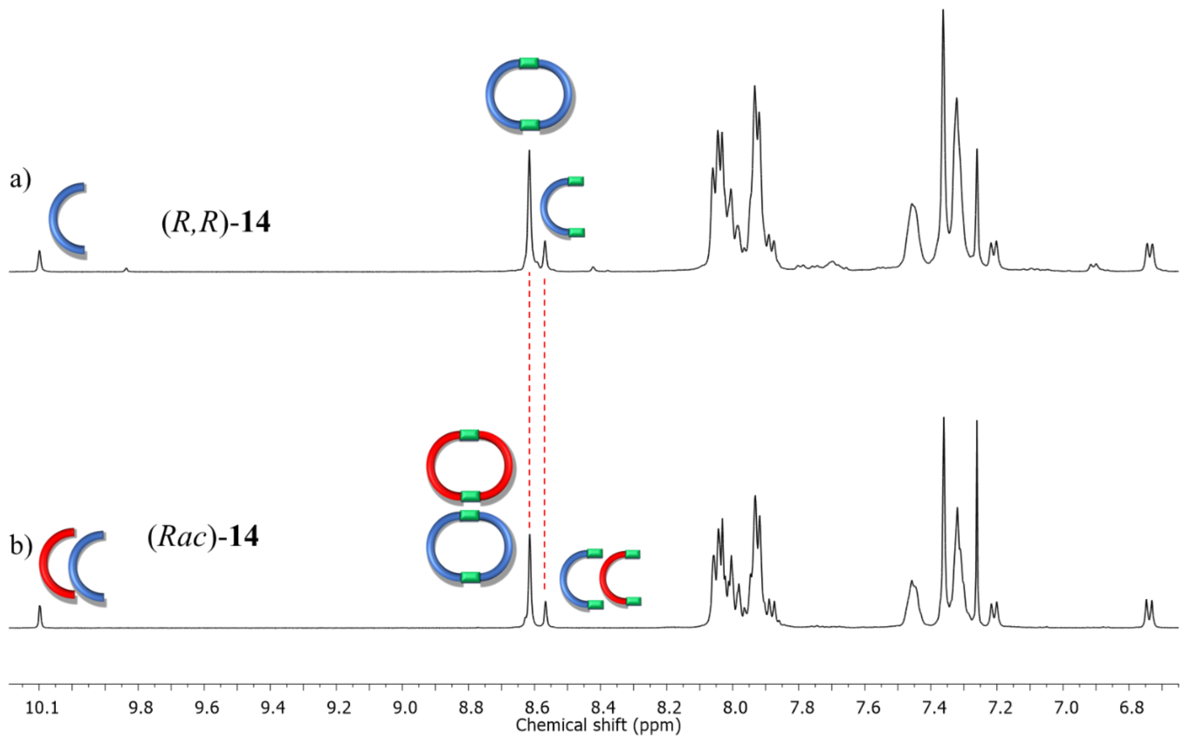

Figure S 49: Partial ^{1}H NMR (500 MHz, $CDCl_3$, 298 K) comparison of macrocycle **14** obtained from a) (*R*)-**3** and **7**; b) (±)-**3** and **7**. The small imine-derived peak at 8.56 ppm belongs to half of the macrocycle **18**. The presence of one set of sharp imine-derived signals indicates the formation of two homochiral macrocycles (*R*,*R*-**14,** *S*,*S*-**14**) only. The diastereomer (*R*,*S*)-**14** did not form.

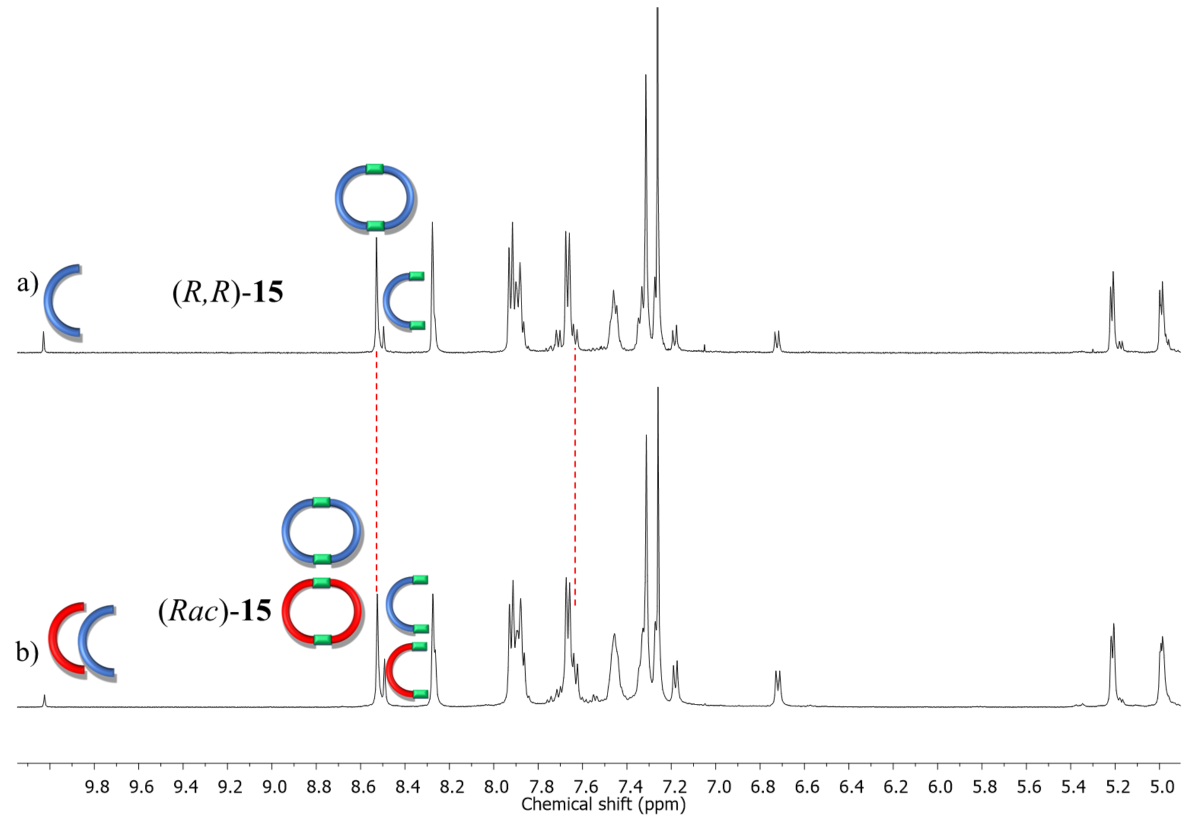

Figure S 50: Partial ^{1}H NMR (500 MHz, $CDCl_3$, 298 K) comparison of macrocycle **15** obtained from a) (*R*)-**4** and **7**; b) (±)-**4** and **7**. The small imine-derived peak at 8.49 ppm belongs to half of the macrocycle **19**. The presence of a single set of sharp imine signals indicates the exclusive formation of the two homochiral macrocycles (*R*,*R*-**15**, *S*,*S*-**15**), with no evidence for the formation of the diastereomer (*R*,*S*)-**15**.

3.8 Synthesis of half of the macrocycle

Half of the macrocycle was prepared by mixing the corresponding aldehyde (0.0046 mmol) and amine (0.014 mmol) in 0.5 mL CDCl3 and heating at 50˚C for 2 h.

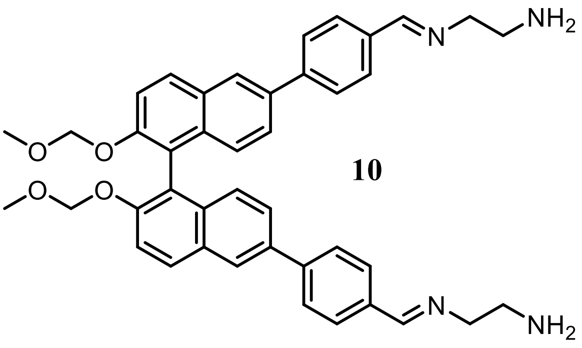

10

^{1}H NMR (500 MHz, $CDCl_3$, 298 K) δ 8.37 (s, 2 H), 8.13 (d, ^3J = 1.5 Hz, 2 H), 8.04 (d, ^3J = 9 Hz, 2 H), 7.82 (d, ^3J = 8.2 Hz, 4 H), 7.74 (d, ^3J = 8.2 Hz, 4 H), 7.63 (d, ^3J = 8.9 Hz, 2 H), 7.53 (d, ^3J = 9 Hz, 2 H), 7.27-7.25 (m, 2 H), 5.12 (d, ^2J = 5.6 Hz, 2 H), 5.03 (d, ^3J = 5.6 Hz, 2 H), 3.71 (d, ^3J = 5.6 Hz, 4 H), 3.19 (s, 6 H), 3.05 (d, ^3J = 5.6 Hz, 4 H) ppm.

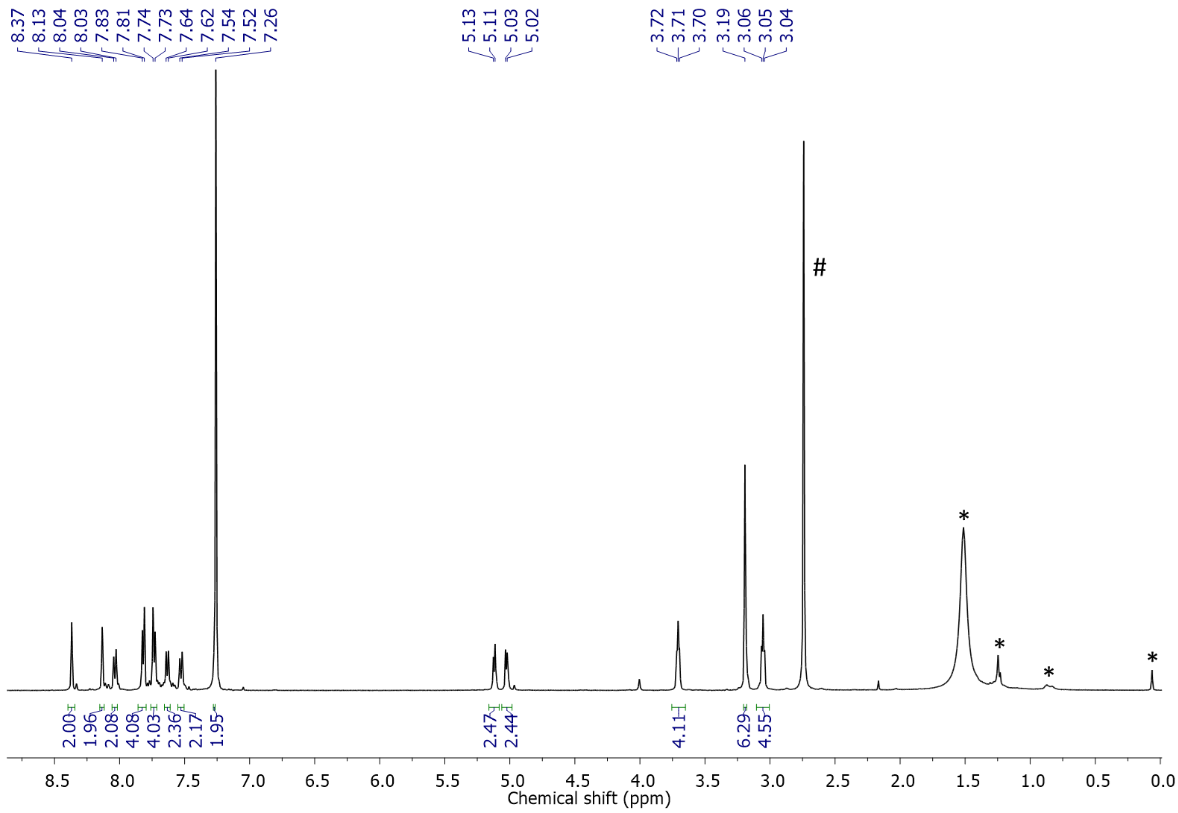

Figure S 51: ^{1}H NMR (500 MHz, $CDCl_3$, 298 K) spectrum of compound **10.** Peaks assigned with asterisks represent the solvent impurities, and the hashtag represents the remaining amine **5**.

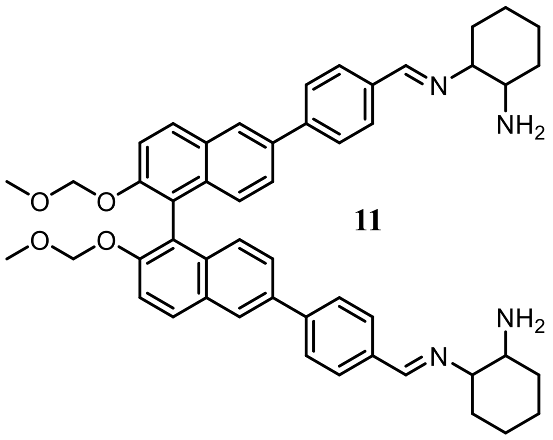

11

^{1}H NMR (500 MHz, $CDCl_3$, 298 K) δ 8.40 (s, 2 H), 8.12 (d, ^{3}J = 1.5 Hz, 2 H), 8.03 (d, ^{3}J = 9 Hz, 2 H), 7.82 (d, ^{3}J = 8.3 Hz, 4 H), 7.72 (d, ^{3}J = 8.3 Hz, 4 H), 7.63 (d, ^{3}J = 9 Hz, 2 H), 7.52 (dd, ^{3}J = 9 Hz, ^{2}J=1.5 Hz, 2 H), 7.27-7.25 (m, 2 H), 5.12 (d, ^{2}J = 5.6 Hz, 2 H), 5.02 (d, ^{3}J = 5.6 Hz, 2 H), 3.19 (s, 6 H), 2.96-2.91 (m, 2 H), 2.86-2.81 (m, 2 H), 1.94-1.92 (m, 2 H), 1.79-1.77 (m, 4 H), 1.44-1.42 (m, 6 H), 1.23-1.21 (m, 4 H) ppm.

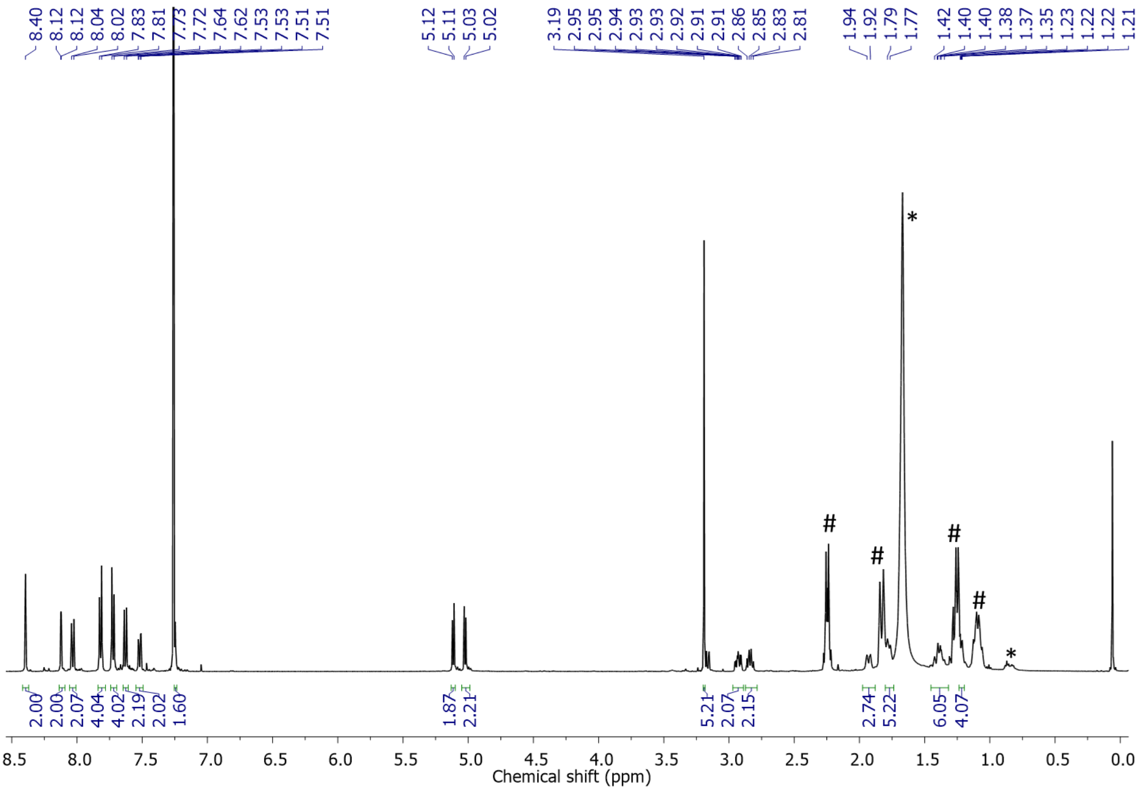

Figure S 52: ^{1}H NMR (500 MHz, $CDCl_3$, 298 K) spectrum of compound **11**. Peaks assigned with the hashtag represent the remaining amine **6**.

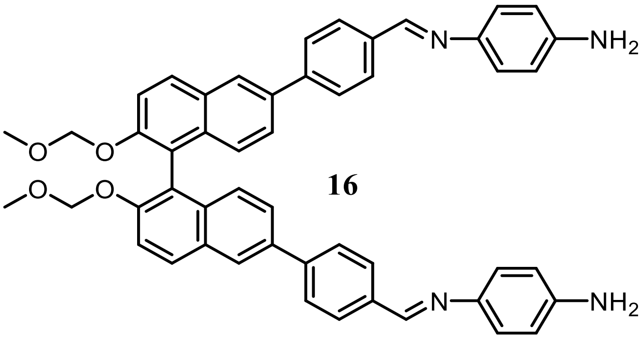

16

^{1}H NMR (500 MHz, $CDCl_3$, 298 K) δ 8.53 (s, 2 H), 8.17 (d, ^{3}J = 1.5 Hz, 2 H), 8.05 (d, ^{3}J = 9.3 Hz, 2 H) 7.96 (d, ^{3}J = 8.3 Hz, 4 H), 7.78 (d, ^{3}J = 8.3 Hz, 4 H), 7.65 (d, ^{3}J = 9.3 Hz, 2 H), 7.56 (dd, ^{3}J = 8.9 Hz, ^{4}J = 1.5 Hz, 2 H), 7.28 (d, ^{3}J = 8.6 Hz, 2 H), 7.19 (d, ^{3}J = 8.6 Hz, 4 H), 6.73 (d, ^{3}J = 8.6 Hz, 4 H), 5.13 (d, ^{2}J = 6.8 Hz, 2 H), 5.04 (d, ^{3}J = 6.8 Hz, 2 H), 3.70 (br, 4 H), 3.21 (s, 6 H) ppm.

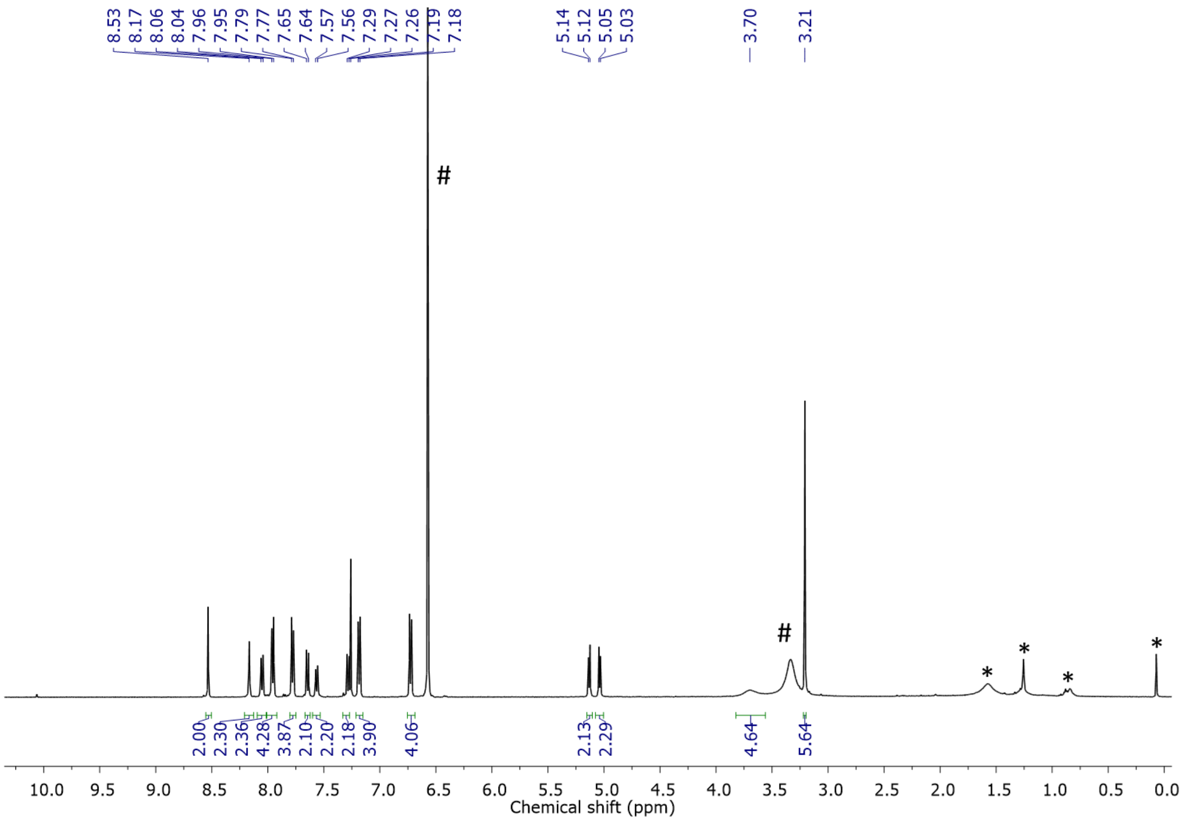

Figure S 53: ^{1}H NMR (500 MHz, $CDCl_3$, 298 K) spectrum of compound **16**. Peaks assigned with asterisks represent the solvent impurities, and with hashtag represents the remaining amine **7**.

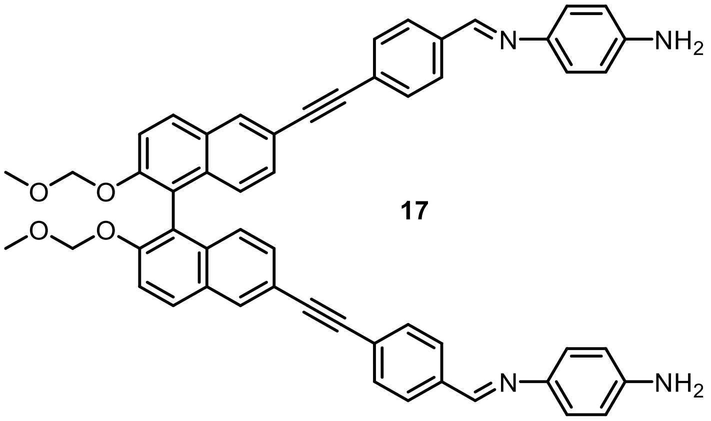

17

^{1}H NMR (500 MHz, $CDCl_3$, 298 K) δ 8.49 (s, 2 H), 8.12 (d, ^{3}J=1.5 Hz, 2 H), 7.96 (d, ^{3}J = 9 Hz, 2 H) 7.86 (d, ^{3}J = 8.6 Hz, 4 H), 7.63-7.61 (m, 6 H), 7.35 (dd, ^{3}J = 8.7 Hz, ^{2}J = 1.5 Hz, 2 H), 7.18 (d, ^{3}J = 8.9 Hz, 4 H), 7.14 (d, ^{3}J = 8.5 Hz, 2 H), 6.72 (d, ^{3}J = 8.9 Hz, 4 H), 5.14 (d, ^{2}J = 6.8 Hz, 2 H), 5.01 (d, ^{3}J = 6.8 Hz, 2 H), 4.32 (br, 4 H), 3.10 (s, 6 H) ppm.

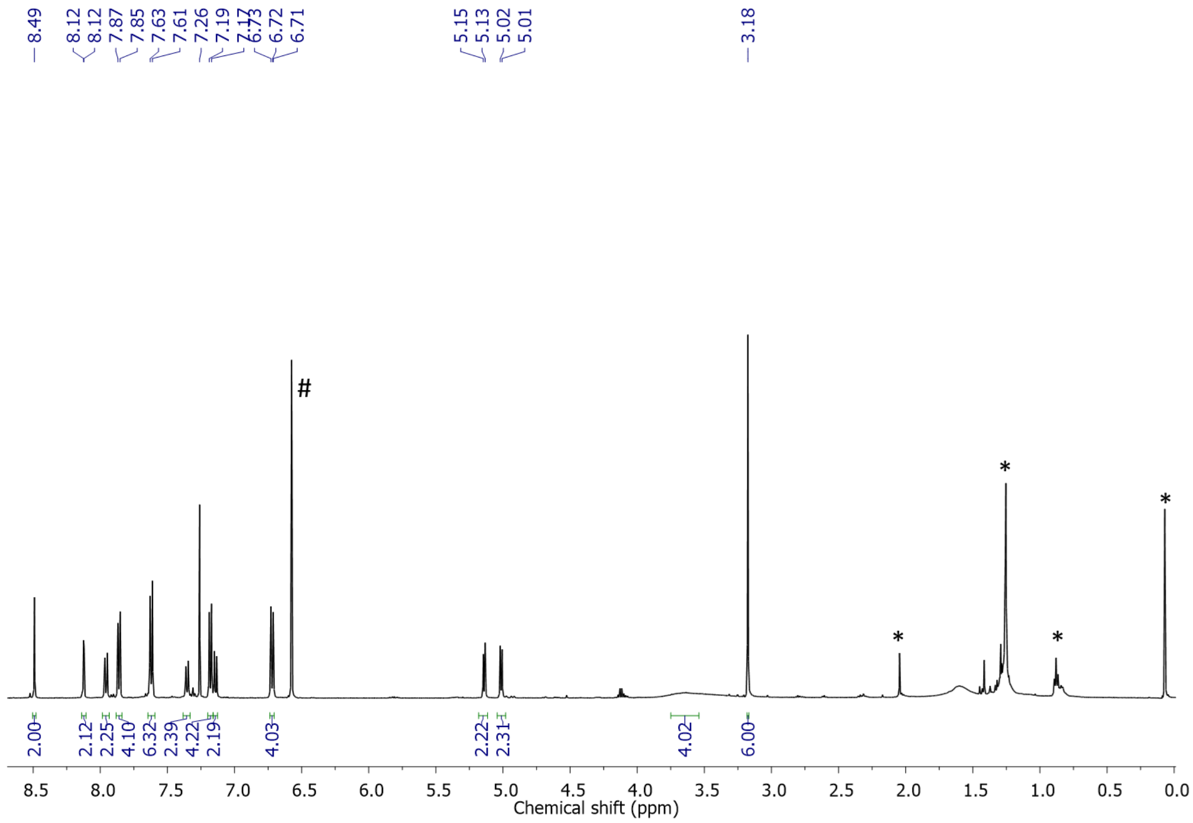

Figure S 54: ^{1}H NMR (500 MHz, $CDCl_3$, 298 K) spectrum of compound **17.** Peaks assigned with asterisks represent the solvent impurities, and with hashtag represents the remaining amine **7**.

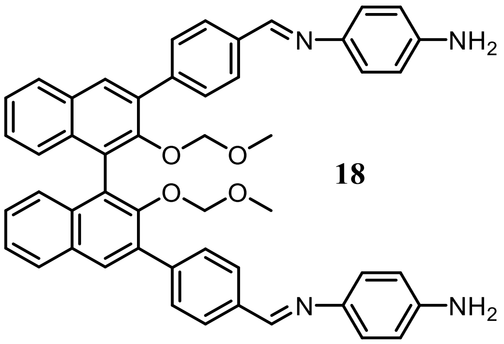

18

^{1}H NMR (500 MHz, $CDCl_3$, 298 K) δ 8.56 (s, 2 H), 8.00-7.98 (m, 6 H), 7.92 (d, ^{3}J = 8.6 Hz, 2 H) 7.87 (d, ^{3}J = 7.8 Hz, 4 H) 7.47-7.42 (m, 2 H), 7.29 (m, 4 H), 7.21 (d, ^{3}J = 7.8 Hz, 4 H), 6.74 (d, ^{3}J = 7.8 Hz, 4 H), 4.44 (d, ^{3}J = 6.2 Hz, 2 H), 4.40 (d, ^{3}J = 6.2 Hz, 2 H), 3.71 (br, 4 H), 2.37 (s, 6 H).

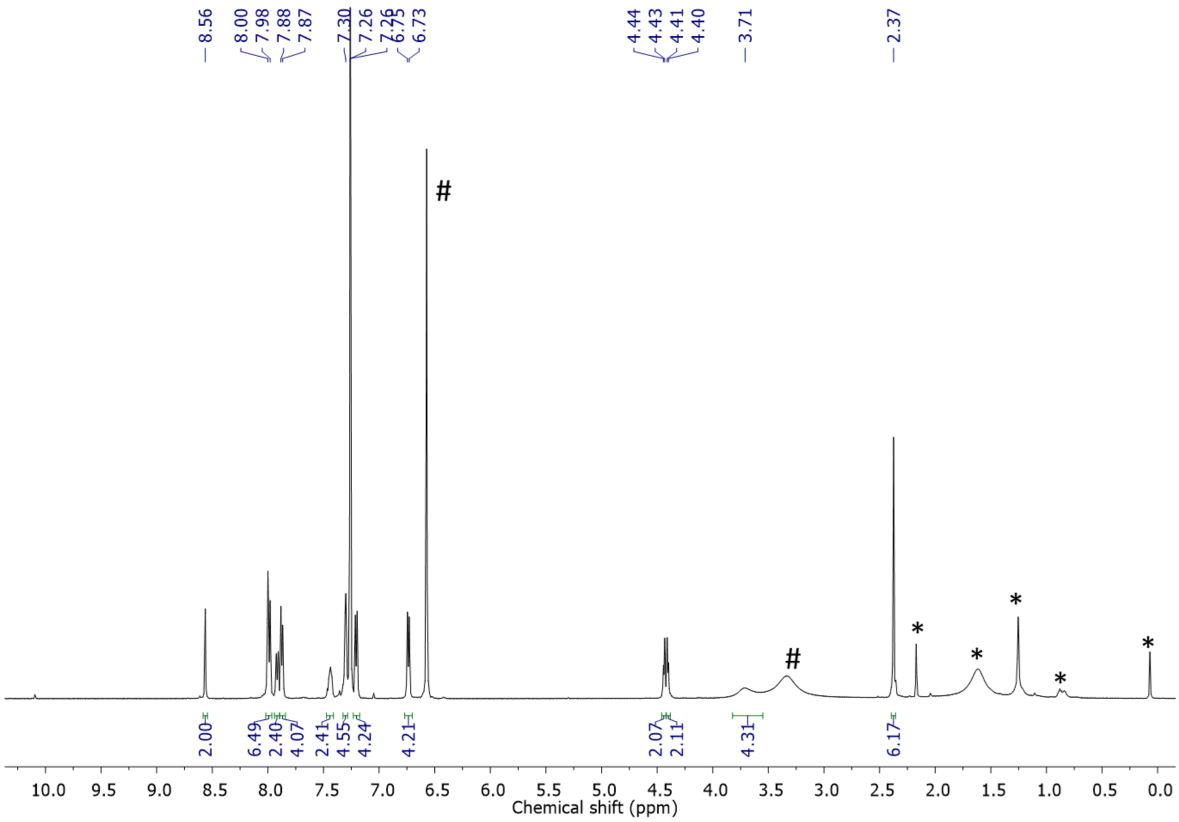

Figure S 55: ^{1}H NMR (500 MHz, $CDCl_3$, 298 K) spectrum of compound **18.** Peaks assigned with asterisks represent the solvent impurities, and with hashtag represents the remaining amine **7**.

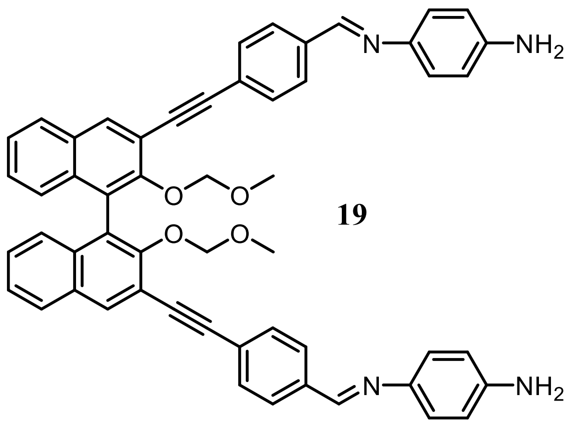

19

^{1}H NMR (500 MHz, $CDCl_3$, 298 K) δ 8.49 (s, 2 H), 8.26 (s, 2 H), 7.88-7.86 (m, 6 H) 7.63 (d, ^3J = 8 Hz, 4 H) 7.46-7.43 (m, 2 H), 7.34-7.31 (m, 2 H), 7.26 (m, 2 H), 7.18 (d, ^3J = 7.5 Hz, 4 H), 6.72 (d, ^3J = 7.5 Hz, 4 H), 5.20 (d, ^3J = 5.9 Hz, 2 H), 4.98 (d, ^3J = 5.9 Hz, 2 H), 3.5 (br, 4 H), 2.50 (s, 6 H) ppm.

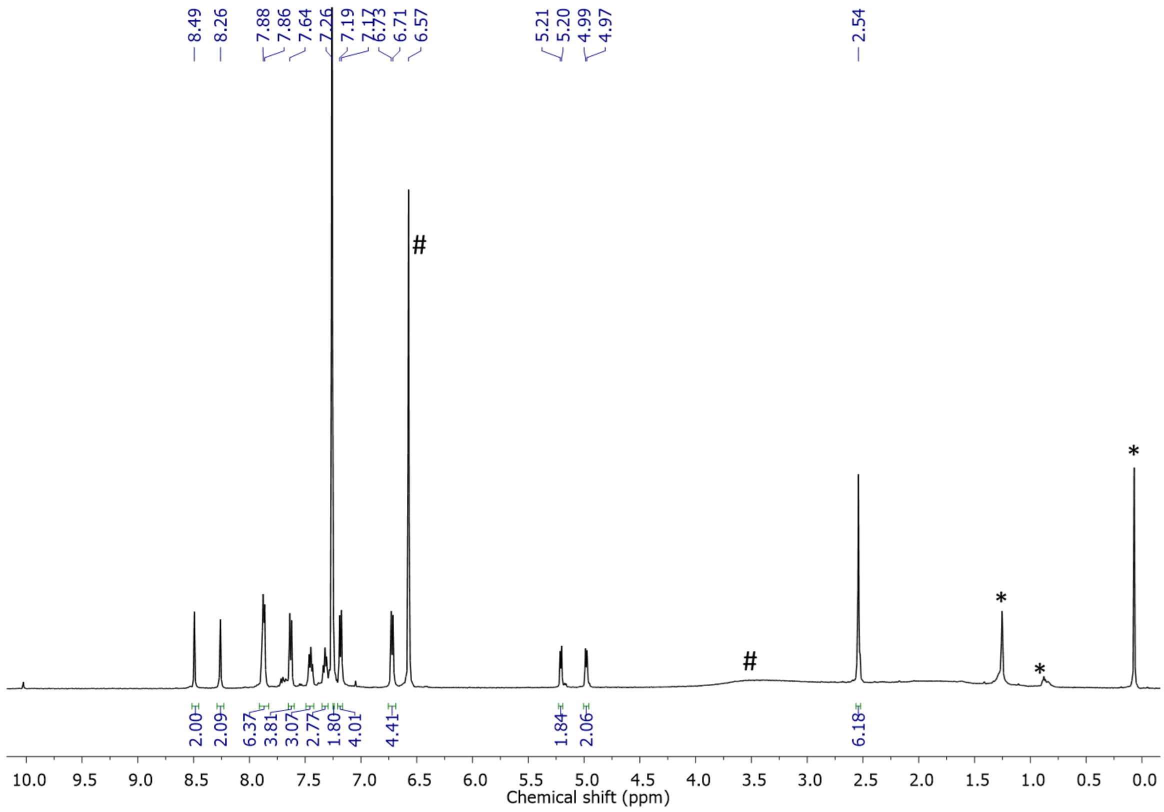

Figure S 56: ^{1}H NMR (500 MHz, $CDCl_3$, 298 K) spectrum of compound **19.** Peaks assigned with asterisks represent the solvent impurities, and with hashtag represents the remaining amine **7**.

3.9 DFT optimized structures:

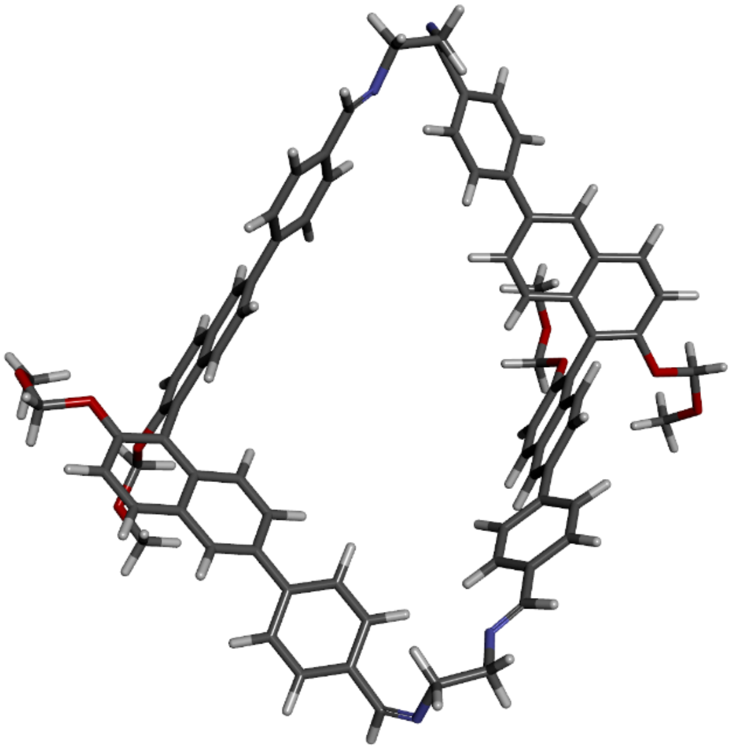

Figure S 57: Geometry optimized structure of macrocycle (*R*,*R*)-**8** at B3LYP/6-311G level of theory.

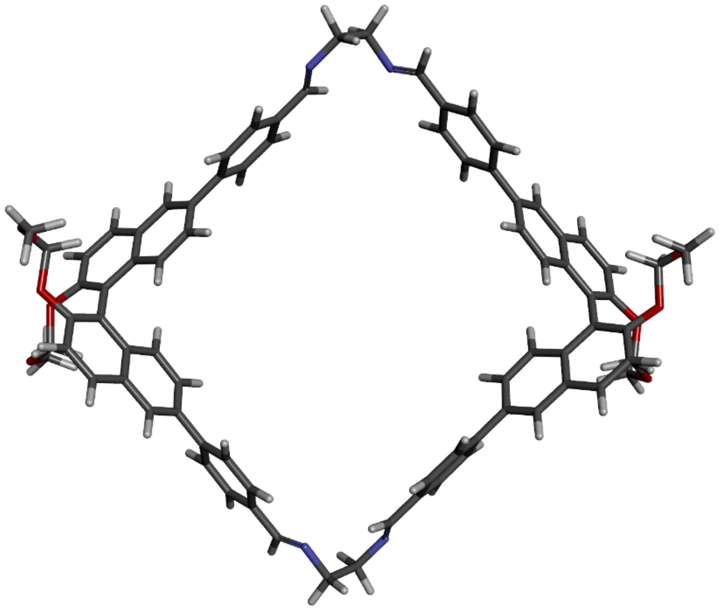

Figure S 58: Geometry optimized structure of macrocycle (*R*,*S*)-**8** at B3LYP/6-311G level of theory.

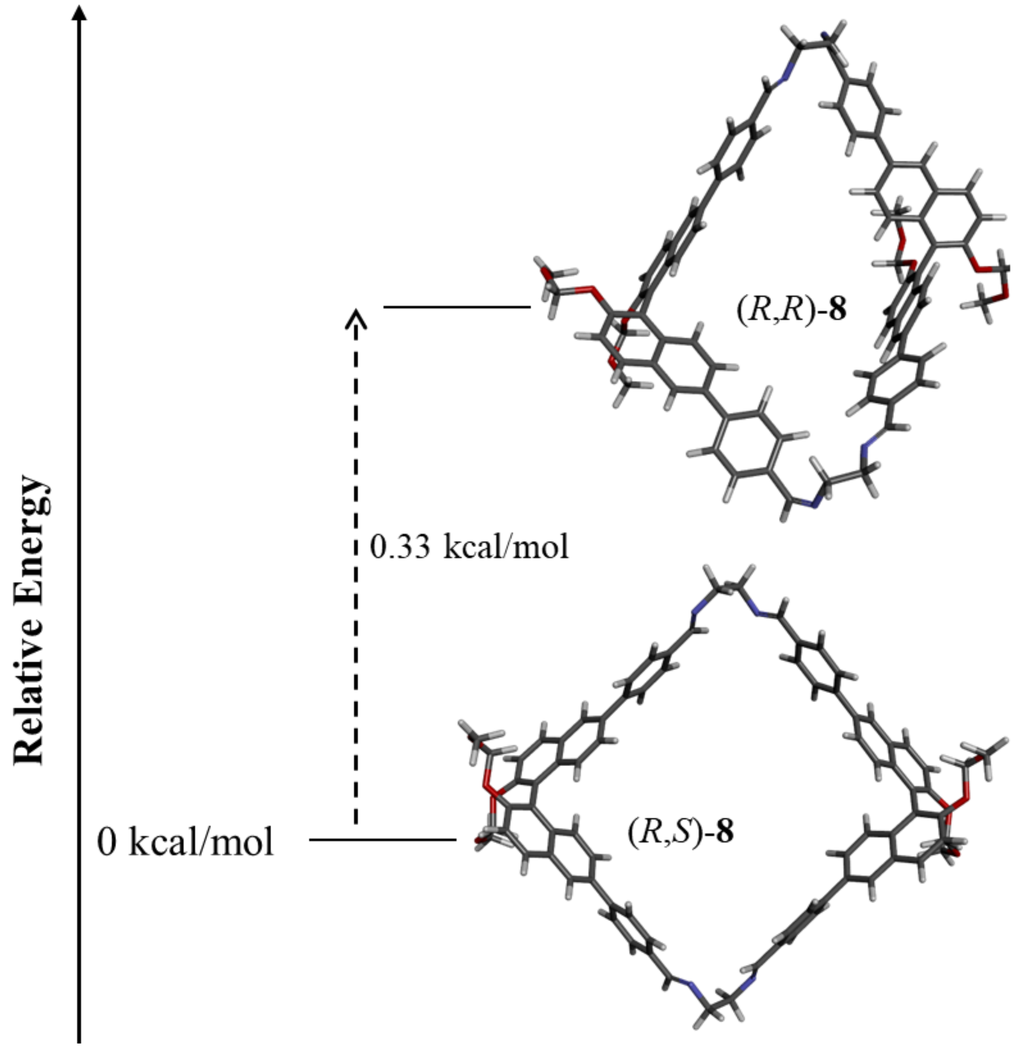

Figure S 59: Relative energies of diastereomeric macrocycles (*R*,*R*)-**8** and (*R*,*S*)-**8** calculated at the B3LYP/6-311G level of theory.

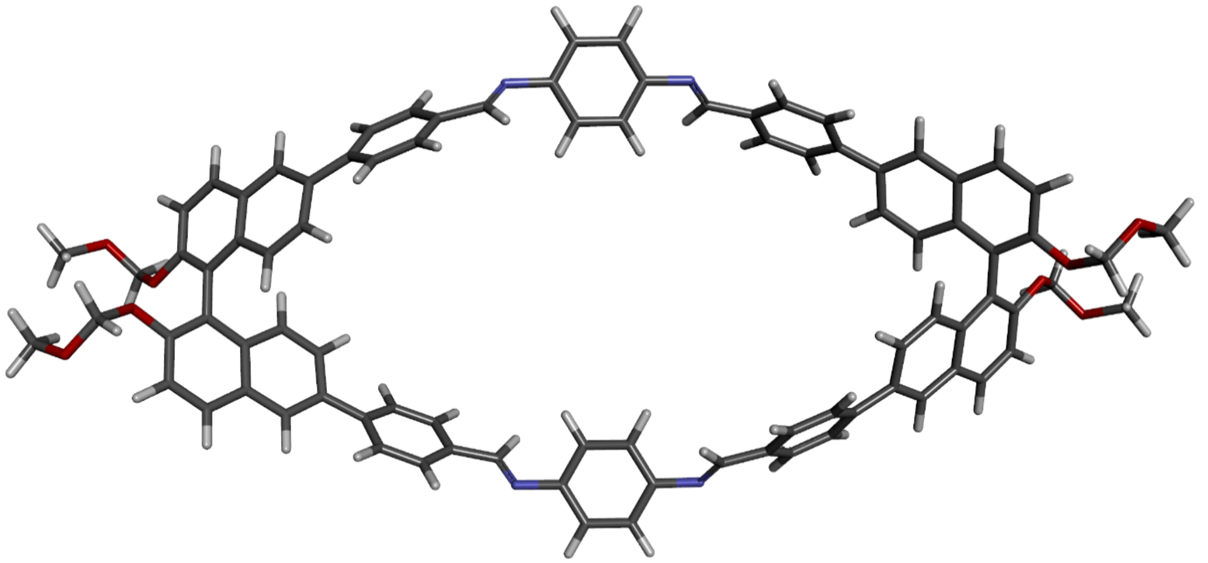

Figure S 60: Geometry optimized structure of macrocycle (*R*,*R*)-**12** at B3LYP/6-311G level of theory.

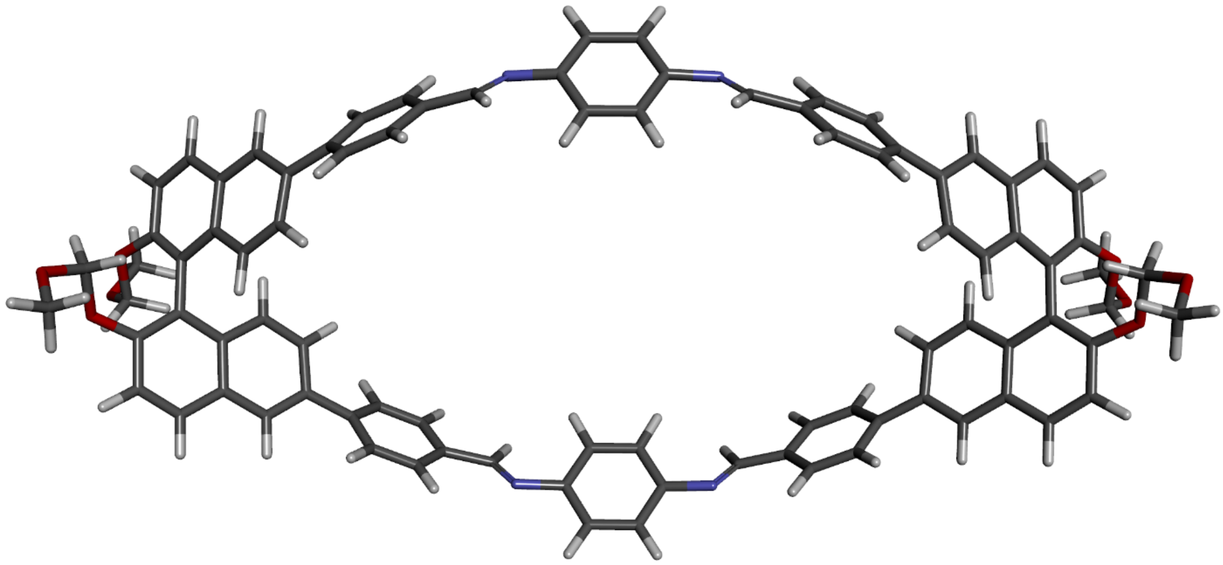

Figure S 61: Geometry optimized structure of macrocycle (*R*,*S*)-**12** at B3LYP/6-311G level of theory.

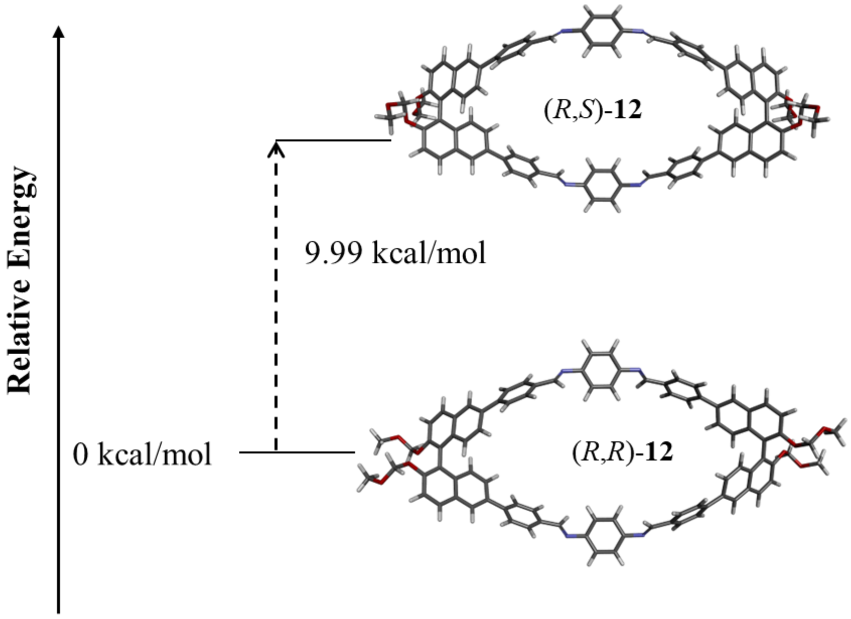

Figure S 62: Relative energies of diastereomeric macrocycles (*R*,*R*)-**12** and (*R*,*S*)-**12** calculated at the B3LYP/6-311G level of theory.

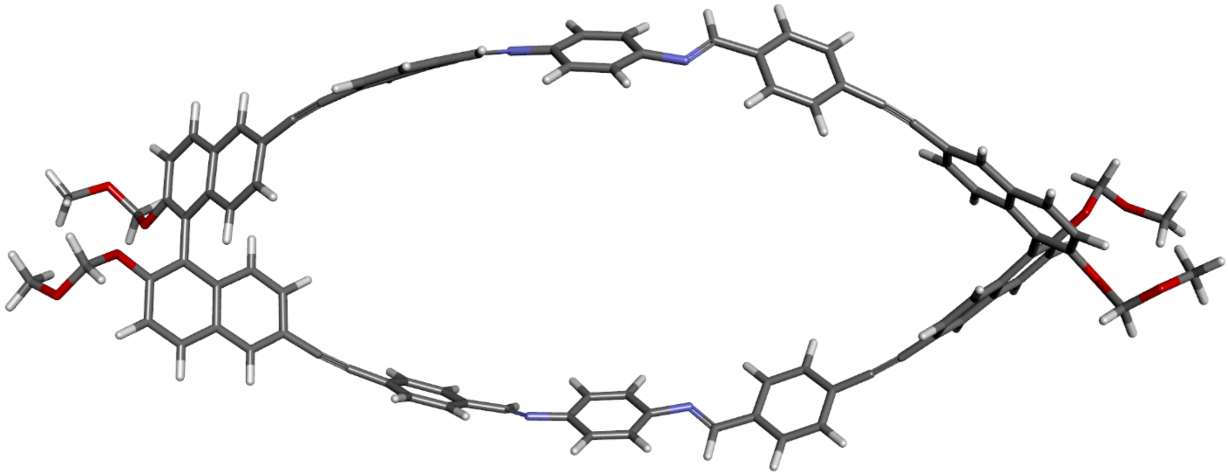

Figure S 63: Geometry optimized structure of macrocycle (*R*,*R*)-**13** at B3LYP/6-311G level of theory.

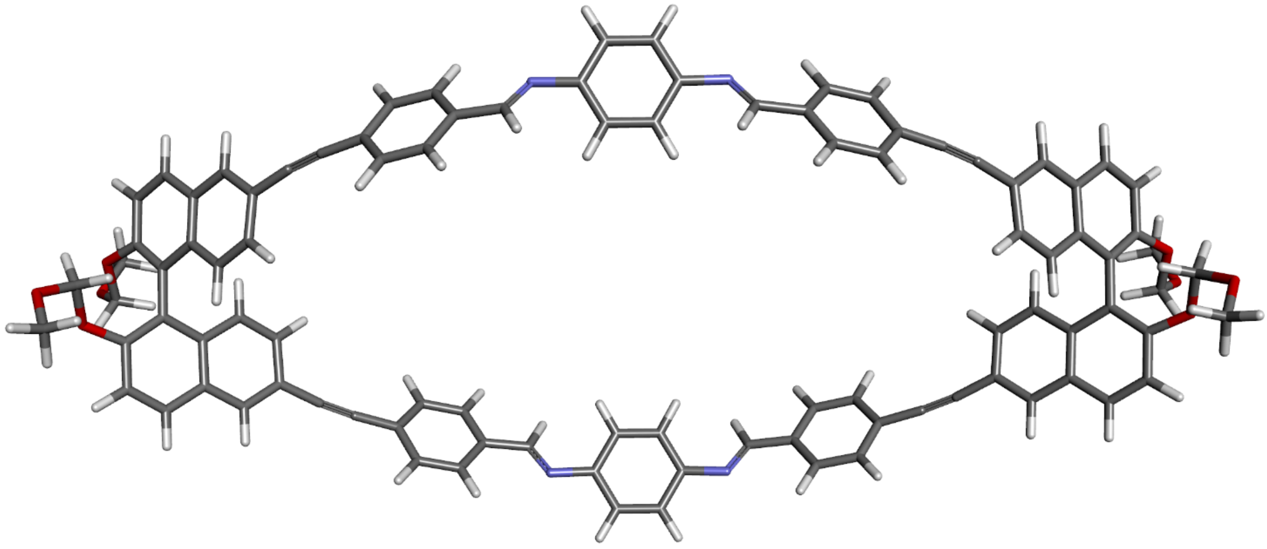

Figure S 64: Geometry optimized structure of macrocycle (*R*,*S*)-**13** at B3LYP/6-311G level of theory.

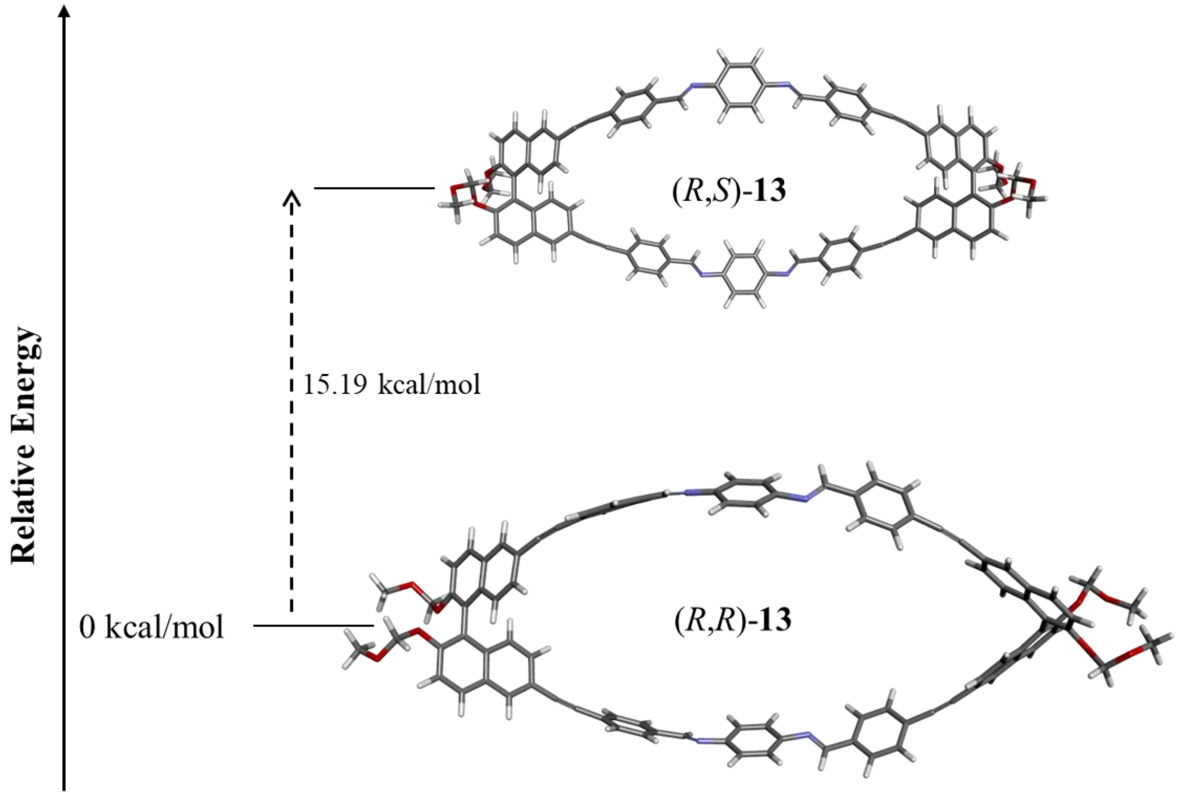

Figure S 65: Relative energies of diastereomeric macrocycles (*R*,*R*)-**13** and (*R*,*S*)-**13** calculated at the B3LYP/6-311G level of theory.

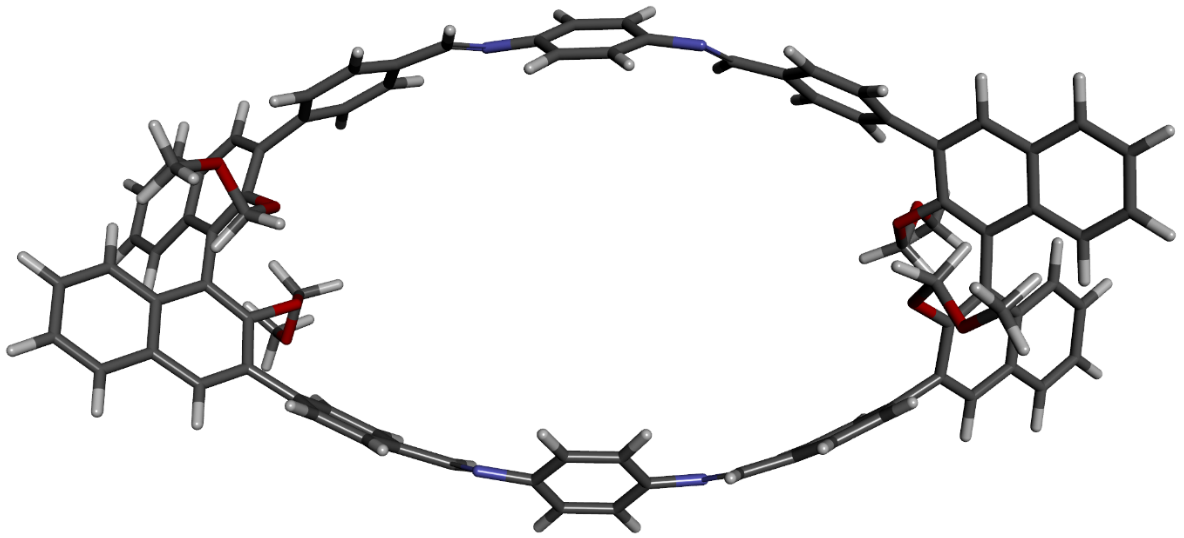

Figure S 66: Geometry optimized structure of macrocycle (*R*,*R*)-**14** at B3LYP/6-311G level of theory.

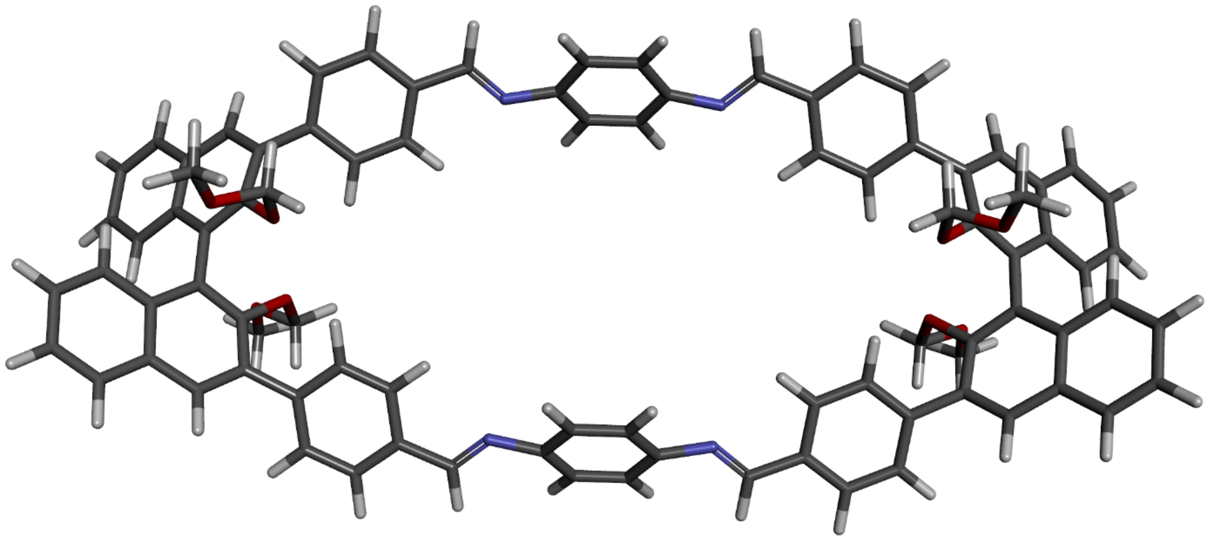

Figure S 67: Geometry optimized structure of macrocycle (*R*,*S*)-**14** at B3LYP/6-311G level of theory.

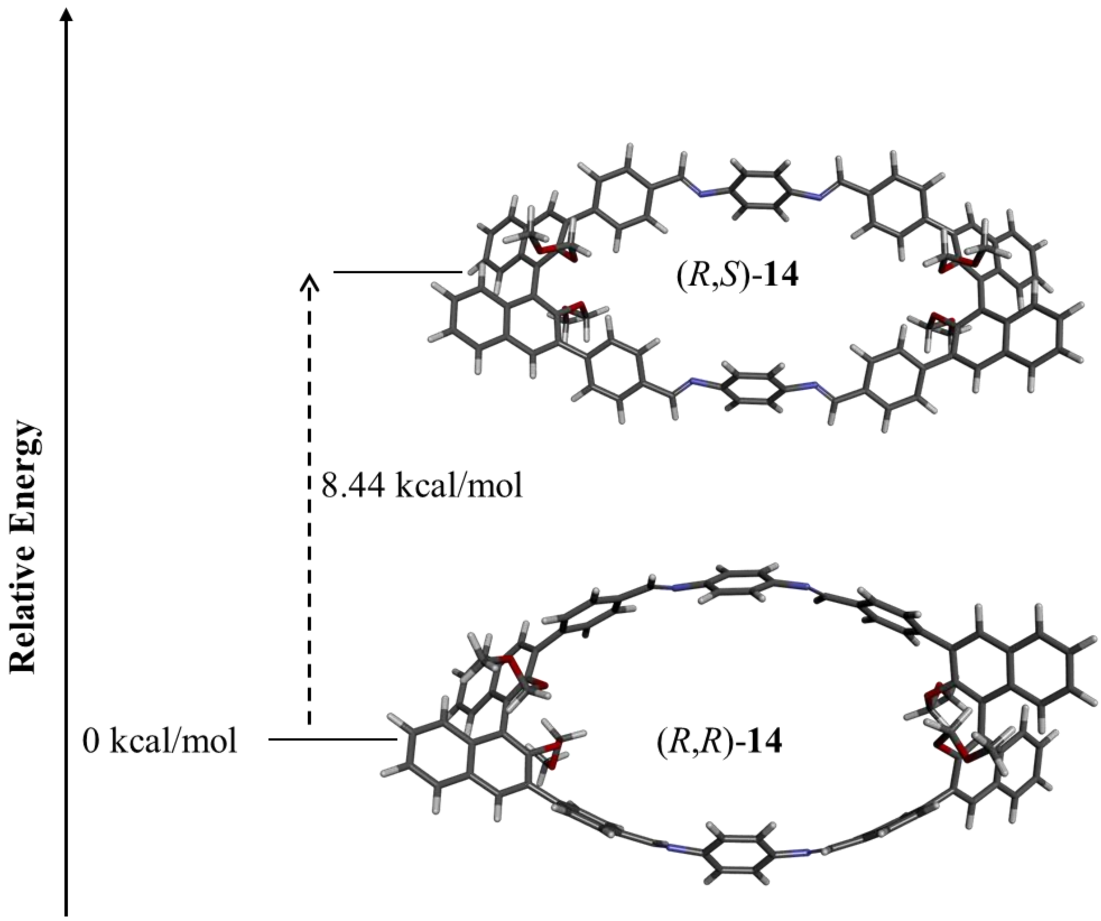

Figure S 68: Relative energies of diastereomeric macrocycles (*R*,*R*)-**14** and (*R*,*S*)-**14** calculated at the B3LYP/6-311G level of theory.

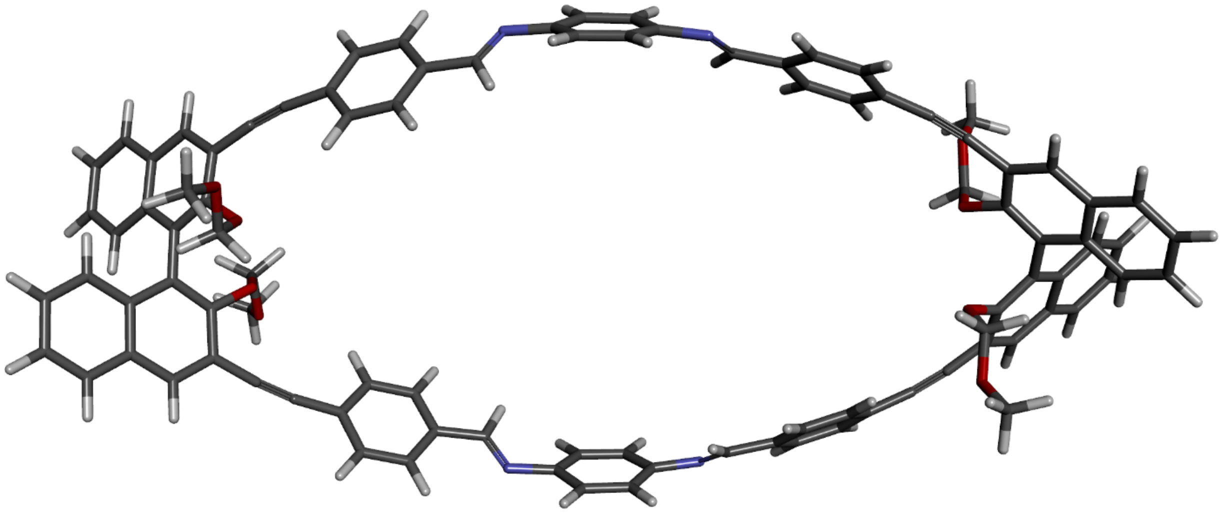

Figure S 69: Geometry optimized structure of macrocycle (*R*,*R*)-**15** at B3LYP/6-311G level of theory.

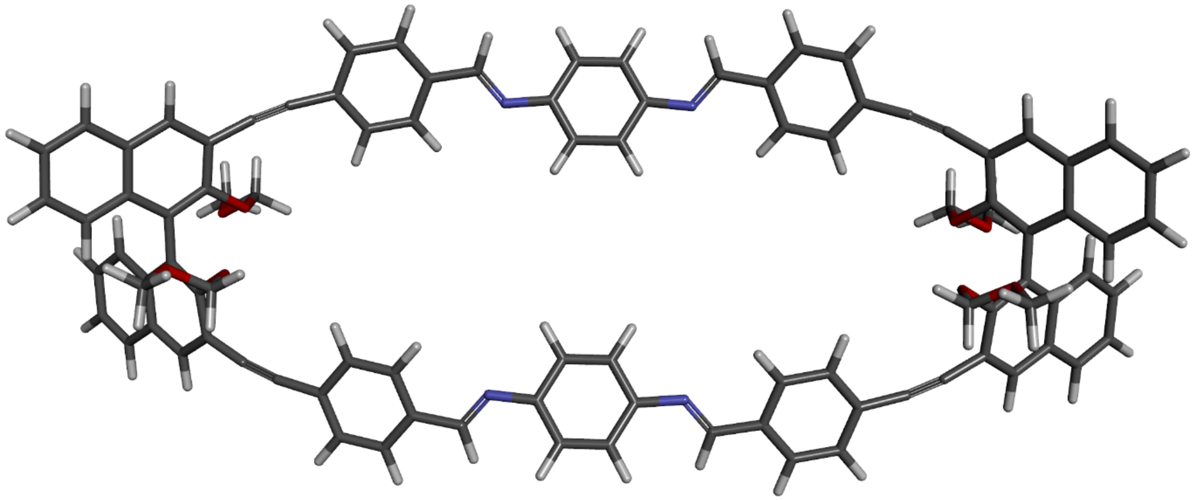

Figure S 70: Geometry optimized structure of macrocycle (*R*,*S*)-**15** at B3LYP/6-311G level of theory.

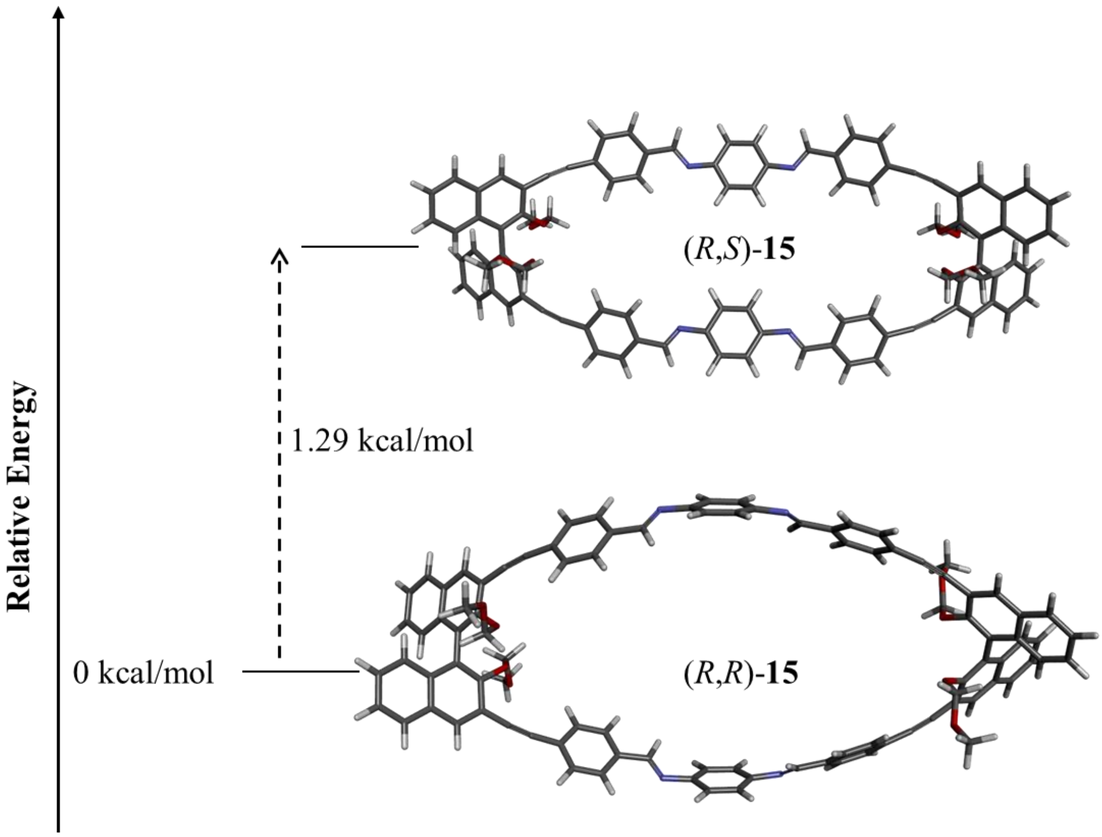

Figure S 71: Relative energies of diastereomeric macrocycles (*R*,*R*)-**15** and (*R*,*S*)-**15** calculated at the B3LYP/6-311G level of theory.

3.10 CD spectra of macrocycles

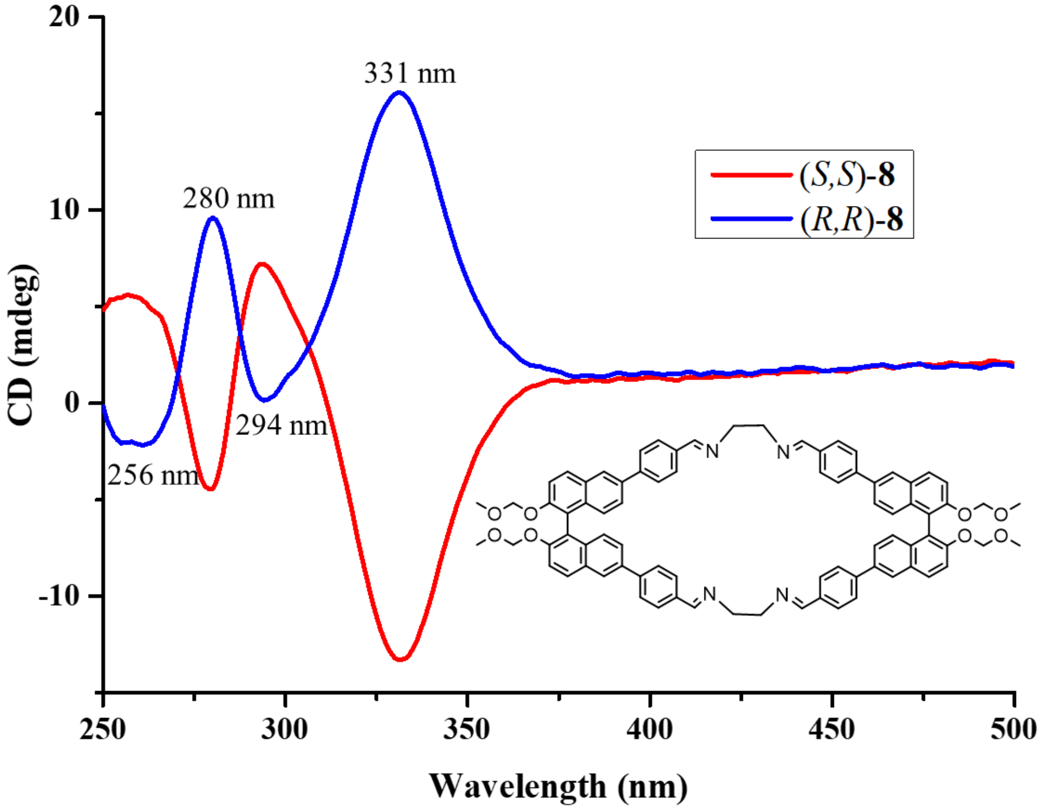

Figure S 72: CD spectra (298 K) of macrocycles (*R*,*R*)-**8** (blue) and (*S*,*S*)-**8** (red) (DCM, 10^{-4} M, 298 K).

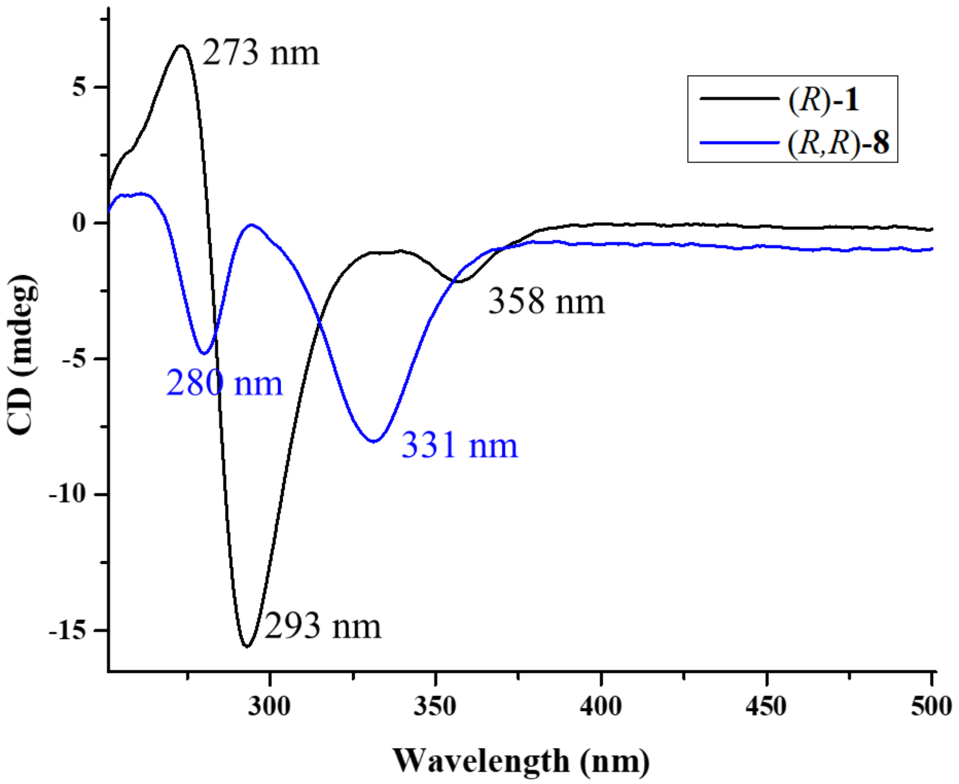

Figure S 73: CD spectra comparison of aldehyde (*R*)-**1** (black) and (*R*,*R*)-**8** (blue) (THF, 5 × 10^{-5} M, 298 K).

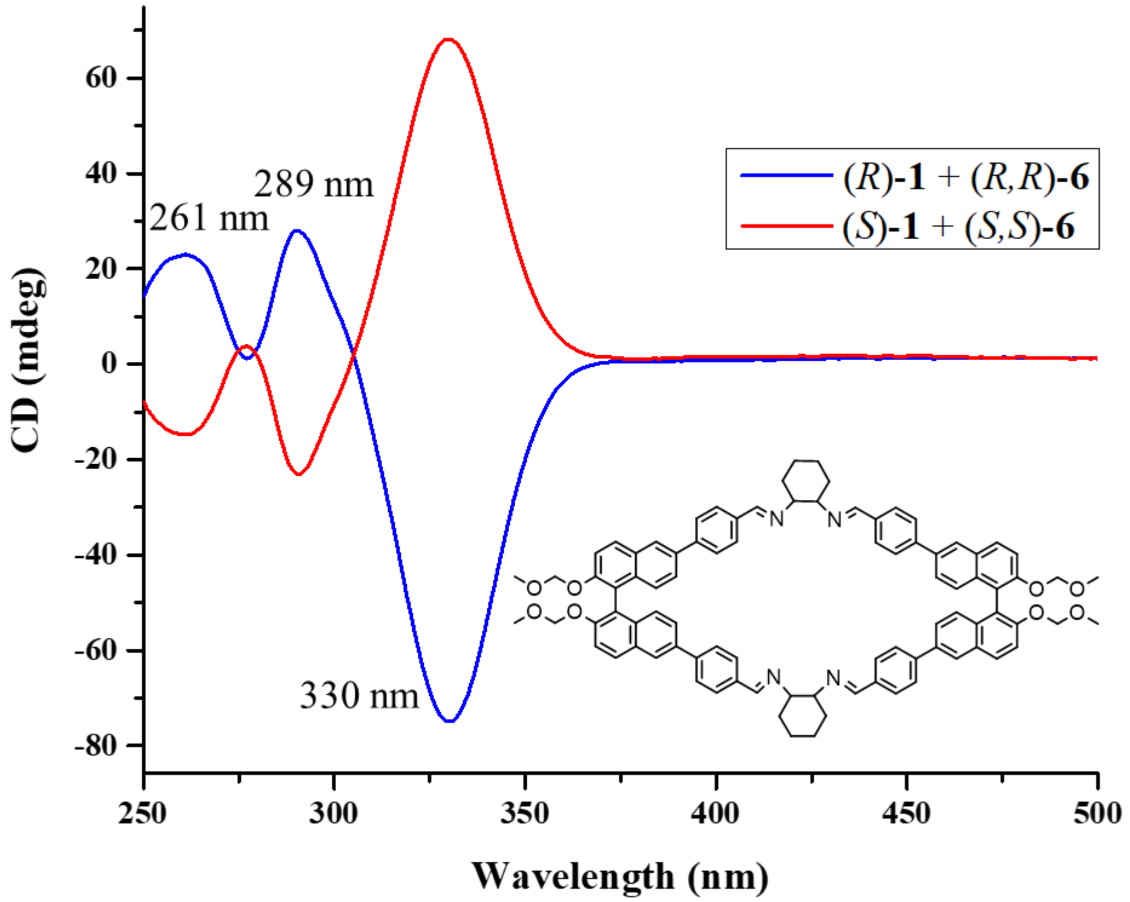

Figure S 74: CD spectra of macrocycles obtained from (*R*)-**1**+(*R*,*R*)-**6** (blue) and (*S*)-**1**+(*S*,*S*)-**6** (red) (DCM, 10^{-4} M, 298 K).

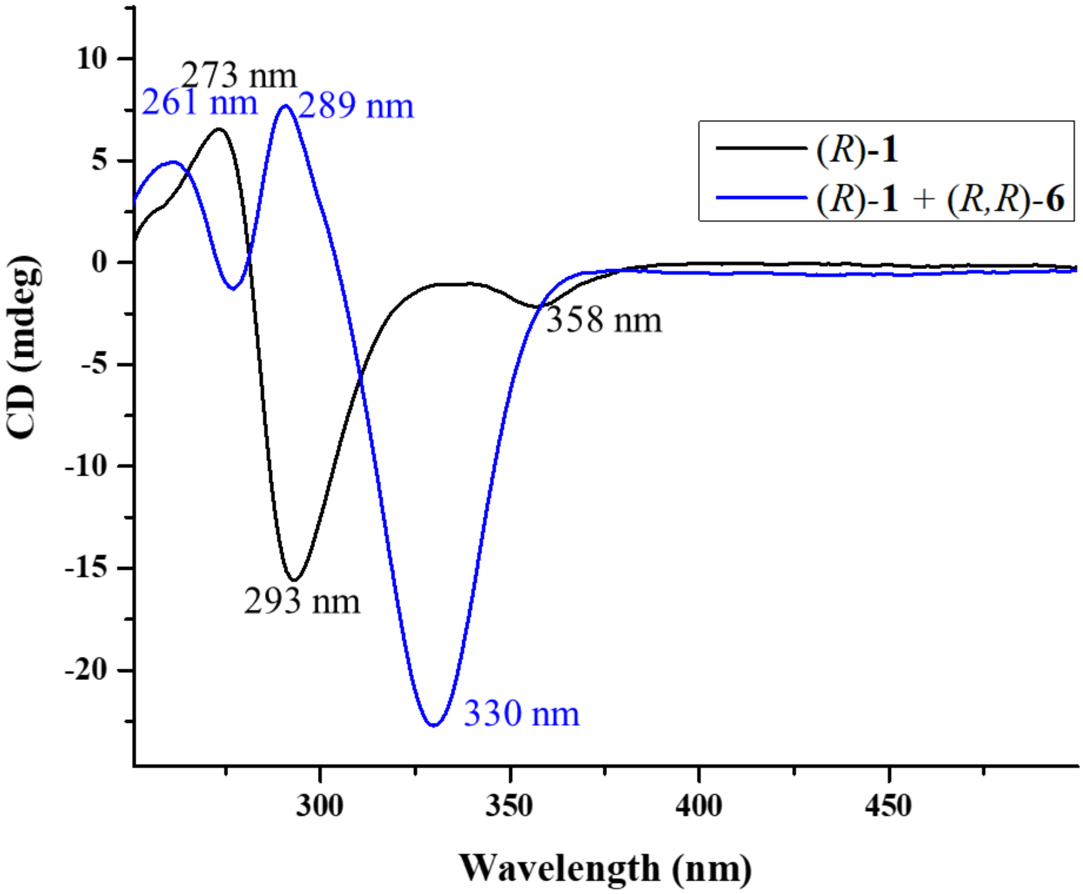

Figure S 75: CD spectra comparison of aldehyde (*R*)-**1** (black) and (*R*)-**1**+(*R*,*R*)-**6** (blue) (THF, 5×10^{-5} M, 298 K).

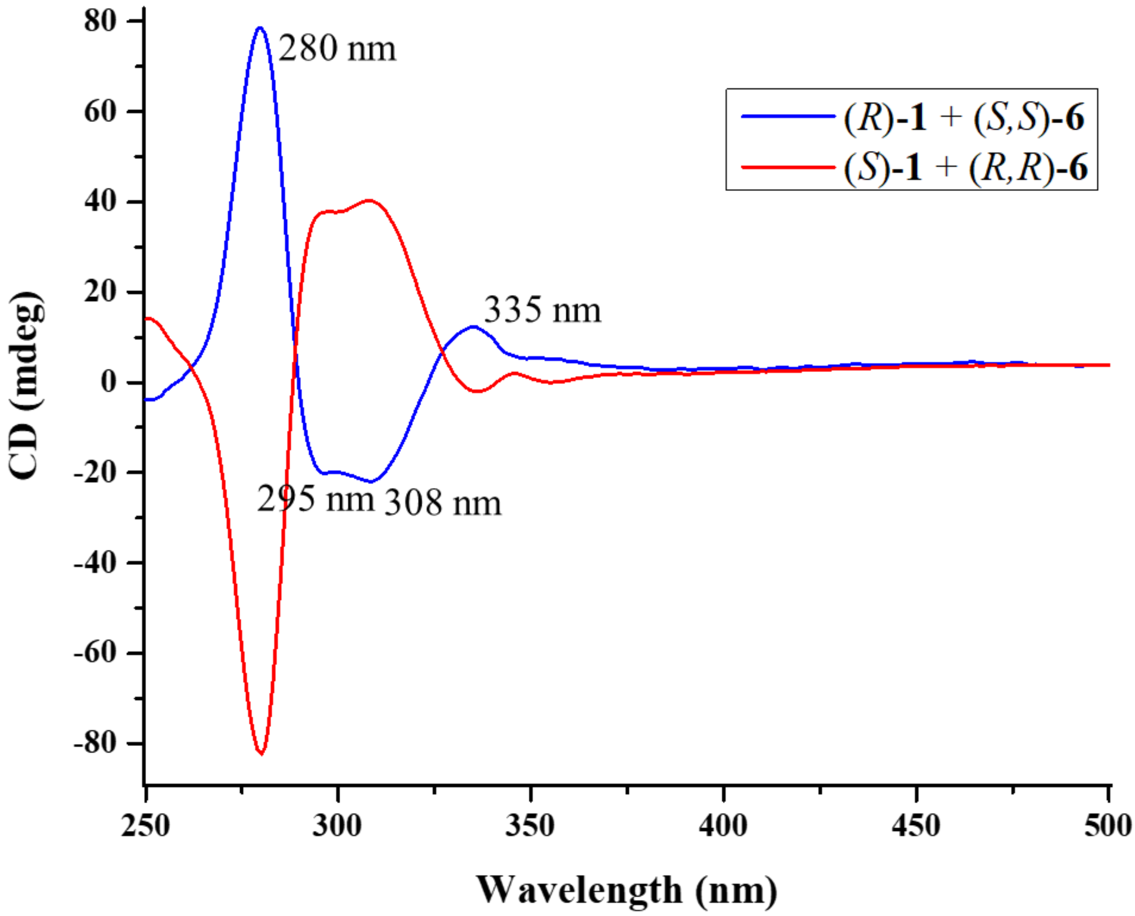

Figure S 76: CD spectra of macrocycles obtained from (*R*)-**1**+(*S*,*S*)-**6** (red) and (*S*)-**1**+(*R*,*R*)-**6** (blue) (DCM, 10^{-4} M, 298 K).

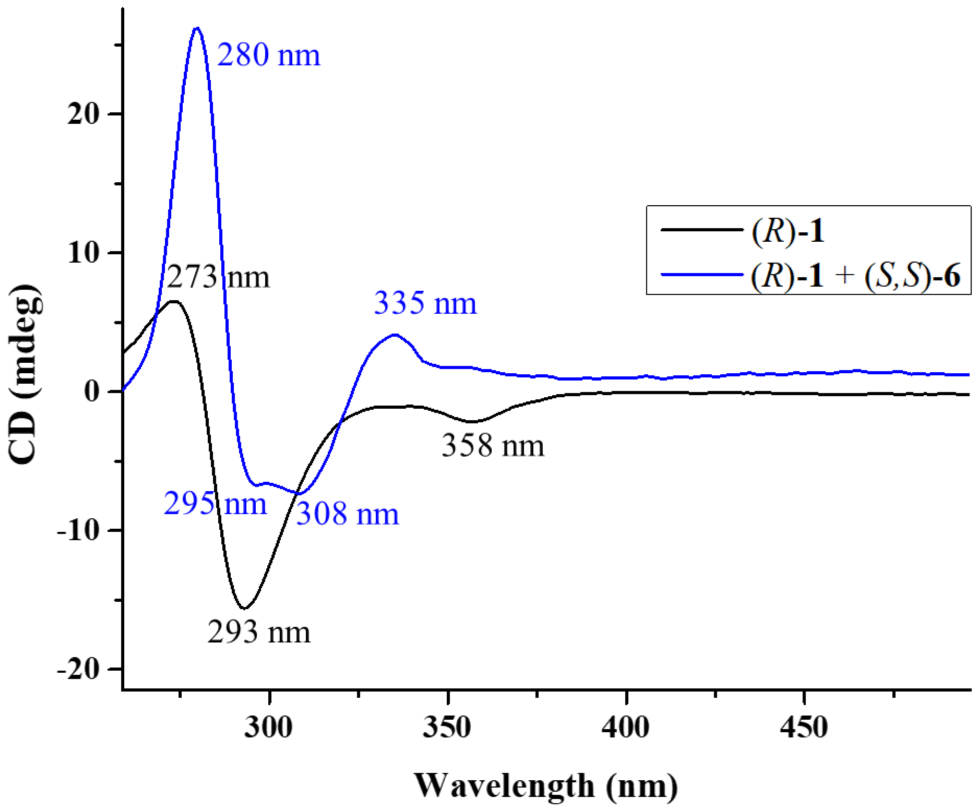

Figure S 77: CD spectra comparison of aldehyde (*R*)-**1** (black) and macrocycle (*R*)-**1**+(*S*,*S*)-**6** (blue) (THF, 5×10^{-5} M, 298 K).

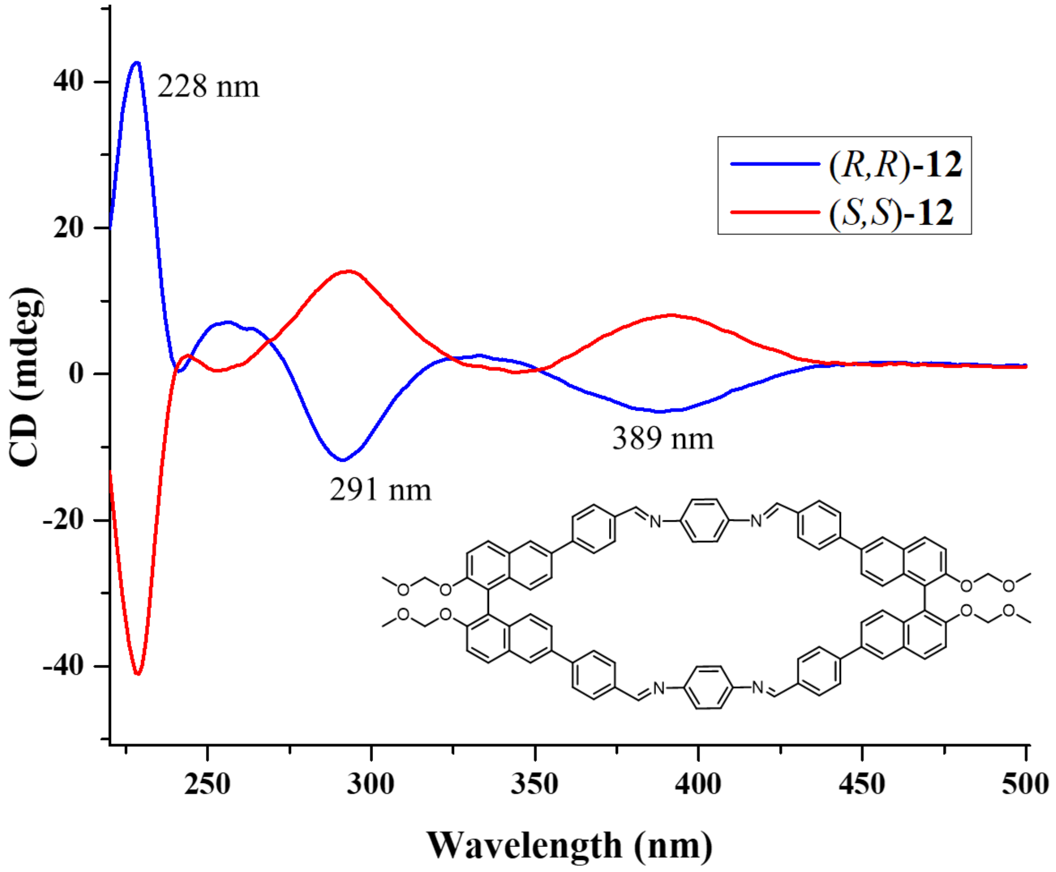

Figure S 78: CD spectra of macrocycles (*R*,*R*)-**12** (blue) and (*S*,*S*)-**12** (red) (THF, 10^{-4} M, 298 K).

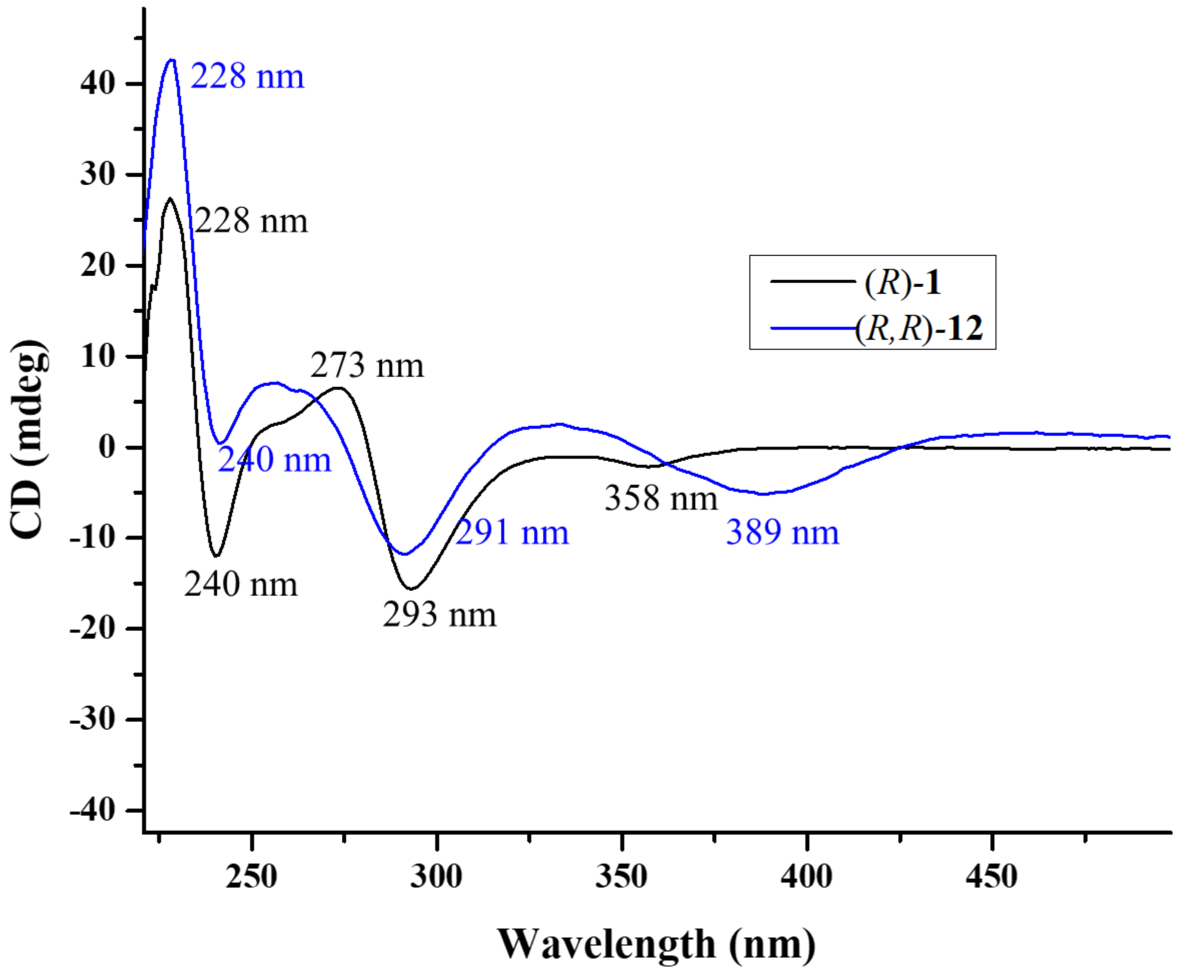

Figure S 79: CD spectra comparison of aldehyde (*R*)-**1** (black) and macrocycle (*R*,*R*)-**12** (blue) (THF, 5×10^{-5} M, 298 K). The emergence of new peat at 389 nm suggests the elongation of conjugation via imine bond formation.

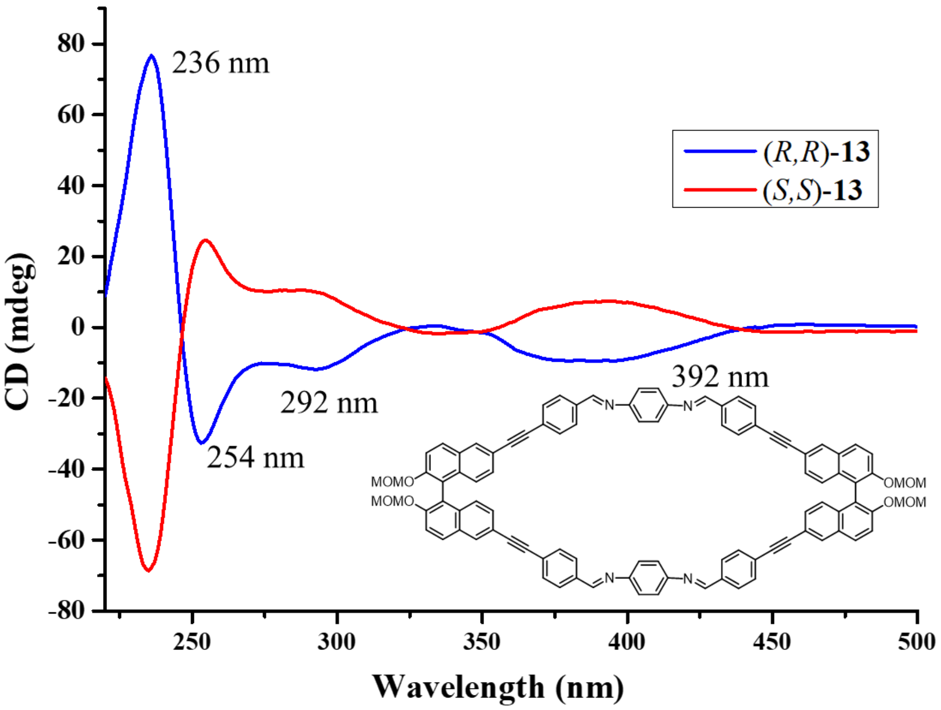

Figure S 80: CD spectra of macrocycles (*R*,*R*)-**13** (blue) and (*S*,*S*)-**13** (red) (DCM, 10^{-4} M, 298 K).

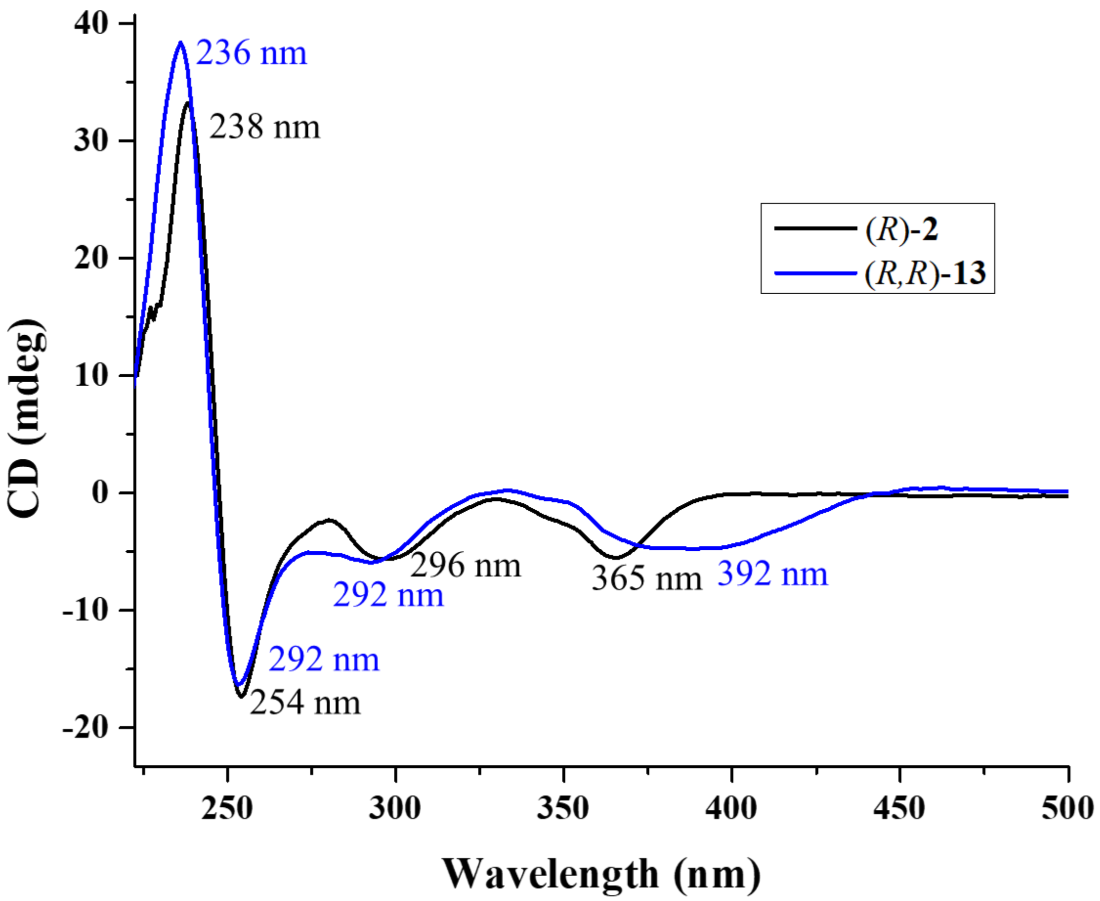

Figure S 81: CD spectra comparison of aldehyde (*R*)-**2** (black) and macrocycle (*R*,*R*)-**13** (blue) (THF, 5×10^{-5} M, 298 K). The emergence of new peat at 392 nm suggests the elongation of conjugation via imine bond formation.

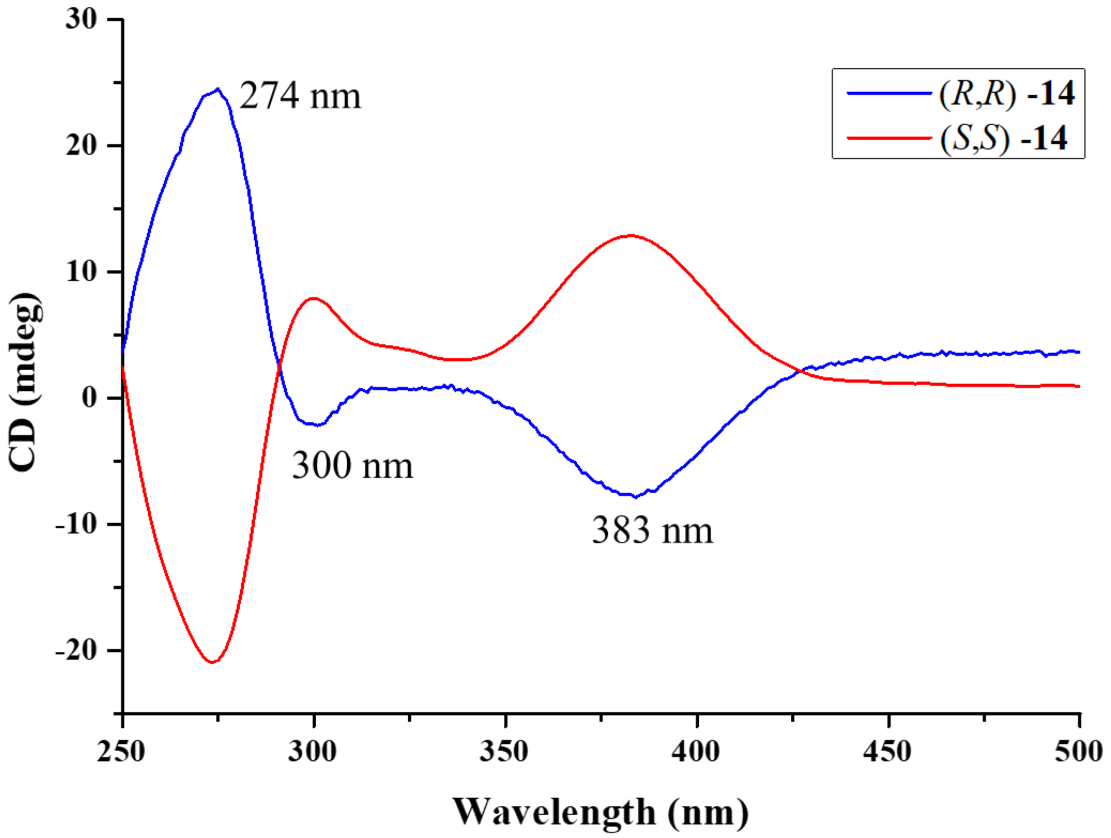

Figure S 82: CD spectra of macrocycles (*R*,*R*)-**14** (blue) and (*S*,*S*)-**14** (red) (DCM, 10^{-4} M, 298 K).

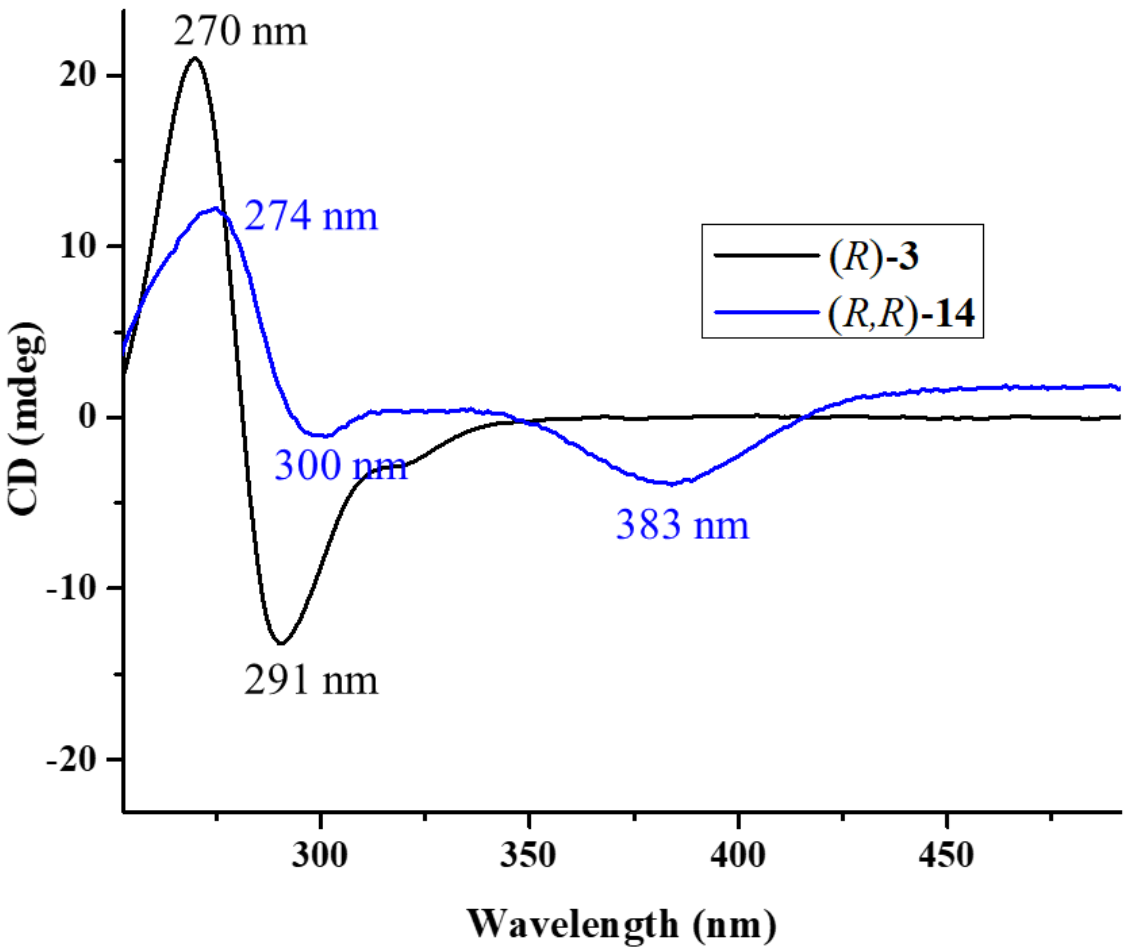

Figure S 83: CD spectra comparison of aldehyde (*R*)-**3** (black) and macrocycle (*R*,*R*)-**14** (blue) (THF, 5×10^{-5} M, 298 K). The emergence of new peat at 383 nm suggests the elongation of conjugation via imine bond formation.

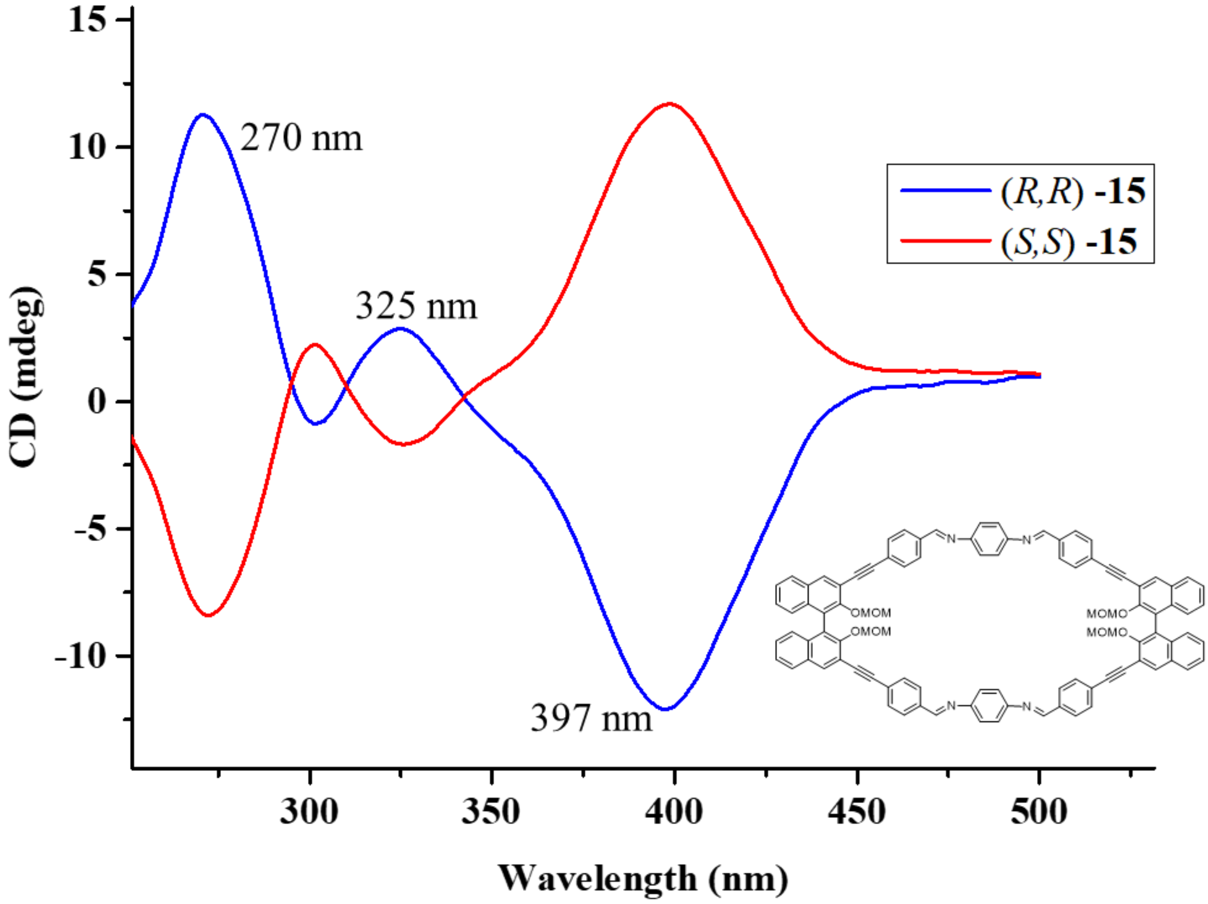

Figure S 84: CD spectra of macrocycles (*R*,*R*)-**15** (blue) and (*S*,*S*)-**15** (red) (THF, 10^{-4} M, 298 K).

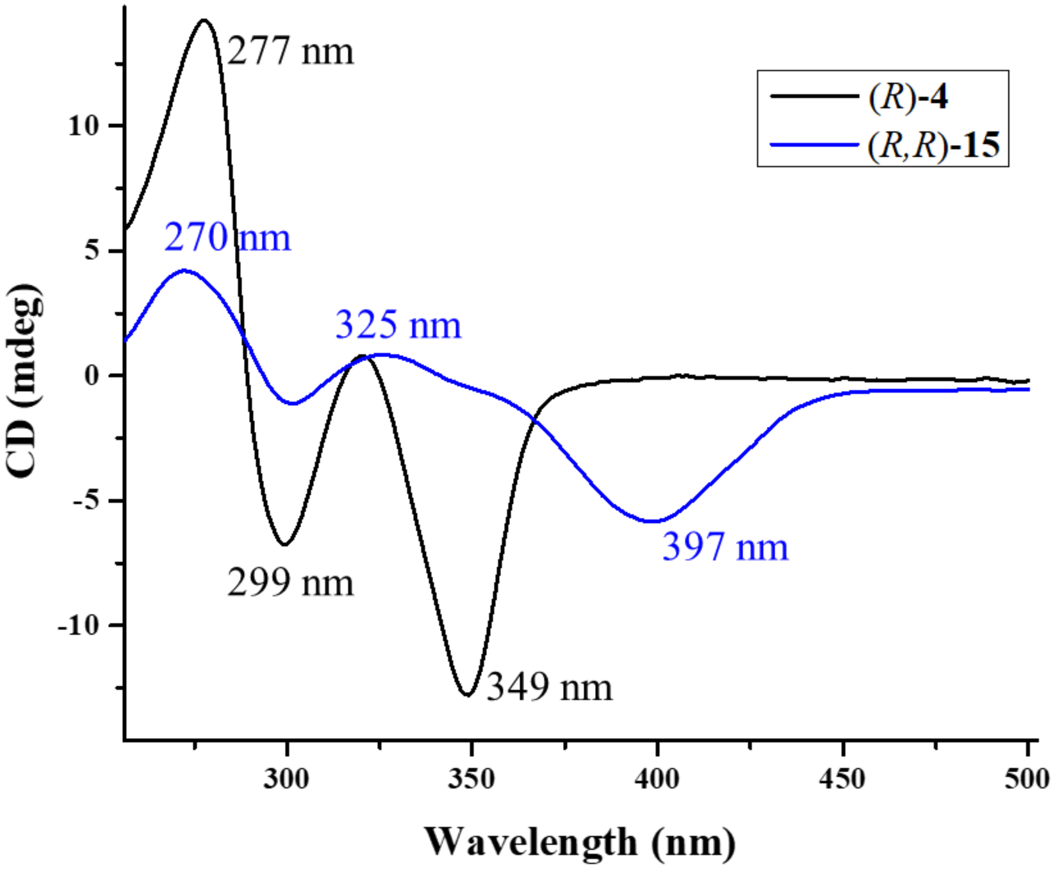

Figure S 85: CD spectra comparison of aldehyde (*R*)-**4** (black) and macrocycle (*R*,*R*)-**15** (blue) (DCM, 5×10^{-5} M, 298 K). The emergence of new peat at 397 nm suggests the elongation of conjugation via imine bond formation

3.11 Specific Rotation of aldehydes and macrocycles:

Table S 1: Specific rotation of aldehydes and macrocycles.

Sample	Concentration	Path length	Wavelength	Optical rotation (deg)	Specific rotation (deg.dm^{-1}.mg^{-1}.mL)
(*R*)-**1**	0.5 mg/mL	20 mm	589 nm	-0.030	-300.0
(*R*)-**12**	0.5 mg/mL	20 mm	589 nm	-0.051	-510.0
(*R*)-**2**	0.5 mg/mL	20 mm	589 nm	-0.027	-270.0
(*R*)-**13**	0.5 mg/mL	20 mm	589 nm	-0.040	-400.0
(*R*)-**3**	0.5 mg/mL	20 mm	589 nm	-0.011	-110.0

(*R*)-**14**	0.5 mg/mL	20 mm	589 nm	-0.020	-200.0
(*R*)-**4**	0.5 mg/mL	20 mm	589 nm	-0.034	-340.0
(*R*)-**15**	0.5 mg/mL	20 mm	589 nm	-0.066	-660.0

3.12 Coordinates for crystal structure:

Table S 2: Crystal data and structure refinement for (±)-**2**.

Identification code	shelx	
Empirical formula	C42 H30 O6	
Formula weight	630.66	
Temperature	273(2) K	
Wavelength	0.71073 Å	
Crystal system	Monoclinic	
Space group	C 2/c	
Unit cell dimensions	a = 19.130(3) Å	α= 90°.
	b = 19.950(3) Å	β= 96.428(5)°.
	c = 9.7527(16) Å	γ = 90°.
Volume	3698.6(10) $Å^3$	
Z	4	
Density (calculated)	1.133 Mg/m^3	
Absorption coefficient	0.075 mm^{-1}	
F(000)	1320	
Crystal size	0.095 x 0.050 x 0.050 mm^3	
Theta range for data collection	2.470 to 24.998°.	
Index ranges	-22<=h<=21, -23<=k<=23, -11<=l<=11	
Reflections collected	21423	
Independent reflections	3267 [R(int) = 0.0515]	
Completeness to theta = 24.998°	99.8 %	
Absorption correction	Semi-empirical from equivalents	
Max. and min. transmission	0.996 and 0.993	
Refinement method	Full-matrix least-squares on F^2	
Data / restraints / parameters	3267 / 0 / 218	
Goodness-of-fit on F^2	1.048	
Final R indices [I>2sigma(I)]	R1 = 0.0451, wR2 = 0.1183	
R indices (all data)	R1 = 0.0663, wR2 = 0.1309	

Extinction coefficient	n/a
Largest diff. peak and hole	0.223 and -0.164 e.Å^{-3}

Table S 3: Atomic coordinates (× 10^4) and equivalent isotropic displacement parameters (Å^2× 10^3) for (±)-**2**. U(eq) is defined as one third of the trace of the orthogonalized U^{ij} tensor.

	x	y	z	U(eq)
C(1)	5996(2)	901(1)	12014(2)	60(1)
C(2)	6039(1)	155(1)	10178(2)	34(1)
C(3)	5857(1)	912(1)	8232(2)	25(1)
C(4)	6580(1)	919(1)	8063(2)	30(1)
C(5)	6810(1)	1276(1)	7015(2)	31(1)
C(6)	6346(1)	1659(1)	6103(2)	26(1)
C(7)	5623(1)	1680(1)	6322(2)	25(1)
C(8)	5381(1)	1289(1)	7393(2)	24(1)
C(9)	5165(1)	2087(1)	5429(2)	31(1)
C(10)	5398(1)	2426(1)	4364(2)	35(1)
C(11)	6112(1)	2380(1)	4099(2)	33(1)
C(12)	6574(1)	2011(1)	4979(2)	31(1)
C(13)	6321(1)	2695(1)	2873(2)	36(1)
C(14)	6448(1)	2936(1)	1806(2)	37(1)
C(15)	6606(1)	3264(1)	567(2)	33(1)
C(16)	6619(1)	3962(1)	535(2)	38(1)
C(17)	6803(1)	4293(1)	-613(2)	40(1)
C(18)	6956(1)	3938(1)	-1757(2)	41(1)
C(19)	6923(1)	3243(1)	-1753(2)	44(1)
C(20)	6752(1)	2908(1)	-594(2)	40(1)
C(21)	7149(1)	4316(1)	-2962(3)	60(1)
O(1)	6423(1)	566(1)	11127(1)	41(1)
O(2)	5577(1)	524(1)	9200(1)	32(1)
O(3)	7250(1)	4082(1)	-4054(2)	84(1)

Table S 4: Bond lengths [Å] and angles [°] for *(±)*-**2**.

C(1)-O(1)	1.422(3)
C(2)-O(1)	1.385(2)
C(2)-O(2)	1.430(2)
C(3)-O(2)	1.375(2)
C(3)-C(8)	1.377(3)
C(3)-C(4)	1.411(2)
C(4)-C(5)	1.359(3)
C(5)-C(6)	1.410(3)
C(6)-C(12)	1.410(2)
C(6)-C(7)	1.425(2)
C(7)-C(9)	1.420(3)
C(7)-C(8)	1.421(2)
C(8)-C(8)#1	1.497(3)
C(9)-C(10)	1.355(3)
C(10)-C(11)	1.423(3)
C(11)-C(12)	1.375(3)
C(11)-C(13)	1.445(3)
C(13)-C(14)	1.196(3)
C(14)-C(15)	1.436(3)
C(15)-C(20)	1.391(3)
C(15)-C(16)	1.393(3)
C(16)-C(17)	1.379(3)
C(17)-C(18)	1.380(3)
C(18)-C(19)	1.388(3)
C(18)-C(21)	1.477(3)
C(19)-C(20)	1.383(3)
C(21)-O(3)	1.198(3)
O(1)-C(2)-O(2)	112.50(16)
O(2)-C(3)-C(8)	115.63(15)
O(2)-C(3)-C(4)	123.21(16)
C(8)-C(3)-C(4)	121.13(16)
C(5)-C(4)-C(3)	119.80(18)
C(4)-C(5)-C(6)	121.65(17)
C(12)-C(6)-C(5)	122.19(16)
C(12)-C(6)-C(7)	119.50(17)
C(5)-C(6)-C(7)	118.30(16)
C(9)-C(7)-C(8)	122.41(16)

C(9)-C(7)-C(6)	117.82(16)
C(8)-C(7)-C(6)	119.76(16)
C(3)-C(8)-C(7)	119.23(15)
C(3)-C(8)-C(8)#1	120.48(16)
C(7)-C(8)-C(8)#1	120.29(16)
C(10)-C(9)-C(7)	121.51(17)
C(9)-C(10)-C(11)	120.86(19)
C(12)-C(11)-C(10)	118.89(17)
C(12)-C(11)-C(13)	122.13(17)
C(10)-C(11)-C(13)	118.90(18)
C(11)-C(12)-C(6)	121.29(17)
C(14)-C(13)-C(11)	175.0(2)
C(13)-C(14)-C(15)	176.5(2)
C(20)-C(15)-C(16)	119.12(18)
C(20)-C(15)-C(14)	122.12(19)
C(16)-C(15)-C(14)	118.76(18)
C(17)-C(16)-C(15)	120.23(19)
C(16)-C(17)-C(18)	120.5(2)
C(17)-C(18)-C(19)	119.79(19)
C(17)-C(18)-C(21)	118.4(2)
C(19)-C(18)-C(21)	121.9(2)
C(20)-C(19)-C(18)	120.0(2)
C(19)-C(20)-C(15)	120.4(2)
O(3)-C(21)-C(18)	126.0(3)
C(2)-O(1)-C(1)	112.83(17)
C(3)-O(2)-C(2)	119.26(14)

Symmetry transformations used to generate equivalent atoms:

#1 -x+1,y,-z+3/2

Table S 5: Anisotropic displacement parameters ($Å^2 \times 10^3$) for (±)-**2**. The anisotropic displacement factor exponent takes the form: $-2\pi^2[h^2 a^{*2} U^{11} + ... + 2 h k a^* b^* U^{12}]$

	U^{11}	U^{22}	U^{33}	U^{23}	U^{13}	U^{12}
C(1)	87(2)	53(2)	41(1)	-4(1)	4(1)	14(1)
C(2)	34(1)	34(1)	33(1)	6(1)	3(1)	2(1)
C(3)	26(1)	27(1)	24(1)	-3(1)	5(1)	-3(1)
C(4)	24(1)	35(1)	30(1)	1(1)	1(1)	2(1)
C(5)	20(1)	38(1)	35(1)	-4(1)	6(1)	-2(1)
C(6)	26(1)	28(1)	27(1)	-5(1)	6(1)	-3(1)
C(7)	24(1)	26(1)	25(1)	-5(1)	5(1)	-2(1)
C(8)	22(1)	26(1)	25(1)	-3(1)	5(1)	0(1)
C(9)	24(1)	34(1)	34(1)	4(1)	7(1)	1(1)
C(10)	32(1)	37(1)	35(1)	8(1)	3(1)	0(1)
C(11)	34(1)	35(1)	32(1)	0(1)	8(1)	-8(1)
C(12)	25(1)	36(1)	31(1)	-3(1)	8(1)	-6(1)
C(13)	33(1)	40(1)	35(1)	3(1)	6(1)	-6(1)
C(14)	29(1)	43(1)	39(1)	4(1)	5(1)	-8(1)
C(15)	23(1)	42(1)	35(1)	7(1)	2(1)	-4(1)
C(16)	35(1)	44(1)	35(1)	0(1)	3(1)	-5(1)
C(17)	38(1)	39(1)	44(1)	8(1)	3(1)	-3(1)
C(18)	35(1)	47(1)	41(1)	14(1)	9(1)	6(1)
C(19)	46(1)	50(1)	38(1)	4(1)	14(1)	8(1)
C(20)	38(1)	38(1)	44(1)	6(1)	8(1)	1(1)
C(21)	60(2)	67(2)	56(2)	24(1)	22(1)	17(1)
O(1)	43(1)	44(1)	35(1)	1(1)	-2(1)	-6(1)
O(2)	25(1)	38(1)	32(1)	13(1)	4(1)	0(1)
O(3)	112(2)	90(2)	57(1)	27(1)	44(1)	32(1)

Table S 6: Hydrogen coordinates (× 10^4) and isotropic displacement parameters (Å^2 × 10^3) for *(±)*-**2**.

	x	y	z	U(eq)
H(1A)	5693	1214	11488	91
H(1B)	6291	1138	12716	91
H(1C)	5716	579	12439	91
H(2A)	5764	-156	10664	40
H(2B)	6360	-106	9689	40
H(4)	6898	680	8667	36
H(5)	7286	1267	6896	37
H(9)	4694	2122	5577	37
H(10)	5086	2691	3801	42
H(12)	7045	1993	4831	37
H(16)	6503	4206	1290	46
H(17)	6825	4759	-617	48
H(19)	7016	3002	-2529	53
H(20)	6733	2442	-591	48
H(21)	7196	4778	-2863	72

Table S 7: Torsion angles [°] for *(±)*-**2**.

O(2)-C(3)-C(4)-C(5)	174.91(17)
C(8)-C(3)-C(4)-C(5)	-3.3(3)
C(3)-C(4)-C(5)-C(6)	1.8(3)
C(4)-C(5)-C(6)-C(12)	-177.49(18)
C(4)-C(5)-C(6)-C(7)	1.5(3)
C(12)-C(6)-C(7)-C(9)	-3.2(3)
C(5)-C(6)-C(7)-C(9)	177.73(17)
C(12)-C(6)-C(7)-C(8)	175.64(16)
C(5)-C(6)-C(7)-C(8)	-3.4(3)
O(2)-C(3)-C(8)-C(7)	-176.95(15)
C(4)-C(3)-C(8)-C(7)	1.4(3)
O(2)-C(3)-C(8)-C(8)#1	3.1(2)
C(4)-C(3)-C(8)-C(8)#1	-178.57(16)

C(9)-C(7)-C(8)-C(3)	-179.20(17)
C(6)-C(7)-C(8)-C(3)	2.0(3)
C(9)-C(7)-C(8)-C(8)#1	0.7(3)
C(6)-C(7)-C(8)-C(8)#1	-178.09(15)
C(8)-C(7)-C(9)-C(10)	-176.22(18)
C(6)-C(7)-C(9)-C(10)	2.6(3)
C(7)-C(9)-C(10)-C(11)	0.5(3)
C(9)-C(10)-C(11)-C(12)	-3.0(3)
C(9)-C(10)-C(11)-C(13)	173.82(19)
C(10)-C(11)-C(12)-C(6)	2.3(3)
C(13)-C(11)-C(12)-C(6)	-174.38(18)
C(5)-C(6)-C(12)-C(11)	179.77(18)
C(7)-C(6)-C(12)-C(11)	0.8(3)
C(20)-C(15)-C(16)-C(17)	-2.7(3)
C(14)-C(15)-C(16)-C(17)	176.74(18)
C(15)-C(16)-C(17)-C(18)	2.0(3)
C(16)-C(17)-C(18)-C(19)	0.1(3)
C(16)-C(17)-C(18)-C(21)	179.9(2)
C(17)-C(18)-C(19)-C(20)	-1.3(3)
C(21)-C(18)-C(19)-C(20)	178.9(2)
C(18)-C(19)-C(20)-C(15)	0.5(3)
C(16)-C(15)-C(20)-C(19)	1.5(3)
C(14)-C(15)-C(20)-C(19)	-177.96(19)
C(17)-C(18)-C(21)-O(3)	-174.9(3)
C(19)-C(18)-C(21)-O(3)	4.9(4)
O(2)-C(2)-O(1)-C(1)	66.5(2)
C(8)-C(3)-O(2)-C(2)	-175.48(16)
C(4)-C(3)-O(2)-C(2)	6.2(2)
O(1)-C(2)-O(2)-C(3)	69.6(2)

Symmetry transformations used to generate equivalent atoms:
#1 -x+1,y,-z+3/2

3.13 Coordinates for DFT optimized structures:

Table S 8: Coordinates of (*R*,*R*)-**8** for the Figure S59.

Standard orientation:

Center Atomic Atomic Coordinates (Angstroms)

Number	Number	Type	X	Y	Z
1	6	0	0.378606	-7.818461	0.274956
2	6	0	-0.300977	-9.036281	0.539634
3	6	0	-1.621807	-9.198841	0.193022
4	6	0	-0.342530	-6.744935	-0.350813
5	6	0	-1.711205	-6.929292	-0.718581
6	6	0	-2.322670	-8.145914	-0.447831
7	6	0	-2.482312	-5.852694	-1.412717
8	6	0	-3.143597	-4.819475	-0.681347
9	6	0	-2.577146	-5.859495	-2.797774
10	6	0	-3.884440	-3.808559	-1.381823
11	6	0	-3.944144	-3.863661	-2.798886
12	6	0	-3.307507	-4.864823	-3.495490
13	6	0	-3.748818	-3.737887	1.419857
14	6	0	-4.502146	-2.734363	0.736714
15	6	0	-4.550922	-2.793136	-0.646896
16	6	0	0.362451	-5.529224	-0.593407
17	6	0	1.685066	-5.387131	-0.243310
18	6	0	2.413918	-6.451388	0.368221
19	6	0	1.745130	-7.638604	0.615848
20	1	0	0.233975	-9.841423	1.027354
21	1	0	-2.128071	-10.124849	0.423815
22	1	0	-4.507621	-3.107962	-3.331466
23	1	0	-3.382476	-4.900262	-4.572729
24	1	0	-3.670705	-3.695214	2.497479
25	1	0	-5.137088	-2.069825	-1.199342
26	1	0	-0.159807	-4.710429	-1.067370
27	1	0	2.196573	-4.458371	-0.458443
28	1	0	2.262009	-8.455127	1.104803
29	6	0	-3.100921	-4.741143	0.740713
30	1	0	-2.541185	-5.486785	1.286530
31	8	0	-1.970843	-6.927121	-3.465620
32	8	0	-3.665918	-8.267910	-0.796911
33	6	0	-0.378606	7.818461	0.274956
34	6	0	0.300977	9.036281	0.539634
35	6	0	1.621807	9.198841	0.193022
36	6	0	0.342530	6.744935	-0.350813
37	6	0	1.711205	6.929292	-0.718581
38	6	0	2.322670	8.145914	-0.447831
39	6	0	2.482312	5.852694	-1.412717
40	6	0	3.143597	4.819475	-0.681347
41	6	0	2.577146	5.859495	-2.797774
42	6	0	3.884440	3.808559	-1.381823
43	6	0	3.944144	3.863661	-2.798886
44	6	0	3.307507	4.864823	-3.495490
45	6	0	3.748818	3.737887	1.419857
46	6	0	4.502146	2.734363	0.736714
47	6	0	4.550922	2.793136	-0.646896
48	6	0	-0.362451	5.529224	-0.593407

49	6	0	-1.685066	5.387131	-0.243310
50	6	0	-2.413918	6.451388	0.368221
51	6	0	-1.745130	7.638604	0.615848
52	1	0	-0.233975	9.841423	1.027354
53	1	0	2.128071	10.124849	0.423815
54	1	0	4.507621	3.107962	-3.331466
55	1	0	3.382476	4.900262	-4.572729
56	1	0	3.670705	3.695214	2.497479
57	1	0	5.137088	2.069825	-1.199342
58	1	0	0.159807	4.710429	-1.067370
59	1	0	-2.196573	4.458371	-0.458443
60	1	0	-2.262009	8.455127	1.104803
61	6	0	3.100921	4.741143	0.740713
62	1	0	2.541185	5.486785	1.286530
63	8	0	1.970843	6.927121	-3.465620
64	8	0	3.665918	8.267910	-0.796911
65	6	0	-3.848478	6.290719	0.717438
66	6	0	-4.338935	5.097739	1.279572
67	6	0	-4.765113	7.332948	0.483193
68	6	0	-5.684498	4.955354	1.618961
69	1	0	-3.657098	4.279384	1.470368
70	6	0	-6.110852	7.187918	0.805634
71	1	0	-4.422815	8.246234	0.015031
72	6	0	-6.591016	6.007098	1.398499
73	1	0	-6.046267	4.023547	2.032590
74	1	0	-6.801493	7.995583	0.595177
75	6	0	3.848478	-6.290719	0.717438
76	6	0	4.338935	-5.097739	1.279572
77	6	0	4.765113	-7.332948	0.483193
78	6	0	5.684498	-4.955354	1.618961
79	1	0	3.657098	-4.279384	1.470368
80	6	0	6.110852	-7.187918	0.805634
81	1	0	4.422815	-8.246234	0.015031
82	6	0	6.591016	-6.007098	1.398499
83	1	0	6.046267	-4.023547	2.032590
84	1	0	6.801493	-7.995583	0.595177
85	6	0	-5.220565	-1.679155	1.493865
86	6	0	-5.755512	-1.938655	2.769232
87	6	0	-5.404937	-0.386583	0.956900
88	6	0	-6.441209	-0.952516	3.473471
89	1	0	-5.659412	-2.925320	3.200384
90	6	0	-6.101485	0.593594	1.652149
91	1	0	-4.979695	-0.147321	-0.008238
92	6	0	-6.629591	0.327638	2.928226
93	1	0	-6.848641	-1.180111	4.451282
94	1	0	-6.245638	1.575444	1.224625
95	6	0	5.220565	1.679155	1.493865
96	6	0	5.755512	1.938655	2.769232
97	6	0	5.404937	0.386583	0.956900
98	6	0	6.441209	0.952516	3.473471

99	1	0	5.659412	2.925320	3.200384
100	6	0	6.101485	-0.593594	1.652149
101	1	0	4.979695	0.147321	-0.008238
102	6	0	6.629591	-0.327638	2.928226
103	1	0	6.848641	1.180111	4.451282
104	1	0	6.245638	-1.575444	1.224625
105	6	0	8.043692	-5.908439	1.696300
106	1	0	8.686352	-6.335250	0.925040
107	6	0	-8.043692	5.908439	1.696300
108	1	0	-8.686352	6.335250	0.925040
109	6	0	7.371596	-1.335101	3.694360
110	1	0	7.715011	-1.007700	4.685131
111	6	0	-7.371596	1.335101	3.694360
112	1	0	-7.715011	1.007700	4.685131
113	7	0	-7.625219	2.519148	3.265713
114	7	0	7.625219	-2.519148	3.265713
115	7	0	8.670154	-5.436770	2.713319
116	7	0	-8.670154	5.436770	2.713319
117	6	0	-8.020051	4.892811	3.916449
118	6	0	8.020051	-4.892811	3.916449
119	6	0	-8.416280	3.424786	4.114435
120	6	0	8.416280	-3.424786	4.114435
121	6	0	-4.230217	-9.598435	-1.051896
122	1	0	-3.473512	-10.195792	-1.574789
123	1	0	-4.533352	-10.061101	-0.115595
124	6	0	4.230217	9.598435	-1.051896
125	1	0	3.473512	10.195792	-1.574789
126	1	0	4.533352	10.061101	-0.115595
127	6	0	-1.589010	-6.782019	-4.873050
128	1	0	-2.462173	-6.870336	-5.515616
129	1	0	-1.095591	-5.809612	-4.996800
130	6	0	1.589010	6.782019	-4.873050
131	1	0	2.462173	6.870336	-5.515616
132	1	0	1.095591	5.809612	-4.996800
133	6	0	-5.211513	-8.924136	-3.158406
134	1	0	-4.887160	-7.886911	-3.118117
135	1	0	-4.472936	-9.520068	-3.702859
136	1	0	-6.180731	-9.001654	-3.640434
137	6	0	5.211513	8.924136	-3.158406
138	1	0	4.887160	7.886911	-3.118117
139	1	0	4.472936	9.520068	-3.702859
140	1	0	6.180731	9.001654	-3.640434
141	6	0	0.543305	-7.877190	-4.529277
142	1	0	1.079885	-6.937113	-4.690297
143	1	0	0.408403	-8.047080	-3.463357
144	1	0	1.095230	-8.696937	-4.977198
145	6	0	-0.543305	7.877190	-4.529277
146	1	0	-1.079885	6.937113	-4.690297
147	1	0	-0.408403	8.047080	-3.463357
148	1	0	-1.095230	8.696937	-4.977198

149	8	0	-0.747943	-7.854658	-5.216134
150	8	0	0.747943	7.854658	-5.216134
151	8	0	-5.404937	-9.452882	-1.808925
152	8	0	5.404937	9.452882	-1.808925
153	1	0	9.468385	-3.334074	3.830153
154	1	0	-9.468385	3.334074	3.830153
155	1	0	8.328702	-3.153400	5.175623
156	1	0	-8.328702	3.153400	5.175623
157	1	0	8.433033	-5.459429	4.755109
158	1	0	-6.931853	4.997298	3.942775
159	1	0	6.931853	-4.997298	3.942775
160	1	0	-8.433033	5.459429	4.755109

Table S 9: Coordinates of (*R*,*S*)-**8** for the Figure S60.

Standard orientation:

Center Number	Atomic Number	Atomic Type	Coordinates (Angstroms) X	Y	Z
1	6	0	2.828867	1.464512	7.714605
2	6	0	2.765939	2.532793	8.654182
3	6	0	1.762932	2.584011	9.585070
4	6	0	1.822010	0.444521	7.763006
5	6	0	0.755307	0.519740	8.730994
6	6	0	0.749471	1.587924	9.620988
7	6	0	-0.300986	-0.543904	8.748962
8	6	0	-1.316162	-0.549316	7.724074
9	6	0	-0.353956	-1.524811	9.732274
10	6	0	-2.340391	-1.553084	7.725085
11	6	0	-2.339629	-2.528915	8.762480
12	6	0	-1.380142	-2.507922	9.739731
13	6	0	-2.391541	0.467457	5.781099
14	6	0	-3.403914	-0.534062	5.764623
15	6	0	-3.351604	-1.527889	6.728851
16	6	0	1.952836	-0.651737	6.863054
17	6	0	3.006435	-0.733154	5.980208
18	6	0	3.995172	0.288601	5.908043
19	6	0	3.881739	1.369241	6.767303
20	1	0	3.528883	3.300455	8.629790
21	1	0	1.706201	3.375201	10.318365
22	1	0	-3.115699	-3.283621	8.774906
23	1	0	-1.371601	-3.228106	10.544880
24	1	0	-2.408470	1.240129	5.024149
25	1	0	-4.124541	-2.285920	6.761595
26	1	0	1.208168	-1.433765	6.877386
27	1	0	3.075431	-1.576127	5.305473
28	1	0	4.635243	2.147248	6.754839

29	6	0	-1.381668	0.455107	6.716444
30	1	0	-0.622093	1.222169	6.689434
31	8	0	0.491828	-1.597659	10.839224
32	8	0	-0.149493	1.754324	10.673402
33	6	0	-2.828867	-1.464512	-7.714605
34	6	0	-2.765939	-2.532793	-8.654182
35	6	0	-1.762932	-2.584011	-9.585070
36	6	0	-1.822010	-0.444521	-7.763006
37	6	0	-0.749471	-1.587924	-9.620988
38	6	0	1.316162	0.549316	-7.724074
39	6	0	0.353956	1.524811	-9.732274
40	6	0	2.340391	1.553084	-7.725085
41	6	0	2.339629	2.528915	-8.762480
42	6	0	1.380142	2.507922	-9.739731
43	6	0	2.391541	-0.467457	-5.781099
44	6	0	3.403914	0.534062	-5.764623
45	6	0	3.351604	1.527889	-6.728851
46	6	0	-1.952836	0.651737	-6.863054
47	6	0	-3.006435	0.733154	-5.980208
48	6	0	-3.995172	-0.288601	-5.908043
49	6	0	-3.881739	-1.369241	-6.767303
50	1	0	-3.528883	-3.300455	-8.629790
51	1	0	-1.706201	-3.375201	-10.318365
52	1	0	3.115699	3.283621	-8.774906
53	1	0	1.371601	3.228106	-10.544880
54	1	0	2.408470	-1.240129	-5.024149
55	1	0	4.124541	2.285920	-6.761595
56	1	0	-1.208168	1.433765	-6.877386
57	1	0	-3.075431	1.576127	-5.305473
58	1	0	-4.635243	-2.147248	-6.754839
59	6	0	1.381668	-0.455107	-6.716444
60	1	0	0.622093	-1.222169	-6.689434
61	8	0	-0.491828	1.597659	-10.839224
62	8	0	0.149493	-1.754324	-10.673402
63	6	0	0.300986	0.543904	-8.748962
64	6	0	-0.755307	-0.519740	-8.730994
65	6	0	4.490699	0.477174	-4.753457
66	6	0	5.163604	-0.736416	-4.503514
67	6	0	4.876362	1.612346	-4.020848
68	6	0	6.186030	-0.810177	-3.566974
69	1	0	4.892351	-1.617726	-5.069728
70	6	0	5.890005	1.533852	-3.065179
71	1	0	4.358542	2.549052	-4.179782
72	6	0	6.561852	0.324824	-2.827637
73	1	0	6.713658	-1.735522	-3.385955
74	1	0	6.159322	2.415012	-2.495203
75	6	0	-4.490699	-0.477174	4.753457
76	6	0	-5.163604	0.736416	4.503514
77	6	0	-4.876362	-1.612346	4.020848
78	6	0	-6.186030	0.810177	3.566974

79	1	0	-4.892351	1.617726	5.069728
80	6	0	-5.890005	-1.533852	3.065179
81	1	0	-4.358542	-2.549052	4.179782
82	6	0	-6.561852	-0.324824	2.827637
83	1	0	-6.713658	1.735522	3.385955
84	1	0	-6.159322	-2.415012	2.495203
85	6	0	5.123545	0.169546	4.947989
86	6	0	5.451388	1.219510	4.067680
87	6	0	5.894429	-1.004848	4.899635
88	6	0	6.499593	1.094915	3.161700
89	1	0	4.858335	2.124408	4.082332
90	6	0	6.961955	-1.119542	4.012865
91	1	0	5.669999	-1.816341	5.578981
92	6	0	7.272932	-0.078255	3.123284
93	1	0	6.736424	1.892260	2.471769
94	1	0	7.556076	-2.025516	4.002919
95	6	0	-5.123545	-0.169546	-4.947989
96	6	0	-5.451388	-1.219510	-4.067680
97	6	0	-5.894429	1.004848	-4.899635
98	6	0	-6.499593	-1.094915	-3.161700
99	1	0	-4.858335	-2.124408	-4.082332
100	6	0	-6.961955	1.119542	-4.012865
101	1	0	-5.669999	1.816341	-5.578981
102	6	0	-7.272932	0.078255	-3.123284
103	1	0	-6.736424	-1.892260	-2.471769
104	1	0	-7.556076	2.025516	-4.002919
105	6	0	-8.383384	0.244922	-2.177491
106	1	0	-9.050288	1.093222	-2.385528
107	6	0	-7.632289	-0.252186	1.818422
108	1	0	-7.743978	-1.125999	1.171837
109	6	0	7.632289	0.252186	-1.818422
110	1	0	7.743978	1.125999	-1.171837
111	6	0	8.383384	-0.244922	2.177491
112	1	0	9.050288	-1.093222	2.385528
113	7	0	-8.367404	0.793612	1.703254
114	7	0	8.367404	-0.793612	-1.703254
115	7	0	-8.577873	-0.526995	-1.168793
116	7	0	8.577873	0.526995	1.168793
117	6	0	9.433709	-0.920438	-0.710309
118	6	0	-9.433709	0.920438	0.710309
119	6	0	9.715052	0.238702	0.272104
120	6	0	-9.715052	-0.238702	-0.272104
121	6	0	-1.597888	1.522602	10.547341
122	1	0	-1.864524	1.549462	9.487905
123	1	0	-1.846684	0.575666	11.010228
124	6	0	1.947495	-1.402837	10.753527
125	1	0	2.256143	-1.534808	9.713321
126	1	0	2.197340	-0.420263	11.134513
127	6	0	-1.947495	1.402837	-10.753527
128	1	0	-2.197340	0.420263	-11.134513

129	1	0	-2.256143	1.534808	-9.713321
130	6	0	1.597888	-1.522602	-10.547341
131	1	0	1.846684	-0.575666	-11.010228
132	1	0	1.864524	-1.549462	-9.487905
133	6	0	-2.177229	3.866419	10.768480
134	1	0	-2.848528	4.466352	11.374385
135	1	0	-1.158098	4.240567	10.861273
136	1	0	-2.492021	3.905206	9.720422
137	6	0	2.464859	-3.724372	11.223328
138	1	0	3.097435	-4.275676	11.911545
139	1	0	1.434080	-4.066461	11.310465
140	1	0	2.818480	-3.872598	10.197539
141	6	0	-2.464859	3.724372	-11.223328
142	1	0	-1.434080	4.066461	-11.310465
143	1	0	-3.097435	4.275676	-11.911545
144	1	0	-2.818480	3.872598	-10.197539
145	6	0	2.177229	-3.866419	-10.768480
146	1	0	1.158098	-4.240567	-10.861273
147	1	0	2.848528	-4.466352	-11.374385
148	1	0	2.492021	-3.905206	-9.720422
149	8	0	-2.280879	2.509317	11.288713
150	8	0	2.581704	-2.325283	11.612687
151	8	0	-2.581704	2.325283	-11.612687
152	8	0	2.280879	-2.509317	-11.288713
153	1	0	-9.949596	-1.151223	0.275941
154	1	0	-10.608003	0.040562	-0.846574
155	1	0	-9.217653	1.825723	0.134088
156	1	0	-10.354716	1.131719	1.262727
157	1	0	10.354716	-1.131719	-1.262727
158	1	0	9.217653	-1.825723	-0.134088
159	1	0	10.608003	-0.040562	0.846574
160	1	0	9.949596	1.151223	-0.275941

Table S 10: Coordinates of (*R*,*R*)-**12** for the Figure S62.

Standard orientation:

Center Number	Atomic Number	Atomic Type	Coordinates (Angstroms) X	Y	Z
1	6	0	10.880420	2.932273	0.318371
2	6	0	11.909120	3.376390	1.192014
3	6	0	12.932090	2.537024	1.567955
4	6	0	10.961980	1.602195	-0.216046
5	6	0	12.000676	0.714654	0.218483
6	6	0	12.953470	1.197333	1.103659
7	6	0	12.000676	-0.714654	-0.218483
8	6	0	10.961980	-1.602195	0.216046
9	6	0	12.953470	-1.197333	-1.103659

10	6	0	10.880420	-2.932273	-0.318371
11	6	0	11.909120	-3.376390	-1.192014
12	6	0	12.932090	-2.537024	-1.567955
13	6	0	8.874900	-2.010400	1.409795
14	6	0	8.729122	-3.298294	0.808855
15	6	0	9.758940	-3.747746	-0.003801
16	6	0	9.948135	1.200799	-1.132612
17	6	0	8.874900	2.010400	-1.409795
18	6	0	8.729122	3.298294	-0.808855
19	6	0	9.758940	3.747746	0.003801
20	1	0	11.877532	4.390636	1.569879
21	1	0	13.727902	2.887068	2.207318
22	1	0	11.877532	-4.390636	-1.569879
23	1	0	13.727902	-2.887068	-2.207318
24	1	0	8.094635	-1.639132	2.058768
25	1	0	9.714679	-4.738498	-0.438885
26	1	0	10.007548	0.222326	-1.584587
27	1	0	8.094635	1.639132	-2.058768
28	1	0	9.714679	4.738498	0.438885
29	6	0	9.948135	-1.200799	1.132612
30	1	0	10.007548	-0.222326	1.584587
31	8	0	13.940680	-0.291248	-1.522398
32	8	0	13.940680	0.291248	1.522398
33	6	0	-10.880420	-2.932273	0.318371
34	6	0	-11.909120	-3.376390	1.192014
35	6	0	-12.932090	-2.537024	1.567955
36	6	0	-10.961980	-1.602195	-0.216046
37	6	0	-12.000676	-0.714654	0.218483
38	6	0	-12.953470	-1.197333	1.103659
39	6	0	-12.000676	0.714654	-0.218483
40	6	0	-10.961980	1.602195	0.216046
41	6	0	-12.953470	1.197333	-1.103659
42	6	0	-10.880420	2.932273	-0.318371
43	6	0	-11.909120	3.376390	-1.192014
44	6	0	-12.932090	2.537024	-1.567955
45	6	0	-8.874900	2.010400	1.409795
46	6	0	-8.729122	3.298294	0.808855
47	6	0	-9.758940	3.747746	-0.003801
48	6	0	-9.948135	-1.200799	-1.132612
49	6	0	-8.874900	-2.010400	-1.409795
50	6	0	-8.729122	-3.298294	-0.808855
51	6	0	-9.758940	-3.747746	0.003801
52	1	0	-11.877532	-4.390636	1.569879
53	1	0	-13.727902	-2.887068	2.207318
54	1	0	-11.877532	4.390636	-1.569879
55	1	0	-13.727902	2.887068	-2.207318
56	1	0	-8.094635	1.639132	2.058768
57	1	0	-9.714679	4.738498	-0.438885
58	1	0	-10.007548	-0.222326	-1.584587
59	1	0	-8.094635	-1.639132	-2.058768

60	1	0	-9.714679	-4.738498	0.438885
61	6	0	-9.948135	1.200799	1.132612
62	1	0	-10.007548	0.222326	1.584587
63	8	0	-13.940680	0.291248	-1.522398
64	8	0	-13.940680	-0.291248	1.522398
65	6	0	-7.456336	-4.043577	-0.982975
66	6	0	-6.927142	-4.833584	0.061330
67	6	0	-6.669363	-3.881333	-2.139839
68	6	0	-5.643967	-5.362357	-0.009675
69	1	0	-7.501899	-4.969616	0.967096
70	6	0	-5.377642	-4.392918	-2.205107
71	1	0	-7.061056	-3.329228	-2.982578
72	6	0	-4.828629	-5.110105	-1.128185
73	1	0	-5.226725	-5.917841	0.818462
74	1	0	-4.774286	-4.211184	-3.086426
75	6	0	7.456336	4.043577	-0.982975
76	6	0	6.927142	4.833584	0.061330
77	6	0	6.669363	3.881333	-2.139839
78	6	0	5.643967	5.362357	-0.009675
79	1	0	7.501899	4.969616	0.967096
80	6	0	5.377642	4.392918	-2.205107
81	1	0	7.061056	3.329228	-2.982578
82	6	0	4.828629	5.110105	-1.128185
83	1	0	5.226725	5.917841	0.818462
84	1	0	4.774286	4.211184	-3.086426
85	6	0	7.456336	-4.043577	0.982975
86	6	0	6.669363	-3.881333	2.139839
87	6	0	6.927142	-4.833584	-0.061330
88	6	0	5.377642	-4.392918	2.205107
89	1	0	7.061056	-3.329228	2.982578
90	6	0	5.643967	-5.362357	0.009675
91	1	0	7.501899	-4.969616	-0.967096
92	6	0	4.828629	-5.110105	1.128185
93	1	0	4.774286	-4.211184	3.086426
94	1	0	5.226725	-5.917841	-0.818462
95	6	0	-7.456336	4.043577	0.982975
96	6	0	-6.669363	3.881333	2.139839
97	6	0	-6.927142	4.833584	-0.061330
98	6	0	-5.377642	4.392918	2.205107
99	1	0	-7.061056	3.329228	2.982578
100	6	0	-5.643967	5.362357	0.009675
101	1	0	-7.501899	4.969616	-0.967096
102	6	0	-4.828629	5.110105	1.128185
103	1	0	-4.774286	4.211184	3.086426
104	1	0	-5.226725	5.917841	-0.818462
105	6	0	-14.627304	-0.508129	2.778971
106	1	0	-15.070190	0.463047	2.998461
107	1	0	-13.930028	-0.820573	3.552963
108	6	0	-14.627304	0.508129	-2.778971
109	1	0	-15.070190	-0.463047	-2.998461

110	1	0	-13.930028	0.820573	-3.552963
111	6	0	14.627304	-0.508129	-2.778971
112	1	0	15.070190	0.463047	-2.998461
113	1	0	13.930028	-0.820573	-3.552963
114	6	0	14.627304	0.508129	2.778971
115	1	0	15.070190	-0.463047	2.998461
116	1	0	13.930028	0.820573	3.552963
117	6	0	-16.740620	-1.252703	1.829457
118	1	0	-17.242752	-0.324862	2.120328
119	1	0	-16.401216	-1.179193	0.796424
120	1	0	-17.422980	-2.089008	1.941759
121	6	0	-16.740620	1.252703	-1.829457
122	1	0	-17.242752	0.324862	-2.120328
123	1	0	-16.401216	1.179193	-0.796424
124	1	0	-17.422980	2.089008	-1.941759
125	6	0	16.740620	-1.252703	-1.829457
126	1	0	17.242752	-0.324862	-2.120328
127	1	0	16.401216	-1.179193	-0.796424
128	1	0	17.422980	-2.089008	-1.941759
129	6	0	16.740620	1.252703	1.829457
130	1	0	17.242752	0.324862	2.120328
131	1	0	16.401216	1.179193	0.796424
132	1	0	17.422980	2.089008	1.941759
133	8	0	-15.621650	-1.531598	2.723517
134	8	0	-15.621650	1.531598	-2.723517
135	8	0	15.621650	-1.531598	-2.723517
136	8	0	15.621650	1.531598	2.723517
137	6	0	-3.401853	5.434242	1.135573
138	1	0	-2.819553	4.971916	1.940831
139	6	0	3.401853	5.434242	-1.135573
140	1	0	2.819553	4.971916	-1.940831
141	6	0	3.401853	-5.434242	1.135573
142	1	0	2.819553	-4.971916	1.940831
143	6	0	-3.401853	-5.434242	-1.135573
144	1	0	-2.819553	-4.971916	-1.940831
145	7	0	2.817725	6.146603	-0.231793
146	7	0	-2.817725	6.146603	0.231793
147	7	0	2.817725	-6.146603	0.231793
148	7	0	-2.817725	-6.146603	-0.231793
149	6	0	1.403580	6.162170	-0.144814
150	6	0	-1.403580	6.162170	0.144814
151	6	0	1.403580	-6.162170	0.144814
152	6	0	-1.403580	-6.162170	-0.144814
153	6	0	0.690532	4.952413	-0.081279
154	6	0	-0.694327	7.373408	0.052259
155	6	0	0.694327	-7.373408	0.052259
156	6	0	-0.690532	-4.952413	-0.081279
157	6	0	-0.690532	4.952413	0.081279
158	6	0	0.694327	7.373408	-0.052259
159	6	0	0.690532	-4.952413	0.081279

160	6	0	-0.694327	-7.373408	-0.052259
161	1	0	-1.231196	4.016330	0.119704
162	1	0	1.231196	4.016330	-0.119704
163	1	0	1.245143	8.302888	-0.084287
164	1	0	-1.245143	8.302888	0.084287
165	1	0	-1.231196	-4.016330	-0.119704
166	1	0	1.231196	-4.016330	0.119704
167	1	0	1.245143	-8.302888	0.084287
168	1	0	-1.245143	-8.302888	-0.084287

Table S 11: Coordinates of (*R*,*S*)-**12** for the Figure S63.

Standard orientation:

Center Number	Atomic Number	Atomic Type	Coordinates (Angstroms) X	Y	Z
1	6	0	2.775914	0.978827	10.889580
2	6	0	3.033001	1.930202	11.918221
3	6	0	2.148744	2.096174	12.951187
4	6	0	1.593218	0.173093	10.982384
5	6	0	0.638614	0.392234	12.043688
6	6	0	0.938865	1.351798	13.003815
7	6	0	-0.638614	-0.392234	12.043688
8	6	0	-1.593218	-0.173093	10.982384
9	6	0	-0.938865	-1.351798	13.003815
10	6	0	-2.775914	-0.978827	10.889580
11	6	0	-3.033001	-1.930202	11.918221
12	6	0	-2.148744	-2.096174	12.951187
13	6	0	-2.291482	0.984110	8.949898
14	6	0	-3.414470	0.121465	8.792338
15	6	0	-3.649477	-0.822537	9.780392
16	6	0	1.414114	-0.840287	9.998839
17	6	0	2.291482	-0.984110	8.949898
18	6	0	3.414470	-0.121465	8.792338
19	6	0	3.649477	0.822537	9.780392
20	1	0	3.936408	2.524912	11.871804
21	1	0	2.328719	2.805548	13.745828
22	1	0	-3.936408	-2.524912	11.871804
23	1	0	-2.328719	-2.805548	13.745828
24	1	0	-2.097995	1.735742	8.196922
25	1	0	-4.520733	-1.463190	9.719363
26	1	0	0.555541	-1.491008	10.063501
27	1	0	2.097995	-1.735742	8.196922
28	1	0	4.520733	1.463190	9.719363
29	6	0	-1.414114	0.840287	9.998839
30	1	0	-0.555541	1.491008	10.063501
31	8	0	-0.168718	-1.622943	14.134191

32	8	0	0.168718	1.622943	14.134191
33	6	0	-2.775914	-0.978827	-10.889580
34	6	0	-3.033001	-1.930202	-11.918221
35	6	0	-2.148744	-2.096174	-12.951187
36	6	0	-1.593218	-0.173093	-10.982384
37	6	0	-0.938865	-1.351798	-13.003815
38	6	0	1.593218	0.173093	-10.982384
39	6	0	0.938865	1.351798	-13.003815
40	6	0	2.775914	0.978827	-10.889580
41	6	0	3.033001	1.930202	-11.918221
42	6	0	2.148744	2.096174	-12.951187
43	6	0	2.291482	-0.984110	-8.949898
44	6	0	3.414470	-0.121465	-8.792338
45	6	0	3.649477	0.822537	-9.780392
46	6	0	-1.414114	0.840287	-9.998839
47	6	0	-2.291482	0.984110	-8.949898
48	6	0	-3.414470	0.121465	-8.792338
49	6	0	-3.649477	-0.822537	-9.780392
50	1	0	-3.936408	-2.524912	-11.871804
51	1	0	-2.328719	-2.805548	-13.745828
52	1	0	3.936408	2.524912	-11.871804
53	1	0	2.328719	2.805548	-13.745828
54	1	0	2.097995	-1.735742	-8.196922
55	1	0	4.520733	1.463190	-9.719363
56	1	0	-0.555541	1.491008	-10.063501
57	1	0	-2.097995	1.735742	-8.196922
58	1	0	-4.520733	-1.463190	-9.719363
59	6	0	1.414114	-0.840287	-9.998839
60	1	0	0.555541	-1.491008	-10.063501
61	8	0	0.168718	1.622943	-14.134191
62	8	0	-0.168718	-1.622943	-14.134191
63	6	0	0.638614	0.392234	-12.043688
64	6	0	-0.638614	-0.392234	-12.043688
65	6	0	4.226053	-0.212356	-7.552430
66	6	0	4.544049	-1.458607	-6.982340
67	6	0	4.598574	0.952166	-6.848090
68	6	0	5.141309	-1.537768	-5.727454
69	1	0	4.307761	-2.367212	-7.519558
70	6	0	5.184121	0.875400	-5.590610
71	1	0	4.358676	1.920895	-7.265634
72	6	0	5.432547	-0.374594	-4.993187
73	1	0	5.350399	-2.508384	-5.293820
74	1	0	5.408665	1.769051	-5.026167
75	6	0	-4.226053	0.212356	7.552430
76	6	0	-4.544049	1.458607	6.982340
77	6	0	-4.598574	-0.952166	6.848090
78	6	0	-5.141309	1.537768	5.727454
79	1	0	-4.307761	2.367212	7.519558
80	6	0	-5.184121	-0.875400	5.590610
81	1	0	-4.358676	-1.920895	7.265634

82	6	0	-5.432547	0.374594	4.993187
83	1	0	-5.350399	2.508384	5.293820
84	1	0	-5.408665	-1.769051	5.026167
85	6	0	4.226053	-0.212356	7.552430
86	6	0	4.598574	0.952166	6.848090
87	6	0	4.544049	-1.458607	6.982340
88	6	0	5.184121	0.875400	5.590610
89	1	0	4.358676	1.920895	7.265634
90	6	0	5.141309	-1.537768	5.727454
91	1	0	4.307761	-2.367212	7.519558
92	6	0	5.432547	-0.374594	4.993187
93	1	0	5.408665	1.769051	5.026167
94	1	0	5.350399	-2.508384	5.293820
95	6	0	-4.226053	0.212356	-7.552430
96	6	0	-4.598574	-0.952166	-6.848090
97	6	0	-4.544049	1.458607	-6.982340
98	6	0	-5.184121	-0.875400	-5.590610
99	1	0	-4.358676	-1.920895	-7.265634
100	6	0	-5.141309	1.537768	-5.727454
101	1	0	-4.307761	2.367212	-7.519558
102	6	0	-5.432547	0.374594	-4.993187
103	1	0	-5.408665	-1.769051	-5.026167
104	1	0	-5.350399	2.508384	-5.293820
105	6	0	-5.846673	0.492880	-3.594980
106	1	0	-5.976974	1.515663	-3.223586
107	6	0	-5.846673	0.492880	3.594980
108	1	0	-5.976974	1.515663	3.223586
109	6	0	5.846673	-0.492880	-3.594980
110	1	0	5.976974	-1.515663	-3.223586
111	6	0	5.846673	-0.492880	3.594980
112	1	0	5.976974	-1.515663	3.223586
113	7	0	-5.972702	-0.528358	2.815989
114	7	0	5.972702	0.528358	-2.815989
115	7	0	-5.972702	-0.528358	-2.815989
116	7	0	5.972702	0.528358	2.815989
117	6	0	6.085305	0.401468	-1.413539
118	6	0	-6.085305	-0.401468	1.413539
119	6	0	5.407148	-0.601855	-0.695125
120	6	0	-5.407148	0.601855	0.695125
121	6	0	6.085305	0.401468	1.413539
122	6	0	-6.085305	-0.401468	-1.413539
123	6	0	5.407148	-0.601855	0.695125
124	6	0	-5.407148	0.601855	-0.695125
125	6	0	6.771344	1.398652	-0.694680
126	6	0	-6.771344	-1.398652	0.694680
127	6	0	6.771344	1.398652	0.694680
128	6	0	-6.771344	-1.398652	-0.694680
129	1	0	4.812777	-1.333228	1.225461
130	1	0	-4.812777	1.333228	1.225461
131	1	0	-7.265201	-2.182726	1.250705

132	1	0	-7.265201	-2.182726	-1.250705
133	1	0	-4.812777	1.333228	-1.225461
134	1	0	4.812777	-1.333228	-1.225461
135	1	0	7.265201	2.182726	-1.250705
136	1	0	7.265201	2.182726	1.250705
137	6	0	-1.297199	1.752829	14.098510
138	1	0	-1.611585	1.876482	13.058831
139	1	0	-1.734815	0.876710	14.560269
140	6	0	1.297199	-1.752829	14.098510
141	1	0	1.611585	-1.876482	13.058831
142	1	0	1.734815	-0.876710	14.560269
143	6	0	-1.297199	1.752829	-14.098510
144	1	0	-1.734815	0.876710	-14.560269
145	1	0	-1.611585	1.876482	-13.058831
146	6	0	1.297199	-1.752829	-14.098510
147	1	0	1.734815	-0.876710	-14.560269
148	1	0	1.611585	-1.876482	-13.058831
149	6	0	-1.284809	4.158727	14.400411
150	1	0	-1.753962	4.880370	15.061258
151	1	0	-0.201819	4.274993	14.432507
152	1	0	-1.643090	4.308524	13.376543
153	6	0	1.284809	-4.158727	14.400411
154	1	0	1.753962	-4.880370	15.061258
155	1	0	0.201819	-4.274993	14.432507
156	1	0	1.643090	-4.308524	13.376543
157	6	0	-1.284809	4.158727	-14.400411
158	1	0	-0.201819	4.274993	-14.432507
159	1	0	-1.753962	4.880370	-15.061258
160	1	0	-1.643090	4.308524	-13.376543
161	6	0	1.284809	-4.158727	-14.400411
162	1	0	0.201819	-4.274993	-14.432507
163	1	0	1.753962	-4.880370	-15.061258
164	1	0	1.643090	-4.308524	-13.376543
165	8	0	-1.681136	2.848574	14.899758
166	8	0	1.681136	-2.848574	14.899758
167	8	0	-1.681136	2.848574	-14.899758
168	8	0	1.681136	-2.848574	-14.899758

Table S 12: Coordinates of (*R*,*R*)-**13** for the Figure S65.

Standard orientation:

Center Number	Atomic Number	Atomic Type	Coordinates (Angstroms) X	Y	Z
1	6	0	13.160232	2.690221	1.264525
2	6	0	14.126833	2.735346	2.305165
3	6	0	15.093429	1.763281	2.419642
4	6	0	13.227077	1.620161	0.308701

5	6	0	14.208000	0.589291	0.459589
6	6	0	15.113396	0.679854	1.505439
7	6	0	14.208000	-0.589291	-0.459589
8	6	0	13.227077	-1.620161	-0.308701
9	6	0	15.113396	-0.679854	-1.505439
10	6	0	13.160232	-2.690221	-1.264525
11	6	0	14.126833	-2.735346	-2.305165
12	6	0	15.093429	-1.763281	-2.419642
13	6	0	11.290472	-2.566077	0.834315
14	6	0	11.176422	-3.597863	-0.151059
15	6	0	12.123802	-3.655334	-1.163998
16	6	0	12.275737	1.615085	-0.753565
17	6	0	11.290472	2.566077	-0.834315
18	6	0	11.176422	3.597863	0.151059
19	6	0	12.123802	3.655334	1.163998
20	1	0	14.098548	3.553871	3.013225
21	1	0	15.850793	1.824144	3.185947
22	1	0	14.098548	-3.553871	-3.013225
23	1	0	15.850793	-1.824144	-3.185947
24	1	0	10.567725	-2.535666	1.637622
25	1	0	12.063558	-4.437433	-1.909676
26	1	0	12.328887	0.834904	-1.498423
27	1	0	10.567725	2.535666	-1.637622
28	1	0	12.063558	4.437433	1.909676
29	6	0	12.275737	-1.615085	0.753565
30	1	0	12.328887	-0.834904	1.498423
31	8	0	16.054663	0.356544	-1.607318
32	8	0	16.054663	-0.356544	1.607318
33	6	0	-13.160232	-2.690221	1.264525
34	6	0	-14.126833	-2.735346	2.305165
35	6	0	-15.093429	-1.763281	2.419642
36	6	0	-13.227077	-1.620161	0.308701
37	6	0	-14.208000	-0.589291	0.459589
38	6	0	-15.113396	-0.679854	1.505439
39	6	0	-14.208000	0.589291	-0.459589
40	6	0	-13.227077	1.620161	-0.308701
41	6	0	-15.113396	0.679854	-1.505439
42	6	0	-13.160232	2.690221	-1.264525
43	6	0	-14.126833	2.735346	-2.305165
44	6	0	-15.093429	1.763281	-2.419642
45	6	0	-11.290472	2.566077	0.834315
46	6	0	-11.176422	3.597863	-0.151059
47	6	0	-12.123802	3.655334	-1.163998
48	6	0	-12.275737	-1.615085	-0.753565
49	6	0	-11.290472	-2.566077	-0.834315
50	6	0	-11.176422	-3.597863	0.151059
51	6	0	-12.123802	-3.655334	1.163998
52	1	0	-14.098548	-3.553871	3.013225
53	1	0	-15.850793	-1.824144	3.185947
54	1	0	-14.098548	3.553871	-3.013225

55	1	0	-15.850793	1.824144	-3.185947
56	1	0	-10.567725	2.535666	1.637622
57	1	0	-12.063558	4.437433	-1.909676
58	1	0	-12.328887	-0.834904	-1.498423
59	1	0	-10.567725	-2.535666	-1.637622
60	1	0	-12.063558	-4.437433	1.909676
61	6	0	-12.275737	1.615085	0.753565
62	1	0	-12.328887	0.834904	1.498423
63	8	0	-16.054663	-0.356544	-1.607318
64	8	0	-16.054663	0.356544	1.607318
65	6	0	-7.749221	-5.750165	-0.059318
66	6	0	-6.822095	-5.602793	0.999573
67	6	0	-7.342419	-6.446261	-1.215774
68	6	0	-5.524807	-6.074060	0.877504
69	1	0	-7.130251	-5.082725	1.895762
70	6	0	-6.041864	-6.928788	-1.327655
71	1	0	-8.045096	-6.579245	-2.026328
72	6	0	-5.104534	-6.725087	-0.298982
73	1	0	-4.802023	-5.924943	1.666265
74	1	0	-5.737712	-7.439856	-2.233234
75	6	0	7.749221	5.750165	-0.059318
76	6	0	6.822095	5.602793	0.999573
77	6	0	7.342419	6.446261	-1.215774
78	6	0	5.524807	6.074060	0.877504
79	1	0	7.130251	5.082725	1.895762
80	6	0	6.041864	6.928788	-1.327655
81	1	0	8.045096	6.579245	-2.026328
82	6	0	5.104534	6.725087	-0.298982
83	1	0	4.802023	5.924943	1.666265
84	1	0	5.737712	7.439856	-2.233234
85	6	0	7.749221	-5.750165	0.059318
86	6	0	7.342419	-6.446261	1.215774
87	6	0	6.822095	-5.602793	-0.999573
88	6	0	6.041864	-6.928788	1.327655
89	1	0	8.045096	-6.579245	2.026328
90	6	0	5.524807	-6.074060	-0.877504
91	1	0	7.130251	-5.082725	-1.895762
92	6	0	5.104534	-6.725087	0.298982
93	1	0	5.737712	-7.439856	2.233234
94	1	0	4.802023	-5.924943	-1.666265
95	6	0	-7.749221	5.750165	0.059318
96	6	0	-7.342419	6.446261	1.215774
97	6	0	-6.822095	5.602793	-0.999573
98	6	0	-6.041864	6.928788	1.327655
99	1	0	-8.045096	6.579245	2.026328
100	6	0	-5.524807	6.074060	-0.877504
101	1	0	-7.130251	5.082725	-1.895762
102	6	0	-5.104534	6.725087	0.298982
103	1	0	-5.737712	7.439856	2.233234
104	1	0	-4.802023	5.924943	-1.666265

105	6	0	-16.691138	0.628641	2.880024
106	1	0	-17.101712	1.629575	2.749922
107	1	0	-15.967429	0.589095	3.690911
108	6	0	-16.691138	-0.628641	-2.880024
109	1	0	-17.101712	-1.629575	-2.749922
110	1	0	-15.967429	-0.589095	-3.690911
111	6	0	16.691138	0.628641	-2.880024
112	1	0	17.101712	1.629575	-2.749922
113	1	0	15.967429	0.589095	-3.690911
114	6	0	16.691138	-0.628641	2.880024
115	1	0	17.101712	-1.629575	2.749922
116	1	0	15.967429	-0.589095	3.690911
117	6	0	-18.862342	-0.324813	2.333618
118	1	0	-19.326635	0.665043	2.289684
119	1	0	-18.571814	-0.639741	1.331459
120	1	0	-19.558725	-1.037554	2.763314
121	6	0	-18.862342	0.324813	-2.333618
122	1	0	-19.326635	-0.665043	-2.289684
123	1	0	-18.571814	0.639741	-1.331459
124	1	0	-19.558725	1.037554	-2.763314
125	6	0	18.862342	-0.324813	-2.333618
126	1	0	19.326635	0.665043	-2.289684
127	1	0	18.571814	-0.639741	-1.331459
128	1	0	19.558725	-1.037554	-2.763314
129	6	0	18.862342	0.324813	2.333618
130	1	0	19.326635	-0.665043	2.289684
131	1	0	18.571814	0.639741	1.331459
132	1	0	19.558725	1.037554	2.763314
133	8	0	-17.708666	-0.310314	3.227305
134	8	0	-17.708666	0.310314	-3.227305
135	8	0	17.708666	-0.310314	-3.227305
136	8	0	17.708666	0.310314	3.227305
137	6	0	-3.704323	7.100732	0.497383
138	1	0	-3.474688	7.618201	1.435255
139	6	0	3.704323	7.100732	-0.497383
140	1	0	3.474688	7.618201	-1.435255
141	6	0	3.704323	-7.100732	0.497383
142	1	0	3.474688	-7.618201	1.435255
143	6	0	-3.704323	-7.100732	-0.497383
144	1	0	-3.474688	-7.618201	-1.435255
145	7	0	2.791553	6.790662	0.361086
146	7	0	-2.791553	6.790662	-0.361086
147	7	0	2.791553	-6.790662	-0.361086
148	7	0	-2.791553	-6.790662	0.361086
149	6	0	1.407310	6.940988	0.136178
150	6	0	-1.407310	6.940988	-0.136178
151	6	0	1.407310	-6.940988	-0.136178
152	6	0	-1.407310	-6.940988	0.136178
153	6	0	0.573750	6.944578	1.269522
154	6	0	-0.573750	6.944578	-1.269522

155	6	0	0.573750	-6.944578	-1.269522
156	6	0	-0.573750	-6.944578	1.269522
157	6	0	-0.809025	6.953560	1.140781
158	6	0	0.809025	6.953560	-1.140781
159	6	0	0.809025	-6.953560	1.140781
160	6	0	-0.809025	-6.953560	-1.140781
161	1	0	-1.424117	6.901242	2.028736
162	1	0	1.042940	6.898387	2.241813
163	1	0	1.424117	6.901242	-2.028736
164	1	0	-1.042940	6.898387	-2.241813
165	1	0	-1.042940	-6.898387	2.241813
166	1	0	1.424117	-6.901242	2.028736
167	1	0	1.042940	-6.898387	-2.241813
168	1	0	-1.424117	-6.901242	-2.028736
169	6	0	10.057696	4.483501	0.096939
170	6	0	-10.057696	4.483501	-0.096939
171	6	0	-10.057696	-4.483501	0.096939
172	6	0	10.057696	-4.483501	-0.096939
173	6	0	-9.029498	5.125972	-0.020745
174	6	0	9.029498	5.125972	0.020745
175	6	0	-9.029498	-5.125972	0.020745
176	6	0	9.029498	-5.125972	-0.020745

Table S 13: Coordinates of (*R*,*S*)-**13** for the Figure S66.

Standard orientation:

Center Number	Atomic Number	Atomic Type	Coordinates (Angstroms) X	Y	Z
1	6	0	2.727089	1.152120	13.276695
2	6	0	2.905096	2.120216	14.306737
3	6	0	1.991501	2.234763	15.320384
4	6	0	1.588162	0.281652	13.340646
5	6	0	0.608744	0.437079	14.388280
6	6	0	0.830932	1.414978	15.352790
7	6	0	-0.608744	-0.437079	14.388280
8	6	0	-1.588162	-0.281652	13.340646
9	6	0	-0.830932	-1.414978	15.352790
10	6	0	-2.727089	-1.152120	13.276695
11	6	0	-2.905096	-2.120216	14.306737
12	6	0	-1.991501	-2.234763	15.320384
13	6	0	-2.390089	0.837693	11.323657
14	6	0	-3.477878	-0.081634	11.205283
15	6	0	-3.640068	-1.041328	12.197551
16	6	0	1.482323	-0.736696	12.348783
17	6	0	2.390089	-0.837693	11.323657
18	6	0	3.477878	0.081634	11.205283
19	6	0	3.640068	1.041328	12.197551

20	1	0	3.772650	2.766795	14.276858
21	1	0	2.111513	2.955148	16.116313
22	1	0	-3.772650	-2.766795	14.276858
23	1	0	-2.111513	-2.955148	16.116313
24	1	0	-2.278835	1.608487	10.574036
25	1	0	-4.472412	-1.730809	12.144596
26	1	0	0.660779	-1.434971	12.397583
27	1	0	2.278835	-1.608487	10.574036
28	1	0	4.472412	1.730809	12.144596
29	6	0	-1.482323	0.736696	12.348783
30	1	0	-0.660779	1.434971	12.397583
31	8	0	-0.027885	-1.635965	16.469449
32	8	0	0.027885	1.635965	16.469449
33	6	0	-2.727089	-1.152120	-13.276695
34	6	0	-2.905096	-2.120216	-14.306737
35	6	0	-1.991501	-2.234763	-15.320384
36	6	0	-1.588162	-0.281652	-13.340646
37	6	0	-0.830932	-1.414978	-15.352790
38	6	0	1.588162	0.281652	-13.340646
39	6	0	0.830932	1.414978	-15.352790
40	6	0	2.727089	1.152120	-13.276695
41	6	0	2.905096	2.120216	-14.306737
42	6	0	1.991501	2.234763	-15.320384
43	6	0	2.390089	-0.837693	-11.323657
44	6	0	3.477878	0.081634	-11.205283
45	6	0	3.640068	1.041328	-12.197551
46	6	0	-1.482323	0.736696	-12.348783
47	6	0	-2.390089	0.837693	-11.323657
48	6	0	-3.477878	-0.081634	-11.205283
49	6	0	-3.640068	-1.041328	-12.197551
50	1	0	-3.772650	-2.766795	-14.276858
51	1	0	-2.111513	-2.955148	-16.116313
52	1	0	3.772650	2.766795	-14.276858
53	1	0	2.111513	2.955148	-16.116313
54	1	0	2.278835	-1.608487	-10.574036
55	1	0	4.472412	1.730809	-12.144596
56	1	0	-0.660779	1.434971	-12.397583
57	1	0	-2.278835	1.608487	-10.574036
58	1	0	-4.472412	-1.730809	-12.144596
59	6	0	1.482323	-0.736696	-12.348783
60	1	0	0.660779	-1.434971	-12.397583
61	8	0	0.027885	1.635965	-16.469449
62	8	0	-0.027885	-1.635965	-16.469449
63	6	0	0.608744	0.437079	-14.388280
64	6	0	-0.608744	-0.437079	-14.388280
65	6	0	5.476415	-0.189330	-7.696028
66	6	0	5.507047	-1.440977	-7.045003
67	6	0	5.933897	0.948889	-6.990932
68	6	0	5.923809	-1.537012	-5.723228
69	1	0	5.176760	-2.321002	-7.578606

70	6	0	6.339956	0.847796	-5.668217
71	1	0	5.928824	1.909787	-7.486202
72	6	0	6.317464	-0.393951	-5.003799
73	1	0	5.915902	-2.500871	-5.228889
74	1	0	6.649523	1.722060	-5.113805
75	6	0	-5.476415	0.189330	7.696028
76	6	0	-5.507047	1.440977	7.045003
77	6	0	-5.933897	-0.948889	6.990932
78	6	0	-5.923809	1.537012	5.723228
79	1	0	-5.176760	2.321002	7.578606
80	6	0	-6.339956	-0.847796	5.668217
81	1	0	-5.928824	-1.909787	7.486202
82	6	0	-6.317464	0.393951	5.003799
83	1	0	-5.915902	2.500871	5.228889
84	1	0	-6.649523	-1.722060	5.113805
85	6	0	5.476415	-0.189330	7.696028
86	6	0	5.933897	0.948889	6.990932
87	6	0	5.507047	-1.440977	7.045003
88	6	0	6.339956	0.847796	5.668217
89	1	0	5.928824	1.909787	7.486202
90	6	0	5.923809	-1.537012	5.723228
91	1	0	5.176760	-2.321002	7.578606
92	6	0	6.317464	-0.393951	5.003799
93	1	0	6.649523	1.722060	5.113805
94	1	0	5.915902	-2.500871	5.228889
95	6	0	-5.476415	0.189330	-7.696028
96	6	0	-5.933897	-0.948889	-6.990932
97	6	0	-5.507047	1.440977	-7.045003
98	6	0	-6.339956	-0.847796	-5.668217
99	1	0	-5.928824	-1.909787	-7.486202
100	6	0	-5.923809	1.537012	-5.723228
101	1	0	-5.176760	2.321002	-7.578606
102	6	0	-6.317464	0.393951	-5.003799
103	1	0	-6.649523	-1.722060	-5.113805
104	1	0	-5.915902	2.500871	-5.228889
105	6	0	-6.594127	0.511310	-3.573258
106	1	0	-6.521940	1.523066	-3.159033
107	6	0	-6.594127	0.511310	3.573258
108	1	0	-6.521940	1.523066	3.159033
109	6	0	6.594127	-0.511310	-3.573258
110	1	0	6.521940	-1.523066	-3.159033
111	6	0	6.594127	-0.511310	3.573258
112	1	0	6.521940	-1.523066	3.159033
113	7	0	-6.854592	-0.505211	2.821269
114	7	0	6.854592	0.505211	-2.821269
115	7	0	-6.854592	-0.505211	-2.821269
116	7	0	6.854592	0.505211	2.821269
117	6	0	6.895942	0.399991	-1.412600
118	6	0	-6.895942	-0.399991	1.412600
119	6	0	6.032032	-0.449290	-0.695168

120	6	0	-6.032032	0.449290	0.695168
121	6	0	6.895942	0.399991	1.412600
122	6	0	-6.895942	-0.399991	-1.412600
123	6	0	6.032032	-0.449290	0.695168
124	6	0	-6.032032	0.449290	-0.695168
125	6	0	7.747083	1.260816	-0.694713
126	6	0	-7.747083	-1.260816	0.694713
127	6	0	7.747083	1.260816	0.694713
128	6	0	-7.747083	-1.260816	-0.694713
129	1	0	5.313647	-1.058103	1.226892
130	1	0	-5.313647	1.058103	1.226892
131	1	0	-8.381959	-1.934956	1.251607
132	1	0	-8.381959	-1.934956	-1.251607
133	1	0	-5.313647	1.058103	-1.226892
134	1	0	5.313647	-1.058103	-1.226892
135	1	0	8.381959	1.934956	-1.251607
136	1	0	8.381959	1.934956	1.251607
137	6	0	-1.443962	1.667733	16.414214
138	1	0	-1.750789	1.778345	15.370856
139	1	0	-1.828373	0.760717	16.863473
140	6	0	1.443962	-1.667733	16.414214
141	1	0	1.750789	-1.778345	15.370856
142	1	0	1.828373	-0.760717	16.863473
143	6	0	-1.443962	1.667733	-16.414214
144	1	0	-1.828373	0.760717	-16.863473
145	1	0	-1.750789	1.778345	-15.370856
146	6	0	1.443962	-1.667733	-16.414214
147	1	0	1.828373	-0.760717	-16.863473
148	1	0	1.750789	-1.778345	-15.370856
149	6	0	-1.598853	4.066850	16.731493
150	1	0	-2.127215	4.749101	17.389392
151	1	0	-0.527297	4.258273	16.781009
152	1	0	-1.951807	4.197927	15.703332
153	6	0	1.598853	-4.066850	16.731493
154	1	0	2.127215	-4.749101	17.389392
155	1	0	0.527297	-4.258273	16.781009
156	1	0	1.951807	-4.197927	15.703332
157	6	0	-1.598853	4.066850	-16.731493
158	1	0	-0.527297	4.258273	-16.781009
159	1	0	-2.127215	4.749101	-17.389392
160	1	0	-1.951807	4.197927	-15.703332
161	6	0	1.598853	-4.066850	-16.731493
162	1	0	0.527297	-4.258273	-16.781009
163	1	0	2.127215	-4.749101	-17.389392
164	1	0	1.951807	-4.197927	-15.703332
165	8	0	-1.909694	2.728502	17.217409
166	8	0	1.909694	-2.728502	17.217409
167	8	0	-1.909694	2.728502	-17.217409
168	8	0	1.909694	-2.728502	-17.217409
169	6	0	-4.314501	-0.012618	10.053455

170	6	0	4.314501	0.012618	10.053455
171	6	0	4.314501	0.012618	-10.053455
172	6	0	-4.314501	-0.012618	-10.053455
173	6	0	-4.908790	0.074726	8.997292
174	6	0	4.908790	-0.074726	8.997292
175	6	0	4.908790	-0.074726	-8.997292
176	6	0	-4.908790	0.074726	-8.997292

Table S 14: Coordinates of (*R*,*R*)-**14** in Figure S68.

Standard orientation:

Center Number	Atomic Number	Atomic Type	Coordinates (Angstroms) X	Y	Z
1	6	0	6.545601	-5.128548	0.075459
2	6	0	6.628929	-4.287974	1.205052
3	6	0	6.522762	-4.536218	-1.197865
4	6	0	6.605075	-2.904505	1.075913
5	1	0	6.647072	-4.730957	2.192269
6	6	0	6.508752	-3.149508	-1.326421
7	1	0	6.468276	-5.162759	-2.074781
8	6	0	6.505986	-2.312933	-0.195780
9	1	0	6.603411	-2.263257	1.945531
10	1	0	6.450187	-2.707741	-2.314268
11	6	0	-6.545601	5.128548	0.075459
12	6	0	-6.628929	4.287974	1.205052
13	6	0	-6.522762	4.536218	-1.197865
14	6	0	-6.605075	2.904505	1.075913
15	1	0	-6.647072	4.730957	2.192269
16	6	0	-6.508752	3.149508	-1.326421
17	1	0	-6.468276	5.162759	-2.074781
18	6	0	-6.505986	2.312933	-0.195780
19	1	0	-6.603411	2.263257	1.945531
20	1	0	-6.450187	2.707741	-2.314268
21	6	0	0.005721	8.432658	-0.066210
22	6	0	0.429958	8.218022	1.255137
23	6	0	0.756800	7.861383	-1.114402
24	6	0	1.528583	7.402080	1.515283
25	1	0	-0.130370	8.650383	2.069910
26	6	0	1.840993	7.032829	-0.853194
27	1	0	0.440963	8.020416	-2.137160
28	6	0	2.222693	6.761701	0.472748
29	1	0	1.822378	7.216845	2.541858
30	1	0	2.373517	6.541572	-1.654782
31	6	0	-0.005721	-8.432658	-0.066210
32	6	0	-0.429958	-8.218022	1.255137
33	6	0	-0.756800	-7.861383	-1.114402
34	6	0	-1.528583	-7.402080	1.515283

35	1	0	0.130370	-8.650383	2.069910
36	6	0	-1.840993	-7.032829	-0.853194
37	1	0	-0.440963	-8.020416	-2.137160
38	6	0	-2.222693	-6.761701	0.472748
39	1	0	-1.822378	-7.216845	2.541858
40	1	0	-2.373517	-6.541572	-1.654782
41	6	0	-3.209872	-5.728562	0.788960
42	1	0	-3.417566	-5.579520	1.854593
43	6	0	-6.251475	0.880692	-0.358401
44	1	0	-6.128128	0.536976	-1.391346
45	6	0	3.209872	5.728562	0.788960
46	1	0	3.417566	5.579520	1.854593
47	6	0	6.251475	-0.880692	-0.358401
48	1	0	6.128128	-0.536976	-1.391346
49	7	0	-6.062708	0.090311	0.644960
50	7	0	-3.738993	-4.977162	-0.117480
51	7	0	3.738993	4.977162	-0.117480
52	7	0	6.062708	-0.090311	0.644960
53	6	0	-5.644835	-1.252059	0.480039
54	6	0	-4.447943	-3.792605	0.178448
55	6	0	4.447943	3.792605	0.178448
56	6	0	5.644835	1.252059	0.480039
57	6	0	-4.756505	-1.745262	1.450081
58	6	0	-5.393740	-3.329105	-0.753999
59	6	0	5.393740	3.329105	-0.753999
60	6	0	4.756505	1.745262	1.450081
61	6	0	-4.154119	-2.987242	1.297517
62	6	0	-5.992997	-2.082314	-0.603876
63	6	0	4.154119	2.987242	1.297517
64	6	0	5.992997	2.082314	-0.603876
65	1	0	-3.407236	-3.307533	2.010781
66	1	0	-4.510650	-1.103467	2.284015
67	1	0	-6.716016	-1.743719	-1.334012
68	1	0	-5.620556	-3.955919	-1.605056
69	1	0	4.510650	1.103467	2.284015
70	1	0	3.407236	3.307533	2.010781
71	1	0	5.620556	3.955919	-1.605056
72	1	0	6.716016	1.743719	-1.334012
73	6	0	-7.073463	8.643166	1.461768
74	6	0	-7.976808	9.335618	2.316407
75	6	0	-7.732854	10.634553	2.703528
76	6	0	-5.905787	9.322579	0.978310
77	6	0	-5.671710	10.657462	1.418871
78	6	0	-6.562601	11.295982	2.256467
79	6	0	-4.892171	10.848818	-1.880332
80	6	0	-3.700398	10.297837	-1.326337
81	6	0	-4.838853	11.854659	-2.822853
82	6	0	-2.439715	10.787325	-1.806067
83	6	0	-2.421563	11.832906	-2.771586
84	6	0	-3.592783	12.360967	-3.267568

85	6	0	-2.506847	8.757385	0.132868
86	6	0	-1.245529	9.169414	-0.403752
87	6	0	-1.242524	10.180426	-1.345303
88	6	0	-5.001830	8.623643	0.106020
89	6	0	-5.295242	7.317214	-0.260331
90	6	0	-6.406772	6.597853	0.284641
91	6	0	-7.272791	7.281266	1.116727
92	1	0	-8.860833	8.815765	2.664310
93	1	0	-8.427091	11.150293	3.353316
94	1	0	-1.463523	12.202078	-3.116037
95	1	0	-3.566062	13.155777	-4.000875
96	1	0	-0.298187	10.520599	-1.751128
97	1	0	-8.131470	6.762989	1.524401
98	6	0	-3.714573	9.249861	-0.342105
99	6	0	7.073463	-8.643166	1.461768
100	6	0	7.976808	-9.335618	2.316407
101	6	0	7.732854	-10.634553	2.703528
102	6	0	5.905787	-9.322579	0.978310
103	6	0	5.671710	-10.657462	1.418871
104	6	0	6.562601	-11.295982	2.256467
105	6	0	4.892171	-10.848818	-1.880332
106	6	0	3.700398	-10.297837	-1.326337
107	6	0	4.838853	-11.854659	-2.822853
108	6	0	2.439715	-10.787325	-1.806067
109	6	0	2.421563	-11.832906	-2.771586
110	6	0	3.592783	-12.360967	-3.267568
111	6	0	2.506847	-8.757385	0.132868
112	6	0	1.245529	-9.169414	-0.403752
113	6	0	1.242524	-10.180426	-1.345303
114	6	0	5.001830	-8.623643	0.106020
115	6	0	5.295242	-7.317214	-0.260331
116	6	0	6.406772	-6.597853	0.284641
117	6	0	7.272791	-7.281266	1.116727
118	1	0	8.860833	-8.815765	2.664310
119	1	0	8.427091	-11.150293	3.353316
120	1	0	1.463523	-12.202078	-3.116037
121	1	0	3.566062	-13.155777	-4.000875
122	1	0	0.298187	-10.520599	-1.751128
123	1	0	8.131470	-6.762989	1.524401
124	6	0	3.714573	-9.249861	-0.342105
125	1	0	-6.368550	12.312259	2.573076
126	1	0	-5.756478	12.263455	-3.224777
127	1	0	5.756478	-12.263455	-3.224777
128	1	0	6.368550	-12.312259	2.573076
129	1	0	5.847893	-10.469223	-1.550383
130	1	0	4.781990	-11.171078	1.085546
131	1	0	-4.781990	11.171078	1.085546
132	1	0	-5.847893	10.469223	-1.550383
133	8	0	2.456114	-7.699797	1.058894
134	8	0	-2.456114	7.699797	1.058894

135	8	0	-4.422338	6.565325	-1.067188
136	8	0	4.422338	-6.565325	-1.067188
137	6	0	4.178280	-6.943845	-2.425335
138	1	0	3.777628	-7.955428	-2.502217
139	1	0	3.459874	-6.207436	-2.782569
140	6	0	2.962580	-7.873808	2.385625
141	1	0	4.012862	-8.168556	2.386462
142	1	0	2.826317	-6.900061	2.853850
143	6	0	-2.962580	7.873808	2.385625
144	1	0	-4.012862	8.168556	2.386462
145	1	0	-2.826317	6.900061	2.853850
146	6	0	-4.178280	6.943845	-2.425335
147	1	0	-3.777628	7.955428	-2.502217
148	1	0	-3.459874	6.207436	-2.782569
149	8	0	5.423847	-6.864123	-3.155675
150	8	0	2.177420	-8.892201	3.047571
151	8	0	-2.177420	8.892201	3.047571
152	8	0	-5.423847	6.864123	-3.155675
153	6	0	2.771557	-9.395225	4.276588
154	1	0	2.113347	-10.184934	4.624880
155	1	0	3.769085	-9.804072	4.092520
156	1	0	2.833316	-8.612931	5.038827
157	6	0	5.393740	-7.516549	-4.455680
158	1	0	6.399153	-7.431061	-4.855681
159	1	0	5.126191	-8.572569	-4.359314
160	1	0	4.691183	-7.022603	-5.133320
161	6	0	-2.771557	9.395225	4.276588
162	1	0	-2.113347	10.184934	4.624880
163	1	0	-3.769085	9.804072	4.092520
164	1	0	-2.833316	8.612931	5.038827
165	6	0	-5.393740	7.516549	-4.455680
166	1	0	-6.399153	7.431061	-4.855681
167	1	0	-5.126191	8.572569	-4.359314
168	1	0	-4.691183	7.022603	-5.133320

Table S 15: Coordinates of (*R*,*S*)-**14** for the Figure S69.

Standard orientation:

Center Number	Atomic Number	Atomic Type	Coordinates (Angstroms) X	Y	Z
1	6	0	2.788053	1.215985	11.207912
2	6	0	3.440453	1.994533	12.205314
3	6	0	2.737731	2.522402	13.265089
4	6	0	1.378039	0.966559	11.327678
5	6	0	0.678047	1.556555	12.417154
6	6	0	1.340467	2.308429	13.363505
7	6	0	-0.678047	-1.556555	12.417154

8	6	0	-1.378039	-0.966559	11.327678
9	6	0	-1.340467	-2.308429	13.363505
10	6	0	-2.788053	-1.215985	11.207912
11	6	0	-3.440453	-1.994533	12.205314
12	6	0	-2.737731	-2.522402	13.265089
13	6	0	-1.486032	0.345111	9.296033
14	6	0	-2.844523	-0.030274	9.068893
15	6	0	-3.480835	-0.761028	10.054530
16	6	0	0.733964	0.142987	10.347630
17	6	0	1.486032	-0.345111	9.296033
18	6	0	2.844523	0.030274	9.068893
19	6	0	3.480835	0.761028	10.054530
20	1	0	4.504238	2.173991	12.108374
21	1	0	3.245495	3.112173	14.016832
22	1	0	-4.504238	-2.173991	12.108374
23	1	0	-3.245495	-3.112173	14.016832
24	1	0	-4.507699	-1.072916	9.908806
25	1	0	4.507699	1.072916	9.908806
26	6	0	-0.733964	-0.142987	10.347630
27	6	0	-2.788053	-1.215985	-11.207912
28	6	0	-3.440453	-1.994533	-12.205314
29	6	0	-2.737731	-2.522402	-13.265089
30	6	0	-1.378039	-0.966559	-11.327678
31	6	0	-1.340467	-2.308429	-13.363505
32	6	0	1.378039	0.966559	-11.327678
33	6	0	1.340467	2.308429	-13.363505
34	6	0	2.788053	1.215985	-11.207912
35	6	0	3.440453	1.994533	-12.205314
36	6	0	2.737731	2.522402	-13.265089
37	6	0	1.486032	-0.345111	-9.296033
38	6	0	2.844523	0.030274	-9.068893
39	6	0	3.480835	0.761028	-10.054530
40	6	0	-0.733964	-0.142987	-10.347630
41	6	0	-1.486032	0.345111	-9.296033
42	6	0	-2.844523	-0.030274	-9.068893
43	6	0	-3.480835	-0.761028	-10.054530
44	1	0	-4.504238	-2.173991	-12.108374
45	1	0	-3.245495	-3.112173	-14.016832
46	1	0	4.504238	2.173991	-12.108374
47	1	0	3.245495	3.112173	-14.016832
48	1	0	4.507699	1.072916	-9.908806
49	1	0	-4.507699	-1.072916	-9.908806
50	6	0	0.733964	0.142987	-10.347630
51	6	0	0.678047	1.556555	-12.417154
52	6	0	-0.678047	-1.556555	-12.417154
53	6	0	3.459027	-0.176124	-7.727378
54	6	0	4.757870	-0.669442	-7.523272
55	6	0	2.713777	0.233080	-6.599789
56	6	0	5.296271	-0.753703	-6.233129
57	1	0	5.345509	-0.996012	-8.371817

58	6	0	3.252412	0.165513	-5.328164
59	1	0	1.711908	0.608111	-6.744622
60	6	0	4.554868	-0.329540	-5.118449
61	1	0	6.298444	-1.143134	-6.096567
62	1	0	2.690115	0.495795	-4.467760
63	6	0	-3.459027	0.176124	7.727378
64	6	0	-4.757870	0.669442	7.523272
65	6	0	-2.713777	-0.233080	6.599789
66	6	0	-5.296271	0.753703	6.233129
67	1	0	-5.345509	0.996012	8.371817
68	6	0	-3.252412	-0.165513	5.328164
69	1	0	-1.711908	-0.608111	6.744622
70	6	0	-4.554868	0.329540	5.118449
71	1	0	-6.298444	1.143134	6.096567
72	1	0	-2.690115	-0.495795	4.467760
73	6	0	3.459027	-0.176124	7.727378
74	6	0	2.713777	0.233080	6.599789
75	6	0	4.757870	-0.669442	7.523272
76	6	0	3.252412	0.165513	5.328164
77	1	0	1.711908	0.608111	6.744622
78	6	0	5.296271	-0.753703	6.233129
79	1	0	5.345509	-0.996012	8.371817
80	6	0	4.554868	-0.329540	5.118449
81	1	0	2.690115	0.495795	4.467760
82	1	0	6.298444	-1.143134	6.096567
83	6	0	-3.459027	0.176124	-7.727378
84	6	0	-2.713777	-0.233080	-6.599789
85	6	0	-4.757870	0.669442	-7.523272
86	6	0	-3.252412	-0.165513	-5.328164
87	1	0	-1.711908	-0.608111	-6.744622
88	6	0	-5.296271	0.753703	-6.233129
89	1	0	-5.345509	0.996012	-8.371817
90	6	0	-4.554868	0.329540	-5.118449
91	1	0	-2.690115	-0.495795	-4.467760
92	1	0	-6.298444	1.143134	-6.096567
93	6	0	-5.087570	0.379691	-3.748204
94	1	0	-6.047499	0.886989	-3.594570
95	6	0	-5.087570	0.379691	3.748204
96	1	0	-6.047499	0.886989	3.594570
97	6	0	5.087570	-0.379691	-3.748204
98	1	0	6.047499	-0.886989	-3.594570
99	6	0	5.087570	-0.379691	3.748204
100	1	0	6.047499	-0.886989	3.594570
101	7	0	-4.422838	-0.171918	2.794114
102	7	0	4.422838	0.171918	-2.794114
103	7	0	-4.422838	-0.171918	-2.794114
104	7	0	4.422838	0.171918	2.794114
105	6	0	4.672268	0.148387	-1.413026
106	6	0	-4.672268	-0.148387	1.413026
107	6	0	5.179648	-0.953927	-0.698930

108	6	0	-5.179648	0.953927	0.698930
109	6	0	4.672268	0.148387	1.413026
110	6	0	-4.672268	-0.148387	-1.413026
111	6	0	5.179648	-0.953927	0.698930
112	6	0	-5.179648	0.953927	-0.698930
113	6	0	4.212745	1.268711	-0.690830
114	6	0	-4.212745	-1.268711	0.690830
115	6	0	4.212745	1.268711	0.690830
116	6	0	-4.212745	-1.268711	-0.690830
117	1	0	5.498572	-1.839627	1.233044
118	1	0	-5.498572	1.839627	1.233044
119	1	0	-3.804788	-2.098147	1.250053
120	1	0	-3.804788	-2.098147	-1.250053
121	1	0	-5.498572	1.839627	-1.233044
122	1	0	5.498572	-1.839627	-1.233044
123	1	0	3.804788	2.098147	-1.250053
124	1	0	3.804788	2.098147	1.250053
125	1	0	-0.789743	-2.745311	14.186194
126	1	0	0.389223	-1.411708	12.489418
127	1	0	-0.389223	1.411708	12.489418
128	1	0	0.789743	2.745311	14.186194
129	1	0	0.389223	-1.411708	-12.489418
130	1	0	0.789743	2.745311	-14.186194
131	1	0	-0.789743	-2.745311	-14.186194
132	1	0	-0.389223	1.411708	-12.489418
133	6	0	1.255043	-2.546037	8.305129
134	1	0	0.776668	-2.957675	7.416194
135	1	0	2.345576	-2.631474	8.237131
136	6	0	-1.255043	2.546037	8.305129
137	1	0	-0.776668	2.957675	7.416194
138	1	0	-2.345576	2.631474	8.237131
139	6	0	1.255043	-2.546037	-8.305129
140	1	0	2.345576	-2.631474	-8.237131
141	1	0	0.776668	-2.957675	-7.416194
142	6	0	-1.255043	2.546037	-8.305129
143	1	0	-2.345576	2.631474	-8.237131
144	1	0	-0.776668	2.957675	-7.416194
145	6	0	1.265938	-4.556490	9.658412
146	1	0	0.861546	-4.909060	10.601680
147	1	0	0.910956	-5.202378	8.848721
148	1	0	2.360341	-4.592754	9.693808
149	6	0	-1.265938	4.556490	9.658412
150	1	0	-0.861546	4.909060	10.601680
151	1	0	-0.910956	5.202378	8.848721
152	1	0	-2.360341	4.592754	9.693808
153	6	0	1.265938	-4.556490	-9.658412
154	1	0	0.910956	-5.202378	-8.848721
155	1	0	0.861546	-4.909060	-10.601680
156	1	0	2.360341	-4.592754	-9.693808
157	6	0	-1.265938	4.556490	-9.658412

158	1	0	-0.910956	5.202378	-8.848721
159	1	0	-0.861546	4.909060	-10.601680
160	1	0	-2.360341	4.592754	-9.693808
161	8	0	-0.846460	1.175834	8.345762
162	8	0	0.846460	-1.175834	8.345762
163	8	0	0.846460	-1.175834	-8.345762
164	8	0	-0.846460	1.175834	-8.345762
165	8	0	-0.786001	3.196237	9.502385
166	8	0	0.786001	-3.196237	9.502385
167	8	0	0.786001	-3.196237	-9.502385
168	8	0	-0.786001	3.196237	-9.502385

Table S 16: Coordinates of (*R*,*R*)-**15** for the Figure S71.

Standard orientation:

Center Number	Atomic Number	Atomic Type	Coordinates (Angstroms) X	Y	Z
1	6	0	7.644546	4.751394	0.105118
2	6	0	6.969574	5.610349	1.002861
3	6	0	6.972868	4.328108	-1.062609
4	6	0	5.651999	5.980911	0.772648
5	1	0	7.482968	5.949897	1.891849
6	6	0	5.654548	4.702252	-1.285449
7	1	0	7.496567	3.688392	-1.758429
8	6	0	4.963372	5.509855	-0.362222
9	1	0	5.119188	6.606261	1.474540
10	1	0	5.138876	4.343398	-2.168071
11	6	0	-7.644546	-4.751394	0.105118
12	6	0	-6.969574	-5.610349	1.002861
13	6	0	-6.972868	-4.328108	-1.062609
14	6	0	-5.651999	-5.980911	0.772648
15	1	0	-7.482968	-5.949897	1.891849
16	6	0	-5.654548	-4.702252	-1.285449
17	1	0	-7.496567	-3.688392	-1.758429
18	6	0	-4.963372	-5.509855	-0.362222
19	1	0	-5.119188	-6.606261	1.474540
20	1	0	-5.138876	-4.343398	-2.168071
21	6	0	-7.644546	4.751394	-0.105118
22	6	0	-6.972868	4.328108	1.062609
23	6	0	-6.969574	5.610349	-1.002861
24	6	0	-5.654548	4.702252	1.285449
25	1	0	-7.496567	3.688392	1.758429
26	6	0	-5.651999	5.980911	-0.772648
27	1	0	-7.482968	5.949897	-1.891849
28	6	0	-4.963372	5.509855	0.362222
29	1	0	-5.138876	4.343398	2.168071
30	1	0	-5.119188	6.606261	-1.474540

31	6	0	7.644546	-4.751394	-0.105118
32	6	0	6.972868	-4.328108	1.062609
33	6	0	6.969574	-5.610349	-1.002861
34	6	0	5.654548	-4.702252	1.285449
35	1	0	7.496567	-3.688392	1.758429
36	6	0	5.651999	-5.980911	-0.772648
37	1	0	7.482968	-5.949897	-1.891849
38	6	0	4.963372	-5.509855	0.362222
39	1	0	5.138876	-4.343398	2.168071
40	1	0	5.119188	-6.606261	-1.474540
41	6	0	3.536922	-5.753591	0.561340
42	1	0	3.101914	-5.277777	1.446768
43	6	0	-3.536922	-5.753591	-0.561340
44	1	0	-3.101914	-5.277777	-1.446768
45	6	0	-3.536922	5.753591	0.561340
46	1	0	-3.101914	5.277777	1.446768
47	6	0	3.536922	5.753591	-0.561340
48	1	0	3.101914	5.277777	-1.446768
49	7	0	-2.804921	-6.408791	0.278221
50	7	0	2.804921	-6.408791	-0.278221
51	7	0	-2.804921	6.408791	-0.278221
52	7	0	2.804921	6.408791	0.278221
53	6	0	-1.407721	-6.538218	0.091860
54	6	0	1.407721	-6.538218	-0.091860
55	6	0	-1.407721	6.538218	-0.091860
56	6	0	1.407721	6.538218	0.091860
57	6	0	-0.614027	-6.599973	1.250592
58	6	0	0.614027	-6.599973	-1.250592
59	6	0	-0.614027	6.599973	-1.250592
60	6	0	0.614027	6.599973	1.250592
61	6	0	0.773511	-6.588463	1.165186
62	6	0	-0.773511	-6.588463	-1.165186
63	6	0	-0.773511	6.588463	1.165186
64	6	0	0.773511	6.588463	-1.165186
65	1	0	1.365528	-6.626245	2.069554
66	1	0	-1.115071	-6.625912	2.207606
67	1	0	-1.365528	-6.626245	-2.069554
68	1	0	1.115071	-6.625912	-2.207606
69	1	0	1.115071	6.625912	2.207606
70	1	0	-1.365528	6.626245	2.069554
71	1	0	-1.115071	6.625912	-2.207606
72	1	0	1.365528	6.626245	-2.069554
73	6	0	-13.151687	-2.246368	2.027671
74	6	0	-14.132394	-2.566858	3.009732
75	6	0	-15.137495	-1.680501	3.324652
76	6	0	-13.229730	-0.983775	1.346180
77	6	0	-14.273062	-0.085386	1.710315
78	6	0	-15.201878	-0.424941	2.672016
79	6	0	-14.273062	0.085386	-1.710315
80	6	0	-13.229730	0.983775	-1.346180

81	6	0	-15.201878	0.424941	-2.672016
82	6	0	-13.151687	2.246368	-2.027671
83	6	0	-14.132394	2.566858	-3.009732
84	6	0	-15.137495	1.680501	-3.324652
85	6	0	-11.240942	1.578641	-0.078293
86	6	0	-11.116576	2.815396	-0.793563
87	6	0	-12.081734	3.127842	-1.739465
88	6	0	-12.242186	-0.662561	0.351306
89	6	0	-11.240942	-1.578641	0.078293
90	6	0	-11.116576	-2.815396	0.793563
91	6	0	-12.081734	-3.127842	1.739465
92	1	0	-14.067370	-3.524390	3.511063
93	1	0	-15.875833	-1.935934	4.072721
94	1	0	-14.067370	3.524390	-3.511063
95	1	0	-15.875833	1.935934	-4.072721
96	1	0	-12.004722	4.060168	-2.282581
97	1	0	-12.004722	-4.060168	2.282581
98	6	0	-12.242186	0.662561	-0.351306
99	6	0	13.151687	2.246368	2.027671
100	6	0	14.132394	2.566858	3.009732
101	6	0	15.137495	1.680501	3.324652
102	6	0	13.229730	0.983775	1.346180
103	6	0	14.273062	0.085386	1.710315
104	6	0	15.201878	0.424941	2.672016
105	6	0	14.273062	-0.085386	-1.710315
106	6	0	13.229730	-0.983775	-1.346180
107	6	0	15.201878	-0.424941	-2.672016
108	6	0	13.151687	-2.246368	-2.027671
109	6	0	14.132394	-2.566858	-3.009732
110	6	0	15.137495	-1.680501	-3.324652
111	6	0	11.240942	-1.578641	-0.078293
112	6	0	11.116576	-2.815396	-0.793563
113	6	0	12.081734	-3.127842	-1.739465
114	6	0	12.242186	0.662561	0.351306
115	6	0	11.240942	1.578641	0.078293
116	6	0	11.116576	2.815396	0.793563
117	6	0	12.081734	3.127842	1.739465
118	1	0	14.067370	3.524390	3.511063
119	1	0	15.875833	1.935934	4.072721
120	1	0	14.067370	-3.524390	-3.511063
121	1	0	15.875833	-1.935934	-4.072721
122	1	0	12.004722	-4.060168	-2.282581
123	1	0	12.004722	4.060168	2.282581
124	6	0	12.242186	-0.662561	-0.351306
125	1	0	-15.988058	0.272162	2.929749
126	1	0	-15.988058	-0.272162	-2.929749
127	1	0	15.988058	0.272162	-2.929749
128	1	0	15.988058	-0.272162	2.929749
129	1	0	14.334380	0.874263	-1.218301
130	1	0	14.334380	-0.874263	1.218301

131	1	0	-14.334380	0.874263	1.218301
132	1	0	-14.334380	-0.874263	-1.218301
133	8	0	10.217658	-1.263873	0.828232
134	8	0	-10.217658	1.263873	0.828232
135	8	0	-10.217658	-1.263873	-0.828232
136	8	0	10.217658	1.263873	-0.828232
137	6	0	10.469582	1.361835	-2.232269
138	1	0	11.455641	0.960083	-2.485602
139	1	0	9.676560	0.780798	-2.701975
140	6	0	10.469582	-1.361835	2.232269
141	1	0	11.455641	-0.960083	2.485602
142	1	0	9.676560	-0.780798	2.701975
143	6	0	-10.469582	1.361835	2.232269
144	1	0	-11.455641	0.960083	2.485602
145	1	0	-9.676560	0.780798	2.701975
146	6	0	-10.469582	-1.361835	-2.232269
147	1	0	-11.455641	-0.960083	-2.485602
148	1	0	-9.676560	-0.780798	-2.701975
149	8	0	10.395353	2.750957	-2.616722
150	8	0	10.395353	-2.750957	2.616722
151	8	0	-10.395353	2.750957	2.616722
152	8	0	-10.395353	-2.750957	-2.616722
153	6	0	10.867539	-3.009189	3.966731
154	1	0	10.795192	-4.082717	4.108006
155	1	0	11.908765	-2.693743	4.090413
156	1	0	10.247555	-2.502633	4.713199
157	6	0	10.867539	3.009189	-3.966731
158	1	0	10.795192	4.082717	-4.108006
159	1	0	11.908765	2.693743	-4.090413
160	1	0	10.247555	2.502633	-4.713199
161	6	0	-10.867539	3.009189	3.966731
162	1	0	-10.795192	4.082717	4.108006
163	1	0	-11.908765	2.693743	4.090413
164	1	0	-10.247555	2.502633	4.713199
165	6	0	-10.867539	-3.009189	-3.966731
166	1	0	-10.795192	-4.082717	-4.108006
167	1	0	-11.908765	-2.693743	-4.090413
168	1	0	-10.247555	-2.502633	-4.713199
169	6	0	9.977732	3.634697	0.573117
170	6	0	8.937223	4.227934	0.382467
171	6	0	9.977732	-3.634697	-0.573117
172	6	0	8.937223	-4.227934	-0.382467
173	6	0	-9.977732	3.634697	-0.573117
174	6	0	-8.937223	4.227934	-0.382467
175	6	0	-9.977732	-3.634697	0.573117
176	6	0	-8.937223	-4.227934	0.382467

Table S 17: Coordinates of (*R*,*S*)-**15** for the Figure S72.

Standard orientation:

Center Number	Atomic Number	Atomic Type	Coordinates (Angstroms) X	Y	Z
1	6	0	2.299259	-1.973640	13.531334
2	6	0	3.304904	-2.182749	14.518802
3	6	0	3.503046	-1.271745	15.530794
4	6	0	1.476013	-0.798225	13.607086
5	6	0	1.726887	0.133121	14.652491
6	6	0	2.708972	-0.099128	15.591331
7	6	0	-1.726887	-0.133121	14.652491
8	6	0	-1.476013	0.798225	13.607086
9	6	0	-2.708972	0.099128	15.591331
10	6	0	-2.299259	1.973640	13.531334
11	6	0	-3.304904	2.182749	14.518802
12	6	0	-3.503046	1.271745	15.530794
13	6	0	-0.279774	1.542054	11.633882
14	6	0	-1.160420	2.662914	11.482513
15	6	0	-2.131446	2.872601	12.451125
16	6	0	0.439285	-0.605828	12.634773
17	6	0	0.279774	-1.542054	11.633882
18	6	0	1.160420	-2.662914	11.482513
19	6	0	2.131446	-2.872601	12.451125
20	1	0	3.916924	-3.073845	14.453322
21	1	0	4.268745	-1.441561	16.276072
22	1	0	-3.916924	3.073845	14.453322
23	1	0	-4.268745	1.441561	16.276072
24	1	0	-2.800583	3.717576	12.356827
25	1	0	2.800583	-3.717576	12.356827
26	6	0	-0.439285	0.605828	12.634773
27	6	0	-2.298522	1.974466	-13.531364
28	6	0	-3.304070	2.183930	-14.518856
29	6	0	-3.502505	1.272999	-15.530857
30	6	0	-1.475696	0.798758	-13.607091
31	6	0	-2.708838	0.100107	-15.591380
32	6	0	1.475696	-0.798758	-13.607091
33	6	0	2.708838	-0.100107	-15.591380
34	6	0	2.298522	-1.974466	-13.531364
35	6	0	3.304070	-2.183930	-14.518856
36	6	0	3.502505	-1.272999	-15.530857
37	6	0	0.279230	-1.542168	-11.633861
38	6	0	1.159478	-2.663345	-11.482521
39	6	0	2.130414	-2.873367	-12.451152
40	6	0	-0.439068	0.605985	-12.634744
41	6	0	-0.279230	1.542168	-11.633861
42	6	0	-1.159478	2.663345	-11.482521
43	6	0	-2.130414	2.873367	-12.451152

44	1	0	-3.915780	3.075240	-14.453389
45	1	0	-4.268126	1.443086	-16.276153
46	1	0	3.915780	-3.075240	-14.453389
47	1	0	4.268126	-1.443086	-16.276153
48	1	0	2.799267	-3.718566	-12.356858
49	1	0	-2.799267	3.718566	-12.356858
50	6	0	0.439068	-0.605985	-12.634744
51	6	0	1.726862	0.132492	-14.652514
52	6	0	-1.726862	-0.132492	-14.652514
53	6	0	0.967976	-4.382817	-7.839254
54	6	0	1.699038	-5.474029	-7.327101
55	6	0	0.166811	-3.628161	-6.946460
56	6	0	1.658876	-5.772818	-5.967068
57	1	0	2.309612	-6.063594	-7.996642
58	6	0	0.129279	-3.932526	-5.597664
59	1	0	-0.390440	-2.786365	-7.333071
60	6	0	0.889257	-5.000211	-5.078706
61	1	0	2.245102	-6.600088	-5.585035
62	1	0	-0.458502	-3.343130	-4.909659
63	6	0	-0.968996	4.382485	7.839297
64	6	0	-1.700003	5.473713	7.327101
65	6	0	-0.167752	3.627843	6.946562
66	6	0	-1.659702	5.772539	5.967080
67	1	0	-2.310634	6.063269	7.996598
68	6	0	-0.130082	3.932246	5.597778
69	1	0	0.389459	2.786037	7.333208
70	6	0	-0.889994	4.999955	5.078774
71	1	0	-2.245878	6.599829	5.585012
72	1	0	0.457770	3.342869	4.909817
73	6	0	0.968996	-4.382485	7.839297
74	6	0	0.167752	-3.627843	6.946562
75	6	0	1.700003	-5.473713	7.327101
76	6	0	0.130082	-3.932246	5.597778
77	1	0	-0.389459	-2.786037	7.333208
78	6	0	1.659702	-5.772539	5.967080
79	1	0	2.310634	-6.063269	7.996598
80	6	0	0.889994	-4.999955	5.078774
81	1	0	-0.457770	-3.342869	4.909817
82	1	0	2.245878	-6.599829	5.585012
83	6	0	-0.967976	4.382817	-7.839254
84	6	0	-0.166811	3.628161	-6.946460
85	6	0	-1.699038	5.474029	-7.327101
86	6	0	-0.129279	3.932526	-5.597664
87	1	0	0.390440	2.786365	-7.333071
88	6	0	-1.658876	5.772818	-5.967068
89	1	0	-2.309612	6.063594	-7.996642
90	6	0	-0.889257	5.000211	-5.078706
91	1	0	0.458502	3.343130	-4.909659
92	1	0	-2.245102	6.600088	-5.585035
93	6	0	-0.927312	5.250083	-3.636270

94	1	0	-1.575864	6.066678	-3.299699
95	6	0	-0.927873	5.249887	3.636344
96	1	0	-1.576364	6.066511	3.299730
97	6	0	0.927312	-5.250083	-3.636270
98	1	0	1.575864	-6.066678	-3.299699
99	6	0	0.927873	-5.249887	3.636344
100	1	0	1.576364	-6.066511	3.299730
101	7	0	-0.272918	4.499020	2.816297
102	7	0	0.272471	-4.499176	-2.816169
103	7	0	-0.272471	4.499176	-2.816169
104	7	0	0.272918	-4.499020	2.816297
105	6	0	0.285168	-4.625454	-1.412057
106	6	0	-0.285397	4.625377	1.412189
107	6	0	0.522262	-5.816693	-0.696737
108	6	0	-0.522376	5.816655	0.696896
109	6	0	0.285397	-4.625377	1.412189
110	6	0	-0.285168	4.625454	-1.412057
111	6	0	0.522376	-5.816655	0.696896
112	6	0	-0.522262	5.816693	-0.696737
113	6	0	-0.000056	-3.450699	-0.692677
114	6	0	-0.000056	3.450662	0.692791
115	6	0	0.000056	-3.450662	0.692791
116	6	0	0.000056	3.450699	-0.692677
117	1	0	0.676838	-6.746311	1.228732
118	1	0	-0.676838	6.746311	1.228732
119	1	0	0.217225	2.554689	1.255540
120	1	0	0.217428	2.554756	-1.255439
121	1	0	-0.676639	6.746377	-1.228549
122	1	0	0.676639	-6.746377	-1.228549
123	1	0	-0.217428	-2.554756	-1.255439
124	1	0	-0.217225	-2.554689	1.255540
125	1	0	-2.880343	-0.621392	16.380279
126	1	0	-1.138568	-1.036870	14.696191
127	1	0	1.138568	1.036870	14.696191
128	1	0	2.880343	0.621392	16.380279
129	1	0	-1.138858	-1.036446	-14.696203
130	1	0	2.880439	0.620348	-16.380338
131	1	0	-2.880439	-0.620348	-16.380338
132	1	0	1.138858	1.036446	-14.696203
133	6	0	-1.854329	-2.207608	10.681721
134	1	0	-2.407987	-1.961870	9.775385
135	1	0	-1.549017	-3.259612	10.682499
136	6	0	1.854329	2.207608	10.681721
137	1	0	2.407987	1.961870	9.775385
138	1	0	1.549017	3.259612	10.682499
139	6	0	-1.855244	-2.206776	-10.681858
140	1	0	-1.550497	-3.258942	-10.682867
141	1	0	-2.408761	-1.960931	-9.775463
142	6	0	1.855244	2.206776	-10.681858
143	1	0	1.550497	3.258942	-10.682867

144	1	0	2.408761	1.960931	-9.775463
145	6	0	-3.732045	-2.841028	12.076821
146	1	0	-4.203472	-2.528970	13.003032
147	1	0	-4.461878	-2.786386	11.262681
148	1	0	-3.379397	-3.873496	12.175306
149	6	0	3.732045	2.841028	12.076821
150	1	0	4.203472	2.528970	13.003032
151	1	0	4.461878	2.786386	11.262681
152	1	0	3.379397	3.873496	12.175306
153	6	0	-3.733195	-2.838992	-12.077206
154	1	0	-4.463047	-2.784277	-11.263088
155	1	0	-4.204443	-2.526462	-13.003349
156	1	0	-3.380980	-3.871580	-12.175973
157	6	0	3.733195	2.838992	-12.077206
158	1	0	4.463047	2.784277	-11.263088
159	1	0	4.204443	2.526462	-13.003349
160	1	0	3.380980	3.871580	-12.175973
161	8	0	0.717791	1.336208	10.662524
162	8	0	-0.717791	-1.336208	10.662524
163	8	0	-0.718248	-1.335975	-10.662487
164	8	0	0.718248	1.335975	-10.662487
165	8	0	2.628751	1.922983	11.862588
166	8	0	-2.628751	-1.922983	11.862588
167	8	0	-2.629523	-1.921476	-11.862654
168	8	0	2.629523	1.921476	-11.862654
169	6	0	-1.093975	3.440418	10.292338
170	6	0	-1.048427	3.965346	9.197440
171	6	0	1.093975	-3.440418	10.292338
172	6	0	1.048427	-3.965346	9.197440
173	6	0	1.092851	-3.440783	-10.292314
174	6	0	1.047310	-3.965693	-9.197407
175	6	0	-1.092851	3.440783	-10.292314
176	6	0	-1.047310	3.965693	-9.197407

----

[1] Gaussian 16, Revision C.01, M. J. Frisch, G. W. Trucks, H. B. Schlegel, G.E. Scuseria, M. A. Robb, J. R. Cheeseman, G. Scalmani, V. Barone, G. A. Petersson, H. Nakatsuji, X. Li, M. Caricato, A. V. Marenich, J. Bloino, B. G. Janesko, R. Gomperts, B. Mennucci, H. P. Hratchian, J. V. Ortiz, A. F. Izmaylov, J. L. Sonnenberg, D. Williams-Young, F. Ding, F. Lipparini, F. Egidi, J. Goings, B. Peng, A. Petrone, T. Henderson, D. Ranasinghe, V. G. Zakrzewski, J. Gao, N. Rega, G. Zheng, W. Liang, M. Hada, M. Ehara, K. Toyota, R. Fukuda, J. Hasegawa, M. Ishida, T. Nakajima, Y. Honda, O. Kitao, H. Nakai, T. Vreven, K. Throssell, J. A. Montgomery Jr, J. E. Peralta, F. Ogliaro, M. Bearpark, J. J. Heyd, E. N. Brothers, K. N. Kudin, V. N. Staroverov, T. Keith, R. Kobayashi, J. Normand, K. Raghavachari, A. Rendell, J. C. Burant, S. S. Iyengar, J. Tomasi, M. Cossi, J. M. Millam, M. Klene, C. Adamo, R. Cammi, J. W. Ochterski, R. L. Martin, K. Morokuma, O. Farkas, J. B. Foresman, D. J. Fox, Gaussian, Inc., Wallingford CT, 2016.

[2] (a) A. D. Becke, *J. Chem. Phys*. **1993**, *98*, 1372; b) C. Lee, W. Yang and R. G. Parr, *Phys. Rev*. **1988**, *37*, 785.

[3] H. Ishitani, M. Ueno, S. Kobayashi, *J. Am. Chem. Soc*. **2000**, *122*, 8180.

[4] X. Wang, V. Ervithayasuporn, Y. Zhang and Y. Kawakami, *Chem. Commun.* **2011**, *47*, 1282.

[5] R. R. Milburn, S. M. S. Hussain, O. Prien, Z. Ahmed, V. Snieckus, *Org. Lett*. **2007**, *9*, 4403.
